# Oral small-molecule GLP-1RA safiglipron versus dapagliflozin in type 2 diabetes: a randomized, double-blind, active-comparator-controlled phase 3 trial

**DOI:** 10.1038/s41591-026-04713-y

**Published:** 2026-10-05

**Authors:** Lixin Guo, Dongni Yu, Yimei Xu, Guo Tang, Liang Peng, Chao Meng, Meng Zhao, Wen Hu, Debin Huang, Chaoyang Zeng, Lei Yu, Guangxiu Wu, Lihui Zhang, Yuan Gao, Tingyan Zhong, Zi Ye

**Affiliations:** 1 Department of Endocrinology, Beijing Hospital; National Center of Gerontology, National Clinical Research Center for Gerontology, The Key Laboratory of Geriatrics of NHC, Institute of Geriatric Medicine, Chinese Academy of Medical Sciences, Beijing, China; 2 Clinical Research and Development, Jiangsu Hengrui Pharmaceuticals, Shanghai, China; 3 Department of General Medicine, Central Hospital Affiliated to Shandong First Medical University, Jinan, China; 4 Department of Endocrinology, The Affiliated Hospital of Xuzhou Medical University, Xuzhou, China; 5 Department of Endocrinology and Metabolism, Huai’an Hospital Affiliated to Xuzhou Medical University, Huai’an, China; 6 Endocrine and Metabolic Diseases Department, The Third Hospital of Changsha, Changsha, China; 7 Department of Endocrinology, Yichang Central People’s Hospital, Yichang, China; 8 Department of Endocrinology, The First Affiliated Hospital of Bengbu Medical College, Bengbu, China; 9 Department of Endocrinology, Chongqing University Affiliated Fuling Hospital, Chongqing, China; 10 Department of Endocrinology, The Second Hospital of Hebei Medical University, Shijiazhuang, China; 11 Department of Endocrinology, Jingjiang People’s Hospital, Jingjiang, China

**Keywords:** Type 2 diabetes, Type 2 diabetes, Randomized controlled trials

## Abstract

Safiglipron is an oral small-molecule glucagon-like peptide-1 (GLP-1) receptor agonist administered without fasting or dietary restrictions. We evaluated its efficacy and safety versus dapagliflozin in OUTSTAND-2, a phase 3, multicenter, randomized, double-blind, double-dummy, active-comparator-controlled trial conducted at 98 sites in China. We randomized 810 adults (mean hemoglobin A1C (HbA1c) 8.60%, median diabetes duration 5.0 years, 35.7% women) to safiglipron 30 mg (*n* = 202), safiglipron 60 mg (*n* = 203), safiglipron 90 mg (*n* = 203) or dapagliflozin 10 mg (*n* = 202) for 32 weeks, followed by a 20-week active-treatment extension. For the primary endpoint, treatment differences versus dapagliflozin in change from baseline in HbA1c at week 32 under the primary non-inferiority estimand were −0.30% (95% confidence interval (CI): −0.51 to −0.09), −0.22% (−0.44 to −0.01) and −0.40% (−0.62 to −0.19) for safiglipron 30 mg, 60 mg and 90 mg, respectively, establishing non-inferiority for all three doses (all *P* < 0.0001; non-inferiority margin 0.4%). Under the treatment policy estimand, 90 mg met the prespecified statistical criterion for superiority (difference −0.25%; 95% CI: −0.45 to −0.05; one-sided *P* = 0.0067), whereas 60 mg did not (*P* = 0.0789), after which fixed-sequence testing stopped. HbA1c target attainment rates with safiglipron versus dapagliflozin were 54.9−63.5% versus 36.5% for HbA1c <7.0% and 38.9−48.6% versus 16.1% for HbA1c ≤6.5%. Mean body weight changes were −2.35%, −3.55%, −4.17% and −3.60% for safiglipron 30 mg, safiglipron 60 mg, safiglipron 90 mg and dapagliflozin, respectively. Fasting plasma glucose, seven-point self-monitored blood glucose profiles, waist circumference, HOMA-β, HOMA-IR and changes in treatment satisfaction showed varying degrees of improvement from baseline with both safiglipron and dapagliflozin. Rescue therapy was used by 0.5−1.5% versus 0% of participants, respectively. Gastrointestinal adverse events were more frequent with safiglipron and mostly mild or moderate; adverse events led to treatment discontinuation in 4.0%, 4.0%, 3.9% and 1.5% of participants, respectively. Safiglipron provides an effective oral treatment option for adults with type 2 diabetes inadequately controlled with metformin. ClinicalTrials.gov identifier: NCT06589765.

## Abstract (web summary)

Once-daily oral safiglipron is non-inferior to dapagliflozin for HbA1c reduction at all tested doses and superior at 90 mg, with greater improvements in glycemic control, similar modest weight loss and an acceptable safety profile in adults with type 2 diabetes.

## Main

Diabetes affects an estimated 589 million adults worldwide, more than 90% of whom have type 2 diabetes^1^. Progressive β cell dysfunction contributes to deterioration of glycemic control and often necessitates treatment intensification beyond metformin^2,3^. GLP-1 receptor agonists and sodium-glucose co-transporter 2 (SGLT2) inhibitors are the preferred pharmacologic additions in this setting, with both providing HbA1c-lowering and cardiorenal benefits, albeit through complementary mechanisms^4,5^. Current guidelines recommend both classes as preferred therapies for type 2 diabetes, in addition to metformin and, in individuals with established atherosclerotic cardiovascular disease, heart failure or chronic kidney disease, independently of metformin^6^.

Among GLP-1 receptor agonists, conventional peptide-based formulations require subcutaneous injection, which may limit adherence^7^. Oral semaglutide provides a non-injectable option but requires fasting administration with restricted water intake owing to its peptide-based absorption enhancer co-formulation^8^. Oral non-peptide small-molecule GLP-1 receptor agonists may avoid these peptide-related administration requirements. Orforglipron, the first oral non-peptide small-molecule GLP-1 receptor agonist approved by the US Food and Drug Administration, has demonstrated glycemic and body weight benefits across phase 3 trials, including an open-label comparison with dapagliflozin in ACHIEVE-2 (refs. ^9–11^). To our knowledge, however, no double-blind phase 3 trial has directly compared an oral small-molecule GLP-1 receptor agonist to an SGLT2 inhibitor.

Safiglipron (proposed International Nonproprietary Name (INN)^12^; HRS-7535) is an oral non-peptide small-molecule GLP-1 receptor agonist with high oral bioavailability^13^. In phase 2 trials, safiglipron reduced HbA1c in individuals with type 2 diabetes inadequately controlled with metformin^14^, body weight in adults with obesity^15^ and albuminuria in people with diabetic kidney disease^16^. We conducted the OUTSTAND-2 trial to evaluate the efficacy and safety of three doses of safiglipron as compared to dapagliflozin in adults with type 2 diabetes inadequately controlled with metformin monotherapy.

## Results

### Trial design and participants

**Extended Data Fig. 1:**
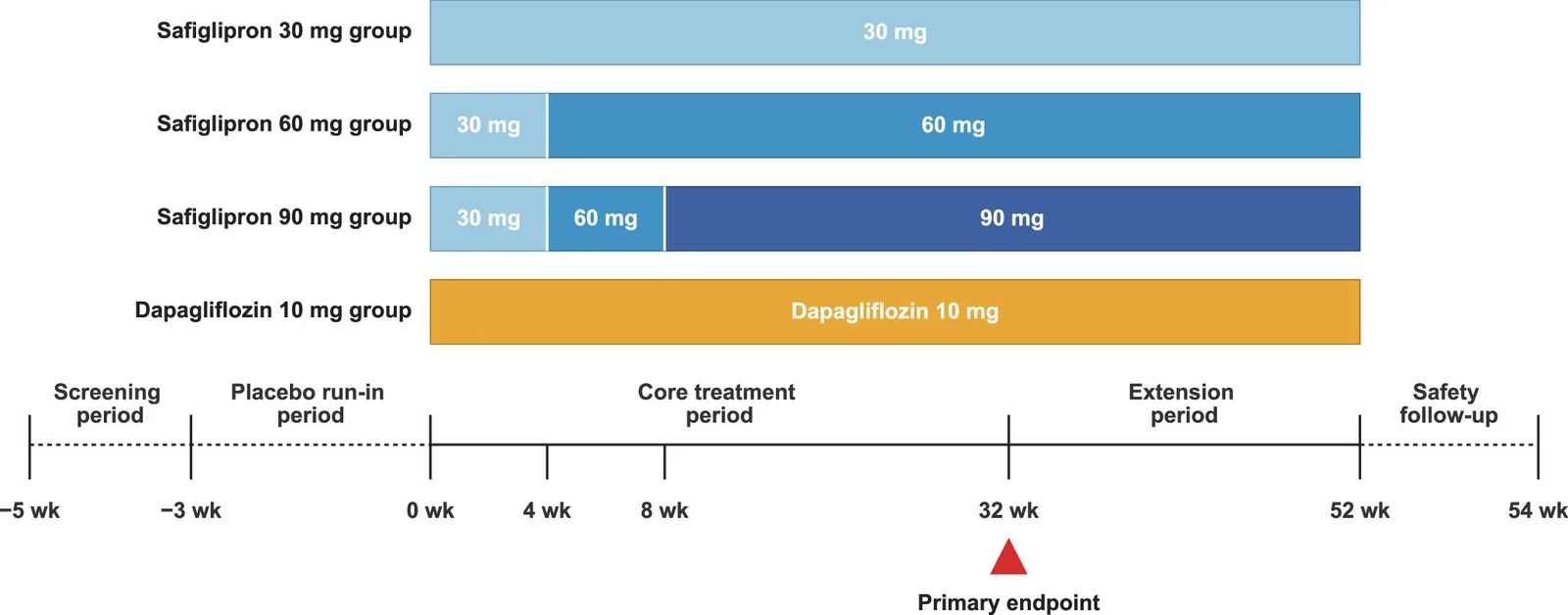
Trial design. The trial included screening, a 3-week single-blind run-in, 52 weeks of double-blind treatment comprising a 32-week core period and 20-week extension, and 2 weeks of safety follow-up. Safiglipron was initiated at 30 mg once daily; the 60-mg group escalated at week 5 and the 90-mg group at weeks 5 and 9. Dapagliflozin was given at 10 mg once daily. All participants continued stable metformin.

OUTSTAND-2 was a multicenter, randomized, double-blind, double-dummy, active-comparator-controlled trial evaluating once-daily oral safiglipron 30 mg, 60 mg and 90 mg versus dapagliflozin 10 mg in adults aged 18−75 years with type 2 diabetes (HbA1c 7.5−11.0%, body mass index (BMI) 19.0−40.0 kg m^−^^2^) inadequately controlled with stable metformin monotherapy. The trial comprised a 3-week single-blind run-in, a 32-week core treatment period, a 20-week extension and a 2-week safety follow-up (Extended Data Fig. 1). The primary endpoint was the change from baseline in HbA1c at week 32. Prespecified key secondary endpoints were HbA1c target achievement, fasting plasma glucose (FPG) and percentage change in body weight at week 32. Prespecified supportive secondary endpoints included changes in indices of β cell function and insulin sensitivity (HOMA-β and HOMA-IR), post-prandial glucose and insulin responses during a mixed-meal tolerance test (MMTT), waist circumference, seven-point self-monitored blood glucose (SMBG) profiles and the proportion receiving rescue therapy. Prespecified exploratory endpoints included changes in blood pressure, lipid parameters and urinary albumin-to-creatinine ratio (UACR). Patient-reported treatment satisfaction was assessed using Diabetes Treatment Satisfaction Questionnaire, status version (DTSQs). Safety endpoints included adverse events, hypoglycemic episodes, laboratory parameters, electrocardiograms and diabetic retinopathy assessments.

**Fig. 1:**
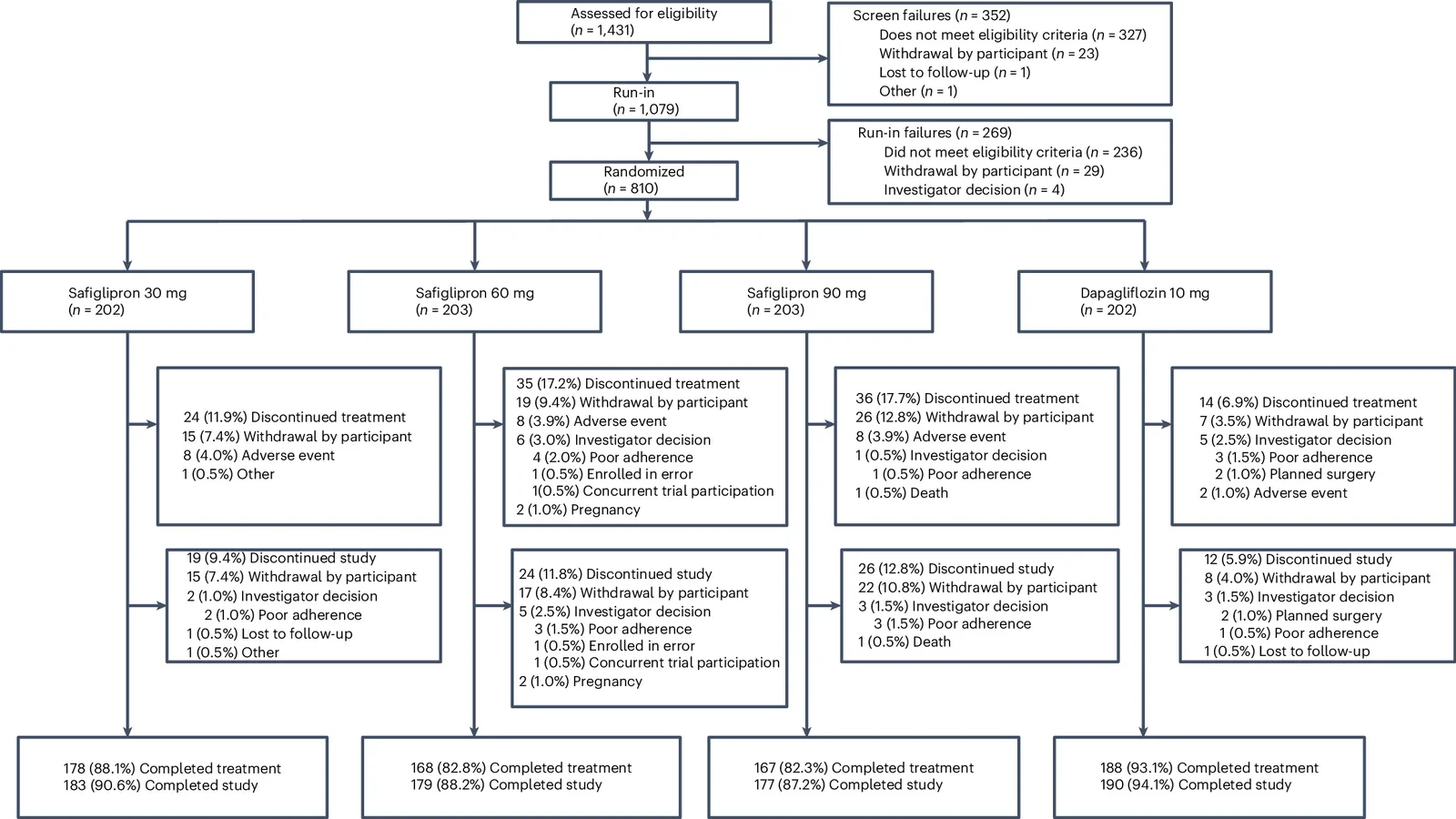
CONSORT diagram. Percentages use the corresponding randomized group size as the denominator. One participant in the safiglipron 60 mg group was found to fail eligibility criteria after randomization, and the investigator determined that no study drug would be administered, leading to immediate withdrawal from the trial.

Between 27 September 2024 and 30 April 2025, a total of 1,431 participants were screened, of whom 810 underwent randomization; 202 were assigned to receive safiglipron 30 mg, 203 to receive safiglipron 60 mg, 203 to receive safiglipron 90 mg and 202 to receive dapagliflozin (Fig. 1). Overall, 809 participants received at least one dose of the assigned study treatment and comprised the safety population. During the 52-week treatment period, 178 (88.1%) participants in the safiglipron 30 mg group, 168 (82.8%) participants in the safiglipron 60 mg group, 167 (82.3%) participants in the safiglipron 90 mg group and 188 (93.1%) participants in the dapagliflozin group completed treatment (Fig. 1). Prespecified intercurrent events are reported in Supplementary Table 2.

Among the 810 randomized participants, the mean age was 52.2 years, 64.3% of the participants were male and the mean body weight was 74.7 kg. The median duration of type 2 diabetes was 5.0 years, and the mean HbA1c level at baseline was 8.60%. The baseline demographic and clinical characteristics were generally balanced across the treatment groups (Table 1).

**Table 1:** Baseline characteristics of the intention-to-treat population

Characteristic	Safiglipron 30 mg (*N* = 202)	Safiglipron 60 mg (*N* = 203)	Safiglipron 90 mg (*N* = 203)	Dapagliflozin 10 mg (*N* = 202)	Total (*N* = 810)
Age, years	52.8 (10.81)	52.1 (10.46)	52.0 (10.80)	52.0 (10.75)	52.2 (10.69)
Age category, *n* (%)					
<65 years	174 (86.1)	181 (89.2)	177 (87.2)	175 (86.6)	707 (87.3)
≥65 years	28 (13.9)	22 (10.8)	26 (12.8)	27 (13.4)	103 (12.7)
Sex, *n* (%)					
Male	135 (66.8)	130 (64.0)	126 (62.1)	130 (64.4)	521 (64.3)
Female	67 (33.2)	73 (36.0)	77 (37.9)	72 (35.6)	289 (35.7)
Body weight, kg	74.11 (12.73)	74.74 (14.02)	75.28 (13.83)	74.61 (15.49)	74.68 (14.03)
BMI, kg m^−^^2^	26.97 (3.59)	27.07 (3.74)	27.27 (3.43)	27.18 (4.04)	27.12 (3.70)
Disease duration, years, median (Q1−Q3)	5.54 (2.9−8.1)	4.21 (2.0−8.7)	4.47 (2.4−9.0)	4.97 (2.7−8.1)	4.97 (2.5−8.2)
HbA1c, %	8.57 (0.78)	8.58 (0.71)	8.62 (0.78)	8.61 (0.78)	8.60 (0.76)
HbA1c category					
≤8.5%	108 (53.5)	109 (53.7)	109 (53.7)	108 (53.5)	434 (53.6)
>8.5%	94 (46.5)	94 (46.3)	94 (46.3)	94 (46.5)	376 (46.4)
FPG, mmol l^−1^	9.98 (1.73)	10.13 (1.98)	10.08 (1.80)	9.93 (1.75)	10.03 (1.82)
Fasting serum insulin, µIU ml^−1^	11.73 (8.19)	11.52 (8.04)	12.58 (8.98)	11.94 (8.08)	11.94 (8.32)
Fasting serum C-peptide, ng ml^−1^	2.02 (0.91)	2.12 (1.06)	2.18 (1.08)	2.14 (1.11)	2.12 (1.04)
HOMA-β, %, geometric mean (geometric s.d.)	31.22 (1.88)	30.42 (1.87)	32.90 (1.94)	31.88 (1.89)	31.59 (1.89)
HOMA-IR, geometric mean (geometric s.d.)	4.26 (1.91)	4.23 (1.93)	4.57 (1.93)	4.28 (2.00)	4.33 (1.94)
Triglycerides, mmol l^−1^	2.17 (1.57)	2.11 (1.53)	2.14 (1.24)	2.32 (1.54)	2.19 (1.47)
Non-HDL cholesterol, mmol l^−1^	3.60 (1.05)	3.69 (1.06)	3.54 (0.97)	3.61 (0.97)	3.61 (1.01)
UACR, mg g^−1^, median (Q1−Q3)	13.50 (4.0−47.0)	12.00 (4.0−46.0)	21.00 (5.0−67.0)	14.00 (4.5−44.0)	14.00 (4.0−50.0)
eGFR, ml min^−1^ 1.73 m^−2^	104.72 (14.42)	105.31 (12.99)	104.92 (15.78)	105.73 (14.57)	105.17 (14.45)
Blood pressure, mmHg					
Systolic	124.57 (11.91)	125.31 (12.27)	125.48 (12.02)	123.12 (11.62)	124.62 (11.97)
Diastolic	81.10 (7.07)	81.47 (7.34)	81.68 (8.32)	81.18 (7.62)	81.36 (7.59)
Heart rate, beats per minute	78.55 (11.32)	78.80 (10.88)	78.01 (9.38)	77.58 (10.83)	78.24 (10.62)

Data are presented as mean (s.d.) or *n* (%), unless otherwise stated.

HDL, high-density lipoprotein; Q1, first quartile; Q3, third quartile.

### Primary outcome

**Fig. 2:**
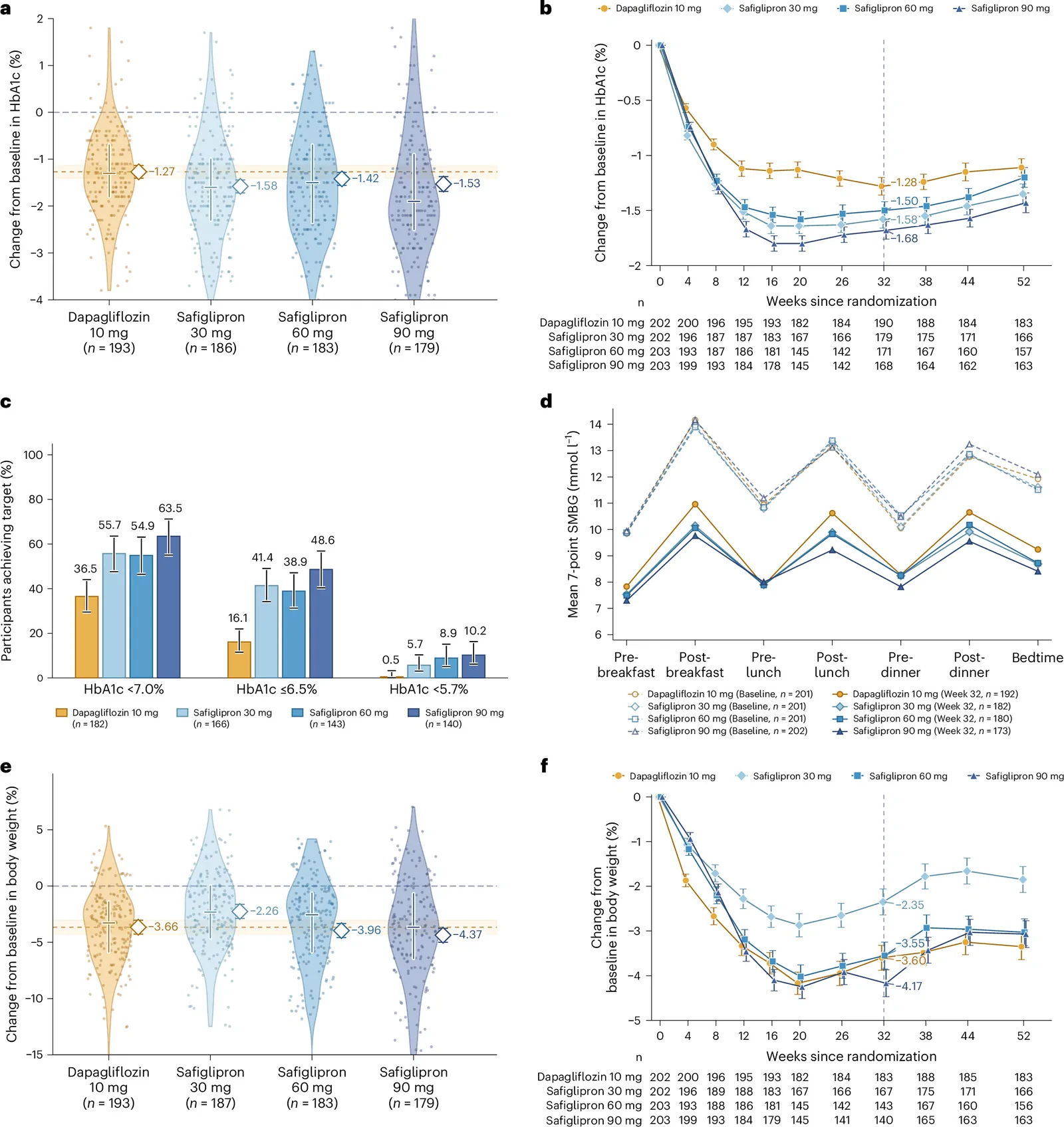
Glycemic and body weight outcomes. **a**, Change from baseline in HbA1c at week 32 under the treatment policy estimand. Violins and points show observed data, boxes show medians and interquartile ranges and diamonds show least-squares means with 95% CIs under the treatment policy estimand. **b**, Least-squares mean changes from baseline in HbA1c through week 52 under the efficacy estimand, except at week 32, for which results under the primary non-inferiority estimand are shown; error bars indicate standard errors. **c**, Model-estimated percentages of participants achieving the three HbA1c targets at week 32 under the efficacy estimand. Bars and numerical labels show GEE-estimated marginal probabilities expressed as percentages, and error bars indicate 95% CIs. Target attainment is a binary outcome for each participant at each threshold. **d**, Observed mean seven-point SMBG profiles at baseline and week 32, measured before and 2 hours after each meal and at bedtime. **e**, Percentage change from baseline in body weight at week 32, presented as in **a**. **f**, Least-squares mean percentage changes from baseline in body weight through week 52 under the efficacy estimand; error bars indicate standard errors. In **a** and **e**, the dashed line indicates the dapagliflozin least-squares mean. Vertical dashed lines in **b** and **f** mark week 32. Treatment policy estimates in **a** and **e** used all randomized participants; the efficacy estimand in **b**, **c** and **f** used a hypothetical strategy for intercurrent events. Exact *n* values are shown in each panel and denote participants with available observed data at the indicated visit. In **c**, these *n* values are not denominators used to calculate the model-estimated percentages. Analyses used the intention-to-treat population. MMRM was used in **a**, **b**, **e** and **f**, and GEE was used in **c**. Source data

**Extended Data Fig. 2:**
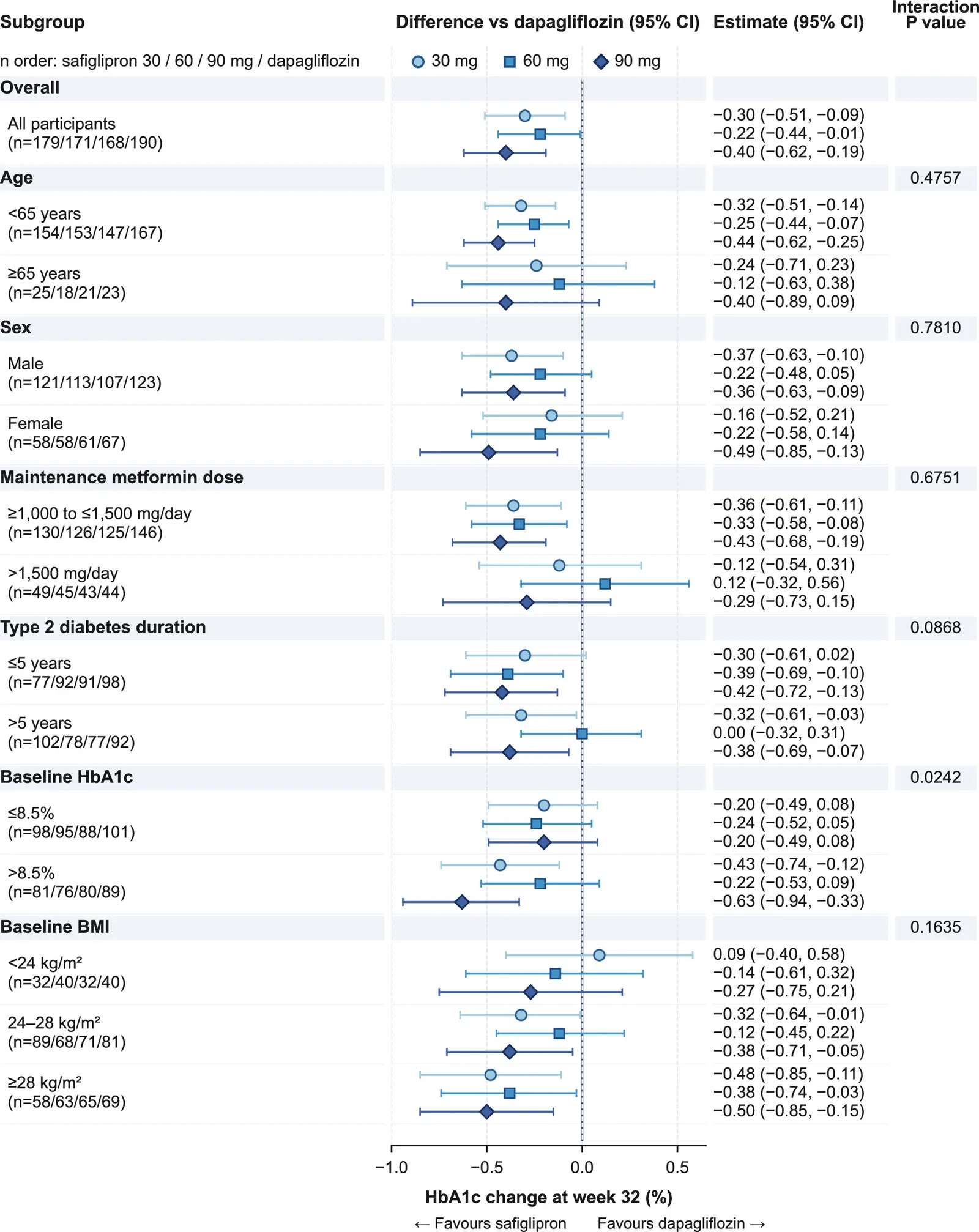
Prespecified subgroup analyses of the primary endpoint at week 32. Least-squares mean differences between each safiglipron dose and dapagliflozin are shown with 95% CIs for the overall population and prespecified subgroups. Estimates were derived in the intention-to-treat population under the primary non-inferiority estimand, with permanent treatment discontinuation and initiation of additional antihyperglycemic medication handled using hypothetical strategies. Global interaction P values were obtained using two-sided Type III joint F tests of the week-32 treatment-by-subgroup contrasts. All interaction P values were nominal and were not adjusted for multiplicity. CI=confidence interval; HbA1c=glycated hemoglobin. Source data

At week 32, least-squares mean changes from baseline in HbA1c under the primary non-inferiority estimand were −1.58% (95% CI: −1.73 to −1.43), −1.50% (−1.66 to −1.35) and −1.68% (−1.84 to −1.53) with safiglipron 30 mg, 60 mg and 90 mg, respectively, compared to −1.28% (−1.43 to −1.13) with dapagliflozin (Table 2). Treatment differences versus dapagliflozin were −0.30% (95% CI: −0.51 to −0.09), −0.22% (−0.44 to −0.01) and −0.40% (−0.62 to −0.19) with safiglipron 30 mg, 60 mg and 90 mg, respectively; the upper confidence limits were below the prespecified 0.4% margin for all three doses, establishing non-inferiority. Under the treatment policy estimand used for superiority testing, the differences were −0.31% (−0.49 to −0.13), −0.14% (−0.34 to 0.06) and −0.25% (−0.45 to −0.05) with safiglipron 30 mg, 60 mg and 90 mg, respectively (Table 2, Fig. 2a and Extended Data Table 1). Superiority was established for 90 mg (one-sided *P* = 0.0067) but not for 60 mg (*P* = 0.0789); the fixed sequence, therefore, stopped, and subsequent hypotheses were not confirmatory. Longitudinal changes from baseline in HbA1c through week 52 under the efficacy estimand are shown in Fig. 2b. In exploratory subgroup analyses, a nominal interaction was observed for baseline HbA1c stratum (≤8.5% versus >8.5%; global *P* for interaction = 0.0242), whereas results were otherwise generally consistent across prespecified subgroups (Extended Data Fig. 2).

**Table 2:** Primary and key secondary efficacy endpoints at week 32 in the intention-to-treat population

Endpoint	Safiglipron 30 mg (*N* = 202)	Safiglipron 60 mg (*N* = 203)	Safiglipron 90 mg (*N* = 203)	Dapagliflozin 10 mg (*N* = 202)
Primary endpoint: HbA1c change from baseline, primary non-inferiority estimand				
LS mean change from baseline, % (95% CI)	−1.58 (−1.73 to −1.43) *n* = 179	−1.50 (−1.66 to −1.35) *n* = 171	−1.68 (−1.84 to −1.53) *n* = 168	−1.28 (−1.43 to −1.13) *n* = 190
Difference versus dapagliflozin, percentage points (95% CI); one-sided *P* value for non-inferiority	−0.30 (−0.51 to −0.09) *P* < 0.0001	−0.22 (−0.44 to −0.01) *P* < 0.0001	−0.40 (−0.62 to −0.19) *P* < 0.0001	NA
Primary endpoint superiority, treatment policy estimand				
LS mean change from baseline, % (95% CI)	−1.58 (−1.72 to −1.45) *n* = 186	−1.42 (−1.56 to −1.27) *n* = 183	−1.53 (−1.68 to −1.37) *n* = 179	−1.27 (−1.40 to −1.15) *n* = 193
Difference versus dapagliflozin, percentage points (95% CI); one-sided *P* value for superiority	−0.31 (−0.49 to −0.13) Not formally tested	−0.14 (−0.34 to 0.06) *P* = 0.0789	−0.25 (−0.45 to −0.05) *P* = 0.0067	NA
Key secondary endpoints, efficacy estimand				
Participants achieving HbA1c <7.0%				
Estimated proportion, % (95% CI)	55.7 (47.62−63.54) *n* = 166	54.9 (46.42−63.11) *n* = 143	63.5 (55.03−71.20) *n* = 140	36.5 (29.49−44.06) *n* = 182
OR versus dapagliflozin (95% CI)	2.19 (1.39−3.45)	2.12 (1.33−3.37)	3.03 (1.89−4.87)	NA
Participants achieving HbA1c ≤6.5%				
Estimated proportion, % (95% CI)	41.4 (34.18−49.06) *n* = 166	38.9 (31.28−47.10) *n* = 143	48.6 (40.39−56.87) *n* = 140	16.1 (11.55−21.96) *n* = 182
OR versus dapagliflozin (95% CI)	3.69 (2.25−6.03)	3.32 (2.00−5.52)	4.93 (2.96−8.20)	NA
Fasting plasma glucose				
LS mean change from baseline, mmol l^−1^ (95% CI)	−2.23 (−2.48 to −1.97) *n* = 167	−2.28 (−2.55 to −2.01) *n* = 143	−2.59 (−2.86 to −2.33) *n* = 140	−2.00 (−2.24 to −1.75) *n* = 182
Difference versus dapagliflozin, mmol l^−1^ (95% CI)	−0.23 (−0.58 to 0.13)	−0.28 (−0.64 to 0.08)	−0.60 (−0.96 to −0.23)	NA
Body weight, % change from baseline				
LS mean percentage change from baseline (95% CI)	−2.35 (−2.92 to −1.78) *n* = 167	−3.55 (−4.14 to −2.95) *n* = 143	−4.17 (−4.76 to −3.58) *n* = 140	−3.60 (−4.15 to −3.05) *n* = 183
Difference versus dapagliflozin, percentage points (95% CI)	1.25 (0.46−2.04)	0.06 (−0.75 to 0.86)	−0.56 (−1.37 to 0.24)	NA

The primary non-inferiority estimand used a hypothetical strategy for permanent treatment discontinuation and initiation of additional glucose-lowering medication. The superiority analysis of the primary endpoint used the treatment policy estimand, whereas key secondary endpoints were analyzed under the efficacy estimand. The fixed sequence stopped at the 60-mg primary endpoint superiority comparison; subsequent hypotheses were not tested within the multiplicity-controlled hierarchy.

LS, least-squares; NA, not applicable.

### Secondary outcomes

**Extended Data Fig. 3:**
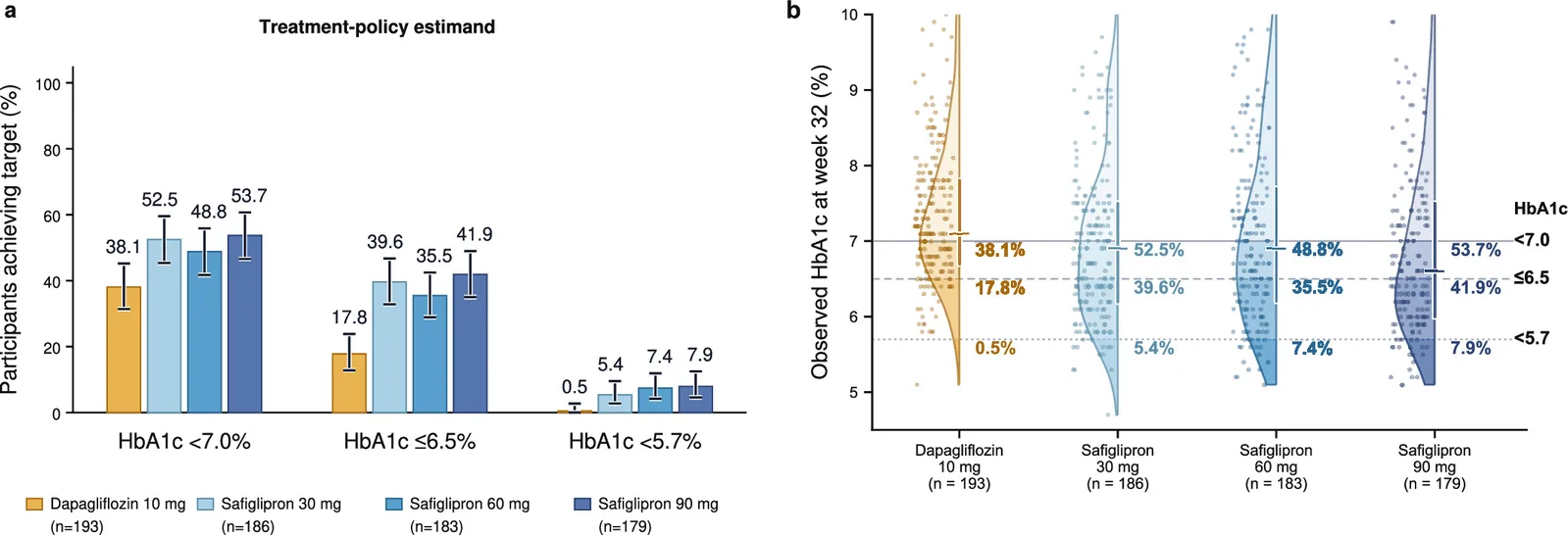
Week-32 HbA1c distributions and target attainment under the treatment-policy estimand. **a**, Model-estimated percentages of participants achieving HbA1c <7.0%, HbA1c ≤6.5% or HbA1c <5.7% at week 32. Bars and numerical labels show GEE-estimated marginal probabilities expressed as percentages, and error bars indicate two-sided 95% CIs. **b**, Observed HbA1c distributions at week 32. Half-violins and points show observed data, vertical lines indicate interquartile ranges, and short horizontal lines indicate medians. Colored percentages reproduce the corresponding model estimates in a, and horizontal lines mark the three HbA1c targets. The treatment-policy analysis used the intention-to-treat population and the prespecified missing-data procedure. Randomized group sizes (N), ordered as displayed, were 202, 202, 203 and 203. The n values shown in the panels denote participants with available observed week-32 HbA1c data (193, 186, 183 and 179, respectively) and are not denominators used to calculate the model-estimated percentages. CI=confidence interval; GEE=generalized estimating equation; HbA1c=glycated hemoglobin. Source data

At week 32, under the efficacy estimand, estimated HbA1c target attainment rates below 7.0% were 55.7%, 54.9% and 63.5% with safiglipron 30 mg, 60 mg and 90 mg, respectively, versus 36.5% with dapagliflozin; corresponding rates at or below 6.5% were 41.4%, 38.9% and 48.6% versus 16.1% (Table 2, Fig. 2c and Supplementary Fig. 2). Treatment policy estimand analyses of target attainment are shown in Extended Data Fig. 3.

Under the efficacy estimand, the least-squares mean changes from baseline in FPG were −2.23 mmol l^−1^ (95% CI: −2.48 to −1.97), −2.28 mmol l^−1^ (−2.55 to −2.01) and −2.59 mmol l^−1^ (−2.86 to −2.33) with safiglipron 30 mg, 60 mg and 90 mg, respectively, as compared to −2.00 mmol l^−1^ (−2.24 to −1.75) with dapagliflozin (Table 2).

Body weight decreased from baseline in all groups. Under the efficacy estimand, the least-squares mean percentage changes in body weight at week 32 were −2.35% (95% CI: −2.92 to −1.78), −3.55% (−4.14 to −2.95) and −4.17% (−4.76 to −3.58) with safiglipron 30 mg, 60 mg and 90 mg, respectively, as compared to −3.60% (−4.15 to −3.05) with dapagliflozin (efficacy estimand; Fig. 2f). The estimated treatment differences versus dapagliflozin were 1.25% (95% CI: 0.46−2.04), 0.06% (95% CI: −0.75 to 0.86) and −0.56% (95% CI: −1.37 to 0.24) (Table 2). Results with the treatment policy estimand were consistent (Fig. 2e).

**Fig. 3:**
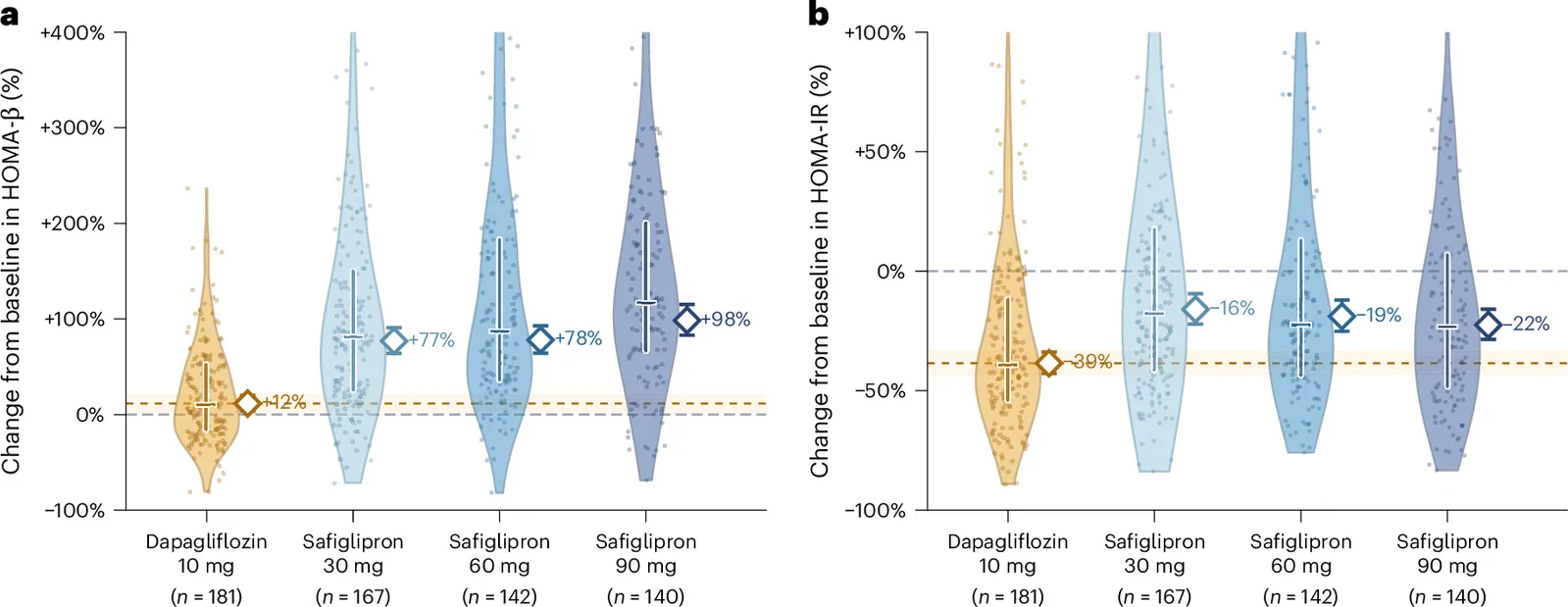
Indices of β cell function and insulin resistance at week 32. **a**, Geometric mean percentage change from baseline to week 32 in HOMA-β. **b**, Geometric mean percentage change from baseline to week 32 in HOMA-IR. Analyses were done in the intention-to-treat population under the efficacy estimand using a hypothetical strategy for intercurrent events; diamonds and error bars indicate model-estimated geometric mean percentage changes and 95% CIs. Violins and points show observed data, vertical lines indicate interquartile ranges and short horizontal lines indicate medians. The dashed line and shaded band indicate the dapagliflozin estimate and 95% CI. Exact *n* values denote participants with available observed week 32 data contributing to the analysis. Source data

HOMA-derived indices were analyzed as prespecified secondary endpoints under the efficacy estimand. Geometric mean percentage increases from baseline in HOMA-β at week 32 were 76.88%, 77.98% and 98.47% with safiglipron 30 mg, 60 mg and 90 mg, respectively, versus 11.52% with dapagliflozin. HOMA-IR decreased by 16.02%, 18.90% and 22.49%, respectively, versus 38.52% with dapagliflozin (Fig. 3 and Supplementary Table 4).

Seven-point SMBG profiles appeared to show reductions in the mean daily, pre-meal and 2-hour post-prandial values and in glucose excursions between the pre-meal and 2-hour post-prandial values across all treatment groups at week 32 (Fig. 2d). Rescue therapy was required by three (1.5%) participants, two (1.0%) participants and one (0.5%) participant in the safiglipron 30 mg, 60 mg and 90 mg groups, respectively, whereas no participant in the dapagliflozin group required rescue therapy by week 32 (Supplementary Table 4). Waist circumference decreased across all treatment groups, generally paralleling the changes in body weight (Supplementary Table 4).

At week 32, DTSQ status version scores increased from baseline by +2.0, +1.9 and +2.2 points with safiglipron 30 mg, 60 mg and 90 mg, respectively, and by +1.6 points with dapagliflozin. Perceived hyperglycemia scores decreased from baseline with safiglipron (−1.3 to −1.5 points), similar to dapagliflozin (−1.4 points), and perceived hypoglycemia scores decreased from baseline with safiglipron (−0.2 to −0.3 points), similar to dapagliflozin (−0.3 points); full results are shown in Supplementary Table 7.

Efficacy outcomes at week 52 are shown in Supplementary Table 5 and Supplementary Fig. 1. These included model-based and observed-case analyses of glycemic measures, body weight and waist circumference, HbA1c target achievement, seven-point SMBG profiles and indices of β cell function.

### Safety

**Extended Data Fig. 4:**
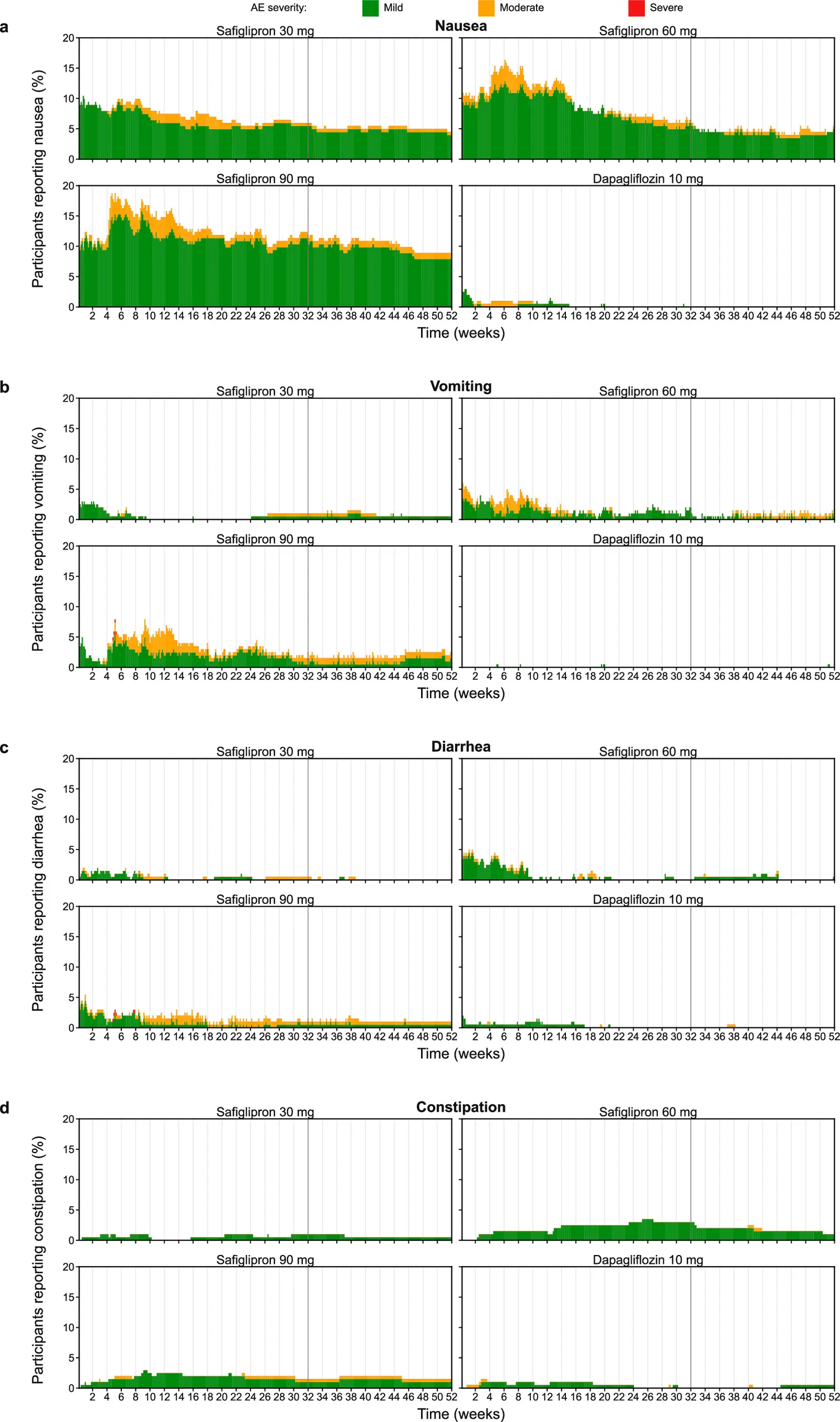
Daily prevalence and severity of gastrointestinal adverse events through week 52. **a**, Nausea. **b**, Vomiting. **c**, Diarrhea. **d**, Constipation. In each panel, each vertical bar represents one study day and shows the proportion of participants in the safety population with an ongoing treatment-emergent event of the specified preferred term on that day, with color indicating severity (green, mild; yellow, moderate; red, severe). A participant was counted once per preferred term on each study day. Percentages were calculated using the number of participants in each randomized treatment group who received at least one dose as the denominator. Events were counted irrespective of relationship to study treatment. The vertical lines denote week 32. Events are listed by Medical Dictionary for Regulatory Activities preferred term. Source data

**Extended Data Fig. 5:**
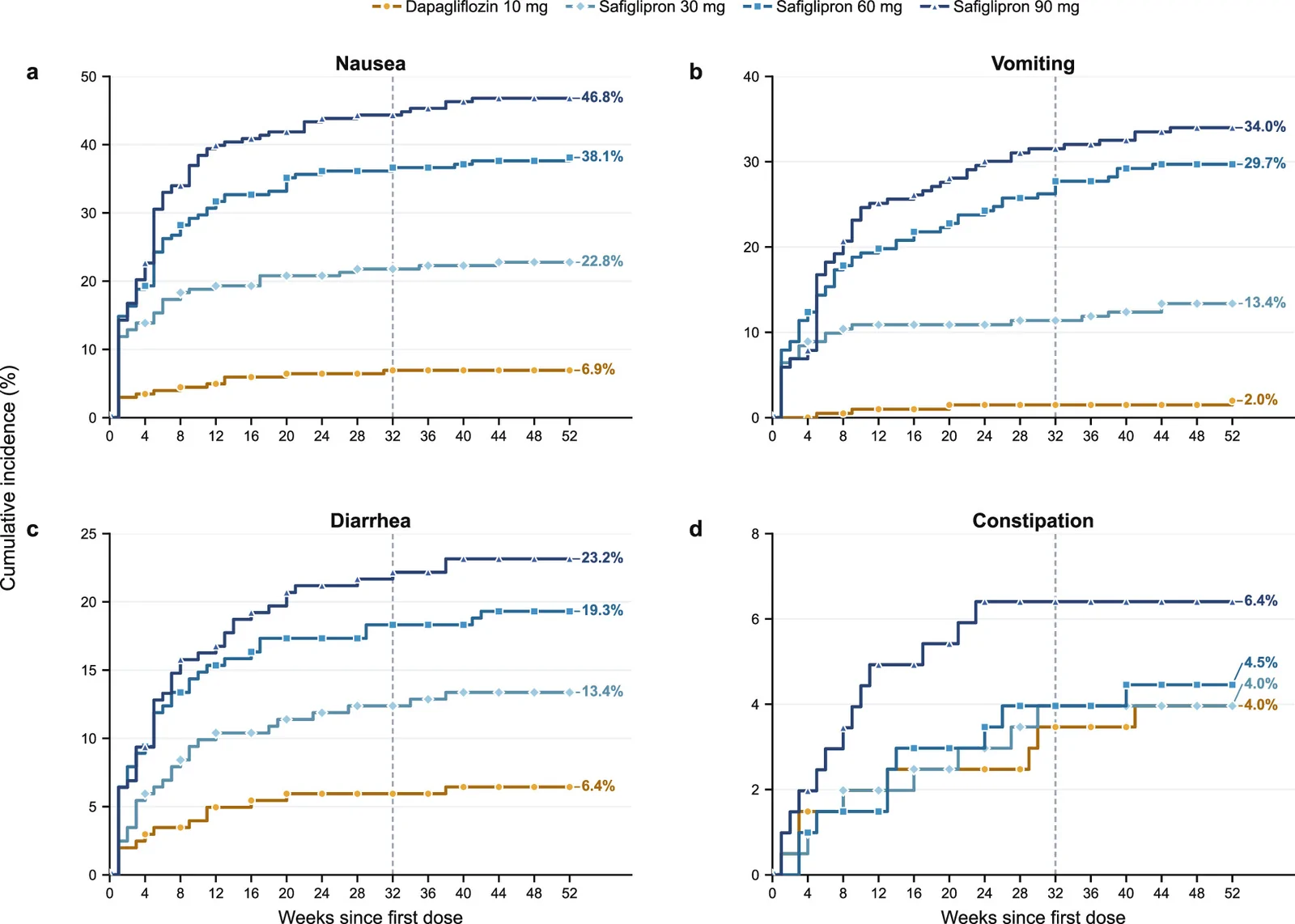
Cumulative incidence of gastrointestinal treatment-emergent adverse events through week 52. **a**, Nausea. **b**, Vomiting. **c**, Diarrhea. **d**, Constipation. Each curve shows the cumulative percentage of participants in the safety population with at least one post-dose treatment-emergent event of the specified Medical Dictionary for Regulatory Activities preferred term, according to the date of first onset. Recurrent events were counted once per participant for each preferred term. Percentages were calculated using the fixed safety-population denominator for each randomized treatment group (dapagliflozin 10 mg, n=202; safiglipron 30 mg, n=202; safiglipron 60 mg, n=202; safiglipron 90 mg, n=203). Events were included irrespective of their relationship to study treatment. The vertical dashed lines indicate week 32, and values at the right indicate cumulative incidence at week 52. These descriptive curves were not adjusted for censoring. Source data

Treatment-emergent adverse events (TEAEs) occurred in 188 (93.1%), 195 (96.5%) and 199 (98.0%) participants receiving safiglipron 30 mg, 60 mg and 90 mg, respectively, compared to 188 (93.1%) participants receiving dapagliflozin during the 52-week treatment period (Table 3). The most common TEAEs were gastrointestinal events, including nausea, vomiting and diarrhea, which were mostly mild to moderate in severity (Supplementary Table 8). First-onset gastrointestinal events accumulated predominantly during the dose-titration period and more slowly thereafter, whereas their daily prevalence decreased over time (Extended Data Figs. 4 and 5). Among selected TEAEs of clinical interest for SGLT2 inhibitor therapy, urinary tract infections were reported in 11 (5.4%), nine (4.5%) and 16 (7.9%) participants receiving safiglipron 30 mg, 60 mg and 90 mg, respectively, and in 12 (5.9%) participants receiving dapagliflozin. Other selected events occurred in no more than 1.5% of participants in any group, and no genital mycotic infection or dehydration was reported (Table 3).

**Table 3:** TEAEs during the 52-week treatment period in the safety population

Events, *n* (%)	Safiglipron 30 mg (*N* = 202)	Safiglipron 60 mg (*N* = 202)	Safiglipron 90 mg (*N* = 203)	Dapagliflozin 10 mg (*N* = 202)
Overview of adverse events				
Any TEAE	188 (93.1)	195 (96.5)	199 (98.0)	188 (93.1)
Maximum severity of TEAE				
Mild	108 (53.5)	109 (54.0)	114 (56.2)	110 (54.5)
Moderate	73 (36.1)	80 (39.6)	80 (39.4)	70 (34.7)
Severe	7 (3.5)	6 (3.0)	5 (2.5)	8 (4.0)
TEAE considered related to study treatment	149 (73.8)	175 (86.6)	174 (85.7)	112 (55.4)
Serious TEAE	21 (10.4)	12 (5.9)	10 (4.9)	18 (8.9)
Death	0	0	1 (0.5)	0
TEAE leading to permanent treatment discontinuation	8 (4.0)	8 (4.0)	8 (3.9)	3 (1.5)
Gastrointestinal TEAE leading to permanent treatment discontinuation	6 (3.0)	7 (3.5)	7 (3.4)	0
Prespecified adverse events of special interest				
Any prespecified adverse event of special interest	0	1 (0.5)	0	0
Clinically significant hepatic enzyme abnormality	0	0	0	0
Clinically significant calcitonin abnormality	0	0	0	0
Acute pancreatitis	0	1 (0.5)	0	0
Hypoglycemic events				
Level 1 hypoglycemia	19 (9.4)	13 (6.4)	18 (8.9)	5 (2.5)
Level 2 hypoglycemia	3 (1.5)	3 (1.5)	4 (2.0)	0
Level 3 hypoglycemia (severe hypoglycemia)	0	0	0	0
TEAEs reported in ≥10% of participants in any group				
Nausea	46 (22.8)	77 (38.1)	95 (46.8)	14 (6.9)
Vomiting	27 (13.4)	60 (29.7)	69 (34.0)	4 (2.0)
Diarrhea	27 (13.4)	39 (19.3)	47 (23.2)	13 (6.4)
Abdominal distension	16 (7.9)	28 (13.9)	25 (12.3)	7 (3.5)
Lipase increased	37 (18.3)	36 (17.8)	32 (15.8)	9 (4.5)
ALT increased	17 (8.4)	24 (11.9)	17 (8.4)	18 (8.9)
Ketone body urine present	4 (2.0)	5 (2.5)	7 (3.4)	21 (10.4)
Decreased appetite	43 (21.3)	50 (24.8)	63 (31.0)	18 (8.9)
Hyperuricemia	24 (11.9)	35 (17.3)	35 (17.2)	23 (11.4)
Hyperlipidemia	19 (9.4)	15 (7.4)	21 (10.3)	26 (12.9)
Upper respiratory tract infection	30 (14.9)	24 (11.9)	26 (12.8)	22 (10.9)
Diabetic retinopathy	19 (9.4)	22 (10.9)	17 (8.4)	26 (12.9)
Selected TEAEs of clinical interest for SGLT2 inhibitor				
Urinary tract infection	11 (5.4)	9 (4.5)	16 (7.9)	12 (5.9)
Polyuria or pollakiuria	0	1 (0.5)	1 (0.5)	0
Vaginal infection	0	0	3 (1.5)	3 (1.5)
Balanoposthitis	0	0	0	1 (0.5)
Genital mycotic infection	0	0	0	0
Dehydration	0	0	0	0
Hypotension	0	1 (0.5)	1 (0.5)	0

Data are *n* (%). The safety population included all randomized participants who received at least one dose of study treatment; participants were analyzed according to randomized treatment group. Percentages were calculated using the number of participants in each group as the denominator. TEAEs were adverse events that occurred or worsened after the first dose of study treatment during the total 52-week treatment period. Maximum severity refers to the highest severity reported for each participant. Treatment-related TEAEs were events considered by the investigator to be related to study treatment. Participants could have more than one event. TEAEs reported in ≥10% of participants in any treatment group are listed by Medical Dictionary for Regulatory Activities preferred term. Level 1 hypoglycemia was defined as a measured glucose concentration of <3.9 mmol l^−1^ and ≥3.0 mmol l^−1^; level 2 hypoglycemia was defined as <3.0 mmol l^−1^; and level 3 hypoglycemia was defined as a severe event requiring assistance, irrespective of glucose concentration. Only post-dose hypoglycemic events were included.

Serious adverse events occurred in 21 (10.4%), 12 (5.9%) and 10 (4.9%) participants receiving safiglipron 30 mg, 60 mg and 90 mg, respectively, compared to 18 (8.9%) participants receiving dapagliflozin. Serious adverse events by preferred term are shown in Supplementary Table 10. TEAEs leading to permanent treatment discontinuation occurred in eight (4.0%), eight (4.0%) and eight (3.9%) participants for the safiglipron 30 mg, 60 mg and 90 mg groups, respectively, and in three (1.5%) participants in the dapagliflozin group; most were attributable to gastrointestinal adverse events in the safiglipron groups (3.0%, 3.5% and 3.4%, respectively). Detailed discontinuation events are shown in Extended Data Table 2 and Supplementary Table 11. Post hoc site-adjusted analyses did not materially alter the comparisons for treatment-related TEAEs or TEAEs leading to permanent treatment discontinuation (Supplementary Table 15).

One participant in the safiglipron 90 mg group died of acute myocardial infarction, which was considered by the investigator to be unrelated to the study treatment. Level 1 hypoglycemia occurred in 19 (9.4%), 13 (6.4%) and 18 (8.9%) participants receiving safiglipron 30 mg, 60 mg and 90 mg, respectively, and in five (2.5%) participants receiving dapagliflozin. Corresponding numbers for level 2 hypoglycemia were three (1.5%), three (1.5%), four (2.0%) and zero. No episodes of severe hypoglycemia were reported.

By week 52, hepatic transaminase levels had generally decreased from baseline across the safiglipron groups (Supplementary Table 12), and no cases met Hyʼs law laboratory criteria (Extended Data Table 3). Changes in diabetic retinopathy stage, vital signs and scheduled electrocardiographic findings through week 52 are summarized in Supplementary Tables 9, 13 and 14.

### Exploratory outcomes

#### Cardiometabolic and renal risk markers

**Extended Data Fig. 6:**
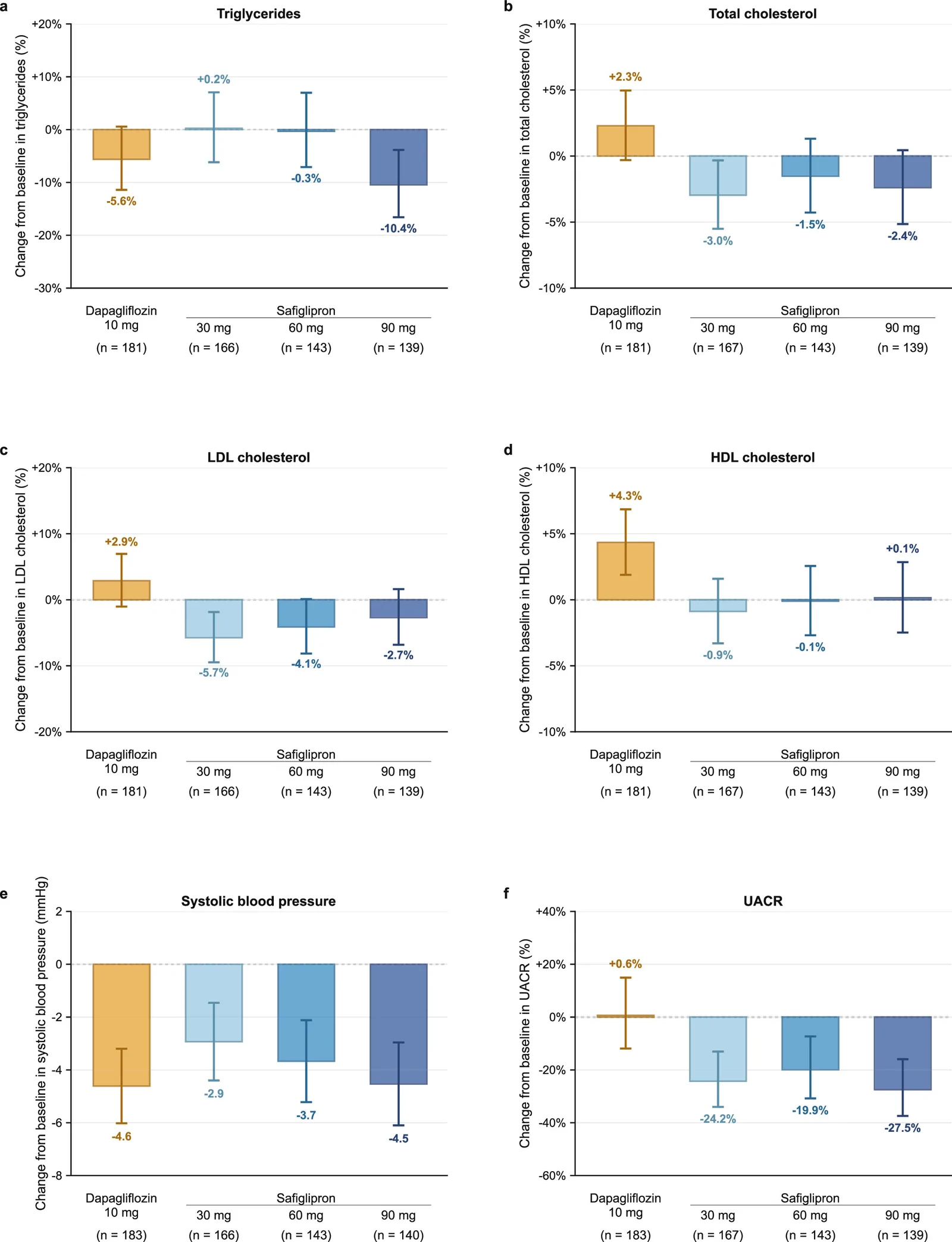
Cardiometabolic and renal outcomes at week 32. **a**, Geometric mean percentage change from baseline in triglycerides. **b**, Geometric mean percentage change from baseline in total cholesterol. **c**, Geometric mean percentage change from baseline in LDL cholesterol. **d**, Geometric mean percentage change from baseline in HDL cholesterol. **e**, Least-squares mean change from baseline in systolic blood pressure. **f**, Geometric mean percentage change from baseline in UACR. Analyses were done in the intention-to-treat population under the efficacy estimand using a hypothetical strategy for intercurrent events. Bars and numerical labels show model estimates, and error bars indicate 95% CIs. The horizontal dashed line indicates no change from baseline. Exact n values denote participants with available observed week-32 data. CI=confidence interval; HDL=high-density lipoprotein; LDL=low-density lipoprotein; UACR=urinary albumin-to-creatinine ratio. Source data

Under the efficacy estimand, prespecified exploratory analyses assessed changes in blood pressure, lipid parameters and UACR at week 32. Safiglipron 90 mg was associated with reductions from baseline in triglycerides (−10.4%), low-density lipoprotein (LDL) cholesterol (−2.7%) and total cholesterol (−2.4%), whereas the effects at lower doses were less consistent (Extended Data Fig. 6). By comparison, dapagliflozin was associated with a reduction in triglycerides (−5.6%) but increases in LDL cholesterol (+2.9%) and total cholesterol (+2.3%). Safiglipron also reduced systolic blood pressure, showing −2.93 mmHg (95% CI: −4.40 to −1.46), −3.67 mmHg (−5.23 to −2.11) and −4.53 mmHg (−6.10 to −2.97) for the 30-mg, 60-mg and 90-mg groups, respectively, compared to −4.61 mmHg (−6.03 to −3.20) for dapagliflozin. All three doses reduced the UACR, with dapagliflozin-adjusted percentage differences of −24.7% (95% CI: −37.8 to −8.9), −20.4% (−34.7 to −3.0) and −28.0% (−40.9 to −12.1) for the 30-mg, 60-mg and 90-mg groups, respectively (Extended Data Fig. 6 and Supplementary Table 6).

### Sensitivity analyses

The prespecified sensitivity analysis using multiple imputation followed by analysis of covariance yielded results consistent with the primary analysis. The prespecified two-dimensional tipping-point analysis identified Pareto-minimal delta pairs at which non-inferiority first failed (Supplementary Table 3).

## Discussion

OUTSTAND-2 is, to our knowledge, the first double-blind phase 3 trial comparing an oral small-molecule GLP-1 receptor agonist with an SGLT2 inhibitor. All three doses of once-daily oral safiglipron were non-inferior to dapagliflozin for HbA1c reduction at week 32 under the primary non-inferiority estimand. This estimand assessed HbA1c-lowering efficacy in a hypothetical scenario in which participants remained on assigned treatment without prolonged use of additional glucose-lowering medication, thereby enabling a direct comparison of the pharmacological efficacy of the two agents. Under the treatment policy estimand, the 90-mg dose met the prespecified statistical criterion for superiority within the multiplicity-controlled fixed sequence. The sequence stopped at the 60-mg superiority comparison; subsequent key secondary findings should, therefore, be interpreted as prespecified supportive estimates rather than multiplicity-controlled confirmatory results. By reflecting the consequences of both efficacy and tolerability, the treatment policy analysis provided a broader, practice-oriented perspective on the population average treatment effect that may inform treatment selection. This perspective was clinically relevant given the higher rates of gastrointestinal adverse events and adverse-event-related discontinuation with safiglipron than with dapagliflozin. Other glycemic outcomes, including FPG, HbA1c target attainment and seven-point SMBG profiles, were broadly similar between safiglipron and dapagliflozin. Together, these findings demonstrate the glucose-lowering efficacy of safiglipron across the three doses evaluated.

Previous head-to-head trials provide useful context for OUTSTAND-2, although differences in agents, doses, populations, study designs and estimands should be considered when interpreting results across studies. In the open-label PIONEER-2 trial, oral semaglutide 14 mg, a peptide GLP-1 receptor agonist, produced a greater reduction in HbA1c than empagliflozin at week 26 (ref. ^17^). In the open-label ACHIEVE-2 trial, the oral small-molecule GLP-1 receptor agonist orforglipron at doses of 3 mg, 12 mg and 36 mg was superior to dapagliflozin for HbA1c reduction at week 40 (ref. ^9^). In OUTSTAND-2, safiglipron 90 mg demonstrated statistical superiority over dapagliflozin under the treatment policy estimand. Taken together, these studies show that oral GLP-1 receptor agonists can provide glycemic efficacy at least similar to that of SGLT2 inhibitors, with statistical superiority demonstrated for selected agents and doses, and OUTSTAND-2 complements the previous open-label evidence by evaluating multiple safiglipron doses under double-blind conditions.

In supportive analyses, body weight reduction was smaller with safiglipron 30 mg than with dapagliflozin, whereas the differences for the 60-mg and 90-mg doses were not statistically significant. By comparison, in ACHIEVE-2, orforglipron 12 mg and 36 mg produced greater body weight reductions than dapagliflozin at week 40 (ref. ^9^). In PIONEER-2, superiority of oral semaglutide over empagliflozin for body weight was not demonstrated at the confirmatory week 26 assessment^17^. These differing body weight outcomes should be interpreted in the context of the agents, dose regimens and treatment objectives of each trial. The 30−90-mg dose range in OUTSTAND-2 was selected on the basis of phase 2 findings to characterize glycemic efficacy in type 2 diabetes rather than to maximize weight reduction^14^. Higher doses of safiglipron (120 mg and 180 mg) have been evaluated separately for weight management in a phase 3 trial, HARBOR-1 (NCT06904105). The more limited body weight effect observed with the 30-mg dose may be particularly relevant for individuals for whom additional weight loss is not a treatment priority, including some individuals in East Asian populations, where type 2 diabetes often occurs at lower BMI, and β cell dysfunction contributes prominently to its pathophysiology^18–21^. For such individuals, effective glycemic control without marked additional weight loss might be desirable, although this potential application requires prospective evaluation.

In prespecified supportive analyses, HOMA-derived indices showed distinct patterns: the profile with safiglipron was characterized by increases in HOMA-β, whereas that with dapagliflozin was characterized by reductions in HOMA-IR. In prespecified exploratory analyses, safiglipron and dapagliflozin were both associated with reductions in systolic blood pressure, whereas changes in UACR and lipid parameters varied across treatments and safiglipron doses. Previous studies have shown that GLP-1 receptor agonists enhance glucose-dependent insulin secretion, suppress appetite and may exert renoprotective effects partly through anti-inflammatory pathways, whereas SGLT2 inhibitors promote glycosuria-mediated caloric loss, natriuresis and restoration of tubuloglomerular feedback^4,5,22^. Accordingly, the two drug classes need not be viewed as mutually exclusive therapeutic options. Their complementary mechanisms across metabolic and cardiorenal pathways provide a rationale for evaluating combined GLP-1 receptor agonism and SGLT2 inhibition.

Preliminary clinical evidence supports further evaluation of this strategy. A phase 2 trial demonstrated incremental glycemic benefit when safiglipron was added to metformin and an SGLT2 inhibitor^23^. In the SOLID-DKD phase 2 trial, safiglipron reduced albuminuria and improved glycemic and body weight outcomes in participants with diabetic kidney disease and severely increased albuminuria, approximately 65% of whom were receiving stable background therapy with an SGLT2 inhibitor^16^. More broadly, modeling analyses have projected potential lifetime cardiovascular, kidney and mortality benefits from combining complementary cardiorenal therapies in individuals with type 2 diabetes and albuminuria^24^. Beyond this biological and clinical rationale, the small-molecule properties of safiglipron may offer a practical advantage for combination development. Unlike currently available oral peptide GLP-1 receptor agonist therapy, safiglipron does not require an absorption enhancer such as sodium *N*-[8-(2-hydroxybenzoyl)amino] caprylate (SNAC) or fasting administration. This dosing profile may facilitate the development of a once-daily oral fixed-dose combination with an SGLT2 inhibitor. Larger and longer-term trials are needed to determine whether such a combination provides incremental metabolic and cardiorenal benefits beyond either treatment alone.

The safety profile of safiglipron was broadly consistent with that reported for other oral small-molecule GLP-1 receptor agonists^9–11,25^. However, gastrointestinal adverse events and TEAEs leading to permanent treatment discontinuation were more frequent with safiglipron than with dapagliflozin, although most gastrointestinal events were mild or moderate. The early peak in gastrointestinal adverse events suggests that alternative starting doses or more gradual titration schedules may warrant evaluation to improve early tolerability. No severe hypoglycemia was observed in any treatment group. By week 52, hepatic transaminase levels had generally decreased from baseline across the safiglipron groups, and no cases met Hyʼs law laboratory criteria. Although no hepatic safety signal was identified during the 52-week treatment period, larger and longer-term studies are required to characterize uncommon or delayed hepatic and other adverse effects.

This study has several limitations. First, the study enrolled only Chinese participants, which may limit the generalizability of the findings to other ethnic populations. Second, although the 52-week treatment period provided evidence for sustained glycemic control efficacy and safety, the long-term cardiovascular or kidney outcomes remain undetermined, which are being investigated in RENEW-CKD, an ongoing cardiorenal outcomes trial (NCT07625774). Third, continuous glucose monitoring was not incorporated; seven-point SMBG provided only intermittent measurements and did not permit assessment of time in range. The studyʼs strengths include its double-blind, double-dummy, active-comparator-controlled design and relatively large sample size, which enabled a rigorous comparison of an oral small-molecule GLP-1 receptor agonist with an SGLT2 inhibitor, a comparison that, to our knowledge, has previously been evaluated only in open-label trials.

In conclusion, in adults with type 2 diabetes inadequately controlled with metformin, all three doses of safiglipron were non-inferior to dapagliflozin for HbA1c reduction at week 32, and the 90-mg dose met the prespecified statistical criterion for superiority under the treatment policy estimand. Supportive and exploratory analyses also suggested favorable trends from baseline in indices of β cell function and several cardiometabolic and renal risk markers. Safety findings were broadly consistent with those reported for other oral small-molecule GLP-1 receptor agonists. Together, these findings support safiglipron as an effective oral treatment option for adults with type 2 diabetes inadequately controlled with metformin.

## Methods

### Study design and participants

The OUTSTAND-2 trial complied with all relevant ethical regulations and was conducted in accordance with the Declaration of Helsinki and Good Clinical Practice guidelines; its protocol, amendments and informed consent materials were approved by the Ethics Committee of Beijing Hospital (the coordinating center) and by the institutional review board or independent ethics committee of each of the 98 participating sites named in Supplementary Table 1.

The OUTSTAND-2 trial was a multicenter, randomized, double-blind, double-dummy, active-comparator-controlled, non-inferiority phase 3 trial conducted at 98 sites in China. The original protocol (v.1.0, 4 June 2024) was amended three times before database lock. Version 1.1 (17 July 2024) clarified study treatment discontinuation after persistent hyperglycemia despite rescue treatment and refined electrocardiographic, vital sign and laboratory procedures. Version 1.2 (26 September 2024) strengthened retinopathy eligibility and assessment, added metformin pharmacokinetic sampling, designated basal insulin as the preferred rescue treatment, added lactic acidosis as an adverse event of special interest and corrected operational details. Version 1.3 (24 March 2025) added dapagliflozin pharmacokinetic assessments and refined fasting visit, metformin dose and laboratory procedures. The complete amendment history is provided in the submitted protocol. The statistical analysis plan was finalized as v.1.0 on 26 May 2026. The trial was prospectively registered at ClinicalTrials.gov (NCT06589765).

Potential participants were recruited by investigators at the 98 participating sites and assessed against protocol-defined eligibility criteria. Screening and randomization occurred between 27 September 2024 and 30 April 2025, and the last participant completed trial follow-up on 21 May 2026. All participants personally provided written informed consent. Participants received transportation reimbursement and compensation for blood collection, as specified in the informed consent materials approved by the ethics committees. Participants were enrolled regardless of sex. Sex was recorded from the national identification document used for trial registration; gender was not assessed separately. Sex was a prespecified subgroup variable for the primary endpoint, although the trial was not powered for sex-specific treatment-effect comparisons. Sex-disaggregated participant numbers are reported in Table 1.

### Inclusion criteria

Participants must meet all of the following criteria to be enrolled in this trial:

1. Male or female, aged 18−75 years (inclusive) on the day of signing the informed consent form (ICF)
2. Type 2 diabetes mellitus diagnosed according to the Chinese Guidelines for the Prevention and Treatment of Type 2 Diabetes (2020 edition), with a disease duration of at least 3 months at screening
3. HbA1c of 7.5−11.0% (inclusive) at screening, as measured by a local laboratory
4. Stable treatment for type 2 diabetes for at least 8 weeks before screening, with no planned change during the trial. In addition to regular diet and exercise, treatment had to consist of metformin monotherapy at a stable daily dose of at least 1,500 mg or a maximum tolerated daily dose of at least 1,000 mg, consistent with the product label.
5. BMI of 19.0−40.0 kg m^−^^2^ (inclusive) at screening
6. Voluntary written informed consent before trial initiation, full understanding of the study procedures and potential adverse reactions and ability and willingness to complete the study in accordance with the protocol, including self-monitoring of blood glucose and completion of participant diaries
7. No plans for pregnancy from signing the ICF until 2 weeks after the last dose, with willingness to use highly effective contraception as specified in the protocol

### Exclusion criteria

Participants who meet any of the following criteria shall not be enrolled in this trial:

Those with the following abnormalities at screening or randomization:

1. Presence of the following laboratory test abnormalities at screening or randomization:
  Fasting venous glucose >15 mmol l^−1^
  Alanine aminotransferase (ALT) or aspartate aminotransferase (AST) >2× upper limit of normal (ULN)
  Total bilirubin (TBIL) >1.5× ULN
  Amylase and/or lipase >3× ULN
  Fasting triglycerides >11.3 mmol l^−1^
  Calcitonin ≥50 ng l^−1^
  Estimated glomerular filtration rate (eGFR) <45 ml min^−1^ 1.73 m^−2^ (calculated using the Chronic Kidney Disease Epidemiology Collaboration (CKD-EPI) equation)
  UACR ≥1,000 mg g^−1^
  Positive hepatitis B surface antigen (HBsAg) and HBV-DNA >ULN
2. Clinically significant electrocardiographic abnormalities at screening or randomization that could affect participant safety, including, but not limited to, supraventricular tachycardia, atrial fibrillation, atrial flutter or second-degree or third-degree atrioventricular block
3. Heart rate >100 beats per minute or Fridericia-corrected QT interval >480 ms at screening or randomization. If either value was abnormal, two additional electrocardiograms could be performed and the mean of the three measurements used.
4. Severe uncontrolled hypertension, defined as systolic blood pressure ≥160 mmHg or diastolic blood pressure ≥100 mmHg, at screening or randomization
5. Diagnosed or suspected type 1 diabetes mellitus, a specific type of diabetes mellitus or secondary diabetes mellitus
6. Acute diabetic complications, including diabetic ketoacidosis, lactic acidosis or hyperglycemic hyperosmolar state, within 6 months before screening
7. Severe or recurrent hypoglycemia within 6 months before screening, including at least three hypoglycemic episodes within 1 week or other recurrent episodes as determined by the investigator
8. Proliferative retinopathy or maculopathy or painful diabetic neuropathy, diabetic foot ulcer or intermittent claudication requiring acute treatment at screening
9. A clinically significant neurological, psychiatric, cardiovascular, endocrine, gastrointestinal, respiratory, urinary, hematological, immune or other disorder within 6 months before screening that, in the investigatorʼs judgment, could interfere with interpretation of the study or increase treatment risk
10. Personal or family history of medullary thyroid carcinoma or multiple endocrine neoplasia type 2
11. Previous or current acute or chronic pancreatitis, pancreatic injury, acute cholecystitis or symptomatic gallbladder disease requiring treatment, except after cholecystectomy when the investigator considered enrollment appropriate
12. Previous significant gastrointestinal disease, including gastric outlet obstruction, inflammatory bowel disease or active ulcer; previous gastrointestinal surgery other than gastrointestinal polypectomy or appendectomy; or long-term use of drugs that directly affect gastrointestinal motility
13. Previous or current hyperthyroidism or another thyroid disorder that was not stably controlled, except for participants receiving a stable thyroid hormone replacement regimen for at least 8 weeks with no planned change during the trial
14. Severe cardiovascular or cerebrovascular disease within 6 months before screening, including, but not limited to, New York Heart Association class III or IV heart failure, unstable angina, stroke, myocardial infarction, severe arrhythmia or coronary revascularization or planned coronary, carotid or peripheral revascularization at screening
15. Known or suspected depression, bipolar disorder, suicidal tendency, schizophrenia or another serious psychiatric disorder; cognitive impairment or a language barrier preventing full understanding of the protocol; or unwillingness to cooperate with study site staff
16. Malignant neoplasm within 5 years before screening, except adequately treated localized cancers such as basal cell skin carcinoma, cervical carcinoma in situ or prostate carcinoma in situ
17. Urinary tract or genital infection at screening or randomization or a history of urinary tract or genital infection caused by use of an SGLT2 inhibitor
18. Use of a GLP-1 receptor agonist, including a multi-target GLP-1 receptor agonist, within 3 months before screening
19. Systemic glucocorticoid therapy within 3 months before screening, except cumulative use for <14 days or topical, inhaled, intraocular or intranasal administration
20. Use of a tricyclic antidepressant, atypical antipsychotic or mood stabilizer within 3 months before screening
21. Weight loss therapy within 3 months before screening, including intensive dietary or exercise therapy, pharmacotherapy, surgery, dietary supplements or another special therapy, except liposuction performed more than 1 year earlier
22. Self-reported body weight change exceeding ±5% within the preceding 3 months at screening or randomization or a plan for intensive diet or exercise therapy for weight loss during the trial, other than diet and exercise measures for diabetes
23. Participation in a clinical trial of a drug or medical device within 3 months before screening or within five half-lives of the investigational product, whichever was longer, defined as provision of informed consent and receipt of an investigational product, placebo or investigational device
24. Known or suspected hypersensitivity to a study drug or any of its components
25. Known or suspected drug or alcohol misuse that, in the investigatorʼs judgment, made participation unsuitable
26. Blood donation or blood loss of at least 400 ml within 3 months before screening or receipt of a blood transfusion
27. Pregnancy, breastfeeding or a plan to become pregnant during the trial
28. Intolerance to venipuncture or needle phobia
29. Any other condition that, in the investigatorʼs judgment, made the participant unsuitable for the trial

### Randomization and masking

Participants were assigned 1:1:1:1 by a central interactive web response system using stratified block randomization according to screening HbA1c (≤8.5% or >8.5%) measured by the central laboratory. The allocation schedule was generated with SAS software. Participants, investigators, site staff, sponsor clinical researchers and clinical research associates were masked to treatment assignment; statistical analysts remained masked until database lock. A third-party laboratory team was unmasked as operationally required. Matching tablets supported the double-dummy design.

### Procedures

The study consisted of a 3-week single-blind placebo run-in period, a 52-week double-blind treatment period (comprising a 32-week core treatment period and a 20-week extension period) and a 2-week safety follow-up period (Extended Data Fig. 1). The 20-week extension was designed to assess the persistence of efficacy and to characterize longer-term safety and tolerability through week 52; efficacy analyses at week 52 were prespecified as supportive. Scheduled study visits occurred at baseline and at weeks 2, 4, 8, 12, 16, 20, 26, 32, 38, 44 and 52.

After the run-in period, participants were assigned to safiglipron or dapagliflozin. Both safiglipron and dapagliflozin were administered orally with no restrictions regarding food intake or dosing time. Participants assigned to the safiglipron groups began with 30 mg once daily for the first 4 weeks. Those assigned to the 60-mg or 90-mg target groups increased to 60 mg from week 5 onward; participants in the 90-mg group underwent a second escalation to 90 mg once daily beginning at week 9. Individualized dose adjustments were permitted to manage tolerability. To preserve blinding, participants received three tablet format components: a safiglipron 30-mg component, a safiglipron 60-mg component and a dapagliflozin 10-mg component, with each supplied as active treatment or matching placebo according to randomized treatment group. At the 90-mg target dose, both the 30-mg and 60-mg safiglipron components were active.

All participants continued stable background therapy with metformin throughout the trial. Participants remained on their assigned study treatment after initiation of rescue therapy. No glucose-lowering medications other than the assigned study drug, metformin and protocol-permitted rescue therapy were allowed during the trial.

For gastrointestinal intolerance, protocol-defined temporary interruption, one-step dose reduction and subsequent re-escalation were permitted. Adherence was calculated as tablets taken divided by tablets expected, multiplied by 100%; 80−120% was the protocol target. Protocol-defined FPG thresholds, confirmed by a local laboratory, triggered rescue treatment, for which basal insulin was recommended while randomized treatment was continued when possible.

At each visit, vital signs were measured; blood and urine samples were collected; and adverse events, concomitant medications and treatment adherence were recorded. A standardized MMTT was done at baseline, at week 32 and at week 52. After an overnight fast, participants consumed a standardized mixed meal within 10 minutes, and venous blood samples were collected at 0 minutes (fasting) and at 120 minutes for measurement of plasma glucose, serum insulin and serum C-peptide. The detailed schedule of trial assessments is provided in the protocol.

### Outcomes

The primary endpoint was the change in HbA1c from baseline to week 32. Key secondary endpoints, all assessed at week 32, were the proportions of participants with an HbA1c of less than 7.0% and of 6.5% or less, the change from baseline in FPG and the percent change from baseline in body weight. Supportive secondary endpoints included changes from baseline at weeks 32 and 52 in body weight, waist circumference, fasting serum insulin and C-peptide concentrations, HOMA-β, HOMA-IR, 2-hour post-prandial plasma glucose, serum insulin and serum C-peptide and seven-point SMBG profiles, as well as the proportion of participants with an HbA1c of less than 5.7% and the proportion of participants receiving rescue therapy. Additional endpoints included the change from baseline at week 52 in HbA1c, FPG, percent change from baseline in body weight and the proportions of participants with an HbA1c of less than 7.0% and of 6.5% or less. Patient-reported outcomes included the change from baseline in the DTSQ status score at weeks 32 and 52. Exploratory endpoints included changes from baseline in blood pressure and percent changes from baseline in lipid parameters and the UACR at weeks 32 and 52.

Safety was assessed throughout the trial by monitoring adverse events, hypoglycemic episodes, laboratory tests, vital signs, 12-lead electrocardiographic findings and retinal examinations with fundus photography at weeks 32 and 52. Prespecified adverse events of special interest included clinically significant hepatic enzyme abnormalities, clinically significant calcitonin abnormalities, acute pancreatitis, severe hypoglycemia and lactic acidosis.

### Statistical analysis

A total of 800 participants (200 per group) was planned. An intercurrent event incidence of 15% was assumed in sample size planning. With a common standard deviation of 1.1 percentage points and no true between-group difference, this provided approximately 87% overall power to establish non-inferiority for all three safiglipron doses at a one-sided *α* of 0.025. The 0.4-percentage-point margin was prespecified in the protocol as a commonly used margin for HbA1c non-inferiority trials.

The intention-to-treat population included all randomized participants analyzed according to assigned group. The safety population included all randomized participants who received at least one dose of study treatment and was analyzed according to assigned group.

Three prespecified estimand frameworks addressed complementary clinical questions under ICH E9(R1)^26^. For the primary non-inferiority estimand, permanent discontinuation of randomized treatment and initiation of additional glucose-lowering medication for more than 14 cumulative days were handled using a hypothetical strategy. This estimand assessed the comparative efficacy of the randomized treatments themselves under continued treatment. The treatment policy estimand included outcomes irrespective of these events and was used for superiority testing within the fixed sequence. This estimand assessed the overall treatment effect from a broader clinical perspective, reflecting the consequences of both efficacy and tolerability. The efficacy estimand used a hypothetical strategy for these events and additionally treated insufficient study drug exposure during the 90 days before the endpoint as an intercurrent event. This supportive estimand assessed efficacy under idealized conditions of continued treatment and adequate study drug exposure.

The primary endpoint was analyzed by restricted maximum-likelihood mixed modeling for repeated measures (MMRM) with baseline HbA1c as a covariate and treatment, visit and treatment-by-visit interaction as fixed effects. An unstructured covariance matrix was prespecified, with compound symmetry if the model did not converge. Under the primary non-inferiority estimand, post-intercurrent-event observations were set to missing, and remaining missing data were handled under a missing-at-random assumption. Sensitivity analysis 1 used multiple imputation followed by analysis of covariance, and sensitivity analysis 2 used delta-adjusted multiple imputation in a tipping-point analysis, with Rubinʼs rules used across imputed datasets.

Prespecified subgroup analyses of the primary endpoint were conducted under the primary non-inferiority estimand using the same MMRM framework as the primary analysis. Subgroup, treatment-by-subgroup, visit-by-subgroup and treatment-by-visit-by-subgroup terms were added as fixed effects, and the treatment-by-visit interaction was retained. The randomization stratum (baseline HbA1c ≤8.5% versus >8.5%) was also included as a fixed effect, except when baseline HbA1c itself defined the subgroup. The same within-participant covariance structure as in the primary analysis was used. For each subgroup variable, the global treatment-by-subgroup interaction *P* value was obtained from a two-sided type III joint *F*-test of the null hypothesis that the week 32 treatment effects were homogeneous across subgroup levels.

For the treatment policy estimand, Markov chain Monte Carlo methods first created monotone missingness (30 imputations; prespecified seed 7535302). Retrieved-dropout imputation was used within treatment group and intercurrent event category when retrieved observations were available for at least 30% of participants with that event and the model was estimable; otherwise, return-to-baseline imputation was used. Binary endpoints were analyzed using generalized estimating equations (GEE) and continuous endpoints using MMRM. Selected insulin, C-peptide, HOMA, lipid and UACR endpoints were log transformed and back transformed. DTSQ outcomes were summarized descriptively without imputation.

A prespecified fixed sequence controlled the family-wise type I error at a one-sided *α* of 0.025. Non-inferiority for HbA1c change was tested for 90 mg, 60 mg and 30 mg, followed by superiority for HbA1c change in the same dose order and then HbA1c below 7.0%, HbA1c at or below 6.5%, FPG and percentage change in body weight, each in descending dose order. Testing stopped at the first one-sided *P* value of 0.025 or greater. In the observed results, testing stopped at superiority of safiglipron 60 mg versus dapagliflozin for HbA1c change (*P* = 0.0789). Subsequent analyses were not confirmatory. Subgroup analyses and interaction tests were prespecified exploratory analyses without multiplicity adjustment. Prespecified safety analyses were descriptive; missing safety data were not imputed. Post hoc exploratory analyses of treatment-related TEAEs and adverse events leading to permanent treatment discontinuation used logistic regression models with treatment group as a fixed effect, with and without a random intercept for study center (Supplementary Table 15). Analyses used SAS v.9.4 or later.

### Reporting summary

Further information on research design is available in the Nature Portfolio Reporting Summary linked to this article.

## Online content

Any methods, additional references, Nature Portfolio reporting summaries, source data, extended data, supplementary information, acknowledgements, peer review information; details of author contributions and competing interests; and statements of data and code availability are available at https://doi.org/10.1038/s41591-026-04713-y.

## Supplementary information

Supplementary Information Methods, Figs. 1 and 2 with legends, Tables 1−15 with legends, references, protocol and statistical analysis plan.

Reporting Summary

## Source data

Source Data Fig. 2 Statistical source data for **a**−**f**: HbA1c, body weight and seven-point SMBG outcomes, with the analysis population or estimand, estimates, uncertainty and observed sample sizes as applicable.

Source Data Fig. 3 Statistical source data for **a** and **b**: HOMA-β and HOMA-IR at week 32.

Source Data Extended Data Fig. 2 Statistical source data for the prespecified subgroup analyses of the primary endpoint.

Source Data Extended Data Fig. 3 Statistical source data for **a** and **b**: week 32 HbA1c target attainment and observed distributions under the treatment policy estimand.

Source Data Extended Data Fig. 4 Statistical source data for **a**−**d**: daily prevalence and severity of nausea, vomiting, diarrhea and constipation through week 52.

Source Data Extended Data Fig. 5 Statistical source data for **a**−**d**: cumulative incidence of nausea, vomiting, diarrhea and constipation through week 52.

Source Data Extended Data Fig. 6 Statistical source data for **a**−**f**: cardiometabolic and renal risk markers at week 32.

## Data Availability

Qualified researchers may request access to anonymized individual participant data that underlie the results reported in this article. Data will be made available beginning 24 months after primary publication of the trial results. Access will be provided to researchers who provide a methodologically sound proposal for a specific purpose. Proposals should be directed to the corresponding authors: L.G. (glx1218@163.com) or Z.Y. (zi.ye@hengrui.com). Requests will be evaluated by the corresponding authors and sponsor on the basis of scientific merit, methodological feasibility, consistency with participantsʼ informed consent and protection of participant confidentiality. Applicants will normally be informed of the decision within 8 weeks. Approved access will be subject to execution of a data-use agreement, and data will be shared through a secure data-transfer process. Source data are provided with this paper.

## Extended data

**Extended Data Table 1:** Primary and key secondary efficacy endpoints at week 32 under the treatment-policy estimand

Endpoint	Safiglipron 30 mg (N=202)	Safiglipron 60 mg (N=203)	Safiglipron 90 mg (N=203)	Dapagliflozin 10 mg (N=202)
**Primary endpoint: HbA1c change from baseline at week 32, percentage points**				
LS mean (SE)	−1.58 (0.07) n=186	−1.42 (0.07) n=183	−1.53 (0.08) n=179	−1.27 (0.07) n=193
Difference vs dapagliflozin (95% CI)	−0.31 (−0.49, −0.13)	−0.14 (−0.34, 0.06)	−0.25 (−0.45, −0.05)	NA
**Key secondary endpoints**				
**Proportion of participants achieving HbA1c <7.0% at week 32**				
% (95% CI)	52.5 (45.35, 59.53) n=186	48.8 (41.71, 55.86) n=183	53.7 (46.58, 60.70) n=179	38.1 (31.39, 45.20) n=193
Odds ratio vs dapagliflozin (95% CI)	1.84 (1.18, 2.87)	1.58 (1.02, 2.45)	2.07 (1.33, 3.22)	NA
**Proportion of participants achieving HbA1c ≤6.5% at week 32**				
% (95% CI)	39.6 (32.81, 46.71) n=186	35.5 (28.90, 42.47) n=183	41.9 (35.00, 48.98) n=179	17.8 (12.81, 23.81) n=193
Odds ratio vs dapagliflozin (95% CI)	3.10 (1.92, 5.03)	2.57 (1.59, 4.14)	3.48 (2.17, 5.57)	NA
**Fasting plasma glucose change from baseline at week 32, mmol/L**				
LS mean (SE)	−2.27 (0.13) n=187	−2.20 (0.15) n=183	−2.37 (0.16) n=179	−2.00 (0.13) n=193
Difference vs dapagliflozin (95% CI)	−0.27 (−0.63, 0.09)	−0.20 (−0.59, 0.19)	−0.37 (−0.79, 0.05)	NA
**Bodyweight percentage change from baseline at week 32, %**				
LS mean (SE)	−2.26 (0.32) n=187	−3.96 (0.32) n=183	−4.37 (0.31) n=179	−3.66 (0.31) n=193
Difference vs dapagliflozin (95% CI)	1.40 (0.53, 2.27)	−0.30 (−1.18, 0.57)	−0.72 (−1.59, 0.16)	NA

Data are presented according to the treatment-policy estimand. Least-squares means, treatment differences and corresponding 95% confidence intervals for changes from baseline in glycated hemoglobin, fasting plasma glucose and bodyweight were estimated using mixed models for repeated measures. Proportions of participants achieving HbA1c targets of <7.0% and ≤6.5%, odds ratios and corresponding 95% confidence intervals were estimated using generalized estimating equations. Missing data were handled using retrieved-dropout or return-to-baseline imputation as prespecified; estimates across imputed datasets were combined using Rubin’s rules. CI=confidence interval; FPG=fasting plasma glucose; GEE=generalized estimating equation; HbA1c=glycated hemoglobin; LS=least squares; MMRM=mixed model for repeated measures; N=number of randomized participants; n=number of participants contributing to the displayed analysis; NA=not applicable; SE=standard error.

**Extended Data Table 2:** Treatment-emergent adverse events leading to permanent treatment discontinuation during the total treatment period

Category	Safiglipron 30 mg (N=202)	Safiglipron 60 mg (N=202)	Safiglipron 90 mg (N=203)	Dapagliflozin 10 mg (N=202)
Any treatment-emergent adverse event leading to permanent discontinuation	8 (4.0)	8 (4.0)	8 (3.9)	3 (1.5)
Gastrointestinal disorders	6 (3.0)	7 (3.5)	7 (3.4)	0
Vomiting	2 (1.0)	4 (2.0)	5 (2.5)	0
Nausea	1 (0.5)	5 (2.5)	4 (2.0)	0
Flatulence	1 (0.5)	1 (0.5)	0	0
Abdominal distension	1 (0.5)	0	1 (0.5)	0
Abdominal discomfort	0	0	1 (0.5)	0
Diarrhea	1 (0.5)	0	0	0
Acute pancreatitis	0	1 (0.5)	0	0
Upper abdominal pain	0	1 (0.5)	0	0
Duodenogastric reflux	1 (0.5)	0	0	0
Gastric ulcer	1 (0.5)	0	0	0
Gastro-oesophageal reflux disease	0	0	1 (0.5)	0
Dyspepsia	0	1 (0.5)	0	0

Data are n (%) unless otherwise stated. Analyses used the safety population, which included all randomized participants who received at least one dose of study treatment; participants were analyzed according to the randomized treatment group. Percentages use the number of participants in each group as the denominator. The total treatment period extended from the first dose through the protocol-defined safety observation window. Events are listed by Medical Dictionary for Regulatory Activities preferred term, with the gastrointestinal-disorders system organ class also shown. Participants could have more than one event.

**Extended Data Table 3:** Postbaseline liver-function laboratory abnormalities during the total treatment period

	Safiglipron 30 mg (N=202)	Safiglipron 60 mg (N=202)	Safiglipron 90 mg (N=203)	Dapagliflozin 10 mg (N=202)
ALT >3×ULN	3 (1.5)	3 (1.5)	0	5 (2.5)
ALT >5×ULN	0	1 (0.5)	0	2 (1.0)
AST >3×ULN	1 (0.5)	1 (0.5)	1 (0.5)	2 (1.0)
AST >5×ULN	0	0	0	1 (0.5)
Total bilirubin >2×ULN	0	3 (1.5)	4 (2.0)	1 (0.5)
ALT or AST >3×ULN	4 (2.0)	3 (1.5)	1 (0.5)	5 (2.5)
ALT or AST >3×ULN and TBIL >2×ULN	0	0	0	0

Data shown are the proportions of participants with maximum post‑baseline hepatic transaminase and total bilirubin levels exceeding the specified thresholds, as number (percentage). ULN=upper limit of normal range.
