# Supplementary material for: Oral small-molecule GLP-1RA safiglipron versus dapagliflozin in type 2 diabetes: a randomized, double-blind, active-comparator-controlled phase 3 trial
Source: Nat Med. 2026 Oct 5;32(11):4340–9. doi: 10.1038/s41591-026-04713-y (PMC13768175; doi:10.1038/s41591-026-04713-y)
Supplement: Supplementary file 1 [file 41591_2026_4713_MOESM1_ESM.pdf]

nature medicine

Supplementary information https://doi.org/10.1038/s41591-026-04713-y

Oral small-molecule GLP-1RA safiglipron versus dapagliflozin in type 2 diabetes: a randomized, double-blind, active-comparator-controlled phase 3 trial

In the format provided by the authors and unedited

Contents

Supplementary Methods—Detailed Statistical Analysis

Supplementary Figures

Supplementary Fig. 1 | Seven-point self-monitored blood glucose profiles at baseline and week 52

Supplementary Fig. 2 | HbA1c distributions and target attainment at week 32 under the efficacy estimand

Supplementary Tables

Supplementary Table 1 | Complete membership and affiliations of the OUTSTAND-2 Investigator Group

Supplementary Table 2 | Prespecified intercurrent events through weeks 32 and 52

Supplementary Table 3 | Primary and sensitivity analyses of HbA1c change from baseline at week 32 under the primary non-inferiority estimand

Supplementary Table 4 | Other supportive and exploratory efficacy endpoints at week 32 under the efficacy estimand (Supplementary estimand 2)

Supplementary Table 5 | Other secondary and exploratory efficacy endpoints at week 52 under the efficacy estimand (Supplementary estimand 2)

Supplementary Table 6 | Model-based changes from baseline in lipid parameters, systolic blood pressure and urinary albumin-to-creatinine ratio at weeks 32 and 52

Supplementary Table 7 | Diabetes Treatment Satisfaction Questionnaire status scores through week 52

Supplementary Table 8 | Gastrointestinal-related treatment-emergent adverse events by maximum severity during the 52-week treatment period

Supplementary Table 9 | Change from baseline in the worst diabetic retinopathy stage during the 52-week treatment period

Supplementary Table 10 | Serious treatment-emergent adverse events during the 52-week treatment period

Supplementary Table 11 | Treatment-emergent adverse events leading to study treatment discontinuation during the 52-week treatment period

Supplementary Table 12 | Selected hepatic, pancreatic and thyroid laboratory parameters through week 52

Supplementary Table 13 | Vital signs through week 52

Supplementary Table 14 | Scheduled 12-lead electrocardiographic parameters through week 52

Supplementary Table 15 | Post hoc site-adjusted comparisons of treatment-related TEAEs and TEAEs leading to permanent treatment discontinuation during the 52-week treatment period

Supplementary References

Trial Protocol

Statistical Analysis Plan

Supplementary Methods—Detailed Statistical Analysis

Estimands and intercurrent events

Three prespecified estimand frameworks addressed complementary clinical questions under ICH E9(R1). For week-32 endpoints, intercurrent events were assessed from baseline through the observed or planned week-32 HbA1c assessment date; for week-52 endpoints, the corresponding window extended through the observed or planned week-52 HbA1c assessment date. Prespecified intercurrent events are summarized in Supplementary Table 2.

Primary estimand: confirmatory non-inferiority

The primary estimand evaluated the difference between each safiglipron dose and dapagliflozin in mean change from baseline in HbA1c at week 32 in the intention-to-treat population. Permanent discontinuation of randomized treatment (intercurrent event 1) and initiation of additional antihyperglycemic medication for more than 14 cumulative days (intercurrent event 2) were each handled using a hypothetical strategy: observations on or after the earliest qualifying intercurrent event were excluded from the analysis. Insufficient exposure was not part of the primary-estimand definition.

Supplementary estimand 1: treatment-policy

Supplementary estimand 1 addressed the treatment-policy question for the superiority comparison on the primary endpoint, the key secondary endpoints in the fixed sequence and prespecified treatment-policy analyses of week-52 endpoints. Outcomes were included irrespective of permanent discontinuation of randomized treatment or initiation of additional antihyperglycemic medication. Insufficient exposure was not an intercurrent event in this estimand. Missing outcomes after application of the treatment-policy strategy were handled using the retrieved-dropout framework, with return-to-baseline used when retrieved-dropout imputation was unsupported, as detailed below.

Supplementary estimand 2: efficacy

Supplementary estimand 2 addressed an efficacy question. It applied hypothetical strategies to permanent treatment discontinuation, initiation of additional antihyperglycemic medication and insufficient study-drug exposure before the endpoint. For the core period, insufficient exposure was defined from week 20 onward as actual exposure during the 90 days before the visit being <50% of target exposure or more than half of the expected pharmacokinetic measurements during that interval being below the lower limit of quantification. For the extension period, insufficient exposure was defined as actual exposure during the 90 days before the visit being <50% of target exposure. Data on or after the earliest qualifying intercurrent event were excluded, and remaining missingness was handled by the prespecified model under a missing-at-random assumption.

Analysis populations and general conventions

The intention-to-treat population included all randomized participants, analyzed according to randomized group. The safety population included all randomized participants who received at least one dose of study treatment, analyzed according to randomized group. All efficacy analyses were based on the intention-to-treat population unless otherwise specified, and all safety analyses were based on the safety population. The baseline for efficacy and safety variables was generally the last non-missing assessment before the first dose in the core treatment period, including same-day predose information under the prespecified ordering rules. Efficacy visit windows and selection rules were prespecified; central-laboratory results were used for endpoints for which both central and local results were available. Missing safety data were not imputed. Analyses were performed using SAS version 9.4 or later.

Sample size and power

A total of 800 participants (200 per group) was planned. For non-inferiority, assuming no true between-group difference, a common standard deviation of 1.1 percentage points and a non-inferiority margin of 0.4 percentage points, this sample size provided 87% overall power to establish non-inferiority for all three safiglipron doses versus dapagliflozin at a one-sided significance level of 0.025. Under an assumed difference of −0.4 percentage points for each safiglipron group versus dapagliflozin with the same standard deviation, the planned sample size also provided 87% overall power for the three superiority comparisons at the same one-sided significance level.

Primary analysis

The primary endpoint was change from baseline in HbA1c at week 32. Under the primary estimand, observations at weeks 4, 8, 12, 16, 20, 26 and 32 were analyzed using a mixed model for repeated measures estimated by restricted maximum likelihood. The model included baseline HbA1c as a covariate and treatment group, visit and treatment-by-visit interaction as fixed effects. Within-

participant covariance was modeled as unstructured, with compound symmetry used if the unstructured model did not converge. Observations on or after the earliest of intercurrent events 1 or 2 were set to missing; remaining missing data were handled by the model under a missing-at-random assumption. Least-squares mean changes, standard errors and two-sided 95% confidence intervals were estimated for each group and for each safiglipron–dapagliflozin contrast. Non-inferiority was established when the upper limit of the two-sided 95% confidence interval for the treatment difference was below 0.4 percentage points, corresponding to a one-sided alpha of 0.025.

Sensitivity and supplementary analyses of the primary endpoint

Sensitivity analysis 1 used multiple imputation of missing HbA1c values followed by analysis of covariance with baseline HbA1c as a covariate and treatment group as a fixed effect. Sensitivity analysis 2 used prespecified delta-adjusted multiple imputation in a two-dimensional tipping-point analysis: paired penalties were applied to imputed outcomes in the safiglipron and dapagliflozin groups, each completed dataset was analyzed using the primary mixed-model framework and estimates were combined using Rubin's rules. The delta pairs are not interpretable as a single scalar tipping threshold.

For Supplementary estimand 1, Markov-chain Monte Carlo imputation first created monotone missingness in 30 datasets using the prespecified random seed 7535302. Missing values unrelated to an intercurrent event were imputed under missing at random. Within each randomized group and intercurrent-event category, retrieved-dropout imputation was used when retrieved outcomes were available for at least 30% of participants with that event and the imputation model was estimable; otherwise, return-to-baseline imputation was used. Each completed dataset was analyzed by mixed modeling for repeated measures and results were combined using Rubin's rules. This treatment-policy analysis supported the prespecified superiority comparison of the primary endpoint.

For Supplementary estimand 2, observations on or after the earliest of intercurrent events 1–3 were set to missing. The processed data were analyzed using the same restricted maximum-likelihood mixed-model structure as the primary analysis, with remaining missing values handled under missing at random.

Fixed-sequence testing and multiplicity

A prespecified fixed-sequence procedure controlled the family-wise type I error at a one-sided alpha of 0.025. The first subscript in each hypothesis identifier denoted dose (1, 90 mg; 2, 60 mg; 3, 30 mg), and the second denoted endpoint. The complete testing order was H11 → H21 → H31 → H12 → H22 → H32 → H13 → H23 → H33 → H14 → H24 → H34 → H15 → H25 → H35 → H16 → H26 → H36. These hypotheses comprised, in sequence, non-inferiority for week-32 HbA1c change under the primary estimand; superiority for week-32 HbA1c change under Supplementary estimand 1; superiority for HbA1c <7.0%; superiority for HbA1c ≤6.5%; superiority for fasting plasma glucose change; and superiority for percentage change in bodyweight, with doses tested from 90 mg to 30 mg within each endpoint family. Testing proceeded only if the preceding hypothesis was confirmed and stopped at the first one-sided P value ≥0.025; hypotheses after a stop were not confirmatory.

Secondary, supportive and exploratory endpoints

Key secondary week-32 endpoints were analyzed under Supplementary estimand 1 for the fixed-sequence framework. HbA1c target attainment (<7.0% and ≤6.5%) was analyzed using generalized estimating equations after application of the prespecified treatment-policy missing-data procedure. Continuous key secondary endpoints, including fasting plasma glucose change and percentage change in bodyweight, were analyzed using mixed models for repeated measures with the corresponding baseline value as a covariate and treatment, visit and treatment-by-visit interaction as fixed effects; the randomization stratification factor was included as prespecified.

Supportive week-32 endpoints were analyzed under Supplementary estimand 2 unless explicitly designated descriptive. Continuous endpoints with repeated measures used the corresponding mixed-model framework; binary endpoints used generalized estimating equations. Week-52 HbA1c, fasting plasma glucose and bodyweight endpoints had prespecified treatment-policy analyses using the Supplementary estimand 1 retrieved-dropout/return-to-baseline procedure and parallel efficacy analyses under Supplementary estimand 2. Selected insulin, C-peptide, HOMA, lipid and urinary albumin-to-creatinine ratio endpoints were log-transformed before modeling and back-transformed for presentation. The seven-point self-monitored blood-glucose profile was summarized from the prespecified seven sampling times; Diabetes Treatment Satisfaction Questionnaire outcomes were summarized descriptively using observed data without imputation.

Exploratory blood-pressure endpoints were analyzed using the Supplementary estimand 2 mixed-model framework. Lipid and

urinary albumin-to-creatinine ratio endpoints were log-transformed and analyzed using mixed models with back-transformed geometric percentage changes and contrasts. Prespecified subgroup analyses of the primary endpoint used the primary-estimand and Supplementary estimand 1 model frameworks. Subgroup and interaction analyses were exploratory and were not adjusted for multiplicity.

Safety analyses

Safety analyses were descriptive and used the safety population; no estimand terminology was applied. Treatment-emergent adverse events were events that occurred or worsened on or after the first dose of study treatment. Events were coded using the Medical Dictionary for Regulatory Activities and summarized by system organ class and preferred term for the core and total treatment periods. Prespecified summaries included all events, treatment-related events, serious events and adverse events of special interest; events leading to temporary interruption, dose reduction or permanent discontinuation; maximum severity; events occurring in at least 5% of participants; hypoglycemic events; and selected gastrointestinal events by preferred term, time and maximum severity. A participant with repeated occurrences of the same preferred term was counted once in participant-level incidence summaries, using the maximum recorded severity for severity summaries.

Laboratory measurements, vital signs and electrocardiographic findings were summarized descriptively by visit and change from baseline. Retinopathy findings were summarized by eye and by worst postbaseline change from baseline. Missing safety data were not imputed. Exposure and adherence were summarized descriptively; adherence was calculated as actual tablets taken divided by planned tablets, multiplied by 100%, with protocol categories of <80%, 80–120% and >120%.

Daily gastrointestinal adverse-event derivation

The prespecified gastrointestinal event set included nausea, vomiting, diarrhea, constipation, abdominal distension and decreased appetite. A participant was counted once per preferred term on each study day; when overlapping records for the same participant and preferred term differed in severity, the maximum severity for that day was used. Daily prevalence used the randomized-treatment safety-population denominator, and events were included irrespective of investigator-assessed relationship to study treatment. Cumulative incidence over time and preferred-term summaries by maximum severity were prepared separately.

Supplementary Fig. 1 | Seven-point self-monitored blood glucose profiles at baseline and week 52

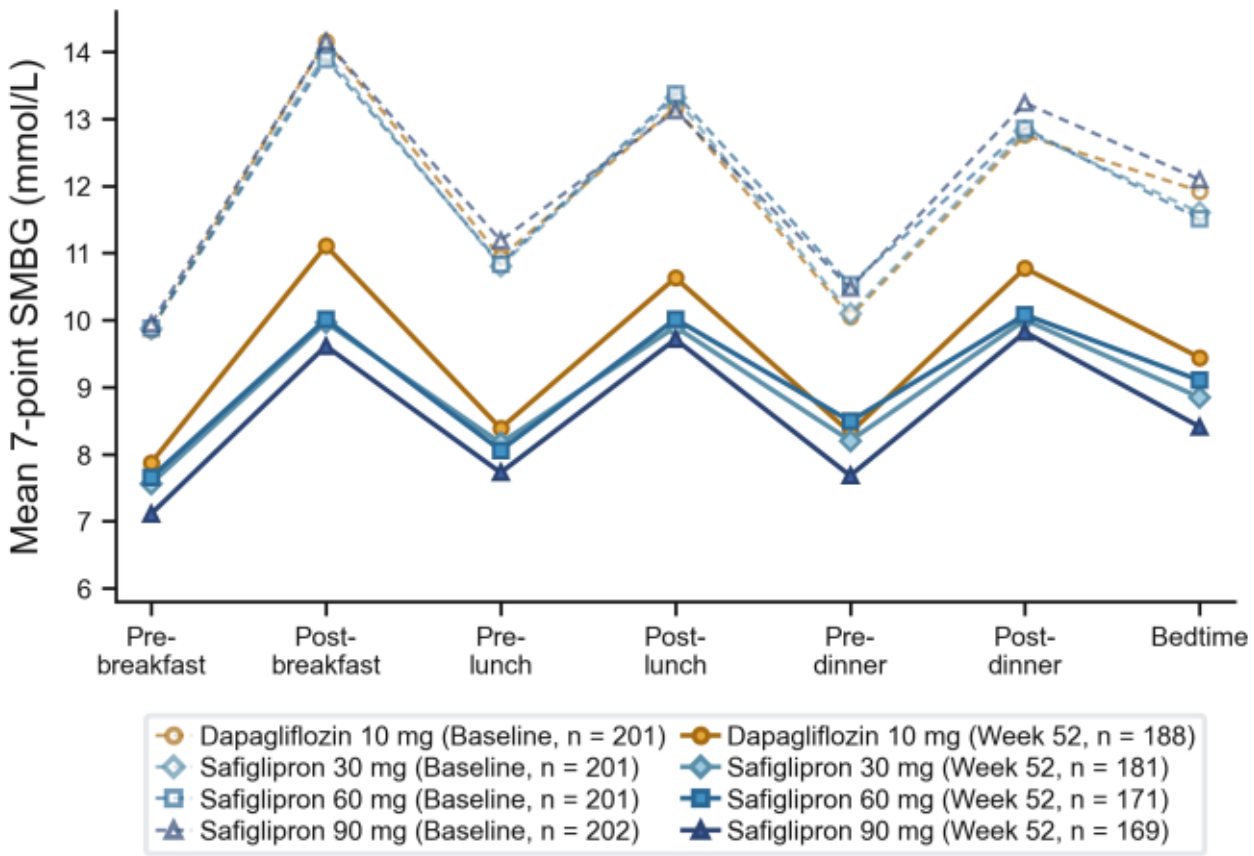

Observed mean seven-point SMBG profiles are shown at baseline (dashed lines and open markers) and week 52 (solid lines and filled markers), measured before and 2 h after each meal and at bedtime. Repeated diary-day measurements within a participant, visit and scheduled timepoint were averaged before treatment-group means were calculated. Exact n values are shown in the legend and denote participants with available observed SMBG data at the indicated visit; for dapagliflozin 10 mg and safiglipron 30 mg, 60 mg and 90 mg, respectively, n was 201, 201, 201 and 202 at baseline and 188, 181, 171 and 169 at week 52. Analyses used the intention-to-treat population. SMBG=self-monitored blood glucose.

Supplementary Fig. 2 | HbA1c distributions and target attainment at week 32 under the efficacy estimand

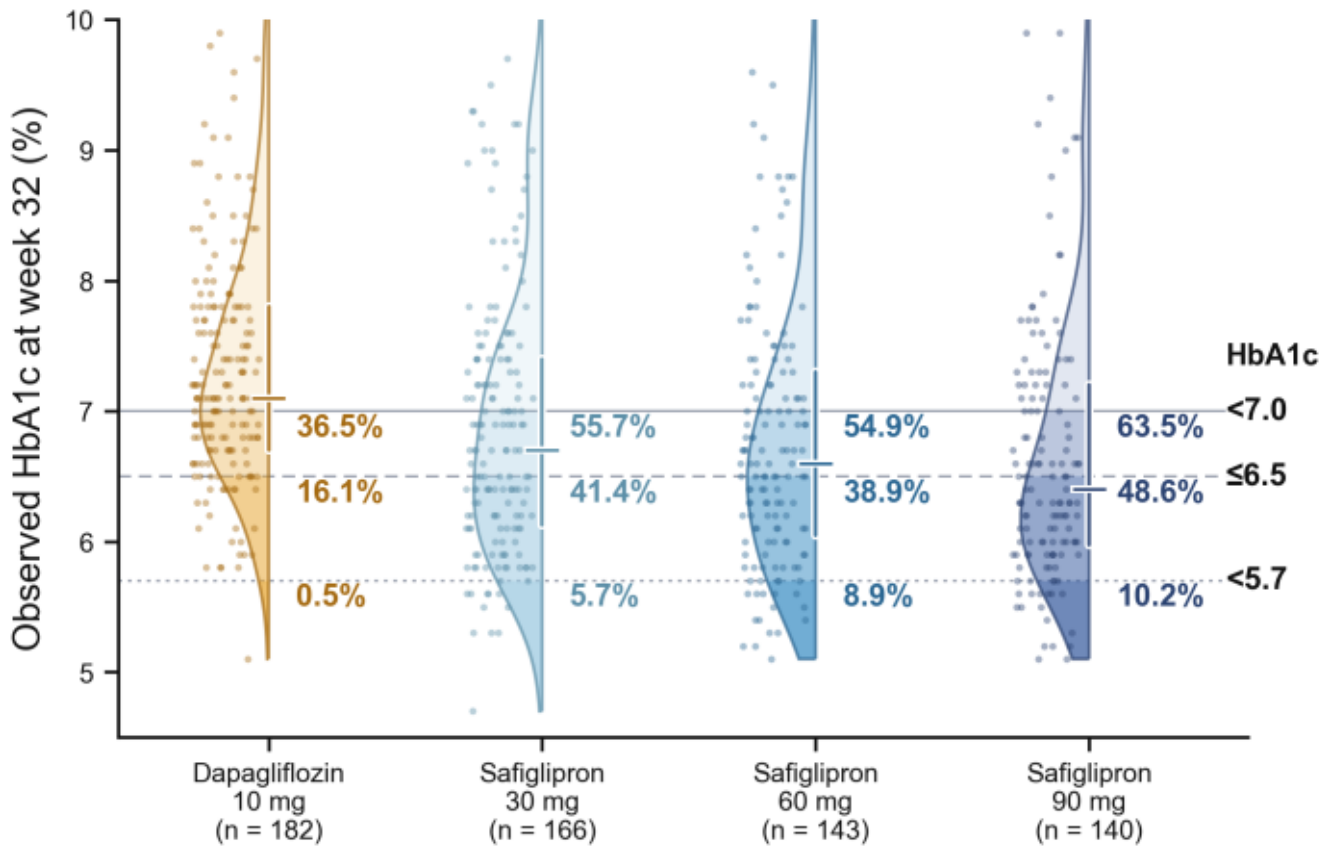

The efficacy estimand used a hypothetical strategy for prespecified intercurrent events. Half-violins and points show observed HbA1c values, vertical lines indicate interquartile ranges and short horizontal lines indicate medians. Colored percentages are GEE marginal estimates of the proportions of participants achieving HbA1c <7.0%, HbA1c ≤6.5% and HbA1c <5.7%; they are not raw proportions calculated from the plotted observations. Horizontal lines mark these targets. Post-intercurrent-event data were excluded. Analyses used the intention-to-treat population. GEE=generalized estimating equation; HbA1c=glycated hemoglobin.

Supplementary Table 1 | Complete membership and affiliations of the OUTSTAND-2 Investigator Group
This table lists all members of the OUTSTAND-2 Investigator Group and their corresponding institutional affiliations. Each study site listed represents the affiliation of the corresponding investigator. A total of 98 sites participated in the study, and each site screened and obtained informed consent from at least one participant.

Study site	Principal investigator	Participants enrolled, n	Participants who completed treatment, n
Beijing Hospital	Lixin Guo	12	11
Anhui Provincial Hospital	Wei Wang	13	13
Xuancheng People's Hospital	Sihong Wang	11	10
The First Affiliated Hospital of Bengbu Medical College	Lei Yu	18	14
Chengdu Fifth People's Hospital	Hongyi Cao	1	1
Chengde Central Hospital	Tie Zhai	11	7
Chongqing University Affiliated Fuling Hospital	Guangxiu Wu	17	14
The Second Hospital Affiliated of Chongqing Medical University	Wei Cheng	10	8
Datong Third People's Hospital	Yan Liu	5	4
Zhongda Hospital Southeast University	Ling Li	10	9
Qingpu Branch of Zhongshan Hospital, Fudan University	Min Zhang	11	10
Gansu Provincial Hospital	Jinyang Wang	9	9
Ganzhou People's Hospital	Shumei Zhong	8	6
Affiliated Hospital of Guangdong Medical University	Juhong Yang	2	2
Nanxishan Hospital of Guangxi Zhuang Autonomous Region (Guangxi Second People's Hospital)	Xiaochang Bin	10	10
The second Affiliated of Harbin Medical University	Hong Qiao	3	3
The Fourth Hospital of Harbin Medical University	Zhifeng Cheng	8	8
Hainan General Hospital	Kaining Chen	1	1
The First Affiliated Hospital of Hainan Medical University	Xiaopan Chen	4	2
Handan First Hospital	Haifang Wang	7	6
Hangzhou First People's Hospital	Xianfeng Zhang	4	4
Cangzhou Integrated Chinese and Western Medicine Hospital, Hebei Province	Xiuhai Su	12	9
The Second Hospital of Hebei Medical University	Lihui Zhang	17	16
The First Affiliated Hospital of Henan University of Science & Technology	Liujun Fu	4	4
Huangshi Central Hospital	Xiaowen Chen	12	12

Study site	Principal investigator	Participants enrolled, n	Participants who completed treatment, n
Huizhou Third People's Hospital	Guiping Li	14	14
Huizhou central People's Hospital	Zhan Lin	7	7
Central Hospital Affiliated to Shandong First Medical University	Xiaolin Dong	35	30
Jiangsu Province Hospital	Tao Yang	3	3
The Second Affiliated Hospital of Air Force Medical University	Bin Gao	8	6
Kunming Medical University Hospital No. 1	Huifang Li	5	3
Liuzhou People's Hospital	Na Liu	13	13
The First Affiliated Hospital of Nanyang Medical College	Dexue Liu	15	13
Hospital No. 1 Affiliated Medical College of Baotou, Inner Mongolia University of Science and Technology	Feng Wei	13	12
Inner Mongolia People's Hospital	Yunfeng Li	7	5
The First Affiliated Hospital of Ningbo University	Li Li	4	2
PingXiang People's Hospital	Yawei Zhang	10	10
Fisrt Hospital of Qinhuangdao	Bowei Liu	2	1
Zhongshan Hospital Xiamen University	Xiaomin Chen	3	3
Shanxi Bethune Hospital	Shiwei Liu	13	10
Shanghai Tenth People's Hospital	Haibing Chen	8	8
Putuo District Central Hospital, Shanghai	Tao Lei	14	8
Shaoyang Central Hospital	Bin Zhou	5	4
Siping Central People's Hospital	Li Sun	5	5
Hospital No. 1 Affiliated to Tianjin University of Chinese Medicine	Bin Wang	13	8
Tonghua City Center Hospital	Wenyan Fu	3	3
Weifang People's Hospital	Haixia Liu	10	8
Weifang City Intermediate Hospital	Jinling Liu	16	13
The Affiliated Hospital of Xuzhou Medical University	Meng Zhao	29	26
Second People's Hospital of Yibin City	Junling Gu	7	6
Yichang Central People's Hospital	Chaoyang Zeng	21	17
Xiling Branch of Yichang Central People's Hospital	Jun Zeng	16	15
The Third Hospital of ChangSha	Debin Huang	24	21
The Second Affiliated Hospital of Zhejiang University School of Medicine	Chao Zheng	1	1

Study site	Principal investigator	Participants enrolled, n	Participants who completed treatment, n
ShengJing Hospital, Affiliated Medical University of China	Xiaoguang Shi	16	14
Zhumadian Central Hospital	Lianwei Wang	13	12
The First People’s Hospital of Changde City	Haifeng Zhou	6	5
Huadong Hospital, Fudan University	Bin Lu	3	3
The First Affiliated Hospital of Guangxi Medical University	Min Liang	3	3
Huai'an Hospital Affiliated Xuzhou Medical College	Wen Hu	25	23
Hospital Affiliated toJiangsu University	Ruirong Pan	16	16
Yueyang Central Hospital	Xianghua Zhang	13	10
The Second Affiliated Hospital of Zhengzhou University	Qingju Li	12	9
The first People's Hospital of Lianyungang	Yuan Hui	3	3
The First Affiliated Hospital of Xi’an Medical University	Ya Li	13	10
Tongji Hospital, Shanghai	Junfeng Han	11	10
The Second Affiliated Hospital of Anhui Medical University	Tianrong Pan	7	5
Affiliated Hospital of Nantong University	Yunjuan Gu	10	8
Jiangxi People's Hospital	Zongyou Cheng	3	3
Luoyang Third People's Hospital	Junhang Tian	4	4
Beijing Pinggu Hospital	Yufeng Li	7	7
The Second Affiliated Hospital of Baotou Medical College, Inner Mongolia University of Science and Technology	Tao Ning	6	6
People's Hospital of Hunan Province	Xinlan Zhao	1	1
Renji Hospital, Shanghai Jiao Tong University School of Medicine	Jing Ma	1	1
Ma'anshan People's Hospital	Weihong Chen	7	5
Shanxi Cardiovascular Hospital	Lin Guo	2	2
Hospital Affiliated with Yan'an University	Shuangyan Lyu	8	7
Baogang Hospital, Inner Mongolia	Ziling Li	2	1
Jinshan Hospital of Fudan University	Xiaohong Shi	1	1
Shanghai Seventh People's Hospital	Xiaohua Li	2	1
The First People's Hospital of XiaoShan HangZhou	Jun Chen	1	1
Huzhou Center Hospital	Jianping Yao	2	1

Study site	Principal investigator	Participants enrolled, n	Participants who completed treatment, n
The Second Hospital of Jiaxing	Qiong Yu	2	2
The People's Hospital of Longhua.Shenzhen	Xiaosu Bai	2	2
Binzhou Medical University Hospital	Haibo Xue	6	5
Jingjiang People's Hospital	Yuan Gao	17	16
Wuxi People's Hospital Affiliated to Nanjing Medical University	Xiaowei Zhu	1	1
The Third People’s Hospital of Hubei Province	Juping Hu	2	2
Jinzhou Central Hospital	Jiao'e Zeng	2	1
Xiangyang Central Hospital	Shaoyong Xu	1	1
The First Affiliated Hospital of Gannan Medical University	Shihua Hong	5	5
General Hospital of Pingxiang Mining Group Co., Ltd	Lijuan Liu	1	1
The First Hospital Affiliated of Chongqing Medical University	Qifu Li	5	5
Nanyang Second General Hospital	Junjie Shang	2	2
Zhengzhou People's Hospital	Qijuan Dong	7	5
First Hospital of ShanXi Medical University	Yan Wang	1	1
Xiangtan City Center Hospital	Guohua Li	8	7
Tongling People's Hospital	Chongzhou Dong	6	5

Data are the numbers of randomized participants enrolled and completing the 52-week randomized treatment period at each site; n denotes the site-specific count.

Supplementary Table 2 | Prespecified intercurrent events through weeks 32 and 52

Intercurrent event	Safiglipron 30 mg (N=202)	Safiglipron 60 mg (N=203)	Safiglipron 90 mg (N=203)	Dapagliflozin 10 mg (N=202)
**By week 32**				
**Participants with any prespecified intercurrent event**	**42 (20.8)**	**63 (31.0)**	**67 (33.0)**	**25 (12.4)**
Permanent discontinuation of randomized treatment	19 (9.4)	30 (14.8)	31 (15.3)	10 (5.0)
Initiation of additional glucose-lowering medication	4 (2.0)	2 (1.0)	2 (1.0)	1 (0.5)
Insufficient study-drug exposure before the visit	21 (10.4)	35 (17.2)	41 (20.2)	15 (7.4)
**By week 52**				
**Participants with any prespecified intercurrent event**	**52 (25.7)**	**73 (36.0)**	**72 (35.5)**	**33 (16.3)**
Permanent discontinuation of randomized treatment	23 (11.4)	35 (17.2)	36 (17.7)	14 (6.9)
Initiation of additional glucose-lowering medication	14 (6.9)	11 (5.4)	5 (2.5)	5 (2.5)
Insufficient study-drug exposure before the visit	22 (10.9)	35 (17.2)	41 (20.2)	15 (7.4)

Data are n (%) in the intention-to-treat population. Percentages use the randomized group size (N) as the denominator.

Participants could experience more than one type of intercurrent event; therefore, category counts may exceed the number with any prespecified intercurrent event.

Initiation of additional glucose-lowering medication was defined as use for more than 14 cumulative days. Insufficient study-drug exposure before the visit was assessed according to the prespecified exposure criteria during the 90 days before the relevant visit.

Supplementary Table 3 | Primary and sensitivity analyses of HbA1c change from baseline at week 32 under the primary non-inferiority estimand

Analysis	Safiglipron 30 mg (N=202)	Safiglipron 60 mg (N=203)	Safiglipron 90 mg (N=203)	Dapagliflozin 10 mg (N=202)
Confirmatory primary MMRM	−1.58 (−1.73 to −1.43) −0.30 (−0.51 to −0.09); P<0.0001 n=179	−1.50 (−1.66 to −1.35) −0.22 (−0.44 to −0.01); P<0.0001 n=171	−1.68 (−1.84 to −1.53) −0.40 (−0.62 to −0.19); P<0.0001 n=168	−1.28 (−1.43 to −1.13) n=190
Sensitivity analysis 1: multiple imputation followed by ANCOVA	−1.59 (−1.74 to −1.44) −0.32 (−0.52 to −0.11); P<0.0001 n=179	−1.53 (−1.68 to −1.38) −0.25 (−0.46 to −0.05); P<0.0001 n=171	−1.68 (−1.83 to −1.53) −0.41 (−0.62 to −0.20); P<0.0001 n=168	−1.27 (−1.42 to −1.13) n=190
Sensitivity analysis 2: Pareto-minimal first-failure boundary cells	(+1.1 / −1.1); 0.14; P=0.0258	(+0.8 / −0.6); 0.16; P=0.0298 (+0.7 / −0.8); 0.15; P=0.0253	(+0.8 / −0.6); 0.12; P=0.0267	NA

Data are from the intention-to-treat (ITT) population under the primary non-inferiority estimand. Each model-based cell shows the number of participants with an observation at week 32 (n), the least-squares mean change from baseline in HbA1c (95% CI), and, for safiglipron, the difference versus dapagliflozin (95% CI) with the one-sided P value for non-inferiority. The prespecified non-inferiority margin was 0.4 percentage points.

The primary analysis used a restricted maximum-likelihood mixed model for repeated measures. Sensitivity analysis 1 used multiple imputation followed by analysis of covariance. Sensitivity analysis 2 used delta-adjusted multiple imputation; each entry gives the safiglipron delta/dapagliflozin delta, the difference versus dapagliflozin and the one-sided P value at a Pareto-minimal first-failure boundary cell. The two cells shown for 60 mg are incomparable boundary pairs.

ANCOVA=analysis of covariance. CI=confidence interval. HbA1c=glycated hemoglobin. ITT=intention-to-treat. LS=least-squares. MMRM=mixed model for repeated measures. NA=not applicable.

Supplementary Table 4 | Other supportive and exploratory efficacy endpoints at week 32 under the efficacy estimand (Supplementary estimand 2)

Endpoint	Safiglipron 30 mg (N=202)	Safiglipron 60 mg (N=203)	Safiglipron 90 mg (N=203)	Dapagliflozin 10 mg (N=202)
**Body weight change from baseline at week 32, kg**				
LS mean (SE)	−1.70 (0.21) n=167	−2.55 (0.22) n=143	−3.08 (0.22) n=140	−2.69 (0.21) n=183
Difference (95% CI, vs. dapagliflozin)	0.99 (0.40, 1.57)	0.14 (−0.45, 0.74)	−0.38 (−0.98, 0.21)	NA
**Waist circumference change from baseline at week 32*, cm**				
Mean (SD)	−2.03 (3.05) n=187	−2.37 (3.73) n=183	−3.25 (4.36) n=179	−2.55 (3.41) n=193
**Proportion of patients achieving glycated hemoglobin target of <5.7% at week 32**				
% (95% CI)	5.7 (3.04, 10.32) n=166	8.9 (5.07, 15.12) n=143	10.2 (6.20, 16.30) n=140	0.5 (0.07, 3.28) n=182
Odds ratio (95% CI, vs. dapagliflozin)	12.67 (1.61, 99.87)	20.59 (2.67, 158.55)	23.94 (3.12, 183.57)	NA
**Fasting serum insulin geometric mean percentage change from baseline at week 32, %**				
% (95% CI)	9.88 (3.23, 16.96) n=167	8.24 (1.26, 15.69) n=142	8.59 (1.56, 16.12) n=140	−23.32 (−27.80, −18.56) n=181
Difference (95% CI, vs. dapagliflozin)	43.29 (31.39, 56.27)	41.15 (29.03, 54.40)	41.61 (29.42, 54.96)	NA
**Fasting serum C-peptide geometric mean percentage change from baseline at week 32, %**				
% (95% CI)	16.74 (11.33, 22.41) n=167	17.27 (11.50, 23.35) n=143	17.02 (11.21, 23.13) n=140	−8.11 (−12.21, −3.81) n=182
Difference (95% CI, vs. dapagliflozin)	27.04 (18.94, 35.68)	27.62 (19.22, 36.60)	27.34 (18.93, 36.35)	NA
**HOMA-IR geometric mean percentage change from baseline at week 32, %**				
% (95% CI)	−16.02 (−22.14, −9.43) n=167	−18.90 (−25.18, −12.10) n=142	−22.49 (−28.52, −15.95) n=140	−38.52 (−42.84, −33.87) n=181

Endpoint	Safiglipron 30 mg (N=202)	Safiglipron 60 mg (N=203)	Safiglipron 90 mg (N=203)	Dapagliflozin 10 mg (N=202)
Difference (95% CI, vs. dapagliflozin)	36.58 (22.98, 51.70)	31.90 (18.33, 47.03)	26.07 (13.05, 40.58)	NA
**HOMA-β geometric mean percentage change from baseline at week 32, %**				
% (95% CI)	76.88 (63.95, 90.82) n=167	77.98 (64.21, 92.90) n=142	98.47 (83.05, 115.20) n=140	11.52 (3.64, 20.01) n=181
Difference (95% CI, vs. dapagliflozin)	58.60 (42.73, 76.24)	59.59 (43.13, 77.93)	77.96 (59.56, 98.49)	NA
**2-hour postprandial serum glucose change from baseline at week 32, mmol/L**				
LS mean (SE)	−4.02 (0.30) n=167	−5.40 (0.32) n=143	−6.36 (0.33) n=139	−2.89 (0.29) n=181
Difference (95% CI, vs. dapagliflozin)	−1.13 (−1.95, −0.31)	−2.52 (−3.37, −1.67)	−3.47 (−4.33, −2.62)	NA
**2-hour postprandial serum insulin geometric mean percentage change from baseline at week 32, %**				
% (95% CI)	58.40 (44.61, 73.51) n=167	35.89 (23.16, 49.93) n=142	25.86 (13.91, 39.06) n=137	11.76 (2.38, 22.00) n=181
Difference (95% CI, vs. dapagliflozin)	41.74 (24.91, 60.83)	21.59 (6.60, 38.69)	12.62 (−1.39, 28.62)	NA
**2-hour postprandial serum C-peptide geometric mean percentage change from baseline at week 32, %**				
% (95% CI)	47.73 (39.11, 56.87) n=167	36.93 (28.37, 46.06) n=143	29.55 (21.36, 38.29) n=139	23.63 (16.69, 30.99) n=181
Difference (95% CI, vs. dapagliflozin)	19.49 (9.94, 29.87)	10.76 (1.57, 20.77)	4.79 (−3.96, 14.33)	NA

Endpoint	Safiglipron 30 mg (N=202)	Safiglipron 60 mg (N=203)	Safiglipron 90 mg (N=203)	Dapagliflozin 10 mg (N=202)
Participants who received rescue therapy by week 32, n (%)	3 (1.5)	2 (1.0)	1 (0.5)	0

Data are from the intention-to-treat population. Unless marked with an asterisk, analyses used Supplementary estimand 2, the efficacy estimand. Values are least-squares mean (SE), geometric mean percentage change from baseline (95% CI), difference versus dapagliflozin (95% CI), model-estimated percentage (95% CI), odds ratio versus dapagliflozin (95% CI), mean (SD), or n (%), as indicated. n denotes participants contributing to the displayed analysis.

Continuous model-based endpoints were analyzed using prespecified mixed models for repeated measures; HbA1c target attainment used generalized estimating equations. Permanent treatment discontinuation, additional antihyperglycemic therapy and insufficient study-drug exposure were handled using hypothetical strategies. Rescue therapy is descriptive. P values are not shown and no multiplicity-adjusted inference was applied.

*Observed-case mean (SD) without imputation for missing data.

CI=confidence interval. GEE=generalized estimating equation. HbA1c=glycated hemoglobin. HOMA-β=homeostatic model assessment of β-cell function. HOMA-IR=homeostatic model assessment of insulin resistance. ITT=intention-to-treat. LS=least-squares. MMRM=mixed model for repeated measures. NA=not applicable. SD=standard deviation. SE=standard error.

Supplementary Table 5 | Other secondary and exploratory efficacy endpoints at week 52 under the efficacy estimand (Supplementary estimand 2)

Endpoint	Safiglipron 30 mg (N=202)	Safiglipron 60 mg (N=203)	Safiglipron 90 mg (N=203)	Dapagliflozin 10 mg (N=202)
**HbA1c change from baseline at week 52, percentage points**				
LS mean (SE)	−1.35 (0.09) n=166	−1.20 (0.09) n=157	−1.43 (0.09) n=163	−1.11 (0.08) n=183
Difference (95% CI, vs. dapagliflozin)	−0.24 (−0.47, −0.01)	−0.09 (−0.33, 0.14)	−0.32 (−0.55, −0.09)	NA
**Fasting plasma glucose change from baseline at week 52*, mmol/L**				
Mean (SD)	−1.94 (2.22) n=183	−1.87 (2.57) n=177	−2.42 (2.33) n=178	−1.98 (1.76) n=192
**Body weight change from baseline at week 52, kg**				
LS mean (SE)	−1.40 (0.23) n=166	−2.27 (0.23) n=156	−2.38 (0.23) n=163	−2.43 (0.22) n=183
Difference (95% CI, vs. dapagliflozin)	1.03 (0.41, 1.65)	0.17 (−0.46, 0.79)	0.05 (−0.57, 0.68)	NA
**Body weight percentage change from baseline at week 52, %**				
LS mean (SE)	−1.85 (0.29) n=166	−3.03 (0.30) n=156	−3.07 (0.30) n=163	−3.35 (0.29) n=183
Difference (95% CI, vs. dapagliflozin)	1.50 (0.70, 2.31)	0.32 (−0.50, 1.13)	0.28 (−0.53, 1.09)	NA
**Waist circumference change from baseline at week 52*, cm**				
Mean (SD)	−1.95 (3.29) n=183	−2.52 (4.06) n=176	−3.07 (4.27) n=178	−2.53 (3.66) n=192
**Participants achieving HbA1c <7.0% at week 52†**				
n (%; 95% CI)	91 (45.0) (38.06, 52.19)	83 (40.9) (34.06, 47.99)	96 (47.3) (40.26, 54.40)	63 (31.2) (24.87, 38.07)
**Participants achieving HbA1c ≤6.5% at week 52†**				
n (%; 95% CI)	62 (30.7) (24.41, 37.55)	62 (30.5) (24.29, 37.38)	68 (33.5) (27.04, 40.44)	24 (11.9) (7.76, 17.16)
**Participants achieving HbA1c <5.7% at week 52†**				
n (%; 95% CI)	5 (2.5) (0.81, 5.68)	11 (5.4) (2.74, 9.49)	13 (6.4) (3.45, 10.70)	2 (1.0) (0.12, 3.53)

Endpoint	Safiglipron 30 mg (N=202)	Safiglipron 60 mg (N=203)	Safiglipron 90 mg (N=203)	Dapagliflozin 10 mg (N=202)
**Fasting serum insulin geometric mean percentage change from baseline at week 52, %**				
% (95% CI)	8.41 (1.78, 15.48) n=165	9.23 (2.33, 16.60) n=153	9.52 (2.75, 16.74) n=163	−20.90 (−25.54, −15.98) n=183
Difference (95% CI, vs. dapagliflozin)	37.07 (25.61, 49.58)	38.10 (26.36, 50.94)	38.47 (26.83, 51.18)	NA
**Fasting serum C-peptide geometric mean percentage change from baseline at week 52, %**				
% (95% CI)	19.87 (14.16, 25.85) n=166	18.93 (13.13, 25.02) n=157	20.16 (14.38, 26.23) n=163	−6.91 (−11.16, −2.46) n=183
Difference (95% CI, vs. dapagliflozin)	28.77 (20.36, 37.76)	27.76 (19.31, 36.80)	29.08 (20.60, 38.15)	NA
**HOMA-β geometric mean percentage change from baseline at week 52, %**				
% (95% CI)	58.33 (46.60, 71.00) n=165	59.37 (47.19, 72.56) n=153	81.72 (68.11, 96.44) n=163	14.76 (6.60, 23.55) n=183
Difference (95% CI, vs. dapagliflozin)	37.97 (24.02, 53.48)	38.87 (24.61, 54.77)	58.35 (42.25, 76.27)	NA
**HOMA-IR geometric mean percentage change from baseline at week 52, %**				
% (95% CI)	−13.12 (−19.42, −6.32) n=165	−12.33 (−18.89, −5.23) n=153	−18.02 (−24.03, −11.54) n=163	−36.60 (−41.01, −31.87) n=183
Difference (95% CI, vs. dapagliflozin)	37.05 (23.49, 52.09)	38.29 (24.38, 53.75)	29.31 (16.45, 43.59)	NA
**2-h postprandial plasma glucose change from baseline at week 52, mmol/L**				
LS mean (SE)	−3.80 (0.30) n=166	−4.42 (0.31) n=157	−5.49 (0.31) n=162	−2.76 (0.29) n=183
Difference (95% CI, vs. dapagliflozin)	−1.04 (−1.86, −0.22)	−1.66 (−2.49, −0.83)	−2.74 (−3.57, −1.91)	NA

Endpoint	Safiglipron 30 mg (N=202)	Safiglipron 60 mg (N=203)	Safiglipron 90 mg (N=203)	Dapagliflozin 10 mg (N=202)
**2-h postprandial serum insulin geometric mean percentage change from baseline at week 52, %**				
% (95% CI)	48.12 (34.86, 62.69)<br>n=164	32.54 (20.22, 46.11)<br>n=152	23.50 (12.29, 35.82)<br>n=161	0.62 (−7.98, 10.01)<br>n=182
Difference (95% CI, vs. dapagliflozin)	47.22 (29.35, 67.56)	31.73 (15.43, 50.32)	22.74 (7.73, 39.85)	NA
**2-h postprandial serum C-peptide geometric mean percentage change from baseline at week 52, %**				
% (95% CI)	42.71 (33.95, 52.04)<br>n=166	32.93 (24.54, 41.89)<br>n=157	29.75 (21.67, 38.37)<br>n=162	17.73 (10.82, 25.07)<br>n=183
Difference (95% CI, vs. dapagliflozin)	21.22 (11.06, 32.30)	12.91 (3.31, 23.41)	10.21 (0.90, 20.38)	NA
Participants who received rescue therapy through week 52‡, n (%)	11 (5.4)	10 (4.9)	4 (2.0)	4 (2.0)

Data are from the intention-to-treat population. Unless marked with a symbol, analyses used Supplementary estimand 2, the efficacy estimand. Values are least-squares mean (SE), geometric mean percentage change from baseline (95% CI), difference versus dapagliflozin (95% CI), mean (SD), n (%; 95% CI), or n (%), as indicated. n beneath an estimate denotes participants with an observation at week 52.

Model-based continuous endpoints were analyzed using prespecified mixed models for repeated measures. Permanent treatment discontinuation, additional antihyperglycemic therapy and insufficient study-drug exposure were handled using hypothetical strategies. Log-transformed endpoints were back-transformed and are presented as geometric mean percentage changes. P values are not shown and no multiplicity-adjusted inference was applied.

*Observed-case mean (SD) without imputation for missing data. †Values are responder n (%; exact two-sided 95% CI) using the randomized treatment-group N as the denominator. ‡Rescue therapy is cumulative through week 52 and descriptive.

CI=confidence interval. HbA1c=glycated hemoglobin. HOMA-β=homeostatic model assessment of β-cell function. HOMA-IR=homeostatic model assessment of insulin resistance. ITT=intention-to-treat. LS=least-squares. MMRM=mixed model for repeated measures. NA=not applicable. SD=standard deviation. SE=standard error.

Supplementary Table 6 | Model-based changes from baseline in lipid parameters, systolic blood pressure and urinary albumin-to-creatinine ratio at weeks 32 and 52

Parameter and visit	Safiglipron 30 mg (N=202)	Safiglipron 60 mg (N=203)	Safiglipron 90 mg (N=203)	Dapagliflozin 10 mg (N=202)
**Triglycerides** **Week 32**	0.23 (−6.17, 7.06) n=166	−0.31 (−7.09, 6.98) n=143	−10.45 (−16.59, −3.85) n=139	−5.61 (−11.41, 0.57) n=181
Triglycerides: difference vs dapagliflozin Week 32	6.18 (−3.10, 16.35) P=0.0991	5.62 (−3.94, 16.13) P=0.1291	−5.13 (−13.74, 4.35) P=0.1390	—
Triglycerides Week 52	−1.55 (−8.01, 5.35) n=166	−0.70 (−7.37, 6.45) n=157	−8.32 (−14.38, −1.82) n=162	−8.31 (−14.08, −2.15) n=182
Triglycerides: difference vs dapagliflozin Week 52	7.37 (−2.25, 17.94) P=0.0688	8.30 (−1.53, 19.12) P=0.0502	−0.01 (−9.01, 9.89) P=0.4995	—
**Total cholesterol** **Week 32**	−2.96 (−5.51, −0.33) n=167	−1.52 (−4.28, 1.31) n=143	−2.39 (−5.15, 0.44) n=139	2.29 (−0.31, 4.96) n=181
Total cholesterol: difference vs dapagliflozin Week 32	−5.13 (−8.58, −1.55) P=0.0027	−3.73 (−7.35, 0.03) P=0.0259	−4.58 (−8.18, −0.84) P=0.0085	—
Total cholesterol Week 52	−0.75 (−3.38, 1.95) n=166	2.24 (−0.53, 5.10) n=157	−0.91 (−3.57, 1.81) n=162	4.06 (1.41, 6.78) n=182
Total cholesterol: difference vs dapagliflozin Week 52	−4.62 (−8.10, −1.00) P=0.0064	−1.74 (−5.38, 2.03) P=0.1796	−4.78 (−8.28, −1.15) P=0.0052	—
**LDL-C** **Week 32**	−5.74 (−9.47, −1.86) n=166	−4.11 (−8.15, 0.10) n=143	−2.69 (−6.82, 1.61) n=139	2.88 (−1.04, 6.95) n=181
LDL-C: difference vs dapagliflozin Week 32	−8.38 (−13.37, −3.11) P=0.0011	−6.80 (−12.04, −1.24) P=0.0086	−5.42 (−10.76, 0.24) P=0.0302	—
LDL-C Week 52	−1.27 (−4.94, 2.55) n=166	0.76 (−3.07, 4.75) n=157	−1.62 (−5.31, 2.22) n=162	4.54 (0.81, 8.41) n=182
LDL-C: difference vs dapagliflozin Week 52	−5.55 (−10.38, −0.46) P=0.0165	−3.61 (−8.60, 1.65) P=0.0873	−5.89 (−10.73, −0.79) P=0.0121	—
**HDL-C** **Week 32**	−0.88 (−3.30, 1.59) n=167	−0.10 (−2.69, 2.56) n=143	0.15 (−2.48, 2.85) n=139	4.34 (1.89, 6.85) n=181

Parameter and visit	Safiglipron 30 mg (N=202)	Safiglipron 60 mg (N=203)	Safiglipron 90 mg (N=203)	Dapagliflozin 10 mg (N=202)
HDL-C: difference vs dapagliflozin<br>Week 32	−5.01 (−8.20, −1.70)<br>P=0.0017	−4.26 (−7.59, −0.80)<br>P=0.0081	−4.02 (−7.38, −0.54)<br>P=0.0121	—
HDL-C<br>Week 52	2.02 (−0.38, 4.48)<br>n=166	1.55 (−0.91, 4.06)<br>n=157	1.56 (−0.86, 4.04)<br>n=162	5.95 (3.56, 8.40)<br>n=182
HDL-C: difference vs dapagliflozin<br>Week 52	−3.71 (−6.84, −0.48)<br>P=0.0124	−4.16 (−7.31, −0.90)<br>P=0.0065	−4.14 (−7.28, −0.91)<br>P=0.0063	—
**Systolic blood pressure**<br>**Week 32**	−2.93 (−4.40, −1.46)<br>n=167	−3.67 (−5.23, −2.11)<br>n=143	−4.53 (−6.10, −2.97)<br>n=140	−4.61 (−6.03, −3.20)<br>n=183
Systolic blood pressure: difference vs dapagliflozin<br>Week 32	1.68 (−0.36, 3.72)<br>P=0.0530	0.94 (−1.16, 3.05)<br>P=0.1896	0.08 (−2.03, 2.19)<br>P=0.4709	—
Systolic blood pressure<br>Week 52	−2.04 (−3.48, −0.61)<br>n=166	−1.15 (−2.62, 0.33)<br>n=157	−3.59 (−5.04, −2.14)<br>n=163	−3.66 (−5.04, −2.28)<br>n=183
Systolic blood pressure: difference vs dapagliflozin<br>Week 52	1.62 (−0.37, 3.61)<br>P=0.0549	2.51 (0.50, 4.53)<br>P=0.0073	0.07 (−1.93, 2.07)<br>P=0.4712	—
**Urinary albumin-to-creatinine ratio**<br>**Week 32**	−24.25 (−34.01, −13.05)<br>n=167	−19.90 (−30.78, −7.31)<br>n=143	−27.48 (−37.43, −15.95)<br>n=138	0.63 (−11.89, 14.94)<br>n=183
Urinary albumin-to-creatinine ratio: Difference vs dapagliflozin<br>Week 32	−24.73 (−37.84, −8.85)<br>P=0.0018	−20.40 (−34.66, −3.04)<br>P=0.0117	−27.94 (−40.92, −12.10)<br>P=0.0006	—
Urinary albumin-to-creatinine ratio<br>Week 52	6.63 (−6.49, 21.61)<br>n=166	13.78 (−0.54, 30.15)<br>n=157	−5.20 (−17.06, 8.35)<br>n=160	17.44 (3.49, 33.27)<br>n=182
Urinary albumin-to-creatinine ratio: Difference vs dapagliflozin<br>Week 52	−9.20 (−24.33, 8.95)<br>P=0.1494	−3.12 (−19.44, 16.51)<br>P=0.3681	−19.28 (−32.84, −2.97)<br>P=0.0113	—

Values are model-based changes from baseline (95% CI) at weeks 32 and 52 in the intention-to-treat population under Supplementary estimand 2, the efficacy estimand. Lipid parameters and urinary albumin-to-creatinine ratio were analyzed after natural-log transformation and are presented as geometric mean percentage changes from baseline; systolic blood pressure was analyzed on the original scale and is presented as least-squares mean absolute change. Difference rows compare each safiglipron dose with dapagliflozin. The three prespecified intercurrent events were handled using a hypothetical strategy. n denotes participants with an observation at that visit.

CI=confidence interval. HDL-C=high-density lipoprotein cholesterol. ITT=intention-to-treat. LDL-C=low-density lipoprotein cholesterol. LS=least-squares. UACR=urinary albumin-to-creatinine ratio.

Supplementary Table 7 | Diabetes Treatment Satisfaction Questionnaire status scores through week 52

DTSQs domain	Visit	Safiglipron 30 mg (N=202)	Safiglipron 60 mg (N=203)	Safiglipron 90 mg (N=203)	Dapagliflozin 10 mg (N=202)
**Overall treatment satisfaction**	**Baseline**	29.85 (5.09) n=202	29.99 (4.97) n=202	29.71 (5.65) n=203	30.21 (5.12) n=202
	Change from baseline at week 32	2.02 (5.33) n=187	1.86 (5.35) n=182	2.16 (5.54) n=179	1.64 (5.01) n=193
	Change from baseline at week 52	2.58 (5.07) n=183	2.26 (5.05) n=179	2.72 (6.05) n=177	2.58 (5.42) n=191
**Perceived frequency of hyperglycaemia**	**Baseline**	2.61 (2.02) n=202	2.66 (1.94) n=202	2.37 (1.88) n=203	2.60 (1.99) n=202
	Change from baseline at week 32	−1.50 (2.45) n=187	−1.40 (2.16) n=182	−1.33 (2.19) n=179	−1.39 (2.10) n=193
	Change from baseline at week 52	−1.49 (2.47) n=183	−1.27 (2.29) n=179	−1.38 (2.10) n=177	−1.38 (2.01) n=191
**Perceived frequency of hypoglycaemia**	**Baseline**	0.72 (1.33) n=202	0.65 (1.37) n=202	0.68 (1.36) n=203	0.72 (1.38) n=202
	Change from baseline at week 32	−0.22 (1.66) n=187	−0.25 (1.47) n=182	−0.16 (1.57) n=179	−0.31 (1.52) n=193
	Change from baseline at week 52	−0.30 (1.66) n=183	−0.09 (1.52) n=179	−0.21 (1.63) n=177	−0.20 (1.81) n=191

Data are observed-case mean (SD) values in the intention-to-treat population; missing data were not imputed. n denotes participants with an observation at the displayed visit. Overall treatment satisfaction ranges from 0 to 36, with higher scores indicating greater satisfaction. Perceived frequencies of hyperglycaemia and hypoglycaemia range from 0 to 6, with higher scores indicating more frequent perceived episodes. No formal between-group comparisons were performed.

DTSQs=Diabetes Treatment Satisfaction Questionnaire status version. ITT=intention-to-treat. SD=standard deviation.

Supplementary Table 8 | Gastrointestinal-related treatment-emergent adverse events by maximum severity during the 52-week treatment period

Preferred term / maximum severity	Safiglipron 30 mg (N=202)	Safiglipron 60 mg (N=202)	Safiglipron 90 mg (N=203)	Dapagliflozin 10 mg (N=202)
**Any gastrointestinal-related TEAE**				
Mild	76 (37.6)	93 (46.0)	102 (50.2)	38 (18.8)
Moderate	22 (10.9)	33 (16.3)	43 (21.2)	9 (4.5)
Severe	0	0	1 (0.5)	0
Missing	0	0	0	0
Total	98 (48.5)	126 (62.4)	146 (71.9)	47 (23.3)
**Nausea**				
Mild	40 (19.8)	64 (31.7)	77 (37.9)	12 (5.9)
Moderate	6 (3.0)	13 (6.4)	18 (8.9)	2 (1.0)
Severe	0	0	0	0
Missing	0	0	0	0
Total	46 (22.8)	77 (38.1)	95 (46.8)	14 (6.9)
**Vomiting**				
Mild	23 (11.4)	44 (21.8)	45 (22.2)	4 (2.0)
Moderate	4 (2.0)	16 (7.9)	23 (11.3)	0
Severe	0	0	1 (0.5)	0
Missing	0	0	0	0
Total	27 (13.4)	60 (29.7)	69 (34.0)	4 (2.0)
**Diarrhea**				
Mild	19 (9.4)	32 (15.8)	33 (16.3)	10 (5.0)
Moderate	8 (4.0)	7 (3.5)	13 (6.4)	3 (1.5)
Severe	0	0	1 (0.5)	0
Missing	0	0	0	0
Total	27 (13.4)	39 (19.3)	47 (23.2)	13 (6.4)
**Constipation**				
Mild	8 (4.0)	8 (4.0)	10 (4.9)	5 (2.5)
Moderate	0	1 (0.5)	3 (1.5)	3 (1.5)
Severe	0	0	0	0
Missing	0	0	0	0
Total	8 (4.0)	9 (4.5)	13 (6.4)	8 (4.0)
**Abdominal distension**				
Mild	13 (6.4)	21 (10.4)	17 (8.4)	6 (3.0)
Moderate	3 (1.5)	7 (3.5)	8 (3.9)	1 (0.5)

Preferred term / maximum severity	Safiglipron 30 mg (N=202)	Safiglipron 60 mg (N=202)	Safiglipron 90 mg (N=203)	Dapagliflozin 10 mg (N=202)
Severe	0	0	0	0
Missing	0	0	0	0
Total	16 (7.9)	28 (13.9)	25 (12.3)	7 (3.5)
**Decreased appetite**				
Mild	39 (19.3)	42 (20.8)	57 (28.1)	16 (7.9)
Moderate	4 (2.0)	8 (4.0)	6 (3.0)	2 (1.0)
Severe	0	0	0	0
Missing	0	0	0	0
Total	43 (21.3)	50 (24.8)	63 (31.0)	18 (8.9)

Data are n (%) in the safety set. Percentages use the treatment-group N as denominator. Severity categories show each participant's maximum severity for the corresponding MedDRA preferred term during the 52-week treatment period; a participant with repeated occurrences of the same preferred term was counted once at the maximum reported severity and could contribute to more than one preferred term. MedDRA=Medical Dictionary for Regulatory Activities. N=number of participants in the safety set. PT=preferred term. TEAE=treatment-emergent adverse event.

Supplementary Table 9 | Change from baseline in the worst diabetic retinopathy stage during the 52-week treatment period

Category	Safiglipron 30 mg (N=202)	Safiglipron 60 mg (N=202)	Safiglipron 90 mg (N=203)	Dapagliflozin 10 mg (N=202)
Worsened	26 (12.9)	28 (13.9)	24 (11.8)	25 (12.4)
Unchanged	161 (79.7)	159 (78.7)	161 (79.3)	165 (81.7)
Improved	4 (2.0)	4 (2.0)	8 (3.9)	8 (4.0)
Indeterminate	1 (0.5)	0	0	0
Not evaluable	10 (5.0)	11 (5.4)	10 (4.9)	4 (2.0)

Data are n (%) in the safety set. The worst postbaseline diabetic retinopathy stage during the 52-week treatment period was compared with baseline using the worse stage in either eye. Participants without an evaluable baseline-to-postbaseline comparison are shown as not evaluable. Missing data were not imputed and no formal between-group comparisons were performed.

Supplementary Table 10 | Serious treatment-emergent adverse events during the 52-week treatment period

Category	Safiglipron 30 mg (N=202)	Safiglipron 60 mg (N=202)	Safiglipron 90 mg (N=203)	Dapagliflozin 10 mg (N=202)
Any serious TEAE	21 (10.4)	12 (5.9)	10 (4.9)	18 (8.9)
Death	0	0	1 (0.5)	0
Any study treatment-related serious TEAE	0	2 (1.0)	4 (2.0)	1 (0.5)
Study treatment-related death	0	0	0	0
Cerebral infarction	3 (1.5)	0	0	3 (1.5)
Dizziness	0	0	0	2 (1.0)
Bell's palsy	0	0	1 (0.5)	0
Vertebrobasilar insufficiency	0	0	1 (0.5)	0
Facial paralysis	0	0	0	1 (0.5)
Thalamic infarction	1 (0.5)	0	0	0
Arteriosclerosis coronary artery	0	3 (1.5)	1 (0.5)	1 (0.5)
Acute myocardial infarction	0	0	1 (0.5)	2 (1.0)
Angina pectoris	0	0	1 (0.5)	1 (0.5)
Angina unstable	0	1 (0.5)	0	0
Acute left ventricular failure	1 (0.5)	0	0	0
Arrhythmia	1 (0.5)	0	0	0
Pneumonia	2 (1.0)	0	0	0
Gastroenteritis	0	1 (0.5)	0	1 (0.5)
Herpes zoster	0	1 (0.5)	0	0
Febrile infection	1 (0.5)	0	0	0
Epididymitis	1 (0.5)	0	0	0
Appendicitis	0	1 (0.5)	0	0
Chronic tonsillitis	0	0	0	1 (0.5)
Schistosomiasis	0	0	0	1 (0.5)
Abdominal distension	0	0	2 (1.0)	0
Constipation	0	0	1 (0.5)	0
Abdominal pain	0	0	0	1 (0.5)
Anal fistula	0	0	0	1 (0.5)
Pancreatitis acute	0	1 (0.5)	0	0
Vomiting	0	0	1 (0.5)	0
Upper gastrointestinal haemorrhage	1 (0.5)	0	0	0
Duodenal ulcer	0	0	0	1 (0.5)
Oesophageal varices haemorrhage	0	1 (0.5)	0	0

Category	Safiglipron 30 mg (N=202)	Safiglipron 60 mg (N=202)	Safiglipron 90 mg (N=203)	Dapagliflozin 10 mg (N=202)
Gastric ulcer	1 (0.5)	0	0	0
Gastric polyps	0	0	0	1 (0.5)
Acute cholecystitis	2 (1.0)	0	0	1 (0.5)
Cholelithiasis	0	1 (0.5)	0	1 (0.5)
Bile duct stone	0	0	1 (0.5)	0
Acute cholangitis	0	0	1 (0.5)	0
Intervertebral disc protrusion	0	0	1 (0.5)	0
Osteoarthritis	1 (0.5)	0	0	0
Arthropathy	0	0	0	1 (0.5)
Spinal osteoarthritis	0	0	0	1 (0.5)
Tenosynovitis	1 (0.5)	0	0	0
Cervical spinal stenosis	0	0	1 (0.5)	0
Rib fracture	1 (0.5)	0	0	1 (0.5)
Craniofacial fracture	0	1 (0.5)	0	1 (0.5)
Jaw fracture	0	0	0	1 (0.5)
Skull base fracture	0	1 (0.5)	0	0
Skull fracture	0	1 (0.5)	0	0
Craniocerebral injury	0	1 (0.5)	0	0
Cerebral concussion	0	0	0	1 (0.5)
Thermal burn	1 (0.5)	0	0	0
Traumatic subarachnoid haemorrhage	0	1 (0.5)	0	0
Foot fracture	0	0	0	1 (0.5)
Macular oedema	1 (0.5)	0	1 (0.5)	0
Cataract	0	0	1 (0.5)	0
Vitreous haemorrhage	0	0	1 (0.5)	0
Glaucoma	1 (0.5)	0	0	0
Diabetic retinopathy	0	0	1 (0.5)	0
Type 2 diabetes mellitus	1 (0.5)	0	0	0
Sudden hearing loss	0	0	0	1 (0.5)
Adenolymphoma	1 (0.5)	0	0	0
Anaphylactic shock	0	0	1 (0.5)	0
Dermatitis allergic	0	0	1 (0.5)	0
Biochemical pregnancy	0	1 (0.5)	0	0
Acute kidney injury	1 (0.5)	0	0	0

Category	Safiglipron 30 mg (N=202)	Safiglipron 60 mg (N=202)	Safiglipron 90 mg (N=203)	Dapagliflozin 10 mg (N=202)
Scalp haematoma	0	1 (0.5)	0	0

Data are n (%) in the safety set. Serious treatment-emergent adverse events occurred or worsened after the first dose and during the 52-week treatment period. Aggregate rows are followed by events listed by MedDRA version 28.0 preferred term. A participant was counted once within each preferred term and could contribute to more than one preferred term.

MedDRA=Medical Dictionary for Regulatory Activities. N=number of participants in the safety set. PT=preferred term. TEAE=treatment-emergent adverse event.

Supplementary Table 11 | Treatment-emergent adverse events leading to study treatment discontinuation during the 52-week treatment period

Preferred term	Safiglipron 30 mg (N=202)	Safiglipron 60 mg (N=202)	Safiglipron 90 mg (N=203)	Dapagliflozin 10 mg (N=202)
Any TEAE leading to study treatment discontinuation	8 (4.0)	8 (4.0)	8 (3.9)	3 (1.5)
Vomiting	2 (1.0)	4 (2.0)	5 (2.5)	0
Nausea	1 (0.5)	5 (2.5)	4 (2.0)	0
Flatulence	1 (0.5)	1 (0.5)	0	0
Abdominal distension	1 (0.5)	0	1 (0.5)	0
Abdominal discomfort	0	0	1 (0.5)	0
Diarrhea	1 (0.5)	0	0	0
Pancreatitis acute	0	1 (0.5)	0	0
Upper abdominal pain	0	1 (0.5)	0	0
Duodenogastric reflux	1 (0.5)	0	0	0
Gastric ulcer	1 (0.5)	0	0	0
Gastroesophageal reflux disease	0	0	1 (0.5)	0
Dyspepsia	0	1 (0.5)	0	0
Decreased appetite	2 (1.0)	2 (1.0)	1 (0.5)	0
Hyperlipidaemia	0	0	0	1 (0.5)
Cholecystitis	0	1 (0.5)	0	0
Cholecystitis acute	1 (0.5)	0	0	0
Asthenia	1 (0.5)	0	0	1 (0.5)
Vaginal infection	0	0	0	1 (0.5)
Blood pressure decreased	0	0	1 (0.5)	0

Data are n (%) in the safety set. Events occurred or worsened after the first dose, led to permanent discontinuation of study treatment and are listed by MedDRA version 28.0 preferred term. A participant was counted once within each preferred term and could contribute to more than one preferred term.

MedDRA=Medical Dictionary for Regulatory Activities. N=number of participants in the safety set. PT=preferred term. TEAE=treatment-emergent adverse event.

Supplementary Table 12 | Selected hepatic, pancreatic and thyroid laboratory parameters through week 52

Parameter	Timepoint and statistic	Safiglipron 30 mg (N=202)	Safiglipron 60 mg (N=202)	Safiglipron 90 mg (N=203)	Dapagliflozin 10 mg (N=202)
**Alanine aminotransferase (U/L)**	**Baseline, mean (SD)**	27.96 (15.57) n=202	30.37 (18.20) n=202	28.46 (15.91) n=203	30.99 (19.03) n=202
	Week 32, geometric mean ratio to baseline (geometric SD)	0.98 (1.65) n=187	0.88 (1.70) n=182	0.88 (1.69) n=179	0.91 (1.60) n=193
	Week 32, geometric mean percentage change from baseline	−2.24 n=187	−12.47 n=182	−12.40 n=179	−9.19 n=193
	Week 52, geometric mean ratio to baseline (geometric SD)	0.92 (1.66) n=183	0.86 (1.79) n=179	0.83 (1.69) n=176	0.83 (1.53) n=191
	Week 52, geometric mean percentage change from baseline	−7.61 n=183	−13.74 n=179	−16.85 n=176	−17.35 n=191
**Aspartate aminotransferase (U/L)**	**Baseline, mean (SD)**	22.72 (10.29) n=202	23.50 (10.88) n=202	23.88 (10.36) n=203	23.84 (10.51) n=202
	Week 32, geometric mean ratio to baseline (geometric SD)	1.03 (1.45) n=187	0.96 (1.46) n=182	0.93 (1.43) n=179	0.96 (1.42) n=193
	Week 32, geometric mean percentage change from baseline	2.51 n=187	−4.38 n=182	−6.70 n=179	−3.54 n=193
	Week 52, geometric mean ratio to baseline (geometric SD)	0.99 (1.43) n=182	0.98 (1.47) n=179	0.92 (1.45) n=176	0.91 (1.36) n=191
	Week 52, geometric mean percentage change from baseline	−1.19 n=182	−2.24 n=179	−8.29 n=176	−8.51 n=191
**Amylase (U/L)**	**Baseline, mean (SD)**	57.71 (24.16) n=202	57.17 (18.69) n=202	55.42 (18.27) n=203	57.66 (22.44) n=202
	Week 32, geometric mean ratio to baseline (geometric SD)	1.12 (1.32) n=187	1.14 (1.30) n=182	1.19 (1.30) n=179	1.06 (1.23) n=193
	Week 32, geometric mean percentage change from baseline	12.46 n=187	13.63 n=182	18.97 n=179	6.17 n=193

Parameter	Timepoint and statistic	Safiglipron 30 mg (N=202)	Safiglipron 60 mg (N=202)	Safiglipron 90 mg (N=203)	Dapagliflozin 10 mg (N=202)
	Week 52, geometric mean ratio to baseline (geometric SD)	1.15 (1.32) n=183	1.13 (1.28) n=179	1.15 (1.29) n=176	1.07 (1.27) n=191
	Week 52, geometric mean percentage change from baseline	15.07 n=183	13.42 n=179	14.85 n=176	6.87 n=191
**Lipase (U/L)**	**Baseline, mean (SD)**	53.33 (48.25) n=202	53.64 (57.04) n=202	45.67 (39.95) n=203	59.82 (60.46) n=202
	Week 32, geometric mean ratio to baseline (geometric SD)	1.20 (1.60) n=187	1.19 (1.66) n=182	1.28 (1.57) n=179	0.99 (1.48) n=193
	Week 32, geometric mean percentage change from baseline	19.94 n=187	19.37 n=182	27.82 n=179	−1.37 n=193
	Week 52, geometric mean ratio to baseline (geometric SD)	1.23 (1.58) n=183	1.24 (1.50) n=179	1.24 (1.43) n=176	1.01 (1.50) n=191
	Week 52, geometric mean percentage change from baseline	23.22 n=183	24.31 n=179	23.98 n=176	1.48 n=191
**Calcitonin (ng/L)**	**Baseline, mean (SD)**	2.75 (3.44) n=202	2.73 (3.27) n=202	2.41 (2.94) n=203	2.95 (3.42) n=201
	Week 32, geometric mean ratio to baseline (geometric SD)	0.98 (2.08) n=186	0.99 (1.92) n=181	1.05 (1.84) n=178	0.99 (1.64) n=191
	Week 32, geometric mean percentage change from baseline	−2.47 n=186	−0.53 n=181	5.32 n=178	−1.34 n=191
	Week 52, geometric mean ratio to baseline (geometric SD)	1.00 (1.99) n=183	0.97 (1.90) n=179	1.01 (2.17) n=177	0.91 (1.93) n=190
	Week 52, geometric mean percentage change from baseline	−0.02 n=183	−2.58 n=179	1.42 n=177	−8.67 n=190

Data are observed-case descriptive summaries in the safety set; missing data were not imputed. Baseline is the last non-missing assessment before the first dose. n denotes participants with an available result for the displayed parameter and timepoint. Percentage change in geometric mean from baseline was calculated as (geometric mean ratio to baseline − 1) × 100.

ALT=alanine aminotransferase. AST=aspartate aminotransferase. SD=standard deviation.

Supplementary Table 13 | Vital signs through week 52

Vital-sign parameter	Timepoint and statistic	Safiglipron 30 mg (N=202)	Safiglipron 60 mg (N=202)	Safiglipron 90 mg (N=203)	Dapagliflozin 10 mg (N=202)
**Systolic blood pressure (mm Hg)**	**Baseline, mean (SD)**	124.57 (11.91) n=202	125.25 (12.28) n=202	125.48 (12.02) n=203	123.12 (11.62) n=202
	Week 32 change from baseline, mean (SD)	−2.64 (10.47) n=187	−3.47 (10.86) n=182	−4.76 (12.27) n=179	−3.98 (11.27) n=193
	Week 52 change from baseline, mean (SD)	−2.36 (12.15) n=183	−2.24 (10.14) n=179	−3.98 (11.42) n=177	−3.01 (10.93) n=191
**Diastolic blood pressure (mm Hg)**	**Baseline, mean (SD)**	81.10 (7.07) n=202	81.46 (7.36) n=202	81.68 (8.32) n=203	81.18 (7.62) n=202
	Week 32 change from baseline, mean (SD)	−0.72 (7.99) n=187	−1.08 (8.15) n=182	−0.86 (8.03) n=179	−2.22 (7.50) n=193
	Week 52 change from baseline, mean (SD)	−1.79 (8.05) n=183	−1.11 (7.87) n=179	−1.15 (7.21) n=177	−2.19 (7.57) n=191
**Pulse rate (beats/min)**	**Baseline, mean (SD)**	79.83 (10.35) n=202	80.05 (10.05) n=202	80.46 (9.38) n=203	79.31 (9.15) n=202
	Week 32 change from baseline, mean (SD)	1.26 (8.43) n=187	3.69 (9.26) n=182	2.50 (9.64) n=179	−0.72 (8.27) n=193
	Week 52 change from baseline, mean (SD)	2.41 (8.87) n=183	2.38 (9.70) n=179	2.42 (9.15) n=177	0.63 (8.50) n=191

Data are observed-case mean (SD) values in the safety set; missing data were not imputed. Baseline is the last non-missing assessment before the first dose, and n denotes participants with an available result at the displayed timepoint.

BP=blood pressure. SD=standard deviation.

Supplementary Table 14 | Scheduled 12-lead electrocardiographic parameters through week 52

ECG parameter	Timepoint and statistic	Safiglipron 30 mg (N=202)	Safiglipron 60 mg (N=202)	Safiglipron 90 mg (N=203)	Dapagliflozin 10 mg (N=202)
**Heart rate (beats/min)**	**Baseline, mean (SD)**	78.6 (11.32) n=202	78.8 (10.88) n=202	78.0 (9.38) n=203	77.6 (10.83) n=202
	Week 32 change from baseline, mean (SD)	1.4 (9.65) n=187	3.4 (9.26) n=181	3.9 (10.93) n=178	−0.7 (8.77) n=193
	Week 52 change from baseline, mean (SD)	2.6 (9.76) n=183	3.0 (9.87) n=178	4.0 (9.75) n=176	0.3 (8.77) n=191
**PR interval (ms)**	**Baseline, mean (SD)**	159.1 (19.75) n=202	157.0 (19.71) n=202	158.4 (22.14) n=203	157.4 (20.23) n=202
	Week 32 change from baseline, mean (SD)	−0.5 (11.84) n=186	1.7 (13.15) n=181	2.3 (12.25) n=178	1.0 (11.87) n=193
	Week 52 change from baseline, mean (SD)	0.1 (12.45) n=182	1.2 (12.66) n=178	1.7 (11.15) n=176	−1.0 (12.05) n=191
**QT interval (ms)**	**Baseline, mean (SD)**	375.4 (26.77) n=202	374.2 (27.41) n=202	377.0 (24.13) n=203	375.4 (27.38) n=202
	Week 32 change from baseline, mean (SD)	−2.4 (26.00) n=187	−4.3 (24.74) n=181	−5.2 (25.00) n=178	3.6 (22.23) n=193
	Week 52 change from baseline, mean (SD)	−5.9 (27.67) n=183	−8.7 (26.53) n=178	−5.2 (26.52) n=176	3.6 (24.87) n=191
**QTcF interval (ms)**	**Baseline, mean (SD)**	408.39 (22.01) n=202	407.48 (21.07) n=202	409.92 (21.55) n=203	406.78 (22.04) n=202
	Week 32 change from baseline, mean (SD)	−0.05 (24.82) n=187	1.08 (22.70) n=181	0.51 (22.48) n=178	2.52 (19.29) n=193
	Week 52 change from baseline, mean (SD)	−2.19 (26.25) n=183	−4.45 (23.66) n=178	0.68 (26.02) n=176	4.41 (24.65) n=191
**RR interval (ms)**	**Baseline, mean (SD)**	780.2 (120.44) n=202	777.0 (111.74) n=202	780.5 (96.25) n=203	789.0 (113.55) n=201
	Week 32 change from baseline, mean (SD)	−14.4 (106.07) n=187	−31.3 (87.57) n=181	−35.1 (101.54) n=178	6.7 (90.24) n=192
	Week 52 change from baseline, mean (SD)	−26.7 (101.95) n=183	−33.4 (94.56) n=178	−34.9 (91.43) n=176	−2.5 (90.47) n=190

Data are observed-case mean (SD) values from scheduled 12-lead electrocardiograms in the safety set; missing data were not imputed. Baseline is the last non-missing assessment before the first dose, and n denotes participants with an available result at the displayed timepoint.

ECG=electrocardiogram. PR=PR interval. QT=QT interval. QTcF=QT interval corrected for heart rate using Fridericia's formula. RR=RR interval. SD=standard deviation.

Supplementary Table 15 | Post hoc site-adjusted comparisons of treatment-related TEAEs and TEAEs leading to permanent treatment discontinuation during the 52-week treatment period

**Outcome**	**Safiglipron dose**	**Unadjusted OR (95% CI)**	**P value**	**Site-adjusted OR (95% CI)**	**P value**
**Treatment-related TEAEs**	30 mg	2.26 (1.49–3.45)	0.0001	2.79 (1.75–4.46)	<0.0001
**Treatment-related TEAEs**	60 mg	5.21 (3.23–8.64)	<0.0001	6.35 (3.71–10.85)	<0.0001
**Treatment-related TEAEs**	90 mg	4.82 (3.01–7.90)	<0.0001	5.59 (3.32–9.42)	<0.0001
**TEAEs leading to permanent treatment discontinuation**	30 mg	2.74 (0.78–12.63)	0.1414	2.93 (0.73–11.79)	0.1299
**TEAEs leading to permanent treatment discontinuation**	60 mg	2.74 (0.78–12.63)	0.1414	2.97 (0.75–11.85)	0.1222
**TEAEs leading to permanent treatment discontinuation**	90 mg	2.72 (0.77–12.56)	0.1434	2.83 (0.71–11.37)	0.1415

All comparisons are versus dapagliflozin 10 mg. These post hoc analyses were conducted in the safety population (N=809; n=202, 202, 203 and 202 for safiglipron 30 mg, 60 mg, 90 mg and dapagliflozin, respectively). Each outcome was analyzed as a participant-level binary indicator of at least one qualifying event during the 52-week treatment period. Separate models were fitted for each outcome. Unadjusted logistic regression models included treatment group only; site-adjusted logistic mixed-effects models included treatment group as a fixed effect and study site as a random intercept. ORs greater than 1 indicate higher odds of the outcome with safiglipron than with dapagliflozin. The site random-intercept variance and ICC were 0.629 and 0.161, respectively, for treatment-related TEAEs, and 1.749 and 0.347, respectively, for TEAEs leading to permanent treatment discontinuation.

CI=confidence interval. ICC=intraclass correlation coefficient. OR=odds ratio. TEAE=treatment-emergent adverse event.

Supplementary References

1. International Council for Harmonisation. Addendum on Estimands and Sensitivity Analysis in Clinical Trials to the Guideline on Statistical Principles for Clinical Trials E9(R1) (2019).

2. Rubin, D. B. Multiple Imputation for Nonresponse in Surveys (Wiley, 1987). https://doi.org/10.1002/9780470316696

A RANDOMIZED, DOUBLE-BLIND, PHASE III STUDY TO EVALUATE THE EFFICACY AND SAFETY OF HRS-7535 COMPARED WITH DAPAGLIFLOZIN IN ADULT PARTICIPANTS WITH TYPE 2 DIABETES INADEQUATELY CONTROLLED ON METFORMIN THERAPY

Protocol No.: HRS-7535-302
Study Phase: Phase III
Compound No.: HRS-7535
Protocol Version No. and Date: 1.3, 24 Mar., 2025

Sponsor: Shandong Shengdi Pharmaceutical Co., Ltd.
Biomedical Park, 1777 Dazheng Road, Innovation Zone, Ji'nan, Shandong
Building 5, Room 102, Industrialization Base for Small and Medium Enterprises

Confidentiality Statement

The information contained in this protocol is confidential and is for use by clinical investigators only, and shall not be disclosed unless required by current laws or regulations. Its copyright is owned by Jiangsu Hengrui Pharmaceuticals Co., Ltd. or its subsidiaries, and it shall not be duplicated or distributed to any person who has not participated in this clinical trial, except for those who have signed a confidentiality agreement with Jiangsu Hengrui Pharmaceuticals Co., Ltd. or its subsidiaries.

PROTOCOL AMENDMENT HISTORY AND CURRENT VERSION AMENDMENT

This study protocol is version 1.3.

Protocol Amendment History

Version No.	Version Date	Enrollment rate estimates
1.3	24 Mar., 2025	50%
1.2	26 Sep., 2024	Not applicable
1.1	17 Jul., 2024	Not applicable
1.0	04 Jun., 2024	Not applicable

Overview of Protocol Amendments in Current Version

**Version No.**	**1.3**	
**Enrollment rate estimates**	50%	
**Reason for Revision**	Main reason for amendment:<br>☐ Revision requested by the drug regulatory authority<br>☐New regulatory guidance requirements<br>☐Institutional Review Board Feedback<br>☐New safety data<br>☐Manufacturing Changes<br>☐New Investigational Product (in case of adaptive trial)<br>☐Change in strategy<br>☐Changes in Standard of Care<br>☐New non-safety data<br>☐Investigator/Site Feedback<br>☐Difficulty in recruiting<br>☐Protocol inconsistencies and/or errors<br>☐Protocol design error<br>☒Others	Other reason for amendment:<br>☐ Revision requested by the drug regulatory authority<br>☐New regulatory guidance requirements<br>☐Institutional Review Board Feedback<br>☐New safety data<br>☐Manufacturing Changes<br>☐New Investigational Product (in case of adaptive trial)<br>☐Change in strategy<br>☐Changes in Standard of Care<br>☐New non-safety data<br>☐Investigator/Site Feedback<br>☐Difficulty in recruiting<br>☒Protocol inconsistencies and/or errors<br>☐Protocol design error<br>☒Others
**Summary of Revisions**	Added dapagliflozin PK testing, updated the operational procedures of the protocol, and revised the inconsistencies in the protocol.	
**is the current revision likely to have a significant impact on any of the following:**<br>• Safety of clinical trial participants;<br>• The scientific nature of the clinical trial;<br>• The reliability of the generation of clinical trial data.	☐Yes No☒	

Summary of Changes to the Current Version of the Protocol

Section Number and Name	Description of Change	Brief Rationale for Changes

Table 1.3 Study flowchart footnote<br>3.1. Objectives and Endpoints<br>8.6. Pharmacokinetics<br>9.6.1. Pharmacokinetic Analysis	• Add dapagliflozin PK testing. And add corresponding exploratory endpoint and pharmacokinetics analysis.	• Add dapagliflozin PK testing, and add corresponding exploratory endpoints and pharmacokinetic analysis.
Table 1.3 Study flowchart footnote	• Added descriptions that participants may not be under fasting conditions during V2 and V4 site visits.	• This is an erratum, V2 and V4 Visits do not involve operations requiring under fasting conditions.
6.8.3. Permitted Concomitant Treatment	• Optimize the description of metformin stable dosage: do not change the planned metformin dosage.	• Optimize the definition description of stable treatment with background medication metformin.
8.3.2. Laboratory Determinations	• The sample type labels were removed for laboratory samples involving Safety Lab Tests.<br>• Delete urine glucose in Urinalysis.	• The laboratory test items including chemistry, blood lipid, amylase, lipase, and calcitonin are intended for safety evaluation. The assessment of "serum" samples could cover the use of the vast majority of current testing methods and equipment, but the protocol overlooks some equipment that uses "plasma" samples for testing. Considering that the involved indicators are all safety lab tests, the type of sample used for testing does not affect the safety evaluation, hence the annotation on the blood sample type for such items is removed.<br>• This is a correction, urine glucose is not a mandatory test item.

Information on previous protocol amendments is summarized in Section 13.4.13.4

TABLE OF CONTENTS

Table of Contents 3
LIST OF TABLES 6
List of Figures 6
1. Protocol summary 7
1.1. Protocol synopsis 7
1.2. Schema 21
1.3. Schedule of activities 22
2. Introduction 29
2.1. Study background 29
2.1.1. Disease background 29
2.1.2. HRS-7535 30
2.1.3. Non-clinical experience 30
2.1.4. Clinical experience 32
2.2. Summary of benefits and risks 34
2.2.1. Benefit summary 34
2.2.2. Risk summary and management measures 35
2.2.3. Overall benefit-risk conclusion 35
3. Study objectives, endpoints, and estimands 36
3.1. Objectives and endpoints 36
3.2. Estimands 38
3.2.1. Primary estimand 38
3.2.2. Supplementary estimand 38
3.2.3. Secondary estimand 39
4. Study design 39
4.1. Study design description 39
4.2. Trial design basis 40
4.3. Access to study intervention after end of study 41
4.4. Definition of trial initiation and completion 41
5. Study population 41
5.1. Selection of trial population 41
5.2. Selection of trial population and basis 41
5.3. Inclusion criteria 42
5.4. Exclusion criteria 42
5.5. Lifestyle requirements 46
5.6. Screen failure and randomization failure 46
6. Trial interventions and concomitant treatment 47
6.1. Information of investigational intervention 47
6.2. Basis for selection of trial intervention 47
6.2.1. Rationale for dose selection 48
6.3. Dosage and administration 48

6.3.1. Dosing regimen ... 48
6.3.2. Medication guidance ... 50
6.3.3. Dose modification ... 50
6.3.3.1. Management of gastrointestinal symptoms ... 51
6.3.3.2. Temporary dose interruption ... 52
6.4. Treatment of overdose ... 53
6.5. Medicinal product preparation, handling, storage and accountability ... 53
6.5.1. Preparation of medicinal product ... 53
6.5.2. Medicinal product handling and storage ... 53
6.5.3. Management of medicinal products ... 54
6.5.3.1. Medicinal product destruction ... 54
6.6. Participant assignment, randomization and blinding ... 55
6.6.1. Participant assignment ... 55
6.6.2. Randomization ... 55
6.6.2.1. Randomization criteria ... 56
6.6.3. Blinding and unblinding ... 56
6.7. Compliance with trial intervention ... 57
6.8. Concomitant treatment ... 58
6.8.1. Medications for gastrointestinal symptoms ... 58
6.8.2. Prohibited concomitant treatment ... 58
6.8.3. Permitted concomitant treatment ... 59
6.8.4. Rescue treatment ... 59
7. Termination of trial intervention for participant or withdrawal from trial ... 60
7.1. Termination of trial intervention for participant ... 60
7.1.1. Discontinuation of trial intervention ... 60
7.2. Withdrawal of participant from the trial ... 62
7.3. Lost to follow-up ... 62
7.4. Specific discontinuation criteria (not applicable) ... 63
8. Test evaluation and procedure ... 63
8.1. Evaluation of screening period and Run-in period ... 63
8.2. Assessment of efficacy and procedures ... 64
8.2.1. Glycated hemoglobin A1c (HbA1c) ... 64
8.2.2. Fasting plasma glucose (FPG) ... 64
8.2.3. Self-measured blood glucose (SMBG) ... 64
8.2.4. Mixed meal test ... 65
8.2.5. Body weight and waist circumference ... 65
8.3. Safety evaluation and procedures ... 66
8.3.1. Vital signs and physical examination ... 67
8.3.2. Laboratory tests ... 67
8.3.3. 12-lead ECG ... 68
8.3.4. Screening and assessment of diabetic retinopathy ... 68

8.4. Adverse event (AE) and serious adverse event (SAE) 69
8.4.1. Definition of AEs and SAEs 69
8.4.2. Collection of AEs and SAEs 70
8.4.3. Identification of AEs and SAEs 71
8.4.4. Recording of AEs and SAEs 71
8.4.5. Follow-up of AEs/SAEs 71
8.4.6. SAE reporting requirements 71
8.4.7. SAE reporting requirements 72
8.4.8. Suspected unexpected serious adverse reaction report 72
8.4.9. Adverse events of special interest (AESI) 72
8.4.9.1. Abnormal liver function tests 73
8.4.9.2. Hypoglycemic episodes 74
8.5. Pregnancy 74
8.6. Pharmacokinetics 75
8.7. Genetics (not applicable) 76
8.8. Biomarkers (not applicable) 76
8.9. Immunogenicity (not applicable) 76
8.10. Healthcare resource utilization and health economics (not applicable) 76
9. Statistical considerations 76
9.1. Analysis population 76
9.2. Analysis supporting the primary objective 76
9.2.1. Statistical hypothesis and decision rules 76
9.2.2. Primary estimand analysis 78
9.2.3. Supplemental estimand analysis 78
9.3. Analysis supporting secondary objectives 78
9.3.1. Secondary estimand analysis 78
9.4. Analyses to support exploratory objectives 79
9.5. Safety analysis 79
9.6. Other analyses 79
9.6.1. Pharmacokinetic analysis 79
9.6.2. Subgroup analysis 79
9.7. Interim analysis (not applicable) 80
9.8. Sample size Estimation 80
9.9. Protocol deviations 80
10. General considerations: regulatory, ethical, and study oversight 80
10.1. Regulatory and ethical considerations 80
10.2. Committee (not applicable) 81
10.3. Informed consent process 81
10.4. Data protection 82
10.4.1. Confidentiality of participant information 82
10.4.2. Data protection 82
10.5. Early termination or discontinuation of trial 83
11. General considerations: risk management and quality assurance 83

11.1. Data quality assurance 83
11.2. Source data 84

12. Appendix: adverse events and serious adverse events - definitions, severity, and causality 86

12.1. Definition and supplement of AE 86

12.1.1. Drug overdose 86

12.2. Description and supplement of SAE definition 86

12.2.1. Hospitalization 86

12.3. Severity 87
12.4. Causality 87

13. Appendix: definitions and supporting operational details 88

13.1. Contraception and pregnancy test 88

13.1.1. Definitions related to fertility 88
13.1.2. Contraception 88
13.1.3. Pregnancy test and FSH test 89

13.2. Laboratory tests 90
13.3. Country-specific variations (not applicable) 90
13.4. Protocol amendment history 90
13.5. CKD-EPI formula 92

14. Appendix: abbreviations 93
15. Appendix: references 95

LIST OF TABLES

Table 1. Study Flowchart 22

Table 2. Study Objectives and Endpoints 36

Table 3. Definition of Primary Estimand and Handling Strategies 38

Table 4. Investigational Product Information 47

Table 5. Drug Dosage and Corresponding Number of Tablets During the Trial 49

Table 6. Rescue Treatment Criteria for Fasting Blood Glucose Levels 60

Table 7. Laboratory Test Items 67

Table 8. Collection of Clinical Trial Safety Events 71

Table 9. Criteria for Determining Abnormal Liver Function Tests 73

Table 10. 2024 ADA-Recommended Classification of Hypoglycemic Episodes 74

LIST OF FIGURES

Figure 1. Overview of study design 21

1. Protocol summary

1.1. Protocol synopsis

**PROTOCOL TITLE**	A randomized, double-blind, Phase III study to evaluate the efficacy and safety of HRS-7535 compared with dapagliflozin in adult participants with type 2 diabetes inadequately controlled on metformin therapy.
**Protocol No.:**	HRS-7535-302
**Version No. and Date**	1.3, Mar. 24, 2025
**Study phase: phase**	Phase III
**Compound number**	HRS-7535
**Sponsor**	Shandong Shengdi Pharmaceutical Co., Ltd.
**Participating Site(s)**	Beijing Hospital and other hospitals
**Objectives**	**Primary Objective**<br>To confirm the non-inferiority of HRS-7535 to dapagliflozin in glycemic control efficacy among adult participants with type 2 diabetes who had inadequate glycemic control after metformin treatment.<br>**Secondary Objective**<br>In adult participants with type 2 diabetes who have poor glycemic control after metformin treatment:<br>• Compare the efficacy of HRS-7535 and dapagliflozin after 32 weeks of treatment;<br>• To evaluate the safety and tolerability of HRS-7535 and dapagliflozin after 32 weeks of treatment;<br>• Compare the efficacy of HRS-7535 and dapagliflozin after 52 weeks of treatment;

	• To evaluate the safety and tolerability of HRS-7535 and dapagliflozin after 52 weeks of treatment;<br>• To evaluate the pharmacokinetics (PK) of HRS-7535;<br>• To assess patient reported outcomes in each treatment group.
**Endpoints and Estimands**	**Primary Endpoint**<br>• The change from baseline in Glycated Hemoglobin A1C (HbA1c) at Week 32 of treatment with HRS-7535 compared to dapagliflozin.<br>**Primary Objective**<br>• Treatment: HRS-7535 30 mg, 60 mg, 90 mg or dapagliflozin 10 mg, orally administered, once daily (QD);<br>• Population: adult participants with type 2 diabetes inadequately controlled on metformin therapy;<br>• Variable (endpoint): change from baseline in HbA1c at Week 32;<br>• Intercurrent events and handling strategies:<br><br>(see table below)<br><br>• Summary at the population level: the mean difference between groups in the change from baseline of HbA1c after 32 weeks of treatment with HRS-7535 and dapagliflozin.<br>**Secondary endpoints:**<br>HRS-7535 versus dapagliflozin at Week 32 of treatment:<br>• Proportion of participants achieving HbA1c < 7.0%<br>• Proportion of participants achieving HbA1c ≤6.5%<br>• Change from baseline in fasting plasma glucose (FPG);<br>• Percentage change in weight from baseline.<br>**Secondary endpoints:**

Concomitant Event	Management Strategy
Intercurrent event 1: permanent discontinuation of study treatment	Hypothetical strategy: subsequently collected data will not be included in the statistical analysis
Intercurrent event 2: initiation of additional antihyperglycemic medications*	Hypothetical strategy: the subsequently collected data will not be included in the statistical analysis.

* Additional antihyperglycemic medications were defined as any antihyperglycemic medications used cumulatively for more than 14 days.

	Efficacy endpoint<br>HRS-7535 versus dapagliflozin at Week 32 of treatment:<br>• Change from baseline in weight and waist circumference from baseline;<br>• Proportion of participants achieving HbA1c < 5.7%;<br>• Changes from baseline in fasting serum insulin, fasting serum C-peptide, homeostasis model assessment of β-cell function (HOMA-β), and insulin resistance index (HOMA-IR);<br>• Changes from baseline in 2-hour postprandial plasma glucose, 2-hour postprandial serum insulin, and 2-hour postprandial serum C-peptide;<br>• Proportion of participants requiring rescue treatment in each group;<br>• Changes from baseline in 7-point self-monitored blood glucose (SMBG) profile.<br>HRS-7535 versus dapagliflozin at Week 52 of treatment:<br>• Change from baseline in HbA1c;<br>• Change from baseline in FPG;<br>• Percentage change from baseline in weight;<br>• Change from baseline in weight and waist circumference;<br>• Proportion of participants achieving HbA1c < 7.0%;<br>• Proportion of participants achieving HbA1c ≤6.5%;<br>• Proportion of participants achieving HbA1c < 5.7%;<br>• Changes from baseline in fasting serum insulin, fasting serum C-peptide, HOMA-β and HOMA-IR;<br>• Changes from baseline in 2-hour postprandial plasma glucose, 2-hour postprandial serum insulin, and 2-hour postprandial serum C-peptide;<br>• Proportion of participants requiring rescue treatment in each group<br>• Changes from baseline in 7-point SMBG profile.<br>Safety endpoints<br>HRS-7535 versus dapagliflozin at Week 32 and Week 52 of treatment:

	• Adverse events, hypoglycemic events, laboratory tests, vital signs, physical examination, 12-lead electrocardiogram, diabetic retinopathy screening and assessment.<br>Pharmacokinetic Endpoints<br>• Plasma HRS-7535 concentration.<br>Patient-Reported Outcome endpoint.<br>• Change from baseline to Week 32 and Week 52 in Diabetes Treatment Satisfaction Questionnaire status version (DTSQs).
**Study Population**	Adult participants with type 2 diabetes who have poor glycemic control despite metformin treatment on the basis of regular diet and exercise control.
**Study Design**	This trial is a multicenter, randomized, double-blind, positive-controlled, parallel-designed Phase III clinical trial, including a screening period of up to 2 weeks, a 3-week Run-in period, a 52-week double-blind treatment period, and a 2-week safety follow-up period. The trial population consists of adult participants with type 2 diabetes who have inadequate glycemic control after metformin treatment on the basis of regular diet and exercise, with a planned enrollment of 800 participants.<br>**Screening period (W-5 ~ W-3, up to 2 weeks)**<br>After signing the Informed Consent Form, participants will undergo screening, and those who meet the inclusion/exclusion criteria could enter the Run-in period.<br>**Run-in period (W-3 ~ W0)**<br>Participants who passed the screening will start continuous oral administration of single-blind Run-in drug for 3 weeks QD from V2（D-21）.<br>The participant underwent baseline inspection at V3 (D-4), and assessments for randomization eligibility at V4 (D1). Participants who met the randomization criteria entered the 52-week double-blind treatment period.<br>**Double-Blind Treatment Period (W1-W52)**

	Participants were randomized in a 1:1:1:1 ratio to the HRS-7535 30 mg group, 60 mg group, 90 mg group, and dapagliflozin group, with stratification based on baseline HbA1c values (≤8.5%, > 8.5%) measured by the central laboratory. Double-blind treatment period medication was initiated after randomization.<br>From the screening period to the end of the double-blind treatment period, all participants should receive stable-dose metformin as background therapy throughout. All participants should undergo assessments of efficacy and safety-related indicators at the scheduled visit time points during the double-blind treatment period as per the protocol.<br>**Safety follow-up phase (W53 ~ W54)**<br>All participants discontinued the double-blind treatment period medication, and the participants will complete the safety follow-up via telephone visit at Week 54.
**Dosing Regimen**	Run-in period (single-blind)<br>All participants received HRS-7535 placebo (1 tablet of 30 mg and 1 tablet of 60 mg) and dapagliflozin placebo (1 tablet of 10 mg), QD. The investigational product was allowed to be taken on the day of V2 or the next day, and the day of administration was considered as day 1 of the Run-in period.<br>Double-blind treatment period<br>Approximately 800 participants will be randomized in a 1:1:1:1 ratio to the HRS-7535 30 mg group, 60 mg group, 90 mg group, and dapagliflozin group, with stratification based on baseline HbA1c values (≤8.5%, > 8.5%) measured by the central laboratory. The double-blind treatment period medication will be initiated after randomization. The investigational product may be administered on the day of randomization or the following day, with the dosing day designated as Day 1 of the double-blind treatment period.<br>The dosing regimens of each group are as follows:

- HRS-7535 30 mg group: HRS-7535 30 mg, administered orally, QD.
- The HRS-7535 60 mg group: dose titration was adopted. The participants received HRS-7535 30 mg, orally administered, QD for the first 4 weeks (D1 to D28); after Week 5 (after D29), they received HRS-7535 60 mg, orally administered, QD.
- HRS-7535 90 mg group: dose titration was adopted. For the first 4 weeks (D1 to D28), HRS-7535 30 mg was administered orally, QD; from Week 5 to Week 8 (D29 to D56), HRS-7535 60 mg was administered orally, QD; after Week 9 (after D57), HRS-7535 90 mg was administered orally, QD.
- Dapagliflozin group: dapagliflozin (brand name: Forxiga®), 10 mg, oral administration, QD.

From the screening period to the end of the double-blind treatment period, all participants should receive stable dosage of metformin as background therapy throughout the course.

Definition of stable dosage: the daily dosage of metformin is not changed except for medical necessity.

To ensure the implementation of blinding, the drug dosage and corresponding number of tablets administered in each group during the Run-in period and double-blind treatment period are as follows:

**Dose Level** / **Investigational Product**	**Run-in period:**	**Double-blind treatment period**			
	**All Participants**	**HRS-7535 30 mg**	**HRS-7535 60 mg**	**HRS-7535 90 mg**	**dapagliflozin group**
HRS-7535 30 mg	0	1	0	1	0
HRS-7535 30 mg placebo	1	0	1	0	1
HRS-7535 60 mg	0	0	1	1	0
HRS-7535 60 mg placebo	1	1	0	0	1
Dapagliflozin Tablets 10 mg	0	0	0	0	1
Dapagliflozin Tablets 10 mg Placebo	1	1	1	1	0

**Inclusion Criteria**	Participants must meet all the following criteria to be enrolled in this trial:<br>1. Male or female, aged between 18 and 75 years (inclusive) on the day of signing the ICF;<br>2. Type 2 diabetes mellitus with a medical history of ≥3 months was diagnosed according to the Chinese Guidelines for the Prevention and Treatment of Type 2 Diabetes (2020 Edition) at screening;<br>3. HbA1c levels between 7.5% and 11.0% (inclusive) at screening (as tested by a local laboratory);<br>4. At screening, stable treatment for type 2 diabetes for ≥8 weeks, with no planned changes to the corresponding treatment regimen during the entire trial period. At screening, the treatment regimen was stable monotherapy with metformin for ≥8 weeks on the basis of regular diet and exercise control, with a daily dose of ≥1500 mg or maximum tolerated dose of ≥1000 mg daily (metformin dose met the requirements of the package insert);<br>5. Body mass index (BMI) of 19.0 – 40.0 kg/m^2 (inclusive) at screening;<br>6. Voluntarily sign the ICF prior to the trial, fully understand the content, process, and possible adverse reactions of the study, and are able and willing to complete the study in accordance with protocol requirements (e.g., self-monitoring of blood glucose and recording participant logs);<br>7. From signing the Informed Consent Form until 2 weeks after the last dose, participants have no plans for pregnancy and are willing to use highly effective contraception as specified in the protocol.
**Exclusion criteria**	Participants who meet any of the following criteria could not be enrolled in this trial:<br>**Those with the following inspection abnormalities at screening:**<br>1. Presence of the following laboratory inspection abnormalities at screening or randomization:<br>• Fasting venous glucose > 15 mmol/L;

- Alanine aminotransferase (ALT) or aspartate aminotransferase (AST) > 2 × upper limit of normal (ULN);
- Total bilirubin (TBIL) > 1.5 × ULN;
- Amylase and/or lipase > 3 × ULN;
- Triglycerides (TG) under fasting conditions > 11.3 mmol/L;
- Calcitonin ≥50 ng/L;
- Estimated glomerular filtration rate (eGFR) < 45 mL/min/1.73 m2 (calculated using the chronic kidney disease epidemiology collaboration [CKD-EPI] equation);
- Urinary albumin creatinine ratio (UACR) ≥1000 mg/g;
- Positive Hepatitis B Virus Surface Antigen (HBsAg) and HBV-DNA > ULN;

2. Clinically significant abnormalities in ECG results at screening or randomization that could affect participant safety, including but not limited to supraventricular tachycardia, atrial fibrillation, atrial flutter, second- or third-degree atrioventricular block, etc.;
3. The electrocardiogram inspection results during screening or randomization indicated a heart rate > 100 bpm or QTcF > 480 ms. If the heart rate or QTcF is abnormal, two additional electrocardiograms could be performed to obtain the mean of three values;
4. Severe uncontrolled hypertension (systolic pressure ≥160 mmHg or diastolic pressure ≥100 mmHg) at screening or randomization;

Those with the following diseases or medical history prior to screening:

5. Diagnosed or suspected type 1 diabetes mellitus, special type of diabetes mellitus or secondary diabetes mellitus;
6. Acute complications of diabetes (diabetic ketoacidosis, lactic acidosis, hyperglycemic hyperosmolar state, etc.) within 6 months prior to screening;
7. Severe hypoglycemic episodes or recurrent hypoglycemic episodes within 6 months prior to screening (≥ 3 hypoglycemic episodes

	within a week, or other hypoglycemic episodes as determined by the investigator);<br>8. Presence of proliferative retinopathy or maculopathy, or painful diabetic neuropathy requiring acute treatment, diabetic foot ulcer, or intermittent claudication at screening;<br>9. Participants with the following clinically significant diseases within 6 months prior to screening: including but not limited to neurological, mental, cardiovascular, endocrine, gastrointestinal, respiratory, urinary, hematological, immune system and other diseases (except for type 2 diabetes related conditions) that may interfere with the study results or pose additional risks to the administration of the study drug as determined by the investigator;<br>10. Participants with a personal or family history of medullary thyroid carcinoma (MTC) or multiple endocrine neoplasia type 2 (MEN 2);<br>11. Participants with prior or known history of acute or chronic pancreatitis, pancreatic injury, acute cholecystitis, or symptomatic gallbladder disease requiring treatment, except for those who have undergone cholecystectomy and are deemed eligible for enrollment by the investigator;<br>12. Prior history of significant gastrointestinal diseases (such as gastric outlet obstruction, inflammatory bowel disease, active ulcer, etc.), or those who have undergone gastrointestinal surgery (except for gastrointestinal polypectomy and appendectomy), or those who have taken drugs that directly affect gastrointestinal motility for a long time;<br>13. Hyperthyroidism medical history before screening, or hyperthyroidism at screening, or other thyroid disorders not stably controlled (except for participants with thyroid hormone alternative treatment regimen stable for at least 8 weeks and no plan to change the treatment regimen during the study);<br>14. History of severe cardiovascular and cerebrovascular diseases within 6 months prior to screening, including but not limited to: congestive heart failure (New York Heart Association [NYHA]

Grade 3-4), unstable angina, stroke, myocardial infarction, severe arrhythmia, or coronary revascularization; or planned to undergo coronary, carotid or peripheral artery revascularization at screening;

15. Participants with known or suspected depression, bipolar disorder, suicidal tendency, schizophrenia or other severe mental diseases; or those who are mentally incapacitated or have language disorders, unable to fully understand the protocol or unwilling to cooperate with the study site staff;
16. History of neoplasm malignant within 5 years prior to screening, except for cured local cancer, such as: local skin basal cell carcinoma, cervical carcinoma in situ, and carcinoma in situ of prostate;
17. At screening or randomization, the participant was in a state of urinary tract infection or genital infection, or had a medical history of urinary tract infection or genital infection caused by the use of SGLT2 inhibitors;

Those who have received any of the following drugs or treatments prior to screening:

18. Use of glucagon-like peptide-1 (GLP-1) receptor agonist drugs (including multi-target GLP-1 receptor agonist drugs) within 3 months prior to screening;
19. Received systemic glucocorticoid therapy within 3 months prior to screening (except for cumulative use <14 days, or topical, inhaled, intraocular, or intranasal administration);
20. Use of tricyclic antidepressants, atypical antipsychotics and mood stabilizers (such as imipramine, chlorpromazine, amitriptyline, mirtazapine, clozapine, olanzapine, paroxetine, phenelzine, thioridazine, valproic acid and its derivatives, lithium salts, etc.) within 3 months before screening;
21. Weight loss therapy within 3 months prior to screening, including intensive diet or exercise therapy for weight loss, drug therapy,

	surgical treatment (except for liposuction history more than 1 year), health products or other special therapies;<br>**Those with any of the following conditions:**<br>22. Self-reported body weight change of >±5% within 3 months prior to screening or randomization, or planned intensive diet or exercise therapy for weight loss during the trial (except for diabetes diet and exercise control measures);<br>23. those who have participated in any clinical trial of drugs or medical devices within 3 months prior to screening or still within 5 half-lives of the study drug (whichever is longer). Participation in a clinical trial is defined as signing informed consent and having used the investigational product (including placebo) or investigational medical device;<br>24. Known or suspected hypersensitivity to the study drug or its components;<br>25. Known or suspected history of drug or alcohol abuse, and judged by the Investigator as unsuitable for participation in this clinical trial;<br>26. Participants with a history of blood donation or blood loss of ≥ 400 mL within 3 months prior to screening, or who have received blood transfusion;<br>27. Female participants who are pregnant, lactating, or planning to become pregnant during the study period;<br>28. Intolerance to venipuncture or needle phobia;<br>29. Any condition determined by the investigator to be unsuitable to participate in this clinical trial.
**Randomization Criteria**	Based on the V3 inspection results, the participant was reconfirmed to meet the following randomization criteria before randomization. Participants who failed randomization in this study were not eligible for rescreening.<br>1. HbA1c 7.5% - 11.0% (central laboratory results);

	2. Glucose under fasting conditions ≤15.0 mmol/L (central laboratory results);<br>3. Laboratory tests, vital signs, body weight, BMI, electrocardiogram, pregnancy status, etc. met the inclusion criteria and did not meet the exclusion criteria;<br>4. Medication compliance ≥ 80% and ≤ 120% during the Run-in period;<br>5. The metformin treatment regimen remained stable during the Run-in period;<br>6. No antihyperglycemic drugs other than those specified in the protocol were used during the Run-in period;<br>7. No condition considered by the investigator to be unsuitable for participation in this clinical trial occurred during the Run-in period.
**Efficacy evaluation**	Efficacy evaluation indicators include HbA1c, FPG, 2-hour postprandial plasma glucose, fasting serum insulin and C-peptide, 2-hour postprandial serum insulin and C-peptide, HOMA-β, HOMA-IR, 7-point SMBG profile, weight, and waist circumference.
**PK Evaluation**	PK blood samples were collected from the participant at V4, V6, V7, V8, V9, V10, V11, V12, and V15, and the plasma concentrations of HRS-7535, dapagliflozin, and metformin were measured.
**Safety evaluation**	Safety evaluation included collection of AEs, hypoglycemic episodes, vital signs and physical examination, 12-lead ECG, laboratory inspection, diabetic retinopathy screening and assessment.
**Sample size Estimation**	The sample size calculation is based on the change from baseline in HbA1c at Week 32. Referring to the commonly used margin for HbA1c, the non-inferiority margin for treatment difference in this study will be set at 0.4%. Participants will be randomized into groups at the ratio of 1:1:1:1. Assuming an treatment difference of 0 after considering intercurrent events and a standard deviation of 1.1%, 800 participants (200 per group) will provide an overall power of 87% to detect non-inferiority of HRS-7535 at all dosage groups (30 mg, 60 mg, 90 mg)

	compared to the dapagliflozin group at a one-sided significance level of 0.025.<br>In addition, assuming that the treatment difference between each dosage group of HRS-7535 (30 mg, 60 mg, 90 mg) and the dapagliflozin group after considering intercurrent event is -0.4%, with a standard deviation of 1.1%, then 800 participants (200 participants per group) will provide an overall power of 87% to detect the superiority of each dosage group of HRS-7535 (30 mg, 60 mg, 90 mg) over the dapagliflozin group at a one-sided significance level of 0.025.
**Statistical Analysis**	**Definition of Analysis Set**<br>Intention-to-treat (ITT) analysis set: this population included all randomized participants. Participants will be analyzed according to the group they are randomly assigned to. ITT is the primary analysis population for efficacy.<br>Safety set (SS): includes all participants who received at least one dose of the investigational product after randomization. Participants will be analyzed according to the group they were randomly assigned to.<br>PK concentration set (PKCS): all participants who have been randomized to receive HRS-7535 treatment and have at least one evaluable PK concentration data post-dose.<br>**Analysis of primary efficacy endpoints**<br>For the primary estimand, the primary study endpoint will be analyzed based on the ITT set. It will be analyzed using a mixed-effect model repeated measure (MMRM) based on restricted estimation maximum likelihood (REML). The model included treatment, Visit, and the interaction between treatment and Visit as fixed effects, baseline HbA1c as the covariate, and Participant as the random effect. The UN or alternative CS covariance matrix was employed. The least squares mean, standard error, 95% confidence interval (CI) of the change from baseline in HbA1c at Week 32 for each group and between groups, as well as the p-value for the difference between each dosage group of HRS-7535 and the dapagliflozin group, were estimated and listed.

	Hypothetical strategy was applied for all intercurrent events, data after intercurrent events were not included in the analysis.<br>A sensitivity analysis will be performed on the primary estimand. See the Statistical Analysis Plan for the detailed method.<br>**Analysis of secondary efficacy endpoints**<br>The secondary efficacy endpoints will be analyzed based on the ITT.<br>The generalized estimating equation (GEE) will be used to analyze the binary endpoint (response/no response).<br>For continuous variable endpoints with two or more repeated measurements after randomization, MMRM model will be used for analysis. For continuous variable endpoints with only one measurement after randomization, analysis of covariance (ANCOVA) will be used, and missing data will be imputed using last observation carried forward (LOCF).<br>**Analysis of other secondary endpoints**<br>The changes in DTSQs scores from baseline at Week 32 and Week 52 will be analyzed using ANCOVA, with missing data imputed by LOCF.<br>**Safety Analysis**<br>All safety analyses were based on SS.<br>Descriptive statistics will be provided for safety endpoints, which include AEs, hypoglycemic episodes, laboratory tests, vital signs, physical examination, 12-lead ECG, and diabetic retinopathy screening and assessment.<br>**Pharmacokinetic Endpoint Analysis**<br>Based on PKCS, plasma concentrations of HRS-7535, dapagliflozin and metformin will be summarized using descriptive statistics (as applicable).

1.2. Schema

Figure 1. Overview of study design1

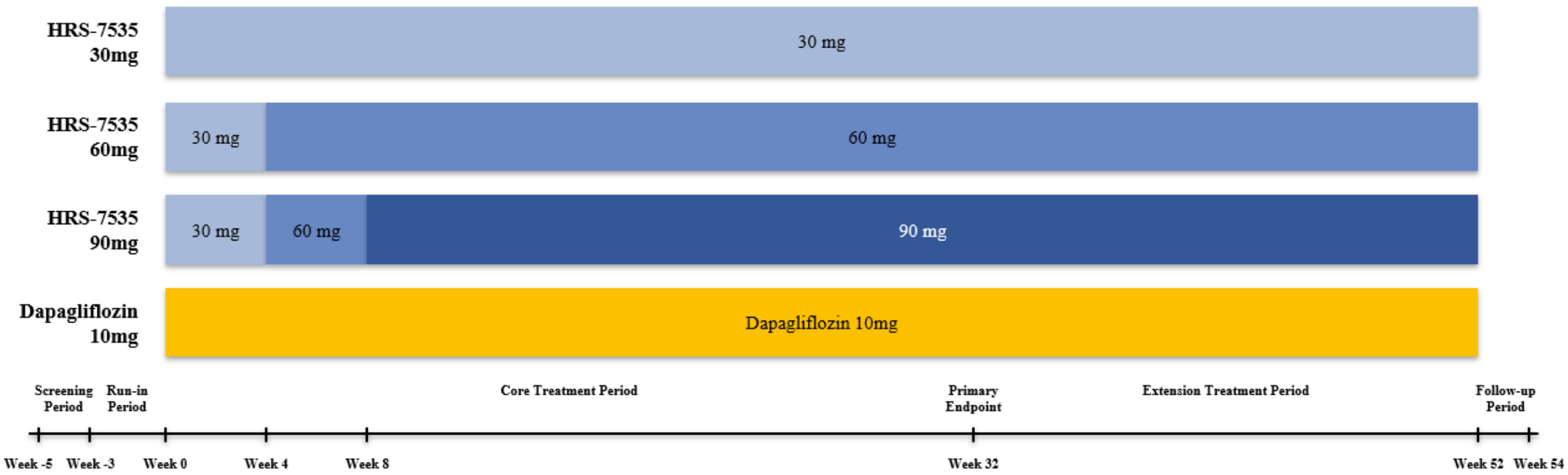

Figure 1. Overview of study design1

1.3. Schedule of activities

Table 1. Study Flowchart1

In order to protect the health of participants, the investigator may arrange unscheduled visits in addition to the visits in the table below.

Table 1. Study Flowchart1

**Item**	**Screening Period[1]**	**Run-in period:**		**Treatment Period**													**Safety Follow-up**
**Visit**	**V1**	**V2**	**V3**	**V4**	**V514**	**V6**	**V7**	**V8**	**V9**	**V10**	**V11**	**V12**	**V13**	**V14**	**V15**	**Early discontinuation[15]**	**V16[14]**
**Visit Time (weeks)**	**W-5 ~ W-3**	**W-3**	**W-1 ~ W0**	**W1**	**W2**	**W4**	**W8**	**W12**	**W16**	**W20**	**W26**	**W32**	**W38**	**W44**	**W52**	**/**	**W54/ 14 days after withdrawal**
**Visit Time (days)**	**D-35~ D-22**	**D-21**	**D-4**	**D1**	**D15**	**D29**	**D57**	**D85**	**D113**	**D141**	**D183**	**D225**	**D267**	**D309**	**D364**	**/**	
**Time Window (days)**	**/**	**±3**	**-3**	**0**	**±3**	**±3**	**±3**	**±3**	**±3**	**±3**	**±3**	**±3**	**±5**	**±5**	**±5**	**/**	**±3**
**Visit under fasting conditions**	**X**	**X**	**X**	**X**	☎ **14**	**X**	**X**	**X**	**X**	**X**	**X**	**X**	**X**	**X**	**X**	**X**	☎ [14]
**Participant information and clinical evaluation**																	
Signature of informed consent form	X																
Assignment of screening number	X																
Inclusion and exclusion criteria	X																
Randomization criteria[2]				X													
Assigning of randomization number				X													

Item	Screening Period[1]	Run-in period:		Treatment Period													Safety Follow-up
**Visit**	**V1**	**V2**	**V3**	**V4**	**V514**	**V6**	**V7**	**V8**	**V9**	**V10**	**V11**	**V12**	**V13**	**V14**	**V15**	**Early discontinuation [15]**	**V16[14]**
**Visit Time (weeks)**	**W-5 ~ W-3**	**W-3**	**W-1 ~ W0**	**W1**	**W2**	**W4**	**W8**	**W12**	**W16**	**W20**	**W26**	**W32**	**W38**	**W44**	**W52**	**/**	**W54/ 14 days after withdrawal**
**Visit Time (days)**	**D-35~ D-22**	**D-21**	**D-4**	**D1**	**D15**	**D29**	**D57**	**D85**	**D113**	**D141**	**D183**	**D225**	**D267**	**D309**	**D364**	**/**	
**Time Window (days)**	**/**	**±3**	**-3**	**0**	**±3**	**±3**	**±3**	**±3**	**±3**	**±3**	**±3**	**±3**	**±5**	**±5**	**±5**	**/**	**±3**
Demographic data	X																
Past medical history	X																
Prior and concomitant Treatment	X	X	X	X	X	X	X	X	X	X	X	X	X	X	X	X	X
History of tobacco and alcohol use	X																
Height	X																
BMI	X		X														
Body weight and waist circumference	X		X			X	X	X	X	X	X	X	X	X	X	X	
**Safety evaluation**																	
Adverse event	X	X	X	X	X	X	X	X	X	X	X	X	X	X	X	X	X
Hypoglycemic episodes		X	X	X	X	X	X	X	X	X	X	X	X	X	X	X	X
Vital signs [3]	X		X			X	X	X	X	X	X	X	X	X	X	X	
Physical examination [4]	X		X									X			X	X	

Item	Screening Period[1]	Run-in period:		Treatment Period													Safety Follow-up
**Visit**	**V1**	**V2**	**V3**	**V4**	**V514**	**V6**	**V7**	**V8**	**V9**	**V10**	**V11**	**V12**	**V13**	**V14**	**V15**	**Early discontinuation[15]**	**V16[14]**
**Visit Time (weeks)**	**W-5 ~ W-3**	**W-3**	**W-1 ~ W0**	**W1**	**W2**	**W4**	**W8**	**W12**	**W16**	**W20**	**W26**	**W32**	**W38**	**W44**	**W52**	**/**	**W54/ 14 days after withdrawal**
**Visit Time (days)**	**D-35~ D-22**	**D-21**	**D-4**	**D1**	**D15**	**D29**	**D57**	**D85**	**D113**	**D141**	**D183**	**D225**	**D267**	**D309**	**D364**	**/**	
**Time Window (days)**	**/**	**±3**	**-3**	**0**	**±3**	**±3**	**±3**	**±3**	**±3**	**±3**	**±3**	**±3**	**±5**	**±5**	**±5**	**/**	**±3**
Hematology	X		X			X		X		X		X			X	X	
Urinalysis	X		X			X		X		X		X			X	X	
UACR	X		▲			▲	▲		▲		▲	▲			▲	▲	
Clinical chemistry	X		X			X	X	X	X	X	X	X	X	X	X	X	
Lipids	X		X				X			X		X			X	X	
Amylase [5]	X		X				X			X		X			X	X	
Lipase [5]	X		X				X			X		X			X	X	
Coagulation test	X																
Thyroid function test	X																
Calcitonin [5]	X						X					X			X	X	
12-lead electrocardiogram [6]	X		X				X			X		X			X	X	
Pregnancy test[7]	X		X				X			X		X			X	X	

Item	Screening Period[1]	Run-in period:		Treatment Period													Safety Follow-up
**Visit**	**V1**	**V2**	**V3**	**V4**	**V514**	**V6**	**V7**	**V8**	**V9**	**V10**	**V11**	**V12**	**V13**	**V14**	**V15**	**Early discontinuation[15]**	**V16[14]**
**Visit Time (weeks)**	**W-5 ~ W-3**	**W-3**	**W-1 ~ W0**	**W1**	**W2**	**W4**	**W8**	**W12**	**W16**	**W20**	**W26**	**W32**	**W38**	**W44**	**W52**	**/**	**W54/ 14 days after withdrawal**
**Visit Time (days)**	**D-35~ D-22**	**D-21**	**D-4**	**D1**	**D15**	**D29**	**D57**	**D85**	**D113**	**D141**	**D183**	**D225**	**D267**	**D309**	**D364**	**/**	
**Time Window (days)**	**/**	**±3**	**-3**	**0**	**±3**	**±3**	**±3**	**±3**	**±3**	**±3**	**±3**	**±3**	**±5**	**±5**	**±5**	**/**	**±3**
Follicle-stimulating hormone (menopausal women only)[7]	X																
Hepatitis B virus test	X																
Screening and assessment of diabetic retinopathy [8]	X											X8			X8	X	
**EFFICACY EVALUATION**																	
Glycated Hemoglobin A1C [9]	X		▲			▲	▲	▲	▲	▲	▲	▲	▲	▲	▲	▲	
Fasting plasma glucose[9]	X		▲			▲	▲	▲	▲	▲	▲	▲	▲	▲	▲	▲	
Fasting serum insulin and C-peptide			▲						▲			▲			▲	▲	
2-hour postprandial plasma glucose, serum Insulin, serum C-peptide [10]			▲									▲			▲	▲	
Fasting SMBG[11]		X	→	→	→	→	→	→	→	→	→	→	→	→	X		
7-point SMBG			X						X			X			X	X	
DTSQs	X		X									X			X	X	

**Item**	**Screening Period[1]**	**Run-in period:**		**Treatment Period**													**Safety Follow-up**
**Visit**	**V1**	**V2**	**V3**	**V4**	**V514**	**V6**	**V7**	**V8**	**V9**	**V10**	**V11**	**V12**	**V13**	**V14**	**V15**	**Early discontinuation[15]**	**V16[14]**
**Visit Time (weeks)**	**W-5 ~ W-3**	**W-3**	**W-1 ~ W0**	**W1**	**W2**	**W4**	**W8**	**W12**	**W16**	**W20**	**W26**	**W32**	**W38**	**W44**	**W52**	**/**	**W54/ 14 days after withdrawal**
**Visit Time (days)**	**D-35~ D-22**	**D-21**	**D-4**	**D1**	**D15**	**D29**	**D57**	**D85**	**D113**	**D141**	**D183**	**D225**	**D267**	**D309**	**D364**	**/**	
**Time Window (days)**	**/**	**±3**	**-3**	**0**	**±3**	**±3**	**±3**	**±3**	**±3**	**±3**	**±3**	**±3**	**±5**	**±5**	**±5**	**/**	**±3**
PK samples[13]				▲		▲	▲	▲	▲	▲	▲	▲			▲	▲	
**Participant Guidance**																	
Diet and lifestyle education	X	X	X	X	X	X	X	X	X	X	X	X	X	X	X	X	X
Contraceptive guidance	X	X	X	X	X	X	X	X	X	X	X	X	X	X	X	X	
Glucose meter instructions		X	X	X	X	X	X	X	X	X	X	X	X	X			
Participant diary usage guidance		X	X	X	X	X	X	X	X	X	X	X	X	X			
Dispensation of investigational product		☆		★		★	★	★	★	★	★	★	★	★			
Accountability of returned investigational product				☆		★	★	★	★	★	★	★	★	★	★	★	
Compliance inspection			X	X	X	X	X	X	X	X	X	X	X	X	X	X	
Telephone visit						☎	☎										

Abbreviations: ☎=telephone visit; ☆=Run-in period drug; ★=double-blind period drug; ▲=central laboratory test; BMI=body mass index; DTSQs=Diabetes Treatment Satisfaction Questionnaire current version; PK=Pharmacokinetics; SMBG=self-monitoring of blood glucose; UACR=Urinary albumin/creatinine ratio; →=ongoing.

1. Screening period: participants could proceed with the screening process only after signing the Informed Consent Form. If an inspection during the screening period is abnormal and there is a valid reason for retesting, it could be retested once within 1 week (HbA1c could only be retested in cases of objective circumstances such as sample collection errors, sample contamination, or sample disqualification). Participants who experience screen failure may undergo re-screening evaluation after 1 month if there is sufficient justification. During re-screening, a new screening number should be assigned, and the Informed Consent Form must be re-signed. Only one re-screening is permitted.

2. Randomization criteria: the complete Run-in period was from D-21 to D-1, and the baseline inspection should be completed at V3 (D-4). Retesting of the V3 baseline inspection is not allowed (except for objective circumstances such as incorrect sample collection, sample contamination, or unqualified samples). Participants who successfully passed the screening but did not continue to participate in the trial, failed to meet the randomization criteria, or met the randomization criteria but did not participate in the randomization allocation were considered randomization failures. Participants who failed randomization were not eligible for re-screening.

3. Vital signs: including systolic pressure, diastolic pressure, and pulse.

4. Physical examination: including general condition, skin, lymph nodes, head and neck, chest (including heart and lungs), abdomen, the musculoskeletal system, and nervous system.

5. Amylase, Lipase, Calcitonin: if the local laboratory could not test Lipase and Calcitonin, they should be tested at the central laboratory.

6. 12-lead ECG: including heart rate, PR, RR, QT, and QTcF. Participants will be monitored after at least 10 min of rest in sitting position; see Section 8.3.3 of the protocol for details; if cardiac AEs occur during the study, ECG frequency could be increased at the discretion of the investigator.8.3.3

7. Pregnancy test: for female participants of childbearing potential, serum pregnancy tests will be performed at V1, V3, V12, and V15. For subsequent visits, urine or serum pregnancy tests may be conducted if the participant is not in menstruation. The investigator may perform additional pregnancy tests as clinically indicated during the study. Postmenopausal women: for those > 45 years and ≤65 years old, both Follicle Stimulating Hormone (FSH) and serum pregnancy tests are required during the screening period. The decision on pregnancy testing during subsequent visits will be based on the FSH results. For those > 65 years old, FSH and pregnancy tests are not required. Detailed regulations are described in Section 13.1.3.13.1.3

8. Diabetic retinopathy screening and assessment: all participants will undergo fundus photography at Week 32 (±2 weeks) and Week 52 Visit (-4 weeks).

9. HbA1c, fasting plasma glucose: HbA1c and fasting plasma glucose are tested at the local laboratory during screening and at the central laboratory during other visits. If some sites could not test plasma glucose during the screening period, serum glucose results are acceptable.

10. 2 h postprandial plasma glucose, serum insulin, and serum C-peptide: in the morning under fasting conditions, after consuming a mixed meal, venous blood samples were collected at 2 h ± 5 min (starting from the first bite) for the detection of plasma glucose, serum insulin, and serum C-peptide; fasting was maintained from the consumption of the mixed meal until the 2 h postprandial blood collection, as detailed in Section 8.2.4.8.2.4

11. Fasting SMBG: it is recommended to measure fasting fingertip blood glucose at least once weekly before dosing during the visit period. If hypoglycemia is suspected, fingertip blood glucose should be tested promptly.

12. 7-point SMBG: conduct continuous 7-point SMBG monitoring on any one day within 3 days before the Visit (starting before breakfast on that day and completing 7-point blood glucose collection within 24 hours), including measurements before (recommended within 30 minutes before meal) and after (120 ± 10 minutes after starting each meal) breakfast, lunch, and dinner, as well as bedtime measurement. If one complete 7-point SMBG test is not completed, a second 7-point SMBG monitoring needs to be performed on a different day.

13. PK samples: PK blood collection will be performed at the scheduled visits, and the time of the second dose before blood collection must be accurately recorded. If a participant withdraws early, a PK blood sample should also be collected at the time of withdrawal (except for those whose withdrawal visit is more than 48 hours after the last dose). At least 40 participants will undergo intensive blood collection at the center during any one visit from V8 to V11, collecting PK blood samples at -60 min before dosing and at 0.5 h (±15 min), 1 h (±20 min), 2 h (±30 min), 4 h (±30 min), 6 h (±30 min), 8 h (±30 min), and 10 h (±30 min) after dosing of HRS-7535/placebo on the day of collection. PK blood samples will be assayed for concentrations of HRS-7535, dapagliflozin, and metformin.

14. Telephone visit: V5 and V16 were telephone visits, and the Investigator could change them to site visits if necessary.

15. Early discontinuation: randomized participants who withdraw early from the study should complete the early withdrawal visit as soon as possible. If the early withdrawal visit occurs ≤ 1 week from the last visit (excluding V4), only a blood pregnancy test for females is required without repeating other safety inspections, unless deemed necessary by the investigator.

16. Telephone inquiry: telephone inquiry was conducted 1 to 2 weeks after V6 and V7 visits to inquire about the general condition of the participant and provide medication guidance.

Note:

1. If the participant has a HBV DNA test report within 1 month recognized by the Investigator during Screening, it could be accepted as the Screening period inspection.

2. Participants are not required to be under fasting conditions during V2 and V4 visits to the hospital.

2. Introduction

2.1. Study background

2.1.1. Disease background

Diabetes is a group of metabolic diseases characterized by chronic hyperglycemia caused by multiple etiologies. Long-term hyperglycemia could cause multi-system damage, leading to chronic progressive lesions, functional decline and failure of tissues and organs such as eyes, kidneys, nerves, heart, and blood vessels. According to the Global Diabetes Overview (10th Edition) released by the International Diabetes Federation in 2021, there were already 537 million diabetic patients (prevalence rate 10.5%) worldwide in 2021. China ranked first with about 140.9 million diabetic patients[1]. Diabetes and its comorbidities are imposing a huge economic burden on healthcare systems worldwide. Type 2 diabetes accounts for more than 90% of all diabetes cases[2].

Type 2 diabetes still lacks etiological treatment, and the main approach is comprehensive management, including dietary control, reasonable exercise, blood glucose monitoring, diabetes education, and the use of hypoglycemic drugs. The hypoglycemic drugs currently marketed in China mainly include biguanides, sulfonylureas, glinides, α-glucosidase inhibitors, thiazolidinediones, dipeptidyl peptidase-4 (DPP-4) inhibitors, sodium-glucose cotransporter-2 (SGLT2) inhibitors, glucagon-like peptide-1 (GLP-1) receptor agonists, and insulin[3,4]. Although there are numerous therapeutic drugs available, the treatment and control rates of diabetes in China still need improvement. The 2013 epidemiological survey results showed rates of 32.2% and 49.2%, respectively[3]. [3]

Most patients with type 2 diabetes require more than one drug to control blood glucose, and combination therapy is an important mode of comprehensive management for these patients. Domestic guidelines[3] recommend metformin as the first-line hypoglycemic drug for patients with type 2 diabetes. If metformin monotherapy fails to achieve glycemic targets, it should be combined with other hypoglycemic drugs such as SGLT2 inhibitors. The 2024 American Diabetes Association (ADA) guidelines[5] emphasize "rational glycemic control, weight management, and improvement of cardiorenal outcomes." For patients with type 2 diabetes who have atherosclerotic cardiovascular disease, high risk of atherosclerotic cardiovascular disease, cardiac failure, or chronic kidney disease, SGLT2 inhibitors or GLP-1 receptor agonists with proven clinical benefit could be initiated regardless of Glycated Hemoglobin A1C levels. The new guidelines also state that, if conditions permit, GLP-1 receptor agonists should be prioritized over insulin when injectable hypoglycemic drugs are needed. For those

who must use insulin, combination with GLP-1 receptor agonists is recommended, reaffirming the advantageous position of GLP-1 receptor agonists in the comprehensive management of patients with type 2 diabetes.[35]

2.1.2. HRS-7535

GLP-1 receptor agonists and SGLT-2 inhibitors are novel glucose-lowering drugs that have been widely used in clinical practice in recent years. GLP-1 receptor agonists could increase glucose-dependent insulin secretion, inhibit glucagon secretion, enhance glucose uptake in muscle tissue, reduce hepatic glucose output, augment the incretin effect, and improve neurotransmitter dysfunction, covering six of the "ominous octet" in the pathogenesis of diabetes[6]. SGLT-2 inhibitors primarily act by inhibiting glucose reabsorption in the proximal renal tubules, lowering the renal glucose threshold and promoting glucose excretion in urine, thereby reducing circulating glucose level[7].

HRS-7535 is a small molecule oral GLP-1 receptor agonist independently developed by our company, intended for the treatment of type 2 diabetes and weight loss. Dapagliflozin is an approved SGLT2 inhibitor, suitable for use in combination with diet and exercise, and in conjunction with metformin to improve glycemic control. This study plans to evaluate the glucose-lowering efficacy and safety of HRS-7535 compared with dapagliflozin in adult participants with type 2 diabetes who have inadequate glycemic control after metformin treatment.

2.1.3. Non-clinical experience

Preclinical toxicology studies of HRS-7535 conducted dose range-determining study in rats and cynomolgus monkeys, 4-week and 26-week repeated dose toxicity study in rats and 4-week and 39-week repeated dose toxicity study in cynomolgus monkeys, fertility and early embryonic developmental toxicity study in rats, embryo-fetal developmental toxicity study in transgenic mice and rabbits expressing human GLP-1 receptor, and genotoxicity study.

In the dose range finding study of HRS-7535 with 1-week repeated oral administration at 30, 100 and 300 mg/kg in rats, the maximum tolerated dose (MTD) was 300 mg/kg, and the no observed adverse effect level (NOAEL) was 100 mg/kg. The primary toxicities were decreased reticulocyte count and increased platelet count. In the 2-week repeated nasogastric administration of 5, 100 and 300 mg/kg HRS-7535 dose range finding study in cynomolgus monkeys, animal deaths were observed in the 300 mg/kg group, primarily showing toxicity to the kidneys and gastrointestinal tract, with an MTD of 100 mg/kg and NOAEL of 5 mg/kg.

The NOAEL for the 4-week repeated dose toxicity study of HRS-7535 in SD rats at doses of 10, 50 and 250 mg/kg was 50 mg/kg; the main toxicity in the 250 mg/kg group was manifested as increased blood phosphorus in animals at the end of administration (mean change level: female: ↑22.7%; male: ↑14.2%). The NOAEL for the 26-week repeated dose toxicity study of HRS-7535 in SD rats at doses of 15, 50 and 150 mg/kg/day was 150 mg/kg/day.

The NOAEL for the 4-week repeated administration of 2, 8 and 32 mg/kg HRS-7535 toxicity study in cynomolgus monkeys was 8 mg/kg; the main toxicity manifested as increased white blood cells count(WBC) and lymphocyte cells count (LYMP) in male animals during the recovery period (mean change level: WBC: ↑263.3%; LYMP: ↑444.5%), and decreased absolute value of reticulocytes（RET#) and percentage of reticulocytes (RET%) were observed in females (mean change level: RET%: ↓52.6%; RET#: ↓47.6%). The NOAEL for the 39-week repeated administration of 2, 10 and 50 mg/kg HRS-7535 toxicity study in cynomolgus monkeys was 10 mg/kg, the main toxicity manifested as sustained decrease of LDH in high-dose (50 mg/kg) group of both sexes from D61 (mean change level: ↓49%-63%), with no recovery during the recovery period.

HRS-7535 is a small molecule agonist of the GLP-1 receptor, which promotes pancreatic insulin secretion, reduces glucagon secretion, inhibits gastric emptying, and affects the central nervous system to enhance satiety and suppress appetite, thereby directly reducing energy intake. Cynomolgus monkeys, as a non-rodent toxicological species relevant to pharmacological effects, showed a decrease in the hematological parameter LDH only in the high-dosage group (50 mg/kg) after 39 weeks of repeated administration. Typically, a decrease in LDH may be observed in conditions such as malnutrition and chronic wasting disease. No histopathological changes were observed in the liver or related tissues, suggesting that the decrease was mediated by the activation of the GLP-1 receptor, leading to reduced food intake and body weight. The exposure ratio of this dosage at the last administration was 4 times that of the clinical daily oral dose of 120 mg, indicating a low clinical risk.

In the genotoxicity studies of HRS-7535, the Ames test yielded negative results, indicating a low risk of DNA point mutations for HRS-7535. In addition, the results of chromosomal aberration test and rat bone marrow micronucleus test were negative. Therefore, the genotoxicity risk of HRS-7535 in humans is expected to be low.

In the fertility and early embryonic development toxicity study in SD rats, the NOAEL for HRS-7535 in parental male and female animals was 75 mg/kg. In the 150 mg/kg dose group, decreased/slowed mean body weight gain was observed in male animals, and decreased

absolute body weight during pregnancy was observed in female animals. The NOAEL for HRS-7535 on fertility and early embryonic development in SD rats was 150 mg/kg. In the embryo-fetal developmental toxicity study in transgenic mice expressing human GLP-1 receptors (pharmacologically relevant rodents), the NOAEL for maternal toxicity of HRS-7535 was 60 mg/kg, and the NOAEL for embryo-fetal development was 20 mg/kg (based on exposure, 0.7 times the exposure ratio of the clinical daily oral dose of 120 mg). In 6 high-dose (180 mg/kg) pregnant animals, test article-related histopathological changes were observed, including increased thymic cortex-to-medulla ratio, lymphocyte depletion predominantly in the cortex, focal erosion/ulcer/inflammation in the non-glandular stomach, focal squamous epithelial hyperplasia, and increased extramedullary hematopoiesis in the spleen. Additionally, individual animals in the high-dose group showed emaciation and reduced activity after administration. Three animals exhibited complete resorption of all implantation sites, with a high post-implantation loss rate. In dose groups ≥60 mg/kg, the mean fetal weight and length per litter were significantly reduced, the incidence of skeletal abnormalities was increased, and the number of ossification sites was significantly decreased. In the embryo-fetal developmental toxicity study in rabbits (pharmacologically non-relevant non-rodents), the NOAEL for parental pregnant rabbits was 150 mg/kg (based on exposure, 0.8 times the exposure ratio of the clinical daily oral dose of 120 mg), and the NOAEL for embryo-fetal development was the high dose of 300 mg/kg.

It is speculated that the toxicity to embryo-fetal development in transgenic mice expressing human GLP-1 receptors exposed to HRS-7535 at doses below the maximum recommended human dose (MRHD) may be secondary to the effects on the maternal body after activation of GLP-1 receptors and impaired function of the inverted yolk sac placenta in rodents (in primates, the yolk sac does not invert to form a placenta). Food restriction has been confirmed to lead to increased pregnancy loss and fetal abnormalities, which is consistent with the toxicity profile of marketed GLP-1 analogs. The above results suggest that participants in the HRS-7535 clinical study should use strict contraceptive measures.

The nonclinical study results of HRS-7535 are detailed in the Investigator's Brochure (IB) of HRS-7535.

2.1.4. Clinical experience

As of May., 2024, HRS-7535 has conducted 7 clinical studies, namely HRS-7535-101 study, HRS-7535-102 study, HRS-7535-103 study, HRS-7535-201 study, HRS-7535-202 study,

HRS-7535-203 study, and HRS-7535-205 study. The HRS-7535-101 study and HRS-7535-201 study have been completed, and the remaining studies are ongoing.

HRS-7535-101 is a randomized, double-blind, dose-escalation, placebo-controlled Phase I study in healthy participants. Pharmacokinetic (PK) results showed that: at doses of 15 mg to 120 mg, the Tmax of oral HRS-7535 was approximately 6 hours under fasting conditions and about 12 h postprandially; the terminal elimination half-life was 5.28 to 9.08 h; exposure increased correspondingly with dose escalation, exhibiting approximately linear PK characteristics; after multiple oral administrations of HRS-7535, steady state was achieved in approximately 3 days, with mild accumulation of systemic exposure compared to single-dose administration; food intake slowed the absorption rate of HRS-7535, reduced peak concentration (cmax), and slightly decreased the area under the concentration-time curve (AUC), presenting PK characteristics similar to sustained-release formulations, which may not significantly affect the relative bioavailability of HRS-7535. In the HRS-7535-101 study, a total of 44 healthy participants received doses ranging from 15 mg to 120 mg, and the incidence of treatment emergent adverse events (TEAEs) was 86.4% (38/44), with all TEAEs being mild. No serious adverse events (SAEs), fatal TEAEs, or adverse events of special interest (AESIs) occurred. No participants permanently discontinued treatment due to TEAEs. For detailed safety information, please refer to the HRS-7535 IB.

Study HRS-7535-102 was a single-dose, open-label study conducted in participants with moderate renal impairment and participants with normal renal function, aiming to compare the PK characteristics of HRS-7535 between these two groups. As of Mar., 2024, a total of 16 participants were enrolled, including 8 participants with moderate renal impairment and 8 participants with normal renal function. All 16 participants received a single dose of HRS-7535 30 mg. Three participants (37.5%) with moderate renal impairment experienced at least one TEAE, all of which were mild. No participants experienced AESI, SAE, or TEAE leading to death or study withdrawal.

Study HRS-7535-103 is a bioavailability and food-effect study conducted in healthy participants, aiming to evaluate the relative bioavailability of HRS-7535 with different formulation processes in healthy participants and the effect of a high-fat meal on the PK characteristics of HRS-7535. The trial is ongoing and no relevant clinical data are available.

The HRS-7535-201 study is a multicenter, randomized, double-blind, placebo-controlled, parallel-designed Phase II clinical study conducted in participants with type 2 diabetes, aiming to evaluate the efficacy and safety of HRS-7535 in patients with type 2 diabetes. As of May. 2024, preliminary results have been obtained. A total of 194 adult participants with type 2

diabetes received doses ranging from 15 mg to 90 mg. In the HRS-7535 15 mg group, 30 mg group, 60 mg group, 90 mg group, and placebo group, 31 participants (79.5%), 28 participants (71.8%), 29 participants (76.3%), 33 participants (84.6%), and 28 participants (71.8%) experienced TEAEs, with the majority being mild or moderate in severity. In the HRS-7535 15 mg group, 30 mg group, and 90 mg group, 3 participants (7.7%), 2 participants (5.1%), and 2 participants (5.1%) experienced TEAEs leading to treatment discontinuation, respectively. In the HRS-7535 15 mg group, 30 mg group, and 90 mg group, 3 participants (7.7%), 2 participants (5.1%), and 1 participant (2.6%) experienced TEAEs leading to withdrawal from the study, respectively. After 16 weeks of treatment, HbA1c levels significantly decreased in all HRS-7535 dose groups. The mean changes in HbA1c from baseline in the HRS-7535 15 mg group, 30 mg group, 60 mg group, 90 mg group, and placebo group were -1.19%, -1.59%, -1.82%, -1.64%, and -0.25%, respectively.

HRS-7535-202 is a multicenter, randomized, double-blind, placebo-controlled, parallel-group Phase II clinical study conducted in obese participants to evaluate the efficacy and safety of HRS-7535 in obese patients. The trial is ongoing and no relevant clinical data are available.

The HRS-7535-203 study is a multicenter, randomized, double-blind, placebo-controlled, parallel-designed Phase II clinical study conducted in participants with type 2 diabetic kidney disease, aiming to evaluate the efficacy and safety of HRS-7535 in participants with type 2 diabetic kidney disease. The trial is ongoing and no relevant clinical data are available.

Study HRS-7535-205 is a multicenter, randomized, double-blind, placebo-controlled, parallel-designed Phase II clinical study conducted in participants with type 2 diabetes inadequately controlled on metformin and SGLT2 inhibitors, aiming to evaluate the efficacy and safety of HRS-7535 in these participants. The trial is ongoing and no relevant clinical data are available.

2.2. Summary of benefits and risks

2.2.1. Benefit summary

Based on the clinical data of HRS-7535's other GLP-1 receptor agonists and its preclinical data, the potential benefits for participants with type 2 diabetes in the proposed study include: reduction in HbA1c, improvement in fasting plasma glucose, and weight loss. Additionally, all participants are expected to benefit from the investigator's customized health guidance, dietary and exercise management, and comprehensive health assessments during the study.

2.2.2. Risk summary and management measures

Based on the safety data as of Mar., 2024, a total of 52 healthy participants were exposed to single or multiple doses of HRS-7535, among which 38 participants (73.1%, 38/52) experienced HRS-7535-related adverse events (AEs). HRS-7535-related AEs with incidence ≥10% included nausea (57.7%), vomiting (48.1%), dizziness (26.9%), abdominal distension (21.2%), headache and protein urine present (19.2% each), and abdominal pain (13.5%). Among participants with poor renal function, 8 participants were exposed to a single dose of HRS-7535 30 mg, and no HRS-7535-related AEs occurred.

Based on the safety data as of May., 2024, a total of 155 participants with type 2 diabetes (HRS-7535-201 study) received HRS-7535 treatment, among which 83 participants (50.0%, 83/155) experienced HRS-7535-related AEs. HRS-7535-related AEs with incidence ≥5% included nausea (17.4%), decreased appetite (16.8%), lipase increased (9.7%), vomiting (9.0%), diarrhea (8.4%) and abdominal distension (5.2%).

Based on the clinical data of other GLP-1 receptor agonists, combined with the existing clinical and preclinical data of HRS-7535, the known potential risks may include but are not limited to the following:

（1） Gastrointestinal reactions, including: nausea, vomiting, loss of appetite, abdominal pain, diarrhea, etc.;

（2） Occurrence of hypoglycemic events;

（3） Amylase and lipase increased;

（4） Cholelithiasis;

（5） Heart rate increased.

2.2.3. Overall benefit-risk conclusion

In conclusion, HRS-7535 has a positive benefit-risk relationship, and the potential risks to participants participating in this trial are considered low and acceptable.

No important known risks or important potential risks have been identified for HRS-7535. Important risks will continue to be monitored in future studies, and risk minimization measures will be developed.

For detailed information on the known and expected benefits and risks of dapagliflozin, refer to the dapagliflozin (forxiga®) prescribing information [8].

3. Study objectives, endpoints, and estimands

3.1. Objectives and endpoints

Table 2 Study objectives and endpointsTable 2. Study Objectives and Endpoints2

Table 2. Study Objectives and Endpoints2

**Primary Objective**	**Primary Endpoint**
To confirm the non-inferiority of HRS-7535 to dapagliflozin in glycemic control efficacy among adult participants with type 2 diabetes who had inadequate glycemic control after metformin therapy.	The change in HbA1c from baseline at Week 32 of treatment with HRS-7535 compared to dapagliflozin.
**Secondary Objective**	**Secondary Endpoints**
In adult participants with type 2 diabetes who have poor glycemic control after metformin treatment:<br>Compare the efficacy of HRS-7535 and dapagliflozin after 32 weeks of treatment.	Key secondary efficacy endpoints:<br>HRS-7535 versus dapagliflozin at Week 32 of treatment:<br>• Proportion of participants achieving HbA1c < 7.0%<br>• Proportion of participants achieving HbA1c ≤6.5%<br>• Change in FPG from baseline;<br>• Percentage change from baseline in weight.
In adult participants with type 2 diabetes who have poor glycemic control after metformin therapy:<br>Comparison of other efficacy indicators between HRS-7535 and dapagliflozin at 32 weeks.	Supportive Secondary Efficacy Endpoint:<br>HRS-7535 versus dapagliflozin at Week 32 of treatment:<br>• Change from baseline in weight and waist circumference;<br>• Proportion of participants achieving HbA1c < 5.7%<br>• Changes from baseline in fasting serum insulin, fasting serum C-peptide, homeostasis model assessment of β-cell function index (HOMAβ), and insulin resistance index (HOMA-IR);<br>• Changes from baseline in 2-hour postprandial plasma glucose, 2-hour postprandial serum insulin, and 2-hour postprandial serum C-peptide;<br>• Proportion of participants requiring rescue treatment in each group<br>• Changes from baseline in 7-point SMBG profile.
In adult participants with type 2 diabetes who have poor glycemic control after metformin therapy:	Supportive Secondary Efficacy Endpoint:

Compare the efficacy of HRS-7535 and dapagliflozin after 52 weeks of treatment.	HRS-7535 versus dapagliflozin at Week 52 of treatment:<br>• Change from baseline in HbA1c;<br>• Change from baseline in FPG;<br>• Percentage change from baseline in weight;<br>• Change from baseline in weight and waist circumference;<br>• Proportion of participants achieving HbA1c < 7.0%<br>• Proportion of participants achieving HbA1c ≤6.5%<br>• Proportion of participants achieving HbA1c < 5.7%<br>• Changes from baseline in fasting serum insulin, fasting serum C-peptide, HOMAβ, and HOMA-IR;<br>• Changes from baseline in 2-hour postprandial plasma glucose, 2-hour postprandial serum insulin, and 2-hour postprandial serum C-peptide;<br>• Proportion of participants requiring rescue treatment in each group<br>• Changes from baseline in 7-point SMBG profile.
In adult participants with type 2 diabetes who have poor glycemic control after metformin therapy:<br>Evaluate the safety and tolerability of HRS-7535 and dapagliflozin at 32 weeks and 52 weeks.	HRS-7535 versus dapagliflozin at Week 32 and 52 of treatment:<br>• Adverse events, hypoglycemic events, laboratory tests, vital signs, physical examination, 12-lead electrocardiogram, diabetic retinopathy screening and assessment.
In adult participants with type 2 diabetes who have poor glycemic control after metformin therapy:<br>To evaluate the pharmacokinetics (PK) of HRS-7535.	• Plasma HRS-7535 concentration.
In adult participants with type 2 diabetes who have poor glycemic control after metformin therapy:<br>To assess patient reported outcomes in each treatment group.	• Change from baseline to Week 32 and Week 52 in Diabetes Treatment Satisfaction Questionnaire status version (DTSQs).
**Exploratory Objective**	**Exploratory endpoints**
In adult participants with type 2 diabetes who have poor glycemic control after metformin therapy:<br>Compare the efficacy of HRS-7535 and dapagliflozin in improving blood pressure, lipid metabolism, and renal function.	Compared with dapagliflozin, at Week 32 and Week 52 of treatment:<br>• Change from baseline in systolic pressure and diastolic pressure

Evaluate the PK characteristics of metformin when co-administered with HRS-7535.<br><br>Evaluate the PK characteristics of dapagliflozin.	• Percent change from baseline in Triglycerides (TG), total cholesterol (TC), low-density lipoprotein cholesterol (LDL-C) and high-density lipoprotein cholesterol (HDL-C);<br>• Percentage change from baseline in UACR.<br>• Concentrations of metformin and dapagliflozin in plasma.

Abbreviations: DTSQ = Diabetes Treatment Satisfaction Questionnaire; FPG = Fasting Plasma Glucose; HbA1c = Glycated Hemoglobin A1C; HDL-C = High-Density Lipoprotein Cholesterol; HOMA-IR = Homeostasis Model Assessment of Insulin Resistance; HOMA-β = Homeostasis Model Assessment of β-cell function; LDL-C = Low-Density Lipoprotein Cholesterol; PK = Pharmacokinetics; SMBG = Self-Monitoring of Blood Glucose; TC = Total Cholesterol; TG = Triglycerides; UACR = Urine Albumin/Creatinine Ratio.

3.2. Estimands

3.2.1. Primary estimand

The primary estimand is for the primary efficacy endpoint (change in HbA1c from baseline at Week 32 of treatment), with the following 5 attributes:

Table 3. Definition of Primary Estimand and Handling Strategies3

**Characteristics:**	**Definitions and Strategies**
Treatment	HRS-7535 30 mg, 60 mg, 90 mg or dapagliflozin 10 mg, orally administered, QD
Population	Participants with type 2 diabetes who have poor glycemic control despite being treated with metformin on the basis of regular diet and exercise control
Variable (endpoint)	Change from Baseline in HbA1c at Week 32
Intercurrent events and handling strategies	
Intercurrent event1: permanent discontinuation of study treatment	Hypothetical strategy: subsequently collected data will not be included in the statistical analysis
Intercurrent event 2: initiation of additional hypoglycemic drugs*	Hypothetical strategy: the subsequently collected data will not be included in the statistical analysis.
Summary at the Population Level	The mean difference in HbA1c change from baseline between the HRS-7535 and dapagliflozin groups after 32 weeks of treatment.

Abbreviation: HbA1c=glycated hemoglobin A1C; QD=once daily.
*Additional hypoglycemic drugs were defined as any hypoglycemic drugs used for more than 14 days cumulatively.

3.2.2. Supplementary estimand

The treatment strategy for both intercurrent event 1 and 2 of the supplementary estimand adopted the therapy strategy, i.e., data collected after the occurrence of intercurrent event 1 and 2 were included in the statistical analysis, while other attributes and concomitant event settings were consistent with the primary estimand.

3.2.3. Secondary estimand

For the secondary estimand targeting the secondary endpoints, the attributes of the 32-week secondary endpoints, intercurrent events and handling strategies are consistent with the primary estimand. The variable (endpoint) is changed to the corresponding endpoint, and the population-level summary is changed to the between-group difference for the corresponding endpoint. For the 52-week secondary endpoints, the intercurrent events are updated to events occurring before 52 weeks, with treatment (handling), population and strategies consistent with the primary estimand. The variable (endpoint) is changed to the corresponding endpoint, and the population-level summary is changed to the between-group difference for the corresponding endpoint.

4. Study design

4.1. Study design description

This trial is a multicenter, randomized, double-blind, positive-controlled, parallel-designed Phase III clinical trial, including a screening period of up to 2 weeks, a 3-week Run-in period, a 52-week double-blind treatment period, and a 2-week safety follow-up period. The trial population consists of adult participants with type 2 diabetes who have inadequate glycemic control after metformin treatment based on regular diet and exercise, with a planned enrollment of 800 participants.

Screening period (W-5 ~ W-3, up to 2 weeks)

After signing the Informed Consent Form, participants will undergo Screening Inspection, and those who meet the inclusion/exclusion criteria may enter the Run-in period.

Run-in period (W-3 ~ W0)

Participants who pass the screening will start continuous oral administration of single-blind Run-in period drug for 3 weeks QD from V2 (D-21).

The participant underwent baseline inspection at V3 (D-4), and the inspection at V4 (D1) assessed whether the participant met the randomization criteria. Participants who met the randomization criteria entered the 52-week double-blind treatment period.

Double-Blind Treatment Period (W1-W52)

Participants were randomized in a 1:1:1:1 ratio to the HRS-7535 30 mg group, 60 mg group, 90 mg group, and dapagliflozin group, with stratification based on baseline HbA1c values (≤8.5%, > 8.5%) measured by the central laboratory. Double-blind treatment period medication was initiated after randomization.

From the screening period to the end of the double-blind treatment period, all participants should receive stable-dose metformin as background therapy throughout. All participants should undergo assessments of efficacy and safety-related parameters at the protocol-specified visit time points during the double-blind treatment period.

Safety follow-up phase (W53 ~ W54)

All participants discontinued the double-blind treatment period medication, and the participants will complete the safety follow-up via telephone visit at Week 54.

4.2. Trial design basis

Metformin is the first-line hypoglycemic drug recommended by current clinical guidelines for the treatment of type 2 diabetes. However, many patients fail to achieve glycemic targets with metformin monotherapy and require combination therapy with other hypoglycemic agents. HRS-7535 is an oral small molecule GLP-1 receptor agonist under development for the treatment of type 2 diabetes and weight loss. Preliminary clinical trials suggest that HRS-7535 has good hypoglycemic effects. In combination with metformin, it is expected to become a new treatment option for patients with type 2 diabetes who have inadequate glycemic control after metformin therapy. The objective of this trial is to evaluate the efficacy and safety of HRS-7535 compared with dapagliflozin in adult participants with type 2 diabetes who have inadequate glycemic control with metformin therapy.

The trial design referred to the "Clinical Research and Development Guidelines for Drugs for Adult Type 2 Diabetes Mellitus", adopting a multicenter, randomized, double-blind, positive-controlled, parallel design to eliminate selection bias and information bias. The total trial duration was approximately 59 weeks, including a 52-week double-blind treatment period, which could meet the needs for evaluating the persistence of efficacy and observing long-term safety. This trial set up a Run-in period before randomization to exclude participants with poor compliance or poor glycemic control before randomization. The primary endpoint was selected as the change in HbA1c from baseline at Week 32. The change in HbA1c from baseline remains a recognized surrogate endpoint in current clinical trials of glucose-lowering drugs, serving as the primary efficacy endpoint in key studies to assess the glucose-lowering effect of the drug. Key confirmatory trials typically use the 6-month time point after stable dose treatment as the primary efficacy assessment time point.

In addition, to better ensure the safety of participants, the following measures were taken in this trial: 1) All participants could continue their original metformin treatment regimen as background therapy, but to avoid affecting efficacy evaluation, the metformin dosage should

remain unchanged during the trial; 2) The trial set up rescue treatment, allowing participants who met the rescue treatment criteria to receive other antidiabetic drugs, and provided all participants entering the double-blind treatment period with glucose meters for convenient self-monitoring of blood glucose, minimizing the risk of worsening glycemic control; 3) The control group in this trial was an active control, avoiding ethical issues arising from prolonged exposure to placebo; 4) Participants could withdraw from the trial at any time, and investigators could also discontinue participants' participation in the trial.

4.3. Access to study intervention after end of study

The investigational product will only be provided to the participants during this trial and will no longer be supplied after the trial ends.

4.4. Definition of trial initiation and completion

The start of the trial is defined as follows:

First participant first visit (FSFV) (ie, when the first participant signs the informed consent form [ICF]).

The definition of the end of the trial is as follows:

Last participant last visit (LSLV) (i.e., the last visit of the last participant).

5. Study population

5.1. Selection of trial population

Age ≥18 years and ≤75 years, adult participants with type 2 diabetes with poor glycemic control after metformin treatment on the basis of regular diet and exercise control.

5.2. Selection of trial population and basis

Based on the objective of this trial, adult participants with type 2 diabetes who had poor glycemic control despite metformin treatment on the basis of regular diet and exercise control were selected as the trial population. Enrolled participants were required to have been treated with a stable dosage of metformin for type 2 diabetes for ≥8 weeks at a daily dosage of ≥1500 mg or a maximum tolerated dose of ≥1000 mg daily (the metformin dosage met the requirements of the package insert), and the metformin dosage was to remain unchanged during the trial to avoid affecting the evaluation of efficacy. An HbA1c range of 7.5% to 11.0% was selected as the cutoff value to include type 2 diabetes patients who required additional glucose-lowering drug therapy, while avoiding the inclusion of patients with poorer glycemic control that could increase safety risks.

Considering the adverse reactions of HRS-7535 and other similar drugs, participants with a medical history of pancreatitis, thyroid disorder, or significant gastrointestinal disease were excluded; considering the usage restrictions of metformin and dapagliflozin, participants with poor renal function, a medical history of diabetic ketoacidosis, or urinary tract infection were excluded. In addition, participants with a medical history of severe diseases (such as neoplasm malignant or cardiovascular disorder) were also excluded to avoid increasing safety risks and affecting safety evaluation.

5.3. Inclusion criteria

Participants must meet all the following criteria to be enrolled in this trial:

1. Male or female, aged between 18 and 75 years (inclusive) on the day of signing the ICF;
2. Type 2 diabetes mellitus with a medical history of ≥3 months was diagnosed according to the Chinese Guidelines for the Prevention and Treatment of Type 2 Diabetes (2020 Edition) at screening.
3. HbA1c levels between 7.5% and 11.0% (inclusive) at screening (as tested by a local laboratory);
4. At screening, stable treatment for type 2 diabetes for ≥8 weeks, with no planned changes to the corresponding treatment regimen during the entire trial period. At screening, on the basis of regular diet and exercise control, the treatment regimen was stable treatment with metformin as monotherapy for ≥8 weeks, with a daily dose of ≥1500 mg or maximum tolerated dose of ≥1000 mg daily (metformin dose met the requirements of the package insert);
5. At screening, 19.0 kg/m2 ≤ body mass index (BMI) ≤ 40.0 kg/m2;
6. Voluntarily sign the ICF prior to the trial, fully understand the content, process, and possible adverse reactions of the trial, and are able and willing to complete the study in accordance with protocol requirements (e.g., self-monitoring of blood glucose and recording participant diary);
7. From signing the Informed Consent Form until 2 weeks after the last dose, participants have no plans for pregnancy and are willing to use highly effective contraception as specified in the protocol.

5.4. Exclusion criteria

Participants who meet any of the following criteria could not be enrolled in this trial:

Those with the following inspection abnormalities at screening:

1. Presence of the following laboratory inspection abnormalities at screening or randomization:

   - Fasting venous glucose > 15 mmol/L;
   - Alanine aminotransferase (ALT) or aspartate aminotransferase (AST) > 2 × upper limit of normal (ULN);
   - Total bilirubin (TBIL) > 1.5 × ULN;
   - Amylase and/or lipase > 3 × ULN;
   - Triglycerides (TG) under fasting conditions > 11.3 mmol/L;
   - Calcitonin ≥50 ng/L;
   - Estimated glomerular filtration rate (eGFR) < 45 mL/min/1.73 m2 (calculated using the chronic kidney disease epidemiology collaboration [CKD-EPI] equation, see Section 13.5);13.5
   - Urinary albumin creatinine ratio (UACR) ≥1000 mg/g;
   - Positive Hepatitis B Virus Surface Antigen (HBsAg) and HBV-DNA > ULN;

2. Clinically significant abnormalities in ECG results at screening or randomization that could affect participant safety, including but not limited to supraventricular tachycardia, atrial fibrillation, atrial flutter, second- or third-degree atrioventricular block, etc.;
3. The electrocardiogram inspection results during screening or randomization indicated a heart rate > 100 bpm or QTcF > 480 ms. If the heart rate or QTcF is abnormal, two additional electrocardiograms could be performed to obtain the mean of three values;
4. Severe uncontrolled hypertension (systolic pressure ≥160 mmHg or diastolic pressure ≥100 mmHg) at screening or randomization;

Those with the following diseases or medical history prior to screening:

5. Diagnosed or suspected type 1 diabetes mellitus, special type of diabetes mellitus or secondary diabetes mellitus;
6. Acute complications of diabetes (diabetic ketoacidosis, lactic acidosis, hyperglycemic hyperosmolar state, etc.) within 6 months prior to screening;
7. Severe hypoglycemic episodes or recurrent hypoglycemic episodes within 6 months prior to screening (≥ 3 hypoglycemic episodes within a week, or other hypoglycemic episodes as determined by the investigator);
8. Presence of proliferative retinopathy or maculopathy, or painful diabetic neuropathy requiring acute treatment, diabetic foot ulcer, or intermittent claudication at screening;

9. Within 6 months prior to screening, participants with known clinically significant diseases including but not limited to neurological, mental, cardiovascular, endocrine, gastrointestinal, respiratory, urinary, hematological, immune system and other diseases (except for type 2 diabetes related conditions) that may interfere with the study results or pose additional risks to the administration of the study drug as determined by the investigator;

10. Participants with a personal or family history of medullary thyroid carcinoma (MTC) or multiple endocrine neoplasia type 2 (MEN 2);

11. Participants with prior or known history of acute or chronic pancreatitis, pancreatic injury, acute cholecystitis, or symptomatic gallbladder disease requiring treatment, except for those who have undergone cholecystectomy and are deemed eligible for enrollment by the investigator;

12. Prior history of significant gastrointestinal diseases (such as gastric outlet obstruction, inflammatory bowel disease, active ulcer, etc.), or those who have undergone gastrointestinal surgery (except for gastrointestinal polypectomy and appendectomy), or those who have taken drugs that directly affect gastrointestinal motility for a long time;

13. Hyperthyroidism medical history before screening, or hyperthyroidism at screening, or other thyroid disorders not stably controlled (except for participants with thyroid hormone Alternative treatment regimen stable for at least 8 weeks and no plan to change the treatment regimen during the study);

14. History of severe cardiovascular and cerebrovascular diseases within 6 months prior to screening, including but not limited to: congestive heart failure (New York Heart Association [NYHA] Grade 3-4), unstable angina, stroke, myocardial infarction, severe arrhythmia, or coronary revascularization; or planned to undergo coronary, carotid or peripheral artery revascularization at screening;

15. Participants with known or suspected depression, bipolar disorder, suicidal tendency, schizophrenia or other severe mental diseases; or those who are mentally incapacitated or have language disorders, unable to fully understand the protocol or unwilling to cooperate with the study site staff;

16. History of neoplasm malignant within 5 years prior to screening, except for cured local cancer, such as: local skin basal cell carcinoma, cervical carcinoma in situ, and carcinoma in situ of prostate;

17. At screening or randomization, the participant was in a state of urinary tract infection or genital infection, or had a medical history of urinary tract infection or genital infection caused by the use of SGLT2 inhibitors.

Those who have received any of the following drugs or treatments prior to screening:

18. Use of GLP-1 receptor agonist drugs (including multi-target GLP-1 receptor agonist drugs) within 3 months before screening;

19. Use of systemic glucocorticoids (excluding topical, inhaled, intraocular, or intranasal administration) for a cumulative duration of < 14 days within 3 months prior to the screening;

20. Use of tricyclic antidepressants, atypical antipsychotics and mood stabilizers (such as imipramine, chlorpromazine, amitriptyline, mirtazapine, clozapine, olanzapine, paroxetine, phenelzine, thioridazine, valproic acid and its derivatives, lithium, etc.) within 3 months before screening;

21. Weight loss therapy within 3 months prior to screening, including intensive diet or exercise therapy for weight loss, drug therapy, surgical treatment (except for liposuction history more than 1 year), health products or other special therapies;

Those with any of the following conditions:

22. Self-reported weight change exceeding ±5% within 3 months during screening or randomization, or planned intensive diet or exercise therapy for weight loss during the trial (except for diabetes diet and exercise control measures);

23. those who have participated in any clinical trial of drugs or medical devices within 3 months prior to screening or still within 5 half-lives of the study drug (whichever is longer). Participation in a clinical trial is defined as signing informed consent and having received the Investigational Product (including Placebo) or investigational medical device;

24. Known or suspected hypersensitivity to the study drug or its components

25. Known or suspected history of drug or alcohol abuse, and judged by the Investigator as unsuitable for participation in this clinical trial;

26. Participants with a history of blood donation or blood loss of ≥ 400 mL within 3 months prior to screening, or who have received blood transfusion;

27. Female participants who are pregnant, lactating, or planning to become pregnant during the study period

28. Intolerance to venipuncture or needle phobia.

29. Any condition determined by the investigator to be unsuitable to participate in this clinical trial.

5.5. Lifestyle requirements

During this study, starting from the Screening Visit, the investigator or designated personnel will provide guidance on healthy diet and lifestyle for diabetes to the participant at each Visit. The participant should follow the corresponding guidance to manage their own diet and lifestyle. Any violations of the guidance should be recorded on the participant diary card, but these violations will not be considered as Protocol deviation.

In addition, the following matters should be noted before each blood collection or inspection:

- Not consume products containing caffeine or xanthine (such as coffee, tea, cola drinks, and chocolate) within 24 hours prior to each blood collection.
- Abstain from alcohol for 24 hours before each blood collection.
- Avoid strenuous exercise for 24 hours before each blood collection for laboratory inspection.

Female participants of childbearing potential and male participants whose female partners are of childbearing potential must use contraceptive measures (see Section 13.1 for details).13.1

5.6. Screen failure and randomization failure

The following participants meet the definition of screen failure:

- Participants who signed the informed consent failed to meet the screening criteria;
- Participants who signed the informed consent did not continue to participate in the trial before confirming whether they met the screening criteria.

Re-screening of participants who have previously failed screening is allowed, provided that each participant is allowed only once throughout the course of the trial. They must sign the ICF again, re-register and obtain a new participant number at the re-screening.

The following participants met the definition of randomization failure:

- Screening Successful Participants, Failed to Meet Randomization Criteria
- Participants who passed the screening did not continue to participate in the trial before confirming whether they met the randomization criteria;
- Participants who passed the screening and met the randomization criteria but did not participate in randomization.

The investigator should inform the participants who failed randomization to discontinue the drugs of Run-in period, return the packaging of the drugs of Run-in period and the remaining

drugs, and provide the participants with reasonable clinical treatment measures. Participants who failed randomization are not eligible for rescreening.

The necessary information of screening/randomization failure should be recorded to ensure the transparency of the report, to meet the requirements of the Consolidated Standards of Reporting Trials (CONSORT), and to respond to the queries of the regulatory authorities. These information include demographics, informed consent, reason for screening/randomization failure, inclusion and exclusion criteria, and any SAE.

6. Trial interventions and concomitant treatment

6.1. Information of investigational intervention

The investigational products in this trial were all investigational medicinal products, including: HRS-7535/placebo and dapagliflozin (Forxiga®)/placebo. The investigational products were provided by the sponsor, uniformly packaged and tested to be qualified. The relevant information is shown in Table 4.Table 4. Investigational Product Information4

Metformin was used as background treatment and was self-provided by the participant.

Table 4. Investigational Product Information4

Trade Name	HRS-7535-	Dapagliflozin (Forxiga®)	HRS-7535 placebo	Dapagliflozin Placebo
**Type**	Study Drug	Positive Comparator Product	Placebo	Placebo
**Dosage Form**	Tablet	Tablet	Tablet	Tablet
**Strength**	30 mg, 60 mg	10 mg	30 mg, 60 mg	10 mg
**The storage conditions are**	Store sealed below 25°C	Sealed, at temperatures not exceeding 30°C	Store sealed below 25°C	Sealed, at temperatures not exceeding 30°C
**Packaging**	Hard aluminum-aluminum blister packaging	Hard aluminum-aluminum blister packaging	Hard aluminum-aluminum blister packaging	Hard aluminum-aluminum blister packaging
**Shelf Life**	See the investigational product packaging for details.	36 months	See the investigational product packaging for details.	See the investigational product packaging for details.
**Manufacturer**	Shandong Suncadia Medicine Co., Ltd.	AstraZeneca Pharmaceuticals LP	Shandong Suncadia Medicine Co., Ltd.	Shandong Suncadia Medicine Co., Ltd.

6.2. Basis for selection of trial intervention

GLP-1 receptor agonists are a new class of oral antidiabetic drugs that could effectively reduce blood glucose, partially restore pancreatic β-cell function, reduce body weight, improve lipid

profiles and lower blood pressure, with proven protective effects on the cardiovascular system and kidneys. HRS-7535 is an oral small molecule GLP-1 receptor agonist with convenient administration, which is expected to become a new option for the treatment of type 2 diabetes. SGLT-2 inhibitor is also a new type of oral antidiabetic drug, which has definite protective effects on the cardiovascular system and kidneys in addition to its hypoglycemic effect. Dapagliflozin is an approved SGLT-2 inhibitor.

6.2.1. Rationale for dose selection

In this study, 30 mg, 60 mg and 90 mg were planned to be selected as the target dosages.

Based on the results of previous clinical studies, HRS-7535 demonstrated good safety and tolerability at doses ranging from 15 - 90 mg.

Based on the existing clinical study data, a population pharmacokinetics (PopPK) model was constructed to simulate and calculate the AUCss of participants, and to evaluate the relationship between AUCss and HbA1c as well as body weight. The analysis results showed that with the increase of average exposure to HRS-7535, both HbA1c and body weight exhibited a decreasing trend. Among them, HbA1c began to show a more obvious reduction effect at the dosage of 30 mg, and basically reached the drug effect plateau at the dosage of 60 mg. With the increase of exposure, the weight loss effect increased, and a more obvious effect was observed at the dosage of 90 mg.

Based on the Safety and tolerability data of HRS-7535-201 study, the impact of different titration starting doses and dose escalation amplitudes on tolerability was evaluated to develop the dosing regimen. According to the study results, a titration regimen with 30 mg as the starting dose and a 30 mg increase every 4 weeks could achieve sufficient tolerability to gastrointestinal Adverse events.

The dosage of the comparator dapagliflozin was 10 mg QD, which is the commonly used and clinically approved dose.

6.3. Dosage and administration

6.3.1. Dosing regimen

<u>Run-in period (single-blind)</u>

All participants received HRS-7535 placebo (1 tablet of 30 mg and 1 tablet of 60 mg) and dapagliflozin placebo (1 tablet of 10 mg), QD. The investigational product was allowed to be

taken on the day of V2 or the next day, with the administration day as day 1 of the Run-in period.

Double-blind treatment period

Approximately 800 participants will be randomized in a 1:1:1:1 ratio to the HRS-7535 30 mg group, 60 mg group, 90 mg group, and dapagliflozin group, with stratification based on baseline HbA1c values (≤8.5%, > 8.5%) measured by the central laboratory. The double-blind treatment period medication will be initiated after randomization. The investigational product may be administered on the day of randomization or the following day, with the dosing day designated as Day 1 of the double-blind treatment period.

The dosing regimens of each group are as follows:

- HRS-7535 30 mg group: HRS-7535 30 mg, administered orally, QD.
- HRS-7535 60 mg group: dose titration was adopted. The participants received HRS-7535 30 mg, orally administered, QD for the first 4 weeks (D1 to D28); after Week 5 (after D29), they received HRS-7535 60 mg, orally administered, QD.
- HRS-7535 90 mg group: dose titration was adopted. For the first 4 weeks (D1 to D28), HRS-7535 30 mg was administered orally, QD; from Week 5 to Week 8 (D29 to D56), HRS-7535 60 mg was administered orally, QD; after Week 9 (after D57), HRS-7535 90 mg was administered orally, QD.
- Dapagliflozin group: dapagliflozin (trade name: Forxiga®), 10 mg, orally administered, QD.

From the screening period to the end of the double-blind treatment period, all participants should receive stable dosage of metformin as background therapy throughout the course.

Definition of stable dose: except for medically necessary reasons, the daily dosage of metformin should not be changed.

To ensure the implementation of blinding, the drug dosage and corresponding number of tablets for each group during the Run-in period and double-blind treatment period are as follows:

Table 5. Drug Dosage and Corresponding Number of Tablets During the Trial5

Dose Level / Investigational Product	Run-in period:	Double-blind treatment period			
	All Participants	HRS-7535 30 mg	HRS-7535 60 mg	HRS-7535 90 mg	dapagliflozin group
HRS-7535 30 mg	0	1	0	1	0
HRS-7535 30 mg placebo	1	0	1	0	1
HRS-7535 60 mg	0	0	1	1	0

Dose Level / Investigational Product	Run-in period:	Double-blind treatment period			
	All Participants	HRS-7535 30 mg	HRS-7535 60 mg	HRS-7535 90 mg	dapagliflozin group
HRS-7535 60 mg placebo	1	1	0	0	1
Dapagliflozin Tablets 10 mg	0	0	0	0	1
Dapagliflozin Tablets 10 mg Placebo	1	1	1	1	0

6.3.2. Medication guidance

The participant should take the investigational product once daily with water, swallow it whole, and not hold or chew the drug before swallowing. It could be taken before or after meals. It is recommended that the participant take the medication at approximately the same time each day and not exceed the dosage specified in the protocol.

Missed dose guidance: if a participant misses a dose of the Investigational Product on any day, they should continue taking the Investigational Product as prescribed on the following day without making up for the missed dose.

Guidance for vomiting after medication: if a participant vomits after taking the Investigational Product, regardless of whether the vomitus contains the Investigational Product, they should not take an additional dose but should continue with the next day’s Investigational Product as per the protocol.

Guidance for overdosing: if the participant takes more than the prescribed amount of the investigational product, they must inform the investigator. The investigator and the sponsor will jointly decide whether to adjust the dosage of the medication subsequently.

During the single-blind Run-in period, the participant received the Run-in period investigational product HRS-7535 placebo, dapagliflozin placebo and background medication metformin (self-provided). In order to not affect the participant's medication compliance, the investigator must comply with the requirements of the single-blind design and not inform the participant of the specific components of the Run-in period investigational product.

6.3.3. Dose modification

Except for the provisions in Section 6.3.3.1 and Section 6.3.3.2, dose modification of HRS-7535/Placebo is not allowed during the trial.6.3.3.16.3.3.2

6.3.3.1. Management of gastrointestinal symptoms

During the trial, if intolerable gastrointestinal symptoms occur, the investigator should implement the following steps:

1. It is recommended that the participant take the medication after meals and eat small, frequent meals. e.g., changing from three to four or more meals a day, and to stop eating immediately upon feeling full.
2. If dietary changes fail to effectively improve gastrointestinal symptoms, symptomatic treatment with antiemetics, gastroprotective agents, or antidiarrheals may be administered based on symptoms and needs.
3. When the participant continues to have intolerable gastrointestinal symptoms after the above management measures, the investigator may adjust the dosage of the study drug according to the following rules:

- Within 1–4 weeks post-randomization (V4–V6, excluding the day of V6 visit), the study treatment was temporarily discontinued for 1–2 days, then continued as planned.
- Within 5–8 weeks post-randomization (V6–V7, excluding the day of V7 visit): the study treatment was temporarily discontinued for 1–2 days, then continued as planned. Participants who still could not tolerate it returned to the study site to consider de-escalating to the next lower dose.
- Between 9 to 52 weeks post-randomization (V7 to V15): after study treatment suspended for 1 to 2 days, continue taking partial drugs from the Packaging under the guidance of the Investigator (temporarily discontinue HRS-7535/Placebo 30 mg) to complete Dose modification. Participants who still could not tolerate should return to the Study site to consider de-escalating to the next lower dose.

4. When intolerable GI symptoms persist, despite the above measures:

- If the participant had persistent intolerable gastrointestinal symptoms, the participants should be permanently discontinued from the Investigational Products
- For participants who did not receive de-escalation, if the gastrointestinal symptoms are tolerable or resolved, they may attempt to take all the medications in the drug packaging again to re-escalate to the highest maintenance dose at randomization; or they may maintain the current dose-adjusted treatment regimen until the end of the double-blind treatment period.

- Participants who received de-escalation will be adjusted to the lowest initial dose at randomization until the end of the double-blind treatment period. The results of dose modifications at each stage are summarized as follows:

**Participant Group**	**Discontinuation of HRS-7535/placebo 30 mg.<br>The dosage will be adjusted to**	**After returning to the study site for de-escalating,<br>The dosage will be adjusted to**
30 mg	Placebo	30 mg
60 mg	60 mg	30 mg
90 mg	60 mg	30 mg

The above measures are general principles. The investigator may actively contact the sponsor based on the actual situation to jointly develop an individualized dose modification strategy under the premise of maintaining blinding, in order to fully ensure the minimization of gastrointestinal system symptom risks for the participant.

6.3.3.2. Temporary dose interruption

If the study drug dosage is interrupted for reasons other than those specified in Section 6.3.3.1, the investigator should refer to the table below to guide the participant to resume the corresponding study treatment as soon as possible, provided that the safety and tolerability of the participant are acceptable.6.3.3.1

<table>
<tr><th>Participant visit</th><th>Number of Dose Interruption Days</th><th>Dose modification</th></tr>
<tr><td>Within 1 to 8 weeks after randomization (V4 to V7, excluding the day of V7 visit)</td><td>Any day</td><td>Continued to receive the planned study treatment as per the protocol</td></tr>
<tr><td rowspan="3">Within 9 to 52 weeks after randomization (V7, excluding the day of V7 visit)</td><td>Day -3</td><td>Continued to receive the planned study treatment as per the protocol</td></tr>
<tr><td>3 to 5 days</td><td>Select one dose modification strategy based on the participant's previous tolerance:
（1） Continue to receive the current study treatment as per the protocol;
（2） Take part of the drugs in the packaging (suspend HRS-7535/Placebo 30 mg). If the gastrointestinal symptoms are tolerable or subside, take all the drugs in the packaging to re-escalate to the highest maintenance dosage at randomization.</td></tr>
<tr><td>≥6 days</td><td>Take part of the drugs in the packaging (suspend HRS-7535/Placebo 30 mg). If the gastrointestinal symptoms are tolerable or subside, take all the drugs in the packaging. Re-escalate to the highest maintenance dosage at randomization.</td></tr>
</table>

If a participant with temporary dose interruption experiences gastrointestinal symptoms while receiving the aforementioned dose modification and treatment, please refer to Section 6.3.3.1 for gastrointestinal symptoms management and dose modification.6.3.3.1

The above measures are general principles. The investigator may actively contact the Sponsor according to the actual situation, and jointly develop an individualized dose modification strategy under the premise of maintaining blinding, to fully ensure the minimization of risk for the participant.

6.4. Treatment of overdose

For the definition of overdose, please refer to Section 12.1. In the event of a drug overdose, it is recommended to monitor the participant for any symptoms or signs of adverse reactions and immediately provide appropriate symptomatic treatment.12.1

6.5. Medicinal product preparation, handling, storage and accountability

6.5.1. Preparation of medicinal product

All Investigational Products will be prepared by qualified personnel according to the drug manual and dispensed to participants in a Blinding manner.

6.5.2. Medicinal product handling and storage

The investigator or his/her authorized representative (e.g., a pharmacist) will ensure that all investigational products are stored in a secure and access-controlled area conforming to storage conditions, and their storage complies with applicable regulatory requirements.

Site systems must be capable of measuring and recording (e.g. via a log) at least daily minimum and maximum temperatures for all site storage locations (including frozen, refrigerated and/or room temperature products, as applicable). This data must be collected continuously from the time of IMP receipt through the duration of the trial. Even with a continuous monitoring system in place, there should be logs or study site procedures to ensure proactive assessment of deviations. This is to ensure that no deviations have occurred since the last assessment by checking the minimum and maximum temperatures each working day, and to ensure that after normal operation is restored, the study site could store or review the minimum/maximum temperatures for all non-working days. Temperature monitoring equipment and storage devices shall be checked regularly for operation (if applicable) to ensure they are in proper working condition.

Any deviations from the product label conditions should be reported promptly once detected. The study site should take active measures to place the product under the storage conditions

described on the label as soon as possible, and report the temperature deviation and measures taken to the sponsor.

The Investigational Product affected by the temperature deviation should be isolated temporarily in the environment meeting the storage conditions, and could only be used after receiving the permission from the Sponsor. If the Sponsor allows the continued use of the Investigational Product after the temperature deviation, this deviation will not be recorded as a Protocol deviation. However, if the Investigational Product is used before obtaining the Sponsor's permission, it will be considered a Protocol deviation. The Sponsor will provide the Study site with detailed steps to report the temperature deviation.

Storage requirements for HRS-7535/Placebo: sealed, stored at no more than 25℃. Storage requirements for Dapagliflozin Tablets/Placebo: sealed, stored at no more than 30℃. For details, refer to the Pharmacy Manual.

The study site should store all medicinal products in accordance with the storage requirements from the date of receipt of the investigational product to the period of drug recovery. The study institution should keep the investigational product in accordance with the relevant regulatory requirements, and should not transfer the investigational product to any non-clinical trial participants, unauthorized personnel and third parties.

For the study in which participants are allowed to take medicinal products home, the staff of the study site will guide the participants on the correct way to store medicinal products.

6.5.3. Management of medicinal products

Qualified study team personnel will dispense the investigational product according to the quantity specified in the process each time. Throughout the trial, the participant should store the drugs as instructed and must return all unused drugs and packaging to the investigator at each visit and at the end of the trial.

The study site must maintain records of the Investigational Product supply, including receipt, distribution, use, recovery, loss, or other disposition. Under no circumstances shall the Investigator provide the Investigational Product to a third party, allow its use for purposes other than those specified in the clinical study protocol, or dispose of the Investigational Product in any other manner.

6.5.3.1. Medicinal product destruction

If applicable, the sponsor or their authorized personnel will instruct about how to destroy unused and recovered investigational products. If the study site is authorized to destroy the

investigational product, the investigator must ensure that the destruction of the investigational product complies with applicable environmental regulations, institutional policies and other provisions, and provide relevant procedures for destruction. All the destructions should be well recorded.

6.6. Participant assignment, randomization and blinding

6.6.1. Participant assignment

After the participant signs the written Informed Consent Form, a participant coding will be automatically assigned, which will be used for all Electronic Case Report Forms (eCRFs) and study documentation. Participants who complete all screening procedures and meet the inclusion/exclusion criteria will be formally enrolled in this trial.

Participants who successfully pass the screening will receive a 3-week Run-in period (single-blind) drug therapy. Baseline inspection will be conducted at V3 (D-4), and V4 (d1) will assess whether the participants meet the randomization criteria. Participants who meet the randomization criteria will be randomized, obtain a randomization number, and receive one or more drug numbers according to the randomization-related protocol, followed by the corresponding double-blind investigational product during the treatment period.

Randomized participants who withdraw from the clinical trial for any reason, regardless of whether the investigational product is used or not, will retain their randomization number, and the participant will not be allowed to enter the trial again.

Before the start of the study, the sponsor will provide HRS-7535, HRS-7535 placebo, Dapagliflozin Tablets and Dapagliflozin Tablets placebo with the existing drug number to the study site. Once participants are screened and enrolled, they are randomized by the randomization system and provided with the double-blind investigational product with the corresponding drug number.

6.6.2. Randomization

The study adopted a stratified block randomization method, with baseline HbA1c values (≤8.5%, > 8.5%) measured by the central laboratory as stratification factors, and eligible participants were randomly assigned to different groups in a 1:1:1:1 ratio.

The randomization specialist used SAS software to complete the generation of the randomization table and Medicinal Product number table, randomized participants who met the randomization criteria using the Interactive Web Response System (IWRS), and gave the corresponding group of Medicinal Product treatment according to the enrollment results. A

randomized participant is defined as a participant who is registered and assigned with a randomized treatment group from the IWRS, regardless whether the treatment is used or not. A participant could not be randomized more than once in this study.

6.6.2.1. Randomization criteria

The participant was randomized after reconfirming the following randomization criteria based on the V3 inspection results.
Participants who failed randomization in this study are not eligible for rescreening.

1. HbA1c 7.5% - 11.0% (central laboratory results);
2. Glucose under fasting conditions ≤15.0 mmol/L (central laboratory results);
3. Laboratory tests, vital signs, body weight, BMI, electrocardiogram, pregnancy status, etc. met the inclusion criteria and did not meet the exclusion criteria;
4. Medication compliance ≥ 80% and ≤ 120% during the Run-in period;
5. The metformin treatment regimen remained stable during the Phase-in period;
6. No hypoglycemic drugs other than those specified in the protocol were used during the Run-in period;
7. No condition considered by the investigator as inappropriate for participation in this clinical trial occurred during the Run-in period.

6.6.3. Blinding and unblinding

The study adopted the stratified block randomization method to randomize participants into 4 groups in a 1:1:1:1 ratio. The randomization specialist used SAS statistical software to complete the generation of the randomization table and drug coding list. Under the guidance of the blinding personnel, the sponsor’s production department packaged the medicinal products according to the drug number. The labels of the investigational product and placebo were completely identical except for the drug number. Blinding must be maintained throughout participant enrollment and administration, documentation and evaluation of study results, inspection of the study process, and data management. The participants, investigators, sponsor's researchers participating in the clinical evaluation, and the clinical research associate (CRA) participating in the study were not aware of the treatment allocation. The third-party laboratory testing personnel were unblinded and did not perform PK testing for the positive control group (dapagliflozin group). To control any potential study bias, the blind codes will

be properly kept as confidential information until the study database is locked so as to prevent disclosure to the investigators and study staff in direct contact with the investigators.

In the event of the following emergencies: (1) SAE; (2) the participant requires emergency resuscitation, the investigator shall decide whether to perform emergency unblinding. If possible, before emergency unblinding of the investigational product, the investigator should first contact the sponsor to discuss whether unblinding is required. If the sponsor is not informed in advance, the investigator shall notify the sponsor as soon as possible after unblinding.

After data verification in a blinded state and data lock, the statistician requests unblinding with the randomization specialist. This involves disclosing the participant's group assignment and medication code to the statistician for statistical analysis of all data.

6.7. Compliance with trial intervention

Compliance with trial intervention = actual number of tablets taken/theoretical number of tablets to be taken × 100%, compliance with HRS-7535/placebo and dapagliflozin/placebo should be calculated separately. Compliance of participants during the Run-in period, compliance between visits during the double-blind treatment period (excluding telephone visits), and overall compliance during the double-blind treatment period should all be ≥ 80% and ≤ 120%.

Throughout the study, the investigator should remind the participants to comply with the protocol and requirements to ensure participant compliance. At each study visit or at the end of the study, participants should return all partially used and unused investigational products and packaging to the study site, where the returned investigational products and packaging will be recorded and archived.

At each visit, the investigator should assess the participant's intake of the investigational product based on drug accountability. The investigator (or designee) will document in detail the dispensing and recovery of the investigational product at each visit and the reason for poor compliance in the source documents (if applicable).

If the participant is found to have poor compliance, the investigator must inform the participant of the importance of using the investigational product according to the protocol. Participants with severely poor compliance, as determined by the investigator, may be withdrawn from the study early.

6.8. Concomitant treatment

Concomitant treatment was given for the interest of the participant as determined by the investigator.

All concomitant medications, blood products, and non-drug interventions administered from the screening period through the end of study visits must be documented in the eCRF.

In principle, participants should avoid receiving any concomitant treatment during the trial. If a participant experiences an AE, the investigator may give appropriate treatment for the benefit of the participant; before using the treatment, if possible, the investigator should discuss with the sponsor to determine the concomitant treatment to be used.

6.8.1. Medications for gastrointestinal symptoms

If a participant experiences gastrointestinal symptoms (such as nausea, vomiting, and diarrhea) considered related to the study drug, it is advisable for the participant to manage symptoms by resting, taking the drug after meals, and eating smaller, more frequent meals. If the participant's gastrointestinal symptoms do not improve with the aforementioned treatments, the investigator may decide to use symptomatic treatments such as antiemetics, gastroprotective agents, or antidiarrheals, and must inform the sponsor and record this information in the eCRF, as detailed in Section 6.3.3.1.6.3.3.1

6.8.2. Prohibited concomitant treatment

From the screening period to the end of the safety follow-up phase, participants could use drugs for the treatment of underlying diseases and AEs, and in principle, no other drugs are recommended.

Participants were prohibited from using the following drugs from the screening period to the last dose of investigational product.

- Any glucose-lowering medications or treatments other than the Investigational Product, metformin, and rescue treatment medications;

Note:

- *Rescue treatment is prohibited from using: drugs containing GLP-1 receptor agonist components (including multi-Target drugs), DPP-4 inhibitors, SGLT2 inhibitors.*
- *In certain clinical situations (e.g., elective surgery, hospitalization, hypertonic state), short-term use of insulin with a cumulative duration of < 14 days is*

allowed, and when reported in the eCRF, this insulin use must be distinguished from insulin use as rescue treatment.

- Any medication or treatment that in the investigator's opinion could significantly interfere with glucose metabolism, such as any systemic corticosteroid therapy lasting ≥14 days (excluding topical, inhaled, intraocular, intranasal, or intra-articular administration).
- Drugs approved for weight loss, such as orlistat, liraglutide, benaglutide, etc.;
- Certain other drugs that may cause increased blood sugar, including but not limited to phenothiazine, estrogen, phenytoin, nicotinic acid, and isoniazid.
- Growth hormone or somatostatin analogues;
- Other incretin drugs;
- Any drug or herbal medicine with known common toxic reactions to major organs, or any drug that may interfere with the interpretation of efficacy and safety data.

After the last dose of study treatment, the investigator, based on clinical experience and the participant's glycemic control, formulated a new antidiabetic regimen for the participant. The use of the following antidiabetic medications is strictly restricted within a certain time frame:

- Drugs containing GLP-1 receptor agonist components are prohibited from the last dose of study treatment until the end of the safety follow-up phase.
- DPP-4 inhibitor is prohibited within 14 days after the last dose of study treatment.

6.8.3. Permitted concomitant treatment

From the screening period to the end of the double-blind treatment period, all participants should use metformin at a stable dose as background therapy throughout. During the double-blind treatment period, the planned dosage of metformin should not be changed except for medically necessary reasons.

Definition of stable dosage: no change in the planned metformin dosage. Compliance between visits (excluding telephone methods) during the participant Run-in period and double-blind treatment period, as well as overall compliance during the double-blind treatment period, should be ≥80% and ≤120%.

6.8.4. Rescue treatment

During the double-blind treatment period, if a participant experiences fasting plasma glucose levels that meet the criteria for rescue treatment (see Table 6) without any apparent trigger, they must undergo an unscheduled visit within 7 days (including the 7th day) under normal

dietary conditions for fasting venous blood glucose testing at a local laboratory to confirm if the criteria for rescue treatment are met.Table 6. Rescue Treatment Criteria for Fasting Blood Glucose Levels6

Table 6. Rescue Treatment Criteria for Fasting Blood Glucose Levels6

Study Time Period	Rescue Treatment Criteria for Fasting Blood Glucose Levels
Double-Blind Treatment Period (V2–V7)	>15.0 mmol/L
Double-Blind Treatment Period (V11-V26)	>13.3 mmol/L
Double-Blind Treatment Period (V15 excluded) from Week 27 to Week 52	>11.1 mmol/L

If the fasting venous blood glucose levels tested by the local laboratory (including results from both scheduled and unscheduled visits) meet the criteria for rescue treatment, the investigator may provide rescue treatment based on the participant's medical needs, with rescue treatment options selected from the approved antidiabetic regimens in the protocol. It is recommended to add basal insulin. During the rescue treatment period, participants will continue to receive the study treatment according to the protocol.

In addition, during the Run-in period, if a participant's self-measured fasting glucose is > 15 mmol/L, an unscheduled visit must be conducted within 7 days (including the 7th day) under normal dietary conditions upon notification, with fasting venous blood glucose tested at a local laboratory. If the test confirms fasting venous glucose > 15 mmol/L, the investigator will inform the participant to discontinue the investigational product during the Run-in period and withdraw from the study.

7. Termination of trial intervention for participant or withdrawal from trial

7.1. Termination of trial intervention for participant

7.1.1. Discontinuation of trial intervention

Early discontinuation of study treatment does not represent withdrawal from the study. Participants who discontinue the study treatment should continue to complete all items in the remaining study visits as required by the protocol to the greatest extent possible.

The study treatment will be permanently discontinued if the participant meets any of the following conditions:

- The participant requested early withdrawal from the study and permanent discontinuation of study treatment;

- Any AE, laboratory test abnormality, or other medical condition arises, and the investigator determines that continuing treatment with the investigational product is not in the participant's best interest;
- Poor glycemic control in the participant: during the Run-in period, the participant's fasting venous blood glucose > 15.0 mmol/L (if self-monitored blood glucose [SMBG] > 15.0 mmol/L, the participant needs to return to the study site within 7 days for confirmation by the study site); during the treatment period, if the participant still exhibits progressively worsening hyperglycemia after receiving rescue treatment (refer to the rescue treatment criteria in Table 6), and if the investigator determines that the blood glucose level remains excessively high without other contributing factors within 2 weeks after rescue treatment, trial intervention may be recommended for discontinuation.Table 6. Rescue Treatment Criteria for Fasting Blood Glucose Levels6
- Amylase and/or lipase > 3 × ULN, confirmed by repeat testing within 3 days, and confirmed as pancreatitis by clinical symptoms or imaging examination;
- You participate in another clinical trial involving an approved or unapproved investigational product;
- Poor compliance of the participant, which may significantly affect the efficacy and safety evaluation;
- A female participant becomes pregnant during the study;
- Lost to follow-up.
- Death of the participant.
- Early termination of the trial by the Sponsor;
- Important protocol deviations, e.g., the participant is no longer eligible to continue participating in the trial after enrollment;
- Any other situation where the investigator determines it is necessary to discontinue treatment with the investigational product.

When the participant discontinues treatment, the participant will continue to complete the remaining study visits as required by the protocol until the safety visit. During the visits after discontinuation, the investigator will not perform the following: dispense investigational product, PK sampling. The investigator could suggest or provide new alternative treatments (except GLP-1-RA and other prohibited medications, see section 6.8.2) to participants based on their actual situation, and the new dose administration should be recorded in the participant diary.6.8.2

7.2. Withdrawal of participant from the trial

Participants could voluntarily withdraw from the trial at any time, or be asked to withdraw from the trial at any time by the investigator or the sponsor due to safety or behavioral reasons, or inability to comply with the study visit times or procedures required by the protocol at the study site where they were located.

If a participant fails to return to the site for follow-up as agreed, every effort should be made to contact the participant. In any case, every effort should be made to document the participant's outcome, if possible. The investigator should inquire about the reason for withdrawal, request the participant to return any unused investigational product, request the participant to return to the site for a final visit, and follow-up on any unresolved AEs if applicable.

If a participant refuses to go to the study site for further visits, safety information should continue to be followed up unless the participant refuses further disclosure of information or to be contacted. In this case, no study evaluation should be conducted, and no data should be collected.

For participants who are lost to follow-up or have died, the steps for study withdrawal will no longer be applicable.

7.3. Lost to follow-up

If a participant misses scheduled visits at the study site more than 3 times and the study site is unable to establish contact with the participant, the participant is considered lost to follow-up.

If a participant fails to return to the study site for necessary study visits, the following actions shall be taken:

- The study site should make efforts to contact the participant, reschedule any missed visits promptly, provide counseling to the participant about the importance of attending visits on time, and determine whether the participant wishes to and/or should continue with the trial.
- Before considering a participant as lost to follow-up, the investigator or designated personnel shall make every possible effort to contact the participant (e.g., attempting to contact the participant at least three times by phone and, if necessary, sending emails to their email address or postal address, and using social media tools). All the attempts to make contact should be recorded in the medical document.
- If the investigator is still unable to contact the participant, the participant is considered as withdrawal from the trial, with the primary reason being lost to follow-up.

7.4. Specific discontinuation criteria (not applicable)

Not applicable.

8. Test evaluation and procedure

Examinations and procedures specified in the protocol should be performed to the best extent possible in accordance with the protocol. However, unexpected situations beyond the control of the investigators may occur, making examinations difficult. Under such circumstances, the investigator must take all necessary measures to protect the safety and interests of the participants. When the procedure required by the protocol could not be performed, the investigator must record the reason. In addition, the study team must be notified by the investigator of unexpected situations in a timely manner.

This clinical trial should be conducted in accordance with the study protocol, the current Good Clinical Practice (GCP) and relevant laws and regulations.

Participants must be under fasting conditions for all study site visits. The fasting condition is defined as an overnight fast for at least 8 hours (no food or liquid intake other than water).

Early withdrawal from the study: after randomization, participants who are unable or unwilling to continue participating in the study for any reason should undergo an early withdrawal visit. If a participant discontinues the study during the visit, the full content of the early withdrawal visit must be completed, and this visit could serve as the early withdrawal visit.

Safety follow-up visit: all participants who have taken the investigational product (including those who entered the Run-in period but were not successfully randomized) will be required to undergo safety follow-up. Participants who withdraw early from the study are also required to undergo safety follow-up visits.

8.1. Evaluation of screening period and Run-in period

The inspection items during the screening period are detailed in Section 1.3 Schedule of Activities.1.3

During the Run-in period, participants who qualify during the screening will uniformly take HRS-7535 placebo and dapagliflozin placebo. Inspections will be conducted again on V3 (D-4), and randomization criteria will be reviewed on V4 (D1). Participants who meet the randomization criteria will be randomly assigned in a 1:1:1:1 ratio to the HRS-7535 30 mg group, 60 mg group, 90 mg group, and dapagliflozin group.

8.2. Assessment of efficacy and procedures

Assessment of Efficacy was conducted at the time points specified in the Schedule of Activities in Section 1.3. The efficacy assessment measures in this study included HbA1c, fasting plasma glucose (FPG), 2-hour postprandial plasma glucose, fasting serum insulin and C-peptide, 2-hour postprandial serum insulin and C-peptide, homeostasis model assessment of β-cell function (HOMA-β) and insulin resistance (HOMA-IR), 7-point SMBG profile, body weight, and waist circumference.1.3

8.2.1. Glycated hemoglobin A1c (HbA1c)

For screening, HbA1c assessment was performed at the local laboratory. For the efficacy assessments of the study at all following visits, HbA1c was measured at a central laboratory.

8.2.2. Fasting plasma glucose (FPG)

During the screening period, FPG inspection was performed at local laboratory. For efficacy assessment, FPG will be measured at central laboratory during subsequent visits.

8.2.3. Self-measured blood glucose (SMBG)

After successful screening, a glucose meter should be provided to the participant, and all glucose measurements during the study must be completed using this meter. In addition to 7-point SMBG and Fasting SMBG, the participant may perform SMBG as needed (e.g., when symptoms of hypoglycemia or severe hyperglycemia occur), and it is recommended that the participant monitor Fasting SMBG at least once a week. The participant should be provided with oral and written instructions on the use of the glucose meter, including regular calibration according to the manufacturer’s instructions. At subsequent visits, site staff should repeat the instructions for using the meter to the participant as necessary. The participant should be instructed to bring the glucose meter and participant diary at every site visit and should be trained on meter calibration as needed. The glucose meter uses test strips calibrated to plasma values, so all measurements obtained using capillary blood are automatically converted to plasma glucose equivalents and displayed on the meter, which are then recorded as SMBG values. The participant should be instructed on how to log SMBG values in the provided diary and should only record measurements obtained with the provided glucose meter.

For the 7-point SMBG profile, participants should be instructed to perform continuous 7-point SMBG monitoring on any one day within 3 days before the visit (starting from before breakfast on that day and completing 7-point blood glucose collection within 24 hours) and record SMBG values. On the measurement day, participants should maintain normal diet and exercise

levels. If a participant fails to complete one continuous 7-point SMBG test, a second 7-point SMBG monitoring should be performed on a different day.

The SMBG values at the following time points should be recorded in the participant's diary and eCRF (including the date of measurement and actual clock time), always starting with the pre-breakfast measurement:

- Before breakfast
- 120 ± 10 min after the start of breakfast
- Before lunch (recommended within 30 min before meals)
- 120 ± 10 min after the start of lunch
- Before dinner (recommended within 30 min before meal)
- 120 ± 10 min after the start of dinner
- Before bedtime

8.2.4. Mixed meal test

Complete the inspection under fasting conditions on the scheduled Visit day, followed by the mixed meal test: consume instant noodles under fasting conditions in the morning, ensuring all noodles are eaten, and start timing at 0 h from the first bite of instant noodles, aiming to finish within 10 min. Collect venous blood at 2 h (±5 min) after the meal for testing: 2-hour postprandial plasma glucose, 2-hour postprandial serum insulin, and 2-hour postprandial serum C-peptide. Maintain fasting status from the mixed meal until the 2-hour post-meal blood collection.

8.2.5. Body weight and waist circumference

Measurement of body weight under fasting conditions:

1) Perform the measurement in a uniform manner using a calibrated scale (either mechanical or electronic scales) and use the same weighing scale whenever possible. Each weight measurement of a participant should be performed under fasting conditions after the participant has emptied his/her bladder.

2) Before weighing, effort should be made to ensure that the pointer of the weighing scale is pointing at zero and that the weighing scale is placed on a stable and smooth surface;

3) After confirming that the participant has emptied his/her bladder, the participant will be asked to enter the weight measuring area, pull up the curtain (if any), take off the clothing and accessories that increase the weight (coat/outer pants/hat/scarf/necklace/belt,

etc., to ensure that the participant does not feel cold after taking off the clothing), wear single clothes only, and take off the shoes.

4) Participants will be required to stand still in the center of the scale pedal, bear weight evenly on both legs, and place both groups on both sides of the body. Then, the body weight should be read in kg, and the value should be recorded and rounded to one decimal place.

Waist circumference measurement under fasting conditions

1) Participants will be required to be fasting, stand upright, breathe naturally and evenly, relax the abdomen, hang both groups naturally down, keep feet together, and bear weight evenly on both legs.

2) The measurement site between the lower edge of the costal arch and the iliac crest should be fully exposed, and the horizontal position of waist circumference measurement should be the midpoint of the line connecting the lower edge of the costal arch on the bilateral midaxillary line and the iliac crest.

3) A tape should be used to embrace the above horizontal position for one circle. During the measurement, the tape should be close to the skin (not separated by clothes), but it should not be too tight against the skin.

4) During measurement, there should be no intentional abdominal contraction or protrusion. Readings will be taken at the end of a calm exhalation. The unit is cm. The value will be recorded with one decimal place retained.

5) The mean value of 2 measurements should be taken as the measurement result. If the difference between the 2 measurements is more than 1 cm, the group of measurements will be discarded and additional 2 measurements should be performed.

During the study, measurements should be performed by the same designated person as much as possible.

Note: if a participant is not under fasting conditions, an appointment should be made within the visit time window for the participant to return to the hospital again for measurements of fasting body weight and waist circumference.

8.3. Safety evaluation and procedures

See Section 1.3 Schedule of Trial Activities for details.1.3

Safety evaluation included collection of AEs (see Section 8.4), hypoglycemic events, laboratory inspection, vital signs, physical examination, 12-lead ECG, diabetic retinopathy screening and assessment.8.4

8.3.1. Vital signs and physical examination

Participants will undergo physical examination including height, weight, and vital signs (including pulse, systolic pressure, and diastolic pressure). Height will be measured at screening only.

The participant's pulse, systolic pressure and diastolic pressure in sitting position will be assessed. Measurements should be performed after 5 minutes of rest. Vital signs should be measured at least 10 hours after caffeine, alcohol, or nicotine intake.

For each blood pressure measurement, the same group as that at screening is selected for measurement, and the measurement will be taken in a sitting position with the elbow positioned at the same level as the heart.

Blood pressure and pulse will be measured three times, with an interval of at least one minute each time. The average will be calculated based on the three measurements.

If the average of the systolic pressure is ≥159.5 mmHg, it will be rounded to 160 mmHg, making the participant not eligible for the study based on exclusion criteria (the same calculation applies to diastolic pressure).

Physical examination included major body organs, referring to general conditions, skin, lymph nodes, head and neck, chest (including heart and lungs), abdomen, musculoskeletal system, and nervous system.

8.3.2. Laboratory tests

Blood and urine samples involved in laboratory tests will be collected at the time points specified in the Schedule of Trial Activities in Section 1.3 and analyzed at the local/central laboratory. Laboratory tests are specified in Table 7.1.3Table 7. Laboratory Test Items7

Table 7. Laboratory Test Items7

Hematology	Blood biochemistry	Urinalysis
White blood cell count	Total bilirubin	urine pH
Red blood cell count	Direct bilirubin	Urine protein
Platelet count	Alanine aminotransferase	Urinary occult blood
Neutrophil count	Aspartate aminotransferase	Urine erythrocyte
Lymphocyte count	γ-Glutamyl transpeptidase	Urine Leukocytes
Hemoglobin	Alkaline phosphatase	Urine ketone body
Hematocrit	Albumin	Urine Bilirubin
Lymphocyte percentage	Urea nitrogen or urea	Urobilinogen
Neutrophil percentage	Creatinine	
Monocyte percentage	eGFR	
Eosinophils	Uric acid	
Absolute basophil count	Calcium total	
	Potassium	
	Sodium:	
	Chlorine	
	Inorganic phosphorus	

Coagulation Function Test	Thyroid Function Test	Others
Prothrombin time<br>Activated partial thromboplastin time<br>International normalized ratio<br>Thrombin time	Free triiodothyronine<br>Thyroxine, Free<br>Thyroid stimulating hormone	Amylase<br>Lipase<br>Calcitonin<br>Pregnancy Test<br>FSH<br>Urinary albumin/creatinine ratio<br>Triglycerides<br>Total cholesterol<br>High-density lipoprotein cholesterol<br>Low-density lipoprotein cholesterol<br>Hepatitis B Virus Surface Antigen (if positive, HBV DNA test will be added)

Abbreviations: eGFR = estimated glomerular filtration rate; FSH = follicle-stimulating hormone; HBV = hepatitis B virus.

8.3.3. 12-lead ECG

12-lead ECG should be performed by a qualified trained physician at the time points specified in the Schedule of Trial Activities in Section 1.3.1.3

All scheduled electrocardiogram inspections should be performed in a supine position after the participant has been seated for at least 10 minutes. Electrocardiogram measurements will include heart rate, PR interval, RR interval, QT interval and QTcF. QTcF will be calculated by Fridericia formula: QTcF = QT/(RR^0.33). When the electrocardiogram report does not automatically provide RR interval results: if the participant has regular heart rhythm, the RR interval could be obtained by dividing 60 by the heart rate; if the participant has irregular heart rhythm, the RR interval will be provided by the physician or technician evaluating the electrocardiogram report results.

Additional electrocardiogram inspection could be performed if clinically indicated.

8.3.4. Screening and assessment of diabetic retinopathy

Baseline fundus photography should be performed for participants who have successfully passed screening (including laboratory test results). A comprehensive ophthalmic evaluation of baseline fundus photography must be completed prior to randomization to confirm eligibility. Participants with any degree of diabetic retinopathy or macular edema at baseline assessment, and who require treatment based on the clinical judgment of an ophthalmologist, will be excluded (see Exclusion Criterion 8).

All participants need to undergo fundus photography according to the trial schedule in Section 1.3. All participants need to undergo fundus photography at screening, V12 (w32) and V15

(w52) or early withdrawal. If necessary, following the screening guidelines for diabetic retinopathy, the frequency of fundus photography may be increased based on the severity of retinal lesions or macular edema at baseline or during the study.1.3

Fundus photography should be performed on both eyes (unless inspection is not possible due to severe cataract, prosthesis, or other diseases), using the widest field of view available at the study site or by qualified ophthalmic care professionals, such as: 2 fields with at least 60 degrees, or 4 wide fields, or 7 standard fields. Referral to an ophthalmologist or qualified medical personnel for inspection should be made whenever possible.

All fundus photographs will be evaluated by a central ophthalmologist or a medical professional with extensive experience in the assessment of diabetic retinopathy and macular edema. The evaluation will include the presence or absence of diabetic retinopathy and/or macular edema and whether treatment is recommended. Fundus photographs deemed unassessable for any reason will be retaken based on the ophthalmologist's recommendation. Throughout the study, all photographs of the participant should be reviewed by the same qualified assessor whenever possible. Any evidence of diabetic retinopathy, macular edema, or other findings of clinical significance on fundus photographs will require additional ophthalmological follow-up according to local standard treatment. Optical coherence tomography is recommended to confirm cases of macular edema.

The results of the comprehensive ophthalmic assessment, including the final diagnosis of non-proliferative retinopathy, proliferative retinopathy or diabetic macular edema, will be provided to the Investigator and recorded in the specific retinopathy eCRF. Any interventions initiated for diabetic retinopathy or macular edema during the study will be recorded in the concomitant medications eCRF.

8.4. Adverse event (AE) and serious adverse event (SAE)

8.4.1. Definition of AEs and SAEs

AE refers to any adverse medical event that occurs after a participant receives an investigational product, but it does not necessarily have a causal relationship with the treatment. An AE could therefore be any unfavorable and unintended sign (including an abnormal laboratory finding), symptom, or disease temporally associated with the use of a medicinal product, whether or not related to the medicinal product. AEs include but are not limited to:

1) Exacerbation of a pre-existing (before entering the clinical trial) medical condition/disease (including exacerbation of symptoms, signs, and laboratory test abnormalities);

2) Any new occurrence of AE: any adverse medical conditions newly occurred (including symptoms, signs, newly diagnosed diseases);

3) Clinically significant abnormal laboratory test results.

SAE refers to any adverse medical event that occurs in a participant after receiving any dosage of the investigational product:

- Events leading to death;
- Life-threatening event (at risk of death at the time of the event);
- For events that require hospitalization (as defined in Section 12.2.1) or prolongation of hospitalization;12.2.1
- An event resulting in persistent or significant disability/incapacity (substantial disruption of the ability to conduct normal life functions);
- Congenital abnormalities or birth defects;
- Important Medical Event
  - Medical and scientific judgments must be used to decide whether it is an Important Medical Event. Important Medical Events may not be immediately life-threatening, result in death, or hospitalization. However, if the event may jeopardize the participant or require intervention to prevent one of the outcomes listed in the SAE definition above, the Important Medical Event should be reported as an SAE.
  - Examples include allergic bronchospasm requiring emergency treatment in the emergency room or at home, non-hospitalized cachexia or convulsions, and drug dependence or drug abuse.

Further details on AEs and SAEs are provided in Sections 12.1 and 12.2.12.112.2

8.4.2. Collection of AEs and SAEs

Clinical trial safety events, including adverse medical events, AEs/SAEs/AESIs, and pregnancy and drug overdose events prior to the first dose, will be collected from the signing of the ICF by the participants until the end of the safety follow-up period (2 weeks after the last dose) (table 8).Table 8. Collection of Clinical Trial Safety Events8

Table 8. Collection of Clinical Trial Safety Events8

Period	Collection Requirements
Starting from the participant's signing of Informed Consent Form until the end of the safety follow-up period*	Untoward medical events/AEs/SAEs prior to first dose: collection started after signing the Informed Consent Form<br>Pregnancy event/drug overdose: collection started after the first dose<br>AESI: collection started after randomization
After the aforementioned periods	SAEs and AESIs related to the investigational product

Abbreviations: AE = adverse event, AESI = adverse event of special interest, SAE = serious adverse event.
*The end of the safety follow-up phase refers to 2 weeks after the last administration of the investigational product.

8.4.3. Identification of AEs and SAEs

At each study visit, the investigator should assess whether the participant has experienced AEs or SAEs by inquiring and conducting physical examinations or laboratory tests. All events, whether observed by the investigator or reported spontaneously by the participant, must be assessed and recorded by the investigator.

8.4.4. Recording of AEs and SAEs

All AEs should be recorded in detail in the AE page of the eCRF, including: event term, start and end dates, severity, seriousness, causality between the event and the investigational product, actions taken regarding the investigational product, and outcome. If the event meets the SAE criteria, it must be reported according to the SAE reporting procedure.

For more details on the assessment of the severity and causality of AEs and SAEs, please refer to Sections 12.3 and 12.4.12.312.4

8.4.5. Follow-up of AEs/SAEs

All AEs should be followed up until symptoms disappear, or clinically relevant changes in laboratory values return to baseline and/or reach a stable state, or a reasonable explanation is obtained (such as lost to follow-up and death).

8.4.6. SAE reporting requirements

In case of SAE, regardless of whether it is an initial report or a follow-up report, the investigator must immediately complete the "SAE/AESI/Drug Overdose Report Form", sign and date it, notify the sponsor within 24 hours of awareness, and report to relevant organizations in a timely manner as required by local regulations.

For SAEs that occur after the safety follow-up phase, those suspected to be related to the investigational product should be collected. SAEs should be documented in detail including symptoms, severity, correlation with the investigational product, time of onset, time of

treatment, measures taken, follow-up time and method, and outcome. If the investigator considers an SAE unrelated to the investigational product but potentially related to study conditions (e.g., discontinuation of original treatment, or comorbidities during the trial), this relationship should be thoroughly explained in the narrative section of the SAE report form.

The email address of the sponsor for receiving reports of SAEs (as well as AESIs, pregnancy and drug overdose) for this project is: hengrui_drug_safety@hengrui.com.

8.4.7. SAE reporting requirements

Except for those SAEs explicitly exempted from immediate reporting per the protocol, the investigator must submit a written report of all SAEs to the sponsor within 24 h of becoming aware of the event. A detailed written follow-up report must be submitted in a timely manner thereafter.

For reports involving fatal events, the Investigator shall provide the Sponsor and Institutional Review Board with other required materials, such as autopsy reports and final medical reports.

8.4.8. Suspected unexpected serious adverse reaction report

Suspected Unexpected Serious Adverse Reaction refers to a serious adverse reaction whose nature and severity of clinical manifestations are beyond the existing information in the Investigator's Brochure of the study drug, the package insert of the marketed Medicinal Product or the Summary of Product Characteristics.

After the sponsor receives safety-related information from any source, it shall immediately analyze and evaluate it, including seriousness, correlation with the study drug, and whether it is an expected event. The sponsor shall quickly report suspected unexpected serious adverse reactions to the drug regulatory authority, the competent health authority and all investigators participating in the clinical trial, and submit them to the clinical trial institution and Institutional Review Board according to the requirements of the study site.

8.4.9. Adverse events of special interest (AESI)

For the reporting of AESIs specified in the clinical study protocol, "SAE/AESI/Drug Overdose Report Form" must be filled out within 24 h of the investigator's first knowledge of the event and reported to the sponsor. This procedure applies to all AESIs regardless of whether they meet the SAE criteria. The reporting procedure for AESIs is the same as that for SAEs. If they meet the SAE definition, they must also be reported according to the SAE reporting process.

AESIs in this study include:

- Liver function test abnormal;

- Level 3 hypoglycemia: see section 8.4.9.2 for specific definition;8.4.9.2
- Calcitonin abnormal: calcitonin level ≥50 ng/L after treatment administration, and the specific value increases by > 50% from baseline;
- Acute pancreatitis
- Lactic acidosis.

8.4.9.1. Abnormal liver function tests

AST and/or ALT abnormalities accompanied by abnormally increased TBIL that meet the following criteria (1), (2) and (3) with no other reasons should always be reported as AESIs.

Table 9. Criteria for Determining Abnormal Liver Function Tests9

**Condition Met**	**Determination Criteria**
1) ALT or AST abnormalities	Normal ALT and AST at baseline: ALT or AST > 3 × ULN during the treatment period;<br>Abnormal ALT or AST at baseline: ALT or AST > 2 × baseline level during the treatment period, and > 3 × ULN; or > 8 × ULN (whichever is lower).
2) TBIL abnormality	Normal TBIL at baseline: TBIL > 2 × ULN during the treatment period;<br>Abnormal TBIL at baseline: TBIL increases by > 1 × ULN during the treatment period or its value is > 3 × ULN (whichever is lower).
(3) No evidence of cholestasis (e.g., elevated serum ALP due to gallbladder or bile duct disease, etc.)	

Abbreviations: ALP=alkaline phosphatase, ALT=alanine aminotransferase, AST=aspartate aminotransferase, TBIL=total bilirubin, ULN=upper limit of normal.

If a participant had abnormal AST and/or ALT levels and abnormally increased TBIL levels during the treatment period or follow-up period, the participant had to return to the study site for investigation and assessment as soon as possible (preferably within 48 h) after learning about the abnormal results. Examination and assessment should include relevant laboratory tests of liver function, thorough inquiry of medical history and physical examination.

In addition to repeat testing of AST and ALT, liver function tests may include albumin, creatine kinase, TBIL, direct and indirect bilirubin, gamma-glutamyl transferase, prothrombin time/international normalized ratio, and alkaline phosphatase. Collection of detailed medical history and relevant information is recommended to include the history of alcohol use, acetaminophen, soft drugs, various supplements, family medical history, history of sexual behavior, travel history, history of contact with patients with jaundice, surgical history, history of blood transfusion, hepatopathy and allergic diseases, or possible occupational chemical exposure. Further examinations may also include tests on acute infections of hepatitis A, B, C, D and E, as well as imaging examination of the liver (such as the biliary tract).

8.4.9.2. Hypoglycemic episodes

8.4.9.2.1. Definition and classification of hypoglycemic episodes

Hypoglycemic episodes were classified according to the 2024 American Diabetes Association (ADA) Standards of Medical Care in Diabetes@[]@ and the 2020 Chinese Diabetes Society (CDS) guidelines for the prevention and treatment of type 2 diabetes in China@[3]@ (table 10).[93]Table 10. 2024 ADA-Recommended Classification of Hypoglycemic Episodes10

Table 10. 2024 ADA-Recommended Classification of Hypoglycemic Episodes10

Hypoglycemia Level	Definition
Level 1	Blood glucose < 3.9 mmol/L and ≥3.0 mmol/L
Level 2	Blood glucose < 3.0 mmol/L
Level 3	No specific glucose threshold; hypoglycemia associated with severe cognitive and/or physical impairment requiring external assistance for recovery

Abbreviations: ADA = American Diabetes Association.

Clinical manifestations of hypoglycemia (related to blood glucose levels and glucose-lowering rate) may include: sympathetic nerve excitement (e.g., palpitations, anxiety, sweating, hunger, etc.) and central nervous system symptoms (e.g., altered consciousness, cognitive impairment, convulsions, and coma). When hypoglycemia is suspected, the participant should measure and record glucose levels as soon as possible using the study-provided glucometer.

8.4.9.2.2. Recording and reporting of hypoglycemic episodes

Grade 3 hypoglycemia was defined as AESI.

Hypoglycemic episodes meeting the definition of SAEs or AESIs should be recorded on both the AE page and hypoglycemic episode page of eCRF, and reported following the procedure for SAE or AESI. All other hypoglycemic episodes will be recorded on the hypoglycemic episode page only, including general information of hypoglycemia (time, SMBG measurement results, symptoms, treatment, possible causes, etc.).

During the study, the participant should record hypoglycemia events in the participant diary.

If blood glucose values are not available at the time of the suspected hypoglycemic episode based on clinical manifestations, the event, i.e., a possible symptomatic hypoglycemic episode, should still be recorded.

8.5. Pregnancy

If a female participant becomes pregnant during the clinical trial, the participant must discontinue the trial intervention; if the partner of a male participant becomes pregnant during the clinical trial, the participant could continue the clinical study. The investigator should fill

out the "Pregnancy Report Form" within 24 hours after becoming aware of the pregnancy event to report it to the sponsor, and report to the relevant authorities as required by local regulations. The investigator was required to follow-up on the pregnancy event until the final outcome (including any early termination of pregnancy or delivery), which, in the case of delivery, would be 1 month thereafter, and report the pregnancy outcome to the sponsor or to other relevant authorities according to local requirements. If the pregnancy outcome meets the SAE criteria (such as ectopic pregnancy, spontaneous abortion, intrauterine fetal demise, neonatal death, congenital anomaly, etc.), the investigator should report it according to the SAE procedures.

If a participant experiences any SAE during pregnancy, the SAE should be reported according to the SAE reporting procedure.

8.6. Pharmacokinetics

PK blood samples will be collected from participants at V4, V6, V7, V8, V9, V10, V11, V12, and V15 as shown in the trial flow chart in Section 1.3. The administration times of the second dose of HRS-7535/placebo, dapagliflozin/placebo, and metformin prior to blood sampling must be accurately recorded, and plasma concentrations of HRS-7535, dapagliflozin, and metformin will be measured. At least 40 participants will undergo intensive blood sampling at any one visit from V8 to V11 at the center, collecting PK blood samples at -60 min before HRS-7535/placebo administration on the day of collection and at 0.5 h (±15 min), 1 h (±20 min), 2 h (±30 min), 4 h (±30 min), 6 h (±30 min), 8 h (±30 min), and 10 h (±30 min) after administration, to fully evaluate the PK characteristics of HRS-7535, dapagliflozin, and metformin.1.3

For participants with unexpected AEs and/or SAEs, additional blood samples may be collected outside the planned sampling, and the sampling date and specific time, along with the last dosing before sampling, must be recorded in eCRF.

Every effort should be made to ensure that the pharmacokinetic samples will be taken according to the planned relative time to administration. The exact sampling times will be indicated in full in the eCRF. If sampling could not be completed as scheduled or the sampling time is missed, the sampling could be performed at another time after a consensus is reached among Investigator, participant and Sponsor.

PK samples of the study drug will be analyzed using a validated method. The study manual will provide information on the collection, processing, storage and transportation of blood samples.

The remaining PK samples may be used for some exploratory analyses, including but not limited to the determination of plasma concentrations of concomitant medications.

8.7. Genetics (not applicable)

Not applicable.

8.8. Biomarkers (not applicable)

Not applicable.

8.9. Immunogenicity (not applicable)

Not applicable.

8.10. Healthcare resource utilization and health economics (not applicable)

Not applicable.

9. Statistical considerations

This section provides an overview of the study data collection and analysis methods as detailed in the Statistical Analysis Plan (SAP) and maintained by the sponsor.

9.1. Analysis population

Intention-to-treat (ITT) analysis set: this population included all randomized participants. Participants will be analyzed according to the group they are randomly assigned to. ITT is the primary analysis population for efficacy.

Safety set (SS): includes all participants who received at least one dose of the Investigational Product after randomization. Participants will be analyzed according to the group they were randomly assigned to.

PK concentration analysis set (PKCS): all participants who have been randomized to receive HRS-7535 treatment and have at least one evaluable PK concentration data post-dose.

9.2. Analysis supporting the primary objective

9.2.1. Statistical hypothesis and decision rules

Let D be the mean difference in the change from baseline in HbA1c at Week 32 between the two treatment groups (HRS-7535 group [90 mg or 60 mg or 30 mg] minus the dapagliflozin group).

When testing the non-inferiority of HRS-7535 relative to dapagliflozin, the null hypothesis (h0) and alternative hypothesis (HA) are:

- Non-inferiority: h01 D ≥ 0.4% and HA1 D < 0.4%

If the upper limit of the two-sided 95% Confidence Interval (CI) is less than 0.4%, the non-inferiority of the HRS-7535 group compared to the dapagliflozin group is considered to be achieved for the primary endpoint.

In addition, if the preceding test in the fixed sequence testing is confirmed, a superiority test based on a one-sided 0.025 will be conducted for the change in HbA1c from baseline at Week 32 between HRS-7535 and dapagliflozin.

Hypothesis Testing Sequence and Decision Rules:

A fixed sequence testing method will be used to conduct the tests at a one-sided significance level of 0.025 according to the following predefined testing sequence. If the preceding test is confirmed, the subsequent test could be performed. If the preceding test fails, the testing will be stopped. Thus, the overall Type I Error rate of this study will be controlled within 0.025 (one-sided).

The alternative hypothesis Hxx, where the first subscript 1, 2, and 3 represent the HRS-7535 90 mg, 60 mg, and 30 mg groups, respectively; the second subscript 1-6 represent different endpoints:

- H11, H21 and H31: non-inferiority of HRS-7535 90 mg, 60 mg and 30 mg groups versus dapagliflozin group in HbA1c change from baseline after 32 weeks of treatment;
- H12, H22 and H32: superiority of HRS-7535 90 mg, 60 mg, 30 mg groups vs. dapagliflozin in HbA1c change from baseline after 32 weeks of treatment;
- H13, H23 and H33: superiority of the proportion of participants with HbA1c< 7.0% in HRS-7535 90 mg, 60 mg and 30 mg groups versus dapagliflozin group after 32 weeks of treatment;
- H14, H24 and H34: superiority of the proportion of participants with HbA1c ≤6.5% after 32 weeks of treatment in HRS-7535 90 mg, 60 mg and 30 mg groups vs. dapagliflozin group;
- H15, H25 and H35: superiority of FPG change from baseline at Week 32 in HRS-7535 90 mg, 60 mg and 30 mg groups vs. dapagliflozin group;
- H16, H26 and H36: superiority of HRS-7535 90 mg, 60 mg and 30 mg groups versus dapagliflozin group in weight change from baseline after 32 weeks of treatment;

The following hypotheses were tested sequentially at a one-sided significance level of 0.025: h11, H21, H31, H12, H22 and H32, H13, H23 and H33, H14, H24 and H34, H15, H25, H35, H16, H26 and H36. Testing was discontinued if the one-sided p-value was greater than 0.025.

9.2.2. Primary estimand analysis

For the primary estimand, the primary study endpoint will be analyzed based on the ITT set. It will be analyzed using a mixed-effect model repeated measure (MMRM) based on restricted estimation maximum likelihood (REML). The model included treatment, Visit, and the interaction between treatment and Visit as fixed effects, baseline HbA1c as the covariate, and Participant as the random effect. The UN or alternative CS covariance matrix was employed. The least squares mean, standard error, 95% CI of the change from baseline in HbA1c at Week 32 for each group and between groups were estimated and listed, as well as the p-value for the difference between each dosage group of HRS-7535 and the dapagliflozin group. Hypothetical strategy was applied for all intercurrent events, data after intercurrent events were not included in the analysis.

A sensitivity analysis will be performed on the primary estimand. See the SAP for the detailed method.

9.2.3. Supplemental estimand analysis

For the supplementary estimand, the primary study endpoint will be analyzed based on the ITT set. It will be analyzed using a mixed-effect model repeated measure (MMRM) based on restricted estimation maximum likelihood (REML). The model included treatment, Visit, and the interaction between treatment and Visit as fixed effects, baseline HbA1c as the covariate, and participant as the random effect. An UN or alternative CS covariance matrix was used. The least squares mean, standard error, 95% CI of the change from baseline in HbA1c at Week 32 for each group and between groups, as well as the p-value for the difference between each dosage group of HRS-7535 and the dapagliflozin group, were estimated and listed. A therapy strategy was applied for all intercurrent events, and data after intercurrent events were included in the analysis.

9.3. Analysis supporting secondary objectives

9.3.1. Secondary estimand analysis

The analysis of secondary estimands will be based on ITT, and all intercurrent events will adopt the hypothetical strategy.

For binary endpoints (response/no response), generalized estimating equations (GEE) will be used for analysis if applicable; for continuous endpoints with two or more repeated measurements after randomization, MMRM will be used for analysis if applicable. For continuous endpoints with only one measurement after randomization, analysis of covariance

(ANCOVA) will be used if applicable, with missing data imputed using last observation carried forward (LOCF).

Descriptive summaries were performed for the proportion of participants receiving rescue treatment, the number of hypoglycemia events, and the incidence.

9.4. Analyses to support exploratory objectives

For continuous endpoints with normal distribution and two or more repeated measurements after randomization, MMRM will be used for analysis where applicable, with specific analysis methods detailed in the SAP. Exploratory endpoints will be summarized descriptively.

9.5. Safety analysis

Safety analysis will be performed based on the SS.

AEs were coded according to the Medical Dictionary for Regulatory Activities (MedDRA). List the severity of all AEs and their relationship to the Investigational Product. Summarize the number and percentage of participants with the following TEAEs:

- TEAE
- Treatment-Related TEAE
- With TEAEs Leading to Treatment Discontinuation
- AESI
- SAE

AEs, hypoglycemia events, vital signs, physical examinations, laboratory inspections, and electrocardiogram were summarized and listed using descriptive statistics.

9.6. Other analyses

9.6.1. Pharmacokinetic analysis

Based on the PKCS, descriptive statistics will be performed for the plasma concentrations of HRS-7535, dapagliflozin and metformin by dose group, planned sampling time point and Visit.

9.6.2. Subgroup analysis

Subgroup analyses will be performed on the primary efficacy endpoints including but not limited to the following, with the same analytical methods as the primary estimand analysis:

- Baseline HbA1c (≤8.5% versus > 8.5%);
- metformin prolonged-release tablet (≤1500 mg/day vs. > 1500 mg/day);
- Gender (males versus females);
- Baseline BMI (≥24 kg/m2 vs. < 24 kg/m2);

- Duration of type 2 diabetes (≥median [months] vs. <median [months]).

Refer to the SAP for details on other subgroup analysis.

9.7. Interim analysis (not applicable)

Not applicable.

9.8. Sample size Estimation

The sample size calculation is based on the change from baseline in HbA1c at Week 32. Referring to the commonly used margin for HbA1c, the non-inferiority margin for treatment difference in this study will be set at 0.4%. Participants will be randomized into groups at the ratio of 1:1:1:1. Assuming an treatment difference of 0 after considering intercurrent event and a standard deviation of 1.1%, 800 participants (200 per group) will provide an overall power of 87% to detect non-inferiority of HRS-7535 at all dosage groups (30 mg, 60 mg, 90 mg) compared to the dapagliflozin group at a one-sided significance level of 0.025.

In addition, assuming that the treatment difference between each dosage group of HRS-7535 (30 mg, 60 mg, 90 mg) and the dapagliflozin group after considering intercurrent events is -0.4%, with a standard deviation of 1.1%, 800 participants (200 participants per group) will provide an overall power of 87% to detect the superiority of each dosage group of HRS-7535 (30 mg, 60 mg, 90 mg) over the dapagliflozin group at a one-sided significance level of 0.025.

9.9. Protocol deviations

A protocol deviation refers to any change and non-compliance with the clinical study protocol design and relevant process documents involved in the protocol. Different protocol deviations have different effects on the rights and interests of participants and on the integrity, accuracy and reliability of trial data. Protocol deviation could come from participants, investigators, or study site staff. Depending on the root cause of the deviation, corrective and preventive actions need to be formulated and implemented promptly.

10. General considerations: regulatory, ethical, and study oversight

10.1. Regulatory and ethical considerations

This clinical trial must be conducted in compliance with the Protocol, ethical principles of the Declaration of Helsinki, current GCP and relevant laws and regulations. Before the trial starts, the Protocol and its amendments, ICF and other relevant documents (such as recruitment materials) should be provided to the Institutional Review Board. This trial must be approved

by the relevant regulatory authorities and the Institutional Review Board of the hospital before it could be implemented.

Neither the sponsor nor the investigator shall unilaterally amend the protocol without the consent of both parties. Any amendment to the protocol must be reviewed and approved by the institutional review board before implementation. If necessary, other relevant documents should be revised accordingly and submitted and/or approved as required by the institutional review board.

If the investigator has to initiate a process that deviates from the protocol to eliminate urgent harm to a participant, the Institutional Review Board and the sponsor must be notified in writing immediately after the deviation is implemented, along with an explanation and documentation of any protocol deviations made. In addition, the investigator is responsible for submitting the interim reports of the study on a regular basis in accordance with the relevant requirements of the Institutional Review Board and shall notify the Institutional Review Board of the completion of the study.

10.2. Committee (not applicable)

No external Data Monitoring Committee (DMC) or Safety Monitoring Committee (SMC) was established for this trial.

10.3. Informed consent process

Informed consent process: informed consent begins before a participant agrees to participate in the clinical trial and continues throughout the entire clinical trial process. The investigator or designated research personnel must provide the latest version of the Informed Consent Form approved by the Institutional Review Board, discuss in detail and fully with the participant or his/her guardian the risks and potential benefits of participating in the trial, introduce the details of the trial, and answer any related questions raised by the participant or his/her guardian. Participants may only start participating in the trial after providing written informed consent. During the entire clinical trial process, participants may voluntarily withdraw from the trial at any time. One copy of the Informed Consent Form will be retained by the participant. Even if participants refuse to participate in this trial, their rights and interests will be fully protected, and the quality of their medical care will not be affected in any way.

Requirements for re-screening informed consent: participants who have previously experienced screen failure (as defined in Section 5.6) are allowed to be re-screened, with each participant permitted only one re-screening throughout the trial. During re-screening,

participants must re-sign the informed consent form and re-register to obtain a new participant number.5.6

10.4. Data protection

10.4.1. Confidentiality of participant information

The participant information will be kept strictly confidential by the investigator, participating study personnel, sponsor and its agency. Confidentiality also covers biological samples and genetic tests in addition to participants' clinical information. Therefore, the trial protocol, documentation, data and all other information generated therefrom will be kept strictly confidential. Without the prior written approval of the sponsor, all information on trial or data should not be disclosed to any unauthorized third party.

All trial procedures will be performed as confidentially as possible.

The Monitor and other authorized representatives of the Sponsor, Institutional Review Board, and drug regulatory authority may inspect all documents and records that the Investigator is required to maintain, including but not limited to: medical records and the Participant's medication records. The Study site shall permit access to these records.

The participant's contact information will be stored at each study center safely and only provided for internal use during the study. At the end of the trial, all records will continue to be stored in a secure place in accordance with the time limit specified by the local Institutional Review Board and regulations.

10.4.2. Data protection

All parties will comply with all applicable laws, including those related to the implementation of institutional and technical measures to ensure the protection of participant data.

Participants' personal data will be stored at the study site in encrypted electronic and/or paper form, password protected or in a locked storage room to ensure that only authorized study staff have access. The study site will take appropriate technical and organizational measures to ensure that personal data could be restored in the event of a disaster. In the event of a potential personal data breach, the site will be responsible for determining if a personal data breach has occurred and, if so, for providing a breach notification as required by law.

To protect the rights and freedoms of the participants with respect to data processing, participants will be assigned a separate specific identification number. Any participant records or datasets transferred to the sponsor will contain this identification number; the participant's first name will not be transferred. All other identifiable data transferred to the sponsor will be

replaced by this separate specific identification number. The study site will maintain a confidential list of participants participating in the trial that could link each participant's identification number to their actual identity and medical records. During the data transfer process, the sponsor will protect the confidentiality of the participants' personal data in accordance with the trial protocol and applicable privacy laws.

10.5. Early termination or discontinuation of trial

This trial could be terminated early for good reasons. This may be due to the decision of the drug regulatory authority, changes in the comments of the Institutional Review Board, issues with the efficacy or safety of the study drug, or the judgment of the sponsor. In addition, the sponsor reserves the right to stop the development of HRS-7535 at any time.

If the sponsor terminates or suspends the trial prematurely, it shall immediately inform the investigator, the clinical trial institution and the drug regulatory authority, and explain the reasons. Upon receipt of the notification, the investigator shall promptly notify the participants and give the participants appropriate treatment and follow-up. In addition, the investigator shall immediately report to the clinical trial institution and the Institutional Review Board, and provide detailed written explanation.

Reason(s) for premature discontinuation or suspension of the trial may include the following:

- Confirmed unexpected, major, or unacceptable risks to the participants;
- The investigator fails to comply with the protocol, the requirements of the ethics committee or drug regulatory authority, the sponsor's procedures, or the current GCP;
- Other situations, such as enrollment difficulties, no longer complying with medical practice, changes in the sponsor's research and development strategy.

The trial may continue once the drug safety, protocol compliance, and data quality issues, etc. that caused the suspension of the trial are resolved, and the approval of the sponsor, Institutional Review Board, or the drug regulatory authority is obtained.

11. General considerations: risk management and quality assurance

11.1. Data quality assurance

In order to ensure the quality of the trial, the sponsor and the investigator will jointly discuss and formulate the clinical trial plan before the official start of the trial, and ensure that the study staff participating in the implementation of the trial have received appropriate training on GCP. Each study center shall manage the investigational medicinal products in accordance with the standard operating procedures, including receipt, storage, dispensing, recovery and destruction.

According to the GCP guidelines, necessary procedures should be taken during the design and implementation stages of the trial to ensure that the collected data are accurate, consistent, complete and credible. All observed results and abnormal findings in the clinical trial must be verified and recorded in a timely manner to ensure data reliability. The instruments, equipment, reagents, standards, etc. used in various inspection items in clinical trials should have strict quality standards and ensure that they work under normal conditions.

The sponsor or designee will conduct monitoring to confirm that the data completed in the eCRF by the study site staff are complete, accurate, and traceable to ensure that the safety and rights of the participants are protected and that the trial is conducted in accordance with the protocol, the GCP and all relevant laws and regulations. The investigator must ensure the accuracy of the source documents (source data) for the information entered into the eCRF.

The regulatory agencies, Institutional Review Board, Sponsor’s Monitors and/or auditors may systematically examine clinical trial-related activities and documents to evaluate whether the trial was conducted in accordance with the requirements of the Protocol, SOP and relevant regulations, and whether the trial data was recorded in a timely, truthful, accurate and complete manner. The Audit should be performed by personnel not directly involved in this clinical trial.

The monitoring plan will provide detailed monitoring strategies (e.g., risk-based operational and quality management measures, such as risk management and mitigation strategies as well as analysis risk-based controls), methods, responsibilities and requirements, including handling of non-compliance issues and monitoring techniques (site-level, remote or on-site monitoring).

The sponsor or its designated personnel is responsible for the data management of this study, including data quality checks.

The sponsor is responsible for actions delegated to other individuals (e.g., contract research organization).

11.2. Source data

The source documents provide evidence for the participants' participation in the clinical study and substantiate the integrity of data collected. Source documents will be archived at the investigator's institution.

The data in the eCRF must be consistent with the source documents, or the discrepancy must be reasonably explained. Depending on the needs of the trial, the investigator may require previous medical records or transcribed records. In addition, current medical records must be provided.

The Sponsor's data monitoring plan defines the composition and source of source data.

The Sponsor's data management plan defines the instructions for the use of electronic information systems.

The Investigator must ensure that the data entered in the eCRF could be traced back to the source records, and archive the original documents.

12. Appendix: adverse events and serious adverse events - definitions, severity, and causality

12.1. Definition and supplement of AE

12.1.1. Drug overdose

Drug overdose is defined as the participant intentionally or unintentionally, actively or passively receiving a single-day dosage of HRS-7535/placebo more than 180 mg, or a single-day dosage of dapagliflozin morethan 10 mg.

In this study, when a drug overdose event occurs, the Investigator shall complete the "SAE/AESI/Drug Overdose Report Form," sign and date it, and notify the Sponsor within 5 calendar days of becoming aware of the event.

If the event of drug overdose meets the SAE criteria, it must be reported according to the SAE reporting procedure.

12.2. Description and supplement of SAE definition

12.2.1. Hospitalization

Hospitalization is defined as any first admission (even if less than 24 hours) or prolongation of hospitalization. Hospitalization does not include the following:

- Rehabilitation facility;
- Sanatorium;
- Routine admission to the emergency department (less than 24 h);
- Same-day surgery (such as outpatient/same-day/ambulatory surgery).

Hospitalization or prolonged hospitalization unrelated to AE is not considered an SAE. For example, the followings are not considered SAEs:

- Admission for treatment of a preexisting condition not associated with the development of a new AE or with a worsening of the preexisting condition (e.g., for workup of a persistent pretreatment laboratory abnormality);
- Hospitalization for administrative reasons (e.g., annual physical examination);
- Protocol-specified admission during the clinical trial (e.g., for a procedure required by the study protocol);
- Elective hospitalization unrelated to AE (e.g., elective cosmetic surgery);
- Scheduled treatment or surgical procedure (should be documented throughout the entire study protocol and/or in participants' individual baseline information);
- Admission exclusively for the administration of blood products.

Diagnostic or therapeutic invasive (such as surgery) and non-invasive procedures should not be reported as AEs, but it should be reported when the disease condition leading to this procedure meets the definition of AE. Acute appendicitis that develops during the AE reporting period and meets the AE requirements should be reported as an AE, and the resulting appendectomy should be recorded as treatment of the AE.

12.3. Severity

When filling out the AE form in the eCRF, the investigator will use mild, moderate, and severe to describe the severity of AEs. To standardize the criteria, the severity of events is graded as follows:

- Mild: usually transient, generally does not affect the participant's activities of daily living, and may only require minimal treatment;
- Moderate: the participant experiences discomfort and disruption of activities of daily living that usually require treatment to alleviate, but do not pose a risk of significant or permanent harm to the participant;
- severe: events interrupt a participant's usual daily activity and may require systemic drug therapy or other treatment.

Pay attention to distinguish between the severity and seriousness of AEs. The mild, moderate, and severe grades above are only used to describe the severity of AEs. For example, headache might be manifested as severe in severity, but it could not be listed as an SAE unless it meets the SAE criteria.

12.4. Causality

The causality assessment between an AE and the Investigational Product refers to the comprehensive evaluation to determine whether there is a reasonable possibility that the Investigational Product caused or contributed to the AE. The factors for judgment include whether the occurrence of the AE has a reasonable temporal relationship with the administration of the Investigational Product, the characteristics of the Investigational Product, the toxicological and pharmacological effects of the Investigational Product, the use of concomitant medications, the participant's underlying diseases, medical history, family history, dechallenge and rechallenge reactions, etc. Facts (evidence) or arguments demonstrating the causality should usually be provided.

The investigator should assess the causality between AEs and the investigational product as "definitely related", "possibly related", "unlikely related", "not related", and "unassessable".

13. Appendix: definitions and supporting operational details

13.1. Contraception and pregnancy test

13.1.1. Definitions related to fertility

Female participants are not required to use contraception if they are considered infertile in any of the following circumstances:

- Postmenopausal status is defined as women aged over 45 with natural menopause for at least 12 months without any other physiological or medical cause. Follicle Stimulating Hormone (FSH) levels in the postmenopausal range in women without hormonal contraception or hormonal replacement therapy could be used to confirm postmenopausal status. However, if menopause is less than 12 months, FSH alone at postmenopausal levels is not sufficient to confirm postmenopausal status.
- Hysterectomy and/or bilateral oophorectomy, bilateral salpingectomy;
- Infertility caused by congenital or acquired conditions (e.g., uterine aplasia, Turner syndrome, ovarian failure, etc. as confirmed by a gynecologist).

13.1.2. Contraception

Male participants with azoospermia (caused by vasectomy or other potential diseases) will be required to use condoms and their partners do not need to take contraceptive measures.

Female participants are not required to use contraception if they are not of childbearing potential (see Section 13.1.1).13.1.1

Female participants of childbearing potential and male participants whose partners are women of childbearing potential must agree to use contraception, refrain from sperm/egg donation from the signing of the ICF to 2 weeks after the last dose of the investigational product, and comply with one of the following requirements:

- Abstinence (avoid heterosexual intercourse).*
- or
- During heterosexual intercourse, female participants (or partners of male participants) must use a highly effective method of contraception, while male participants must use a condom concurrently.

*The reliability of sexual abstinence needs to be assessed based on the duration of the clinical trial and the participant's personal preference and lifestyle. For those who choose to completely not have a sexual life, abstinence (avoiding heterosexual intercourse) could be used alone for contraception. Periodic abstinence (e.g., based on ovulation, symptomatic body temperature, etc.), use of spermicides only, lactational menopause, and withdrawal are not acceptable methods of contraception.

Highly effective contraceptive methods are those that result in a failure rate of less than 1% per year when used consistently and correctly and include the following:

- Contraceptive pills (containing estrogen/progestin or progestogen) proven to be effective, administered orally, by injection, intravaginally, implantably, or transdermally;
- Properly placed intrauterine device (IUD) or effective intrauterine hormone-releasing system (IUS);
- Medically confirmed vasectomy of a female participant's male partner. For female participants, the male partner should be their only sexual partner.
- Bilateral tubal ligation or bilateral tubal occlusion (the occlusion surgery has been proven effective by related instruments).

In addition, participants need to know: the investigator needs to be notified as soon as the chosen contraceptive method is discontinued, or as soon as the participant or the participant's partner suspects or confirms a pregnancy.

13.1.3. Pregnancy test and FSH test

In this study, the pregnancy test and FSH testing rules are as follows:

- Female participants aged 45 years or younger must complete pregnancy tests as specified in the protocol, with 2 serum pregnancy tests performed during the screening period (V1) and before randomization (V3). After a negative pregnancy test result during the screening period (V1), appropriate contraceptive measures should be initiated, and the Run-in medication could be started. A serum pregnancy test will be repeated before randomization (V3), and the investigational product will be administered only after confirmation of a negative result. Pregnancy testing will also be routinely conducted during the treatment period or upon early withdrawal, or additionally performed when a menstrual cycle is missed or when signs of pregnancy are suspected. Additional pregnancy testing will be performed when required by the Institutional Review Board or local regulations. If a positive pregnancy result is confirmed, the female participant will discontinue the investigational product but may remain in the study.
- For postmenopausal female participants aged > 45 years and ≤65 years, a pregnancy test and FSH test will be required for confirmation. If the FSH results indicate postmenopausal level (FSH> 40 mIU/mL), these female participants could be considered of non-childbearing potential, and no further pregnancy test is needed during follow-up; if the FSH results indicate non-postmenopausal level (FSH≤40 mIU/mL), pregnancy test is still required during the trial.

- Menopausal women over 65 years old, deemed infertile by the investigator, are not required to undergo pregnancy tests and FSH testing.

13.2. Laboratory tests

HbA1c, fasting FPG, fasting serum Insulin, fasting serum C-peptide, 2h-postprandial plasma Glucose, 2h-postprandial serum Insulin, 2h postprandial serum C-peptide, UACR, and PK blood samples need to be sent to the central laboratory for testing according to the Visit schedule.

Lipase and calcitonin were sent to the central laboratory for testing according to the selection of the local laboratory.

13.3. Country-specific variations (not applicable)

Not applicable.

13.4. Protocol amendment history

This study protocol is version 1.3. The revision information of the current version is summarized in "Protocol Revision History and Current Version Revision".

The modification records of the previous version 1.2 are as follows.

Section Number and Name	Description of Change	Brief Rationale for Changes
Protocol Synopsis - Exclusion Criteria<br>5.4. Exclusion criteria	• Exclusion criteria "proliferative retinopathy or maculopathy requiring acute treatment, painful diabetic neuropathy, diabetic foot ulcer, or intermittent claudication at screening" was changed to "proliferative retinopathy or maculopathy, or painful diabetic neuropathy, diabetic foot ulcer, or intermittent claudication requiring acute treatment at screening".	• Considering that GLP-1RA drugs are associated with the risk of retinopathy and maculopathy, such participants were excluded to ensure safety.
Table 1.3 Study Flowchart	• Remove the checkmarks for "Blood Glucose Meter Usage Guidance" and "Participant Diary Usage Guidance" at V15.	• This is an erratum. The double-blind treatment period had ended at V15, and no further guidance related to the double-blind treatment period would be provided.
Table 1.3 Study flowchart footnote<br>8.3.4. Diabetic Retinopathy Screening and Assessment	• Screening and assessment of diabetic retinopathy. At the 32-week inspection, the original inspection population was participants with retinopathy at screening, which was changed to all participants.	• Expand the scope of fundus inspection population to ensure the safety of participants.

Table 1.3 Study flowchart footnote<br>3.1. Objectives and Endpoints<br>8.6. Pharmacokinetics<br>9.6.1. Pharmacokinetic Analysis	• PK testing of metformin was added, and PK sampling points were added for some participants. Corresponding exploratory endpoints and pharmacokinetic analyses were also added.	• Add PK testing to more fully evaluate the relationship between metformin exposure and HRS-7535. And add corresponding exploratory endpoints and pharmacokinetic analysis.
6.8.4. Rescue Treatment	• Rescue treatment "recommended to add basal insulin and sulfonylureas" was changed to "recommended to add basal insulin".	• Insulin is clearly recommended as the preferred medication for rescue treatment to ensure that participants receive rescue treatment safely and effectively.
8.4.9. Adverse events of special interest	• Added adverse event of special interest: lactic acidosis.	• Considering that the background medication in this trial is metformin, the adverse event of special interest related to metformin, lactic acidosis, was added to better ensure the safety of the participants.
	• Corrected editorial errors throughout the body text.	• Corrected editorial errors throughout the body text.

The modification records of the previous version 1.1 are as follows.

Section Number and Name	Description of Change	Brief Rationale for Changes
7.1.1. Discontinuation of trial intervention	• Supplement the description of the discontinuation criteria for trial intervention: 'Persistent and progressively worsening hyperglycemia despite rescue treatment during the treatment period' with 'If the participant still has hyperglycemia judged by the Investigator as excessively high without other inducements within 2 weeks after rescue treatment, discontinuation of trial intervention may be recommended.'	• According to the review opinion of the Institutional Review Board, the description of the discontinuation Phase limit was added for the worsening hyperglycemia.
Table 1.3 Study flowchart<br>8.3.3. 12-lead ECG	• 12-lead electrocardiogram added PR interval data measurement.	• The 12-lead electrocardiogram should measure and collect PR interval data to obtain more complete ECG-related safety data.
8.3.1. Vital Signs and Physical Examination	• Delete the description of "Vital signs must be measured before drug administration."	• Optimize the operation scheme without restricting the relationship between the measurement time of vital signs and drug administration.
8.3.2. Laboratory Determinations	• The term “Urea nitrogen” was modified to “Urea nitrogen or Urea”.	• Some local laboratories at the centers did not have Urea nitrogen test, only Urea test item, and the test item description was improved.

13.5. CKD-EPI formula

CKD-EPI formula estimation:

$$eGFR = a \times \left(\frac{\text{blood creatinine(mg/dL)}}{b}\right)^{c} \times 0.993^{\text{Age}};$$

Gender	a	b	c	
Female	144	0.7	-0.329 (creatinine ≤0.7 mg/dL)	-1.209 (serum creatinine > 0.7 mg/dL)
male	141	0.9	-0.411 (creatinine ≤0.9 mg/dL)	-1.209 (serum creatinine > 0.9 mg/dL)

Blood creatinine: 1 mg/dL = 88.4 μmol/L

Note: eGFR results are rounded down to the nearest whole number.

References:

Levey AS, Stevens LA, Schmid CH, Zhang Y, Castro III AF, Feldman HI, Kusek JW, Eggers P, Van Lente F, Greene T, Coresh J. A new equation to estimate glomerular filtration rate. Annals of internal medicine. 2009 May 5;150(9):604-12.

14. Appendix: abbreviations

ABBREVIATION	Chinese Full Name	Full name in English
ADA	American Diabetes Association	American Diabetes Association
AE	Adverse event	Adverse Event
AESI	Adverse events of special interest	Adverse Event of Special Interest
ALP	Alkaline phosphatase	Alkaline Phosphatase
ALT	Alanine aminotransferase	Alanine Aminotransferase
ANCOVA	Analysis of covariance	Analysis of Variation
AST	Aspartate aminotransferase	Aspartate Aminotransferase
AUC	Area under the plasma concentration-time curve	Area Under the Plasma Concentration-Time Curve
BMI	BMI	Body Mass Index
CI	Confidence Interval	Confidence Interval
CKD-EPI	Chronic Kidney Disease Epidemiology Collaboration	Chronic Kidney Disease Epidemiology Collaboration
Cmax	Peak concentration	Maximum Plasma Concentration
CONSORT	Uniform Standards for Clinical Study Reports	Consolidated Standards of Reporting Trials
CRA	Clinical Study Monitor	Clinical Research Associate
CS	Compound symmetry	Compound Symmetry
DMC	DATA MONITORING COMMITTEE	Data Monitoring Committee
DPP-4	Dipeptidyl peptidase 4	Dipeptidyl Peptidase-4
DTSQ	Diabetes Treatment Satisfaction Questionnaire	Diabetes Treatment Satisfaction Questionnaire
eCRF	Electronic case report form	Electronic Case Report Form
eGFR	Estimated Glomerular Filtration Rate	Estimated Glomerular Filtration Rate
FPG	Fasting Blood Glucose	Fasting Plasma Glucose
FSFV	First Participant First Visit	First Participant First Visit
FSH	Follicle-stimulating hormone	Follicle-Stimulating Hormone
GCP	Good Clinical Practice	Good Clinical Practice
GEE	Generalized estimating equation	Generalized Estimating Equation
GLP-1	Glucagon-like Peptide-1	Glucagon-Like Peptide-1
HbA1c	HbA1c	Glycated Hemoglobin A1C
HBsAg	Hepatitis B surface antigen	Hepatitis B Surface Antigen
HBV	Hepatitis B Virus	Hepatitis B Virus
HDL-C	HDL-C	High Density Lipoprotein - Cholesterol
HOMA-β	Homeostasis model assessment of β-cell function index	Homeostasis Model Assessment of β-cell Function
HOMA-IR	Homeostasis Model Assessment of Insulin Resistance Index	Homeostasis Model Assessment of Insulin Resistance
IB	Investigator’s Brochure	Investigator’s Brochure
ICF	Informed Consent Form	Informed Consent Form
ITT	Intention-to-treat analysis set	Intention-to-treat
IUD	Intrauterine device	Intrauterine Device
IUS	Intrauterine system	Intrauterine System
IWRS	Interactive Web Response System	Interactive Web Response System
LDH	Lactate Dehydrogenase	Lactate Dehydrogenase
LDL-C	Low-density lipoprotein cholesterol	Low-Density Lipoprotein - Cholesterol
LOCF	Last observation carried forward	Last Observation Carried Forward
LSLV	Last Participant Last Visit	Last Participant Last Visit
LYMP	Lymphocyte count	Lymphocyte Count

ABBREVIATION	Chinese Full Name	Full name in English
MedDRA	Medical Dictionary for Regulatory Activities	Medical Dictionary for Regulatory Activities
MEN2	Multiple endocrine neoplasia type 2	Multiple Endocrine Neoplasia Type 2
MMRM	Mixed-effects model for repeated measures	Mixed Model of Repeated Measurement
MRHD	Maximum recommended human dose	Maximum Recommended Human Dose
MTC	Thyroid Medullary Carcinoma	Medullary Thyroid Cancer
MTD	Maximum Tolerated Dose	Maximum Tolerated Dose
NOAEL	No-observed-adverse-effect Level	No Observed Adverse Effect Level
NYHA	New York Heart Association	New York Heart Association
PK	Pharmacokinetics	Pharmacokinetics
PopPK	Population pharmacokinetics	Population Pharmacokinetics
PKCS	Pharmacokinetic Concentration Analysis Set	Pharmacokinetics Concentration Set
QD	Once Daily	Quaque Die (Once Daily)
REML	Restricted estimation maximum likelihood	Restricted Maximum Likelihood
RET	Reticulocyte count	Reticulocyte Count
SAE	Serious adverse event	Serious Adverse Event
SAP	Statistical analysis plan	Statistical Analysis Plan
SGLT2	Sodium-glucose cotransporter-2	Sodium-Glucose Transport Protein 2
SMBG	Self-measured blood glucose	Self-Monitoring of Blood Glucose
SMC	Safety Monitoring Committee	Safety Monitoring Committee
SOP	Standard operating procedures	Standard Operation Procedure
SS	Safety Analysis Set	Safety Set
TBIL	Total Bilirubin	Total Bilirubin
TC	Total cholesterol	Total Cholesterol
TEAE	Treatment-emergent adverse event	Treatment-Emergent Adverse Event
TG	Triglycerides	Triglyceride
UACR	Urine albumin/creatinine ratio	Urine Albumin-to-Creatinine Ratio
ULN	Upper limit of normal range	Upper Limit of Normal
UN	Unstructured	Unstructured
WBC	White blood cell count	White Blood Cell Count

15. Appendix: references

1 Sun H, Saeedi P, Karuranga S, Pinkepank M, Ogurtsova K, Duncan BB, Stein C, Basit A, Chan JC, Mbanya JC, Pavkov ME. IDF Diabetes Atlas: Global, regional and country-level diabetes prevalence estimates for 2021 and projections for 2045. Diabetes research and clinical practice. 2022 Jan 1;183:109119.

2 Magliano DJ, Boyko EJ. IDF Diabetes Atlas 10th edition scientific committee. IDF DIABETES ATLAS [Internet]. 10th ed. Brussels: International Diabetes Federation. 2021.

3 Chinese Diabetes Society. Guideline for the prevention and treatment of type 2 diabetes mellitus in China (2020 edition) [J]. Chinese Journal of Diabetes Mellitus, 2021, 13(4): 315-409.

4 ElSayed NA, Aleppo G, Aroda VR, Bannuru RR, Brown FM, Bruemmer D, Collins BS, Hilliard ME, Isaacs D, Johnson EL, Kahan S. 9. Pharmacologic approaches to glycemic treatment: standards of care in diabetes—2023. Diabetes Care. 2023 Jan 1;46(Supplement_1):S140-57.

5 ElSayed NA, Aleppo G, Bannuru RR, Bruemmer D, Collins BS, Ekhlaspour L, Gaglia JL, Hilliard ME, Johnson EL, Khunti K, Lingvay I. 9. Pharmacologic Approaches to Glycemic Treatment: Standards of Care in Diabetes—2024. Diabetes Care. 2024 Jan 2;47.

6 DeFronzo RA, Ferrannini E, Groop L, Henry RR, Herman WH, Holst JJ, Hu FB, Kahn CR, Raz I, Shulman GI, Simonson DC. Type 2 diabetes mellitus. Nature reviews Disease primers. 2015 Jul 23;1(1):1-22.

7 Gallo LA, Wright EM, Vallon V. Probing SGLT2 as a therapeutic target for diabetes: basic physiology and consequences. Diabetes and Vascular Disease Research. 2015 Mar;12(2):78-89.

8 Dapagliflozin (Forxiga®) Prescribing Information (Revision Date: November 07, 2023)

9 ElSayed NA, Aleppo G, Aroda VR, Bannuru RR, Brown FM, Bruemmer D, Collins BS, Hilliard ME, Isaacs D, Johnson EL, Kahan S. 6. Glycemic targets: standards of care in diabetes—2023. Diabetes care. 2023 Jan 1;46(Supplement_1):S97-110.

A RANDOMIZED, DOUBLE-BLIND, PHASE III STUDY TO EVALUATE THE EFFICACY AND SAFETY OF HRS-7535 COMPARED WITH DAPAGLIFLOZIN IN ADULT SUBJECTS WITH TYPE 2 DIABETES INADEQUATELY CONTROLLED ON METFORMIN THERAPY

Statistical analysis plan

Version No.:	1.0
Version Date	2026-5-26
Company	Jiangsu Hengrui Pharmaceuticals Co., Ltd.

Confidential▲Long-term
This document is proprietary to Jiangsu Hengrui Pharmaceuticals Co., Ltd. and any unauthorized use, disclosure or reproduction in any form is prohibited.
The contents of this document are confidential and proprietary to Jiangsu Hengrui Pharmaceuticals Co., Ltd. Unauthorized use, disclosure or reproduction is strictly prohibited.

Directory:

List of tables and figures 3
ABBREVIATIONS 4
1. Version and amendments 6
2. Introduction 6
    2.1. Study design (trial design) 6
    2.2. Objectives and endpoints (trial objectives and endpoints) 7
    2.3. Estimand 9
        2.3.1. Primary estimand 9
        2.3.2. Supplementary estimand 10
        2.3.3. Secondary estimand 11
    2.4. Sample size Estimation 12
    2.5. Randomization and blinding 13
        2.5.1. Randomization (randomization) 13
        2.5.2. Blinding 13
3. Statistical hypothesis and multiplicity 13
4. Statistical analyses 15
    4.1. General considerations 15
        4.1.1. Analysis sets 15
        4.1.2. General rules 15
        4.1.3. Baseline 17
        4.1.4. Missing data 18
        4.1.5. Rules for intercurrent events 18
        4.1.6. Stratification and subgroup 20
        4.1.7. Analysis window 20
        4.1.8. Analysis period 21
    4.2. Characteristics of participants 22
        4.2.1. Participant distribution 22
        4.2.2. Protocol deviations (protocol deviations) 22
        4.2.3. Summary of analysis sets 22
        4.2.4. Demographics and baseline characteristics 22
        4.2.5. Past medical history 23
        4.2.6. Prior and concomitant medication 23
    4.3. Analysis of efficacy (efficacy analyses) 24
        4.3.1. Primary endpoints analysis 24
        4.3.2. Analysis of key secondary endpoints 26
        4.3.3. Secondary endpoints analysis 27
        4.3.4. Exploratory endpoint analysis 29
        4.3.5. Subgroup analyses 30
        4.3.6. Other analyses 31
    4.4. Safety analysis (safety analyses) 31
        4.4.1. Adverse event (adverse events) 31
        4.4.2. Laboratory test 32
        4.4.3. Vital signs (vital signs) 33
        4.4.4. ECG (electrocardiogram) 33
        4.4.5. Special safety analysis 34
        4.4.6. Other safety analyses 34
    4.5. Drug exposure and compliance 35
    4.6. Other analyses 36
    4.7. Interim analysis (interim analysis) 36
5. Changes to protocol-planned analyses 36
6. References 36
7. Appendices 37

Confidential▲Long-term

This document is proprietary to Jiangsu Hengrui Pharmaceuticals Co., Ltd. and any unauthorized use, disclosure or reproduction in any form is prohibited.

The contents of this document are confidential and proprietary to Jiangsu Hengrui Pharmaceuticals Co., Ltd. Unauthorized use, disclosure or reproduction is strictly prohibited.

LIST OF TABLES AND FIGURES

FIGURE 1 STUDY DESIGN DIAGRAM 7
TABLE 1 DEFINITION OF PRIMARY ESTIMAND AND HANDLING STRATEGIES 9
TABLE 2 DEFINITION OF SUPPLEMENTARY ESTIMAND 2 AND HANDLING STRATEGIES 10
TABLE 3. ESTIMAND SETTING FOR SECONDARY ENDPOINTS 11
TABLE 4 ANALYSIS WINDOW SETTINGS 20
TABLE 5. SUMMARY TABLE OF KEY SECONDARY ENDPOINTS ANALYSIS................................. 26
TABLE 6. SUMMARY OF SECONDARY ENDPOINTS ANALYSIS 27

This document is proprietary to Jiangsu Hengrui Pharmaceuticals Co., Ltd. and any unauthorized use, disclosure or reproduction in any form is prohibited.
The contents of this document are confidential and proprietary to Jiangsu Hengrui Pharmaceuticals Co., Ltd. Unauthorized use, disclosure or reproduction is strictly prohibited.

ABBREVIATIONS

ABBREVIATION	Full name in English	Chinese Full Name
AE	Adverse Event	Adverse event
AESI	Adverse Event of Special Interest	Adverse events of special interest
ANCOVA	Analysis of Covariance	Analysis of covariance
ATC	Anatomic Therapeutic Chemical	Anatomical Therapeutic Chemical Classification System
AUC	Area Under the Curve	Area under the curve
BLQ	Below Limit of Quantitation	Below limit of quantitation
BMI	Body Mass Index	BMI
CI	Confidence Interval	Confidence Interval
CS	Compound Symmetry	Compound symmetry
CV	Coefficient of Variation	Coefficient of variation
eGFR	Estimated Glomerular Filtration Rate	Estimated Glomerular Filtration Rate
FPG	Fasting Plasma Glucose	Fasting Blood Glucose
GEE	Generalized Estimating Equation	Generalized estimating equation
GeoCV	Geometric Coefficient of Variation	Geometric coefficient of variation
GeoMean	Geometric Mean	Geometric Mean
HbA1c	Hemoglobin A1c	HbA1c
HDL-C	High Density Lipoprotein - Cholesterol	HDL-C
HOMA-β	Homeostasis Model Assessment of β-cell Function	Homeostasis model assessment of β-cell function index
HOMA-IR	Homeostasis Model Assessment of Insulin Resistance	Homeostasis Model Assessment of Insulin Resistance Index
ICH	The International Council for Harmonisation of Technical Requirements for Pharmaceuticals for Human Use	International Council for Harmonisation of Technical Requirements for Pharmaceuticals for Human Use
ITT	Intention to Treat	Intention-to-Treat Analysis
LDL-C	Low-Density Lipoprotein – Cholesterol	Low-density lipoprotein cholesterol
LLQ	Lower Limit of Quantitation	Quantitative Lower Limit
MAR	Missing at Random	Missing at Random
Max	Maximum	Maximum value:
MedDRA	Medical Dictionary for Regulatory Activities	Medical Dictionary for Regulatory Activities
MI	Multiple Imputation	Multiple imputation
Min	Minimum	Minimum value:
MMRM	Mixed-Effect Model Repeated Measures	Mixed-effects model repeated measures
PD	Pharmacodynamics	Pharmacodynamics

Confidential▲Long-term
This document is proprietary to Jiangsu Hengrui Pharmaceuticals Co., Ltd. and any unauthorized use, disclosure or reproduction in any form is prohibited.
The contents of this document are confidential and proprietary to Jiangsu Hengrui Pharmaceuticals Co., Ltd. Unauthorized use, disclosure or reproduction is strictly prohibited.

ABBREVIATION	Full name in English	Chinese Full Name
PK	Pharmacokinetics	Pharmacokinetics
PKCS	Pharmacokinetics Concentration Set	PK Concentration Set
PT	Preferred Term	Preferred term
REML	Restricted Estimation Maximum Likelihood	Restricted estimation maximum likelihood
SAE	Serious Adverse Event	Serious adverse event
SAP	Statistical Analysis Plan	Statistical analysis plan
S.D.	Standard Deviation	Standard deviation
SOC	System Organ Classification	System organ class
SMBG	Self-Monitoring of Blood Glucose	Self-measured blood glucose
SS	Safety Set	Safety set
T2DM	Type 2 diabetes mellitus	Type 2 diabetes mellitus
TEAE	Treatment Emergent Adverse Event	Treatment-emergent adverse event
TRAE	Treatment Related Adverse Event	Treatment related adverse event
UACR	Urine Albumin-to-Creatinine Ratio	Urine protein/creatinine ratio
UN	Unstructured	Unstructured
WHODrug	World Health Organization Drug Dictionary	World Health Organization Drug Dictionary

Confidential▲Long-term
This document is proprietary to Jiangsu Hengrui Pharmaceuticals Co., Ltd. and any unauthorized use, disclosure or reproduction in any form is prohibited.
The contents of this document are confidential and proprietary to Jiangsu Hengrui Pharmaceuticals Co., Ltd. Unauthorized use, disclosure or reproduction is strictly prohibited.

1. Version and amendments

The Statistical Analysis Plan (SAP) version 1.0 is based on the latest version of the study protocol (version 1.3, 24 Mar., 2025), describing the statistical analysis plan for efficacy, safety, pharmacokinetics and immunogenicity.

This SAP is the initial version and has no revision history.

SAP Version (date)	Summary of revised content
SAP V1.0（2026-5-26）	Not applicable

2. Introduction

2.1. Study design (trial design)

This trial is a multicenter, randomized, double-blind, active-controlled, parallel-group Phase III clinical trial, including a screening period of up to 2 weeks, a 3-week Run-in period, a 52-week double-blind treatment period, and a 2-week safety follow-up period. The trial population consists of adult subjects with type 2 diabetes who have inadequate glycemic control after metformin treatment on the basis of regular diet and exercise, with a planned enrollment of 800 subjects.

Screening phase (W-5 ~ W-3, up to 2 weeks)

After signing the Informed Consent Form, subjects will undergo screening inspections, and those who meet the inclusion/exclusion criteria may enter the Run-in period.

Run-in period (W-3 ~ W0)

Subjects who pass the screening will start continuous oral administration of single-blind run-in drug for 3 weeks QD from V2, D-21.

The subject will undergo baseline inspection at V3 (D-4), and the inspection at V4 (d1) will be performed to assess whether the subject meets the randomization criteria. Subjects who met the randomization criteria will enter the 52-week double-blind treatment period.

Double-Blind Treatment Period (W1-W52)

Subjects will be randomized in a 1:1:1:1 ratio to the HRS-7535 30 mg group, 60 mg group, 90 mg group and dapagliflozin group, with stratification by baseline HbA1c values (≤8.5%, > 8.5%) measured at the central laboratory. Subjects will start taking double-blind treatment period drugs after randomization.

From the screening phase to the end of the double-blind treatment period, all subjects should

This document is proprietary to Jiangsu Hengrui Pharmaceuticals Co., Ltd. and any unauthorized use, disclosure or reproduction in any form is prohibited.
The contents of this document are confidential and proprietary to Jiangsu Hengrui Pharmaceuticals Co., Ltd. Unauthorized use, disclosure or reproduction is strictly prohibited.

receive stable-dose metformin as background therapy throughout. All subjects should undergo assessments of efficacy and safety-related parameters at the protocol-specified visit time points during the double-blind treatment period.

Safety follow-up phase (W53 ~ W54)

All subjects will discontinue the double-blind treatment period medication, and the subjects will complete the safety follow-up via telephone visit at Week 54. The study design flowchart is shown below:

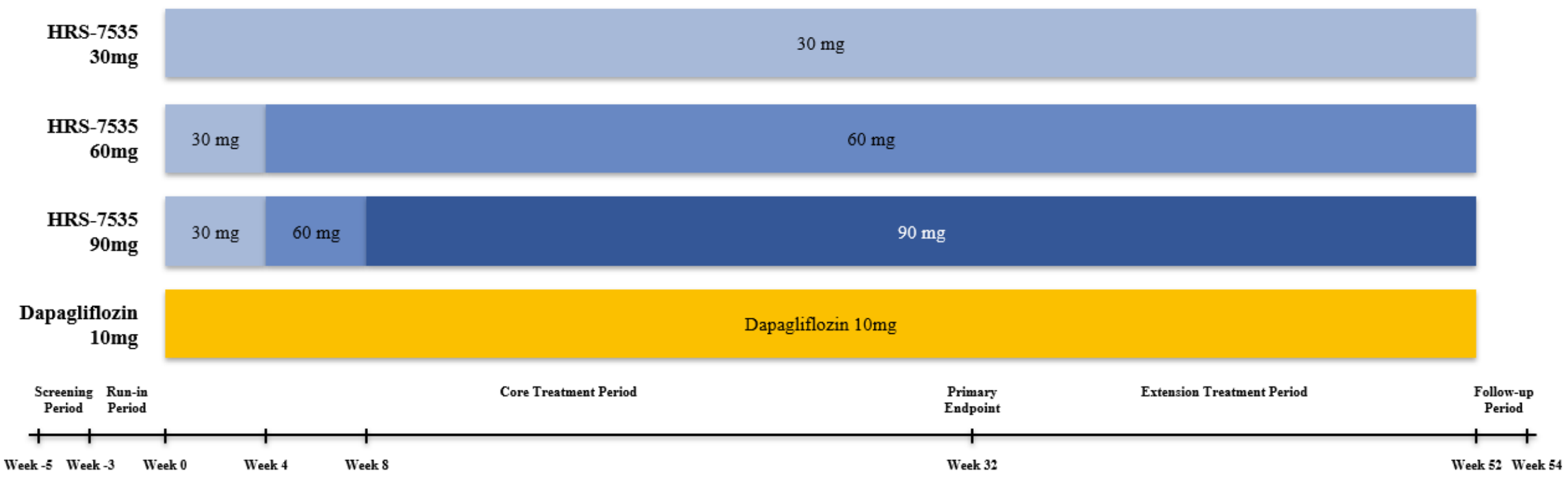

FIGURE 1 STUDY DESIGN DIAGRAM

2.2. Objectives and endpoints (trial objectives and endpoints)

**Primary Objective**	**Primary Endpoint**
To confirm the non-inferiority of HRS-7535 to dapagliflozin in glycemic control efficacy among adult subjects with type 2 diabetes who had inadequate glycemic control after metformin therapy.	The change from baseline in HbA1c at Week 32 of treatment with HRS-7535 compared to dapagliflozin.
**Secondary Objective**	**Secondary Endpoints**
In adult subjects with type 2 diabetes who have poor glycemic control after metformin treatment:<br><br>Compare the efficacy of HRS-7535 and dapagliflozin after 32 weeks of treatment.	Key secondary efficacy endpoints:<br><br>HRS-7535 versus dapagliflozin at Week 32 of treatment:<br>• Proportion of subjects achieving HbA1c < 7.0%<br>• Proportion of subjects achieving HbA1c ≤6.5%<br>• Change from baseline in FPG;<br>• Percentage change from baseline in weight .
In adult subjects with type 2 diabetes who have poor glycemic control after metformin treatment:	Supportive Secondary Efficacy Endpoint:

This document is proprietary to Jiangsu Hengrui Pharmaceuticals Co., Ltd. and any unauthorized use, disclosure or reproduction in any form is prohibited.
The contents of this document are confidential and proprietary to Jiangsu Hengrui Pharmaceuticals Co., Ltd. Unauthorized use, disclosure or reproduction is strictly prohibited.

Comparison of other efficacy indicators between HRS-7535 and dapagliflozin after 32 weeks of treatment.	HRS-7535 versus dapagliflozin at Week 32 of treatment:<br>• Change from baseline in weight and waist circumference ;<br>• Proportion of subjects achieving HbA1c < 5.7%<br>• Changes from baseline in fasting serum insulin, fasting serum C-peptide, homeostasis model assessment of β-cell function (HOMA-β), and insulin resistance index (HOMA-IR);<br>• Changes from baseline in 2-hour postprandial plasma glucose, 2-hour postprandial serum insulin, and 2-hour postprandial serum C-peptide;<br>• Proportion of subjects requiring rescue treatment in each group<br>• Changes from baseline in 7-point SMBG profile.
In adult subjects with type 2 diabetes who have poor glycemic control after metformin treatment:<br>Compare the efficacy of HRS-7535 and dapagliflozin after 52 weeks of treatment.	<u>Supportive Secondary Efficacy Endpoint:</u><br>HRS-7535 versus dapagliflozin at Week 52 of treatment:<br>• Change from baseline in HbA1c;<br>• Change from baseline in FPG;<br>• Percentage Change from baseline in weight;<br>• Change from baseline in weight and waist circumference ;<br>• Proportion of subjects achieving HbA1c < 7.0%<br>• Proportion of subjects achieving HbA1c ≤6.5%<br>• Proportion of subjects achieving HbA1c < 5.7%<br>• Changes from baseline in fasting serum insulin, fasting serum C-peptide, HOMAβ, and HOMA-IR;<br>• Changes from baseline in 2-hour postprandial plasma glucose, 2-hour postprandial serum insulin, and 2-hour postprandial serum C-peptide;<br>• Proportion of subjects requiring rescue treatment in each group<br>• Change from baseline in 7-point SMBG profile.

Confidential▲Long-term
This document is proprietary to Jiangsu Hengrui Pharmaceuticals Co., Ltd. and any unauthorized use, disclosure or reproduction in any form is prohibited.
The contents of this document are confidential and proprietary to Jiangsu Hengrui Pharmaceuticals Co., Ltd. Unauthorized use, disclosure or reproduction is strictly prohibited.

In adult subjects with type 2 diabetes who have poor glycemic control after metformin therapy:<br>Evaluate the safety and tolerability of HRS-7535 and dapagliflozin at 32 weeks and 52 weeks.	Compared with dapagliflozin, at Week 32 and Week 52 of treatment:<br>• Adverse events, hypoglycemic events, laboratory tests, vital signs, physical examination, 12-lead electrocardiogram, diabetic retinopathy screening and assessment.
In adult subjects with type 2 diabetes who have poor glycemic control after metformin therapy:<br>To evaluate the pharmacokinetics (PK) of HRS-7535.	• Plasma HRS-7535 concentration.
In adult subjects with type 2 diabetes who have poor glycemic control after metformin therapy:<br>To assess patient reported outcomes in each treatment group.	• Change from baseline to Week 32 and Week 52 in Diabetes Treatment Satisfaction Questionnaire status version (DTSQs).
**Exploratory Objective**	**Exploratory endpoints**
In adult subjects with type 2 diabetes who have poor glycemic control after metformin treatment:<br>Compare the efficacy of HRS-7535 and dapagliflozin in improving blood pressure, lipid metabolism, and renal function.<br>To evaluate the PK characteristics of metformin when coadministered with HRS-7535	Compared with dapagliflozin, at Week 32 and Week 52 of treatment:<br>• Change from baseline in systolic blood pressure and diastolic blood pressure<br>• Percent change from baseline in TG, TC, LDL-C and HDL-C ;<br>• Percentage change in UACR relative to baseline.<br>• Concentration of metformin in plasma.

Abbreviations: DTSQ = Diabetes Treatment Satisfaction Questionnaire; FPG = Fasting Plasma Glucose; HbA1c = Glycated Hemoglobin; HDL-C = High-Density Lipoprotein Cholesterol; HOMA-IR = Homeostasis Model Assessment of Insulin Resistance; HOMA-β = Homeostasis Model Assessment of β-cell function; LDL-C = Low-Density Lipoprotein Cholesterol; PK = Pharmacokinetics; SMBG = Self-Monitoring of Blood Glucose; TC = Total Cholesterol; TG = Triglycerides; UACR = Urine Albumin/Creatinine Ratio.

2.3. Estimand

2.3.1. Primary estimand

The primary estimand (non-inferiority estimand) for the primary efficacy endpoint (change from baseline in HbA1c at Week 32) consists of the following 5 attributes:

TABLE 1 DEFINITION OF PRIMARY ESTIMAND AND HANDLING STRATEGIES

**Characteristics:**	**Definitions and Strategies**
Treatment	HRS-7535 30 mg, 60 mg, 90 mg or dapagliflozin 10 mg, orally administered, QD
Population	Subjects with type 2 diabetes who have poor glycemic control despite being treated with metformin on the basis of regular diet and exercise control

This document is proprietary to Jiangsu Hengrui Pharmaceuticals Co., Ltd. and any unauthorized use, disclosure or reproduction in any form is prohibited.
The contents of this document are confidential and proprietary to Jiangsu Hengrui Pharmaceuticals Co., Ltd. Unauthorized use, disclosure or reproduction is strictly prohibited.

Characteristics:	Definitions and Strategies
Variable (endpoint)	Change from Baseline in HbA1c at Week 32
Intercurrent events and handling strategies	
Intercurrent event 1: permanent discontinuation of study treatment	Hypothetical strategy: subsequently collected data will not be included in the statistical analysis
Intercurrent event 2: initiation of additional antihyperglycemic medications*	Hypothetical strategy: subsequently collected data will not be included in the statistical analysis.
Summary at the population level	The mean difference in HbA1c change from baseline between the HRS-7535 and dapagliflozin groups at 32 weeks

Abbreviation: HbA1c=glycated hemoglobin; QD=once daily.
*Additional antihyperglycemic medications were defined as any antihyperglycemic medications used cumulatively for more than 14 days.

2.3.2. Supplementary estimand

The handling strategy for Intercurrent events 1-2 of Supplemental Estimand 1 (Treatment policy estimand) adopts the treatment policy strategy, with other attributes and Intercurrent events settings being consistent with the primary estimand.

The five attributes of the supplemental estimand 2 (Efficacy estimand) are as follows:

TABLE 2 DEFINITION OF SUPPLEMENTARY ESTIMAND 2 AND HANDLING STRATEGIES

Characteristics:	Definitions and Strategies
Treatment	HRS-7535 30 mg, 60 mg, 90 mg or dapagliflozin 10 mg, orally administered, QD
Population	Subjects with type 2 diabetes who have poor glycemic control despite being treated with metformin on the basis of regular diet and exercise control
Variable (endpoint)	Change from Baseline in HbA1c at Week 32
Intercurrent events and handling strategies	
Intercurrent event 1: permanent discontinuation of study treatment	Hypothetical strategy: the subsequently collected data will not be included in the statistical analysis
Intercurrent events 2: initiation of additional antihyperglycemic medications*	Hypothetical strategy: the subsequently collected data will not be included in the statistical analysis.
Intercurrent event 3: insufficient drug exposure prior to endpoint	Hypothetical strategy: if the subject had insufficient drug exposure within 90 days before the visit, the data collected at this visit point would not be included in the statistical analysis.
Summary at the Population Level	Mean difference in HbA1c change from baseline between HRS-7535 and dapagliflozin groups after 32 weeks of treatment

Abbreviation: HbA1c=glycated hemoglobin; QD=once daily.
*Additional antihyperglycemic medications were defined as any antihyperglycemic medications used cumulatively for more than 14 days.

This document is proprietary to Jiangsu Hengrui Pharmaceuticals Co., Ltd. and any unauthorized use, disclosure or reproduction in any form is prohibited.
The contents of this document are confidential and proprietary to Jiangsu Hengrui Pharmaceuticals Co., Ltd. Unauthorized use, disclosure or reproduction is strictly prohibited.

For the definition of insufficient drug exposure, please refer to Section 4.1.5.

2.3.3. Secondary estimand

For the key secondary estimand, the attributes of intercurrent events and handling strategies are consistent with supplementary estimand 1. The variables (endpoints) are changed to the corresponding endpoints, and the population-level summary is changed to the between-group difference for the corresponding endpoints.

For the secondary estimand targeting supportive secondary endpoints and exploratory endpoints, the attributes of intercurrent events and handling strategies for the 32-week secondary endpoint are consistent with supplementary estimand 2, with the variable (endpoint) changed to the corresponding endpoint, and the population-level summary changed to the between-group difference for the corresponding endpoint. For the 52-week secondary endpoint, the intercurrent events are updated to events occurring before 52 weeks, with the treatment (handling), population, and strategy consistent with supplementary estimand 2, the variable (endpoint) changed to the corresponding endpoint, and the population-level summary changed to the between-group difference for the corresponding endpoint.

TABLE 3. ESTIMAND SETTING FOR SECONDARY ENDPOINTS

**Endpoint**	**Treatment policy estimand**	**Efficacy estimand**	**No Estimand**
Key secondary efficacy endpoints:<br>HRS-7535 versus dapagliflozin at Week 32 of treatment:<br>• Proportion of subjects achieving HbA1c < 7.0%<br>• Proportion of subjects achieving HbA1c ≤6.5%<br>• Change from baseline in FPG;<br>• Percentage change from baseline in weight.	√	√	
Supportive Secondary Efficacy Endpoint:<br>HRS-7535 versus dapagliflozin at Week 52:<br>• Change from baseline in HbA1c;<br>• Change from baseline in FPG ;<br>• Percentage change from baseline in weight ;	√	√	

This document is proprietary to Jiangsu Hengrui Pharmaceuticals Co., Ltd. and any unauthorized use, disclosure or reproduction in any form is prohibited.
The contents of this document are confidential and proprietary to Jiangsu Hengrui Pharmaceuticals Co., Ltd. Unauthorized use, disclosure or reproduction is strictly prohibited.

• Change from baseline in weight;			
<u>Supportive Secondary Efficacy Endpoint:</u><br>HRS-7535 versus dapagliflozin at Week 32 of treatment:<br>• Change in body weight relative to baseline<br>• Proportion of subjects achieving HbA1c < 5.7%<br>• Changes from baseline in fasting serum insulin, fasting serum C-peptide, homeostasis model assessment of β-cell function (HOMA-β), and insulin resistance index (HOMA-IR);<br>• Changes from baseline in 2-hour postprandial plasma glucose, 2-hour postprandial serum insulin, and 2-hour postprandial serum C-peptide;<br><br><u>Exploratory endpoints</u><br>HRS-7535 versus dapagliflozin at Week 52 of treatment:<br>• Change from baseline in systolic blood pressure and diastolic blood pressure<br>• Percent change in TG, TC, LDL-C and HDL-C from baseline;<br>• Percentage change in UACR relative to baseline.		√	
Other Secondary and Exploratory Endpoints			√

2.4. Sample size Estimation

The sample size calculation is based on the change from baseline in HbA1c at Week 32. Referring to the commonly used margin for HbA1c[1], the non-inferiority margin for treatment difference in this study will be set at 0.4%. Subjects will be randomized into groups at the ratio of 1:1:1:1. Assuming an treatment difference of 0 after considering intercurrent events and a standard deviation of 1.1%, 800 subjects (200 per group) will provide an overall power of 87% to detect non-inferiority of HRS-7535 at all dosage levels (30 mg, 60 mg, 90 mg) compared to the dapagliflozin group at a one-sided significance level of 0.025.

This document is proprietary to Jiangsu Hengrui Pharmaceuticals Co., Ltd. and any unauthorized use, disclosure or reproduction in any form is prohibited.
The contents of this document are confidential and proprietary to Jiangsu Hengrui Pharmaceuticals Co., Ltd. Unauthorized use, disclosure or reproduction is strictly prohibited.

In addition, assuming that the treatment difference between each dosage group of HRS-7535 (30 mg, 60 mg, 90 mg) and the dapagliflozin group after considering intercurrent events is -0.4%, with a standard deviation of 1.1%, then 800 subjects (200 per group) will provide an overall power of 87% to detect the superiority of each dosage group of HRS-7535 (30 mg, 60 mg, 90 mg) over the dapagliflozin group at a one-sided significance level of 0.025.

2.5. Randomization and blinding

2.5.1. Randomization (randomization)

This study adopted a randomized controlled study design, using stratified block randomization. Eligible participants were randomized in a 1:1:1:1 ratio to HRS-7535 30 mg, 60 mg, 90 mg and dapagliflozin groups. The randomization was stratified by HbA1c (≤8.5% vs > 8.5%) during the screening phase.

2.5.2. Blinding

This study is a double-blind and double-dummy study. The project statistical analysts remained blinded throughout the study period and did not perform real group summary analyses until database lock and unblinding.

Blinding must be maintained throughout participant enrollment and administration, documentation and evaluation of study results, inspection of the study process, and data management. The participants, investigators, sponsor's researchers participating in the clinical evaluation, and the clinical research associate (CRA) participating in the study were not aware of which investigational product the subjects received. The third-party laboratory testing personnel were unblinded. To control any potential study bias, the blind codes were properly kept as confidential information to prevent disclosure to the investigators and study staff in direct contact with the investigators until the study database lock was completed.

3. Statistical hypothesis and multiplicity

Let D be the mean difference in the change from baseline in HbA1c at Week 32 between the two treatment groups (HRS-7535 group [90 mg or 60 mg or 30 mg] minus the dapagliflozin group).

When testing the non-inferiority of HRS-7535 relative to dapagliflozin, the null hypothesis (H_0) and alternative hypothesis (H_1) are:

- Non-inferiority: H_0: D ≥0.4% and H_1: D < 0.4%

This document is proprietary to Jiangsu Hengrui Pharmaceuticals Co., Ltd. and any unauthorized use, disclosure or reproduction in any form is prohibited.
The contents of this document are confidential and proprietary to Jiangsu Hengrui Pharmaceuticals Co., Ltd. Unauthorized use, disclosure or reproduction is strictly prohibited.

The fixed sequence testing procedure was performed from high dosage to low dosage. If the upper limit of the two-sided 95% confidence interval (CI) was less than 0.4%, the HRS-7535 group was considered non-inferior to the dapagliflozin group in terms of the primary endpoint. In addition, if the non-inferiority test of the fixed sequence is confirmed, a fixed-sequence testing for superiority from high dosage to low dosage based on a one-sided 0.025 will be conducted for the change from baseline in HbA1c at 32 weeks between HRS-7535 and dapagliflozin.

Hypothesis Testing Sequence and Decision Rules:

A fixed-sequence testing procedure was used to conduct the tests at a one-sided significance level of 0.025 according to the following predefined testing sequence. If the preceding test was confirmed, the subsequent test could be performed. If the preceding test was not passed, the testing would be stopped. Thus, the overall Type I error rate of this study would be controlled within 0.025 (one-sided).

The alternative hypothesis Hxx, where the first subscript 1, 2, and 3 represent the HRS-7535 90 mg, 60 mg, and 30 mg groups, respectively; the second subscript 1-6 represent different endpoints:

- H_{11}, H_{21} and H_{31}: non-inferiority of HRS-7535 90 mg, 60 mg and 30 mg groups versus dapagliflozin group in HbA1c change from baseline after 32 weeks of treatment; Based on the test results of the primary estimand, the clinical question is the comparison between HRS-7535 and dapagliflozin in terms of pure efficacy (with premature discontinuation of treatment and initiation of additional antihyperglycemic medications adopting a hypothetical strategy), following the fixed sequence of H_{11}, H_{21}, and H_{31}.
- H_{12}, H_{22} and H_{32}: superiority of HRS-7535 90 mg, 60 mg, 30 mg groups vs. dapagliflozin in HbA1c change from baseline after 32 weeks of treatment;

  Based on the test results of supplementary estimand 1, the clinical question is the comparison between HRS-7535 and dapagliflozin under conditions consistent with clinical practice (premature discontinuation of treatment and initiation of additional antihyperglycemic drugs using a therapeutic strategy), following the fixed sequence of H_{12}, H_{22}, and H_{32}. Subsequent tests follow the same clinical question and estimand settings.

Confidential▲Long-term
This document is proprietary to Jiangsu Hengrui Pharmaceuticals Co., Ltd. and any unauthorized use, disclosure or reproduction in any form is prohibited.
The contents of this document are confidential and proprietary to Jiangsu Hengrui Pharmaceuticals Co., Ltd. Unauthorized use, disclosure or reproduction is strictly prohibited.

- H_{13}, H_{23} and H_{33}: superiority in the proportion of subjects with HbA1c < 7.0% after 32 weeks of treatment in HRS-7535 90 mg, 60 mg and 30 mg groups vs. dapagliflozin group;
- H_{14}, H_{24} and H_{34}: superiority of the proportion of subjects with HbA1c ≤6.5% after 32 weeks of treatment in HRS-7535 90 mg, 60 mg and 30 mg groups vs. dapagliflozin group;
- H_{15}, H_{25} and H_{35}: superiority of FPG change from baseline at Week 32 in HRS-7535 90 mg, 60 mg and 30 mg groups versus dapagliflozin group;
- H_{16}, H_{26} and H_{36}: superiority of HRS-7535 90 mg, 60 mg and 30 mg groups versus dapagliflozin group in body weight change from baseline after 32 weeks of treatment;

The following hypotheses were tested sequentially at a one-sided significance level of 0.025: H_{11}, H_{21}, H_{31}, H_{12}, H_{22} and H_{32}, H_{13}, H_{23} and H_{33}, H_{14}, H_{24} and H_{34}, H_{15}, H_{25}, H_{35}, H_{16}, H_{26} and H_{36}. If the one-sided p-value of any test exceeded 0.025, the subsequent hypothesis testing was discontinued.

4. Statistical analyses

4.1. General considerations

4.1.1. Analysis sets

The investigational product in the following analysis set definitions refers to HRS-7535 and dapagliflozin, and the investigational drug refers to HRS-7535.

Intention-To-Treat (ITT) set: all randomized participants were analyzed according to the randomized groups.

Safety Set (SS): all participants who received at least one dose of the investigational product constitute the safety analysis set of this study, and the analysis will be performed according to the group assigned at randomization.

PK Concentration Set (PKCS): all participants who received at least one dose of the Investigational Product or metformin and had at least one post-dose PK concentration data.

PK Parameter Set (PKPS): all participants who received at least one dose of HRS-7535 or metformin and had at least one post-dose PK concentration parameter during the intensive sampling phase.

4.1.2. General rules

Study Days:

Confidential▲Long-term
This document is proprietary to Jiangsu Hengrui Pharmaceuticals Co., Ltd. and any unauthorized use, disclosure or reproduction in any form is prohibited.
The contents of this document are confidential and proprietary to Jiangsu Hengrui Pharmaceuticals Co., Ltd. Unauthorized use, disclosure or reproduction is strictly prohibited.

The date of the first administration in the core treatment period was recorded as the study start date.

- Study day = date of the event - date of study start, if before the start of the study.
- Study day = date of the event - date of study start + 1, if on or after the start of the study.

General analysis

Unless otherwise specified, summary of the following descriptive statistics will be given according to the variable type:

- Continuous variables were summarized using number of subjects (n), mean, standard deviation (S.D.), median, minimum (min), and maximum (max).
- Categorical variables will be summarized using frequency and percentage.
- Time-to-event data: the Kaplan-Meier method will be used to estimate the survival function and the median time to event, and the survival curve will be plotted.
- The PK concentration data were summarized by number of subjects (n), n of BLQ, mean (mean), geometric mean (GeoMean), standard deviation (S.D.), coefficient of variation (CV%), geometric coefficient of variation (GeoCV%), median (median), minimum (min), and maximum (max).

Decimal places

The decimal in the analysis report were rounded and presented according to the following rules:

- The decimal places of the minimum and maximum will be consistent with the decimal of the raw data to be collected. The mean and median will have 1 more decimal than the raw data, the standard deviation will have 2 more decimal than the raw data, but a maximum of 2 decimal will be retained.
- Percentages will be rounded to 1 decimal. If the frequency is 0, the percentage will not be displayed.
- The p-value will be retained to four decimals . If the p-value is < 0.0001, it will be reported as “< 0.0001”; if the p-value is > 0.9999, it will be reported as “> 0.9999”.
- The Confidence Interval (CI) will have 1 more decimal place than the raw data, but no more than 2 decimal places.

Unscheduled visit

Confidential▲Long-term
This document is proprietary to Jiangsu Hengrui Pharmaceuticals Co., Ltd. and any unauthorized use, disclosure or reproduction in any form is prohibited.
The contents of this document are confidential and proprietary to Jiangsu Hengrui Pharmaceuticals Co., Ltd. Unauthorized use, disclosure or reproduction is strictly prohibited.

The definition of the efficacy data analysis window is described in Section 4.1.7. For safety data, if descriptive statistical summaries are analyzed according to planned visits, only data from scheduled visit time points will be included, excluding unscheduled visits. If the incidence of abnormalities or the most clinically significant post-baseline changes relative to baseline are summarized using conversion tables, data from unscheduled visit time points will also be included. Unscheduled inspections will also be used to assess baseline. Listings will also present results from unscheduled visits.

Laboratory Tests Results

For continuous laboratory test results, if the record contains special characters (e.g., <xx, >xx), the following rules shall be applied for processing:

1) When <xx or ≤xx occurs, half of the xx value will be taken for analysis.
2) When >xx or ≥xx occurs, the value of xx will be taken for analysis.

Analysis Software

All statistical analyses will be performed in SAS® 9.4 or later versions.

4.1.3. Baseline

For each subject, unless specified otherwise, the baseline is defined as the last non-missing measurement/assessment taken before the first dose of the investigational product during the core treatment period (recorded in the first entry of V4 dosing records), including the day of administration. This included test results from unscheduled visits. If a scheduled visit and an unscheduled visit occur on the same day and it is not possible to determine which happened first, the test result from the scheduled visit is used. The results from the unscheduled visit on the same day are considered as pre-baseline. If there are multiple visit results on the same day (scheduled/unscheduled), the mean value is taken as the test result. For randomized participants who did not receive the drug, the baseline is defined as the last non-missing measurement/assessment taken before randomization (including the day of randomization). This included test results from unscheduled visits. If a scheduled visit and an unscheduled visit occur on the same day and it is not possible to determine which happened first, the test result from the scheduled visit is used. The results from the unscheduled visit on the same day are considered as pre-baseline. If there are multiple visit results on the same day (scheduled/unscheduled), the mean value is taken as the test result.

Confidential▲Long-term
This document is proprietary to Jiangsu Hengrui Pharmaceuticals Co., Ltd. and any unauthorized use, disclosure or reproduction in any form is prohibited.
The contents of this document are confidential and proprietary to Jiangsu Hengrui Pharmaceuticals Co., Ltd. Unauthorized use, disclosure or reproduction is strictly prohibited.

4.1.4. Missing data

The primary methods for handling missing data in this study are as follows:

- Multiple Imputation (MI): detailed steps are described in Section 4.3 Efficacy Analysis Methods.

4.1.4.1. 1. Date missing

Missing date data are imputed in accordance with Hengrui's standard "WI-BD-04-003 Rules for Imputing Missing Dates in Clinical Trial Data V2.0"[2].

4.1.4.2. PK concentration data

Below the Limit of Quantitation (BLQ)

All PK concentrations BLQ: 1) were presented as "<XXX" (XXX is the LLQ value) in listings; 2) for descriptive statistics, BLQ values before the first dose were imputed as 0 while BLQ values after the first dose were imputed as LLQ/2. If more than 50% of the concentration data at a specific sampling time point are LLQ, the concentration data for that time point will only be summarized as n, n of LLQ, and maximum value.

The lower limit of quantitation (LLQ) for HRS-7535 was 2 ng/mL.

Deviations, Missing and Anomalous Values

In statistical summarization analysis, if any of the following conditions occurs, PK concentrations will be treated as missing:

- The concentration value is recorded as not measured or not collected.
- If the actual sampling time for PK concentration exceeds the window, or if the clinical pharmacologist identifies a bias in a particular PK concentration. This data will be annotated in the summary table.

For the list, the actual collected data will be used.

4.1.4.3. Safety data

Missing safety data will not be processed.

4.1.5. Rules for intercurrent events

For the estimand with the endpoint at Week 32, the judgment range of the following intercurrent events was from baseline to the HbA1c test date at Week 32 (or the planned HbA1c test date if missing); for the estimand with the endpoint at Week 52, the judgment range of the

This document is proprietary to Jiangsu Hengrui Pharmaceuticals Co., Ltd. and any unauthorized use, disclosure or reproduction in any form is prohibited.
The contents of this document are confidential and proprietary to Jiangsu Hengrui Pharmaceuticals Co., Ltd. Unauthorized use, disclosure or reproduction is strictly prohibited.

following intercurrent events was from baseline to the HbA1c test date at Week 52 (or the planned HbA1c test date if missing).

Intercurrent Event 1:

Premature discontinuation of treatment: the treatment ends before the endpoint (excluding the day of the endpoint) and the reason for ending is not recorded as “completed”. The event date is the actual date of the last dose. If there is no date of the last dose, the event date is the date of the first dose. For randomized subjects who did not receive the treatment, the event date is the randomization date.

Intercurrent Event 2:

Initiation of additional antihyperglycemic medications: additional antihyperglycemic medications are defined as any antihyperglycemic medications used for a cumulative duration exceeding 14 days. Data were extracted from the "Concomitant Medications" page in EDC ("Reason for Use" = "Prohibited Additional antihyperglycemic medications" and "Rescue medications"). The cumulative 14-day definition refers to the total number of days any additional antihyperglycemic medication was used from randomization to the primary endpoint (regardless of the number of antihyperglycemic medications taken on the same day, it is counted as 1 day). The date of intercurrent events is defined as the date when cumulative medication use reaches 14 days.

Intercurrent Event 3:

Insufficient drug exposure prior to endpoint: for the core treatment period, insufficient drug exposure is defined as the actual total drug exposure within 90 days before the Visit (based on the previous two visits) being less than 50% of the target total exposure, or the number of PK measurements below the lower limit of quantification (BLQ) within 90 days exceeding 50% of the total expected PK measurements (3 times) within 90 days. Starting from V10 (Week 20) Visit, insufficient exposure before the Visit is calculated. If the conditions are met, the analysis value of that Visit is set as missing.

For the extension treatment period, insufficient drug exposure was defined as the actual total drug exposure within 90 days prior to the visit point (HbA1c test date) (based on the previous two visits) being less than 50% of the target total exposure.

The event date for intercurrent events is defined as the earliest occurrence date among all intercurrent events.

This document is proprietary to Jiangsu Hengrui Pharmaceuticals Co., Ltd. and any unauthorized use, disclosure or reproduction in any form is prohibited.

The contents of this document are confidential and proprietary to Jiangsu Hengrui Pharmaceuticals Co., Ltd. Unauthorized use, disclosure or reproduction is strictly prohibited.

4.1.6. Stratification and subgroup

The randomization stratification factors were as follows:

- HbA1c (≤8.5% vs. > 8.5%) at screening

The subgroup variables included:

- Age group (< 65 vs. ≥65);
- Sex (Male vs. Female);
- metformin prolonged-release tablet (≤1500 mg/day vs. > 1500 mg/day);
- Disease course of type 2 diabetes (≤5 years vs.> 5 Years);
- Baseline HbA1c (≤8.5% vs. > 8.5%);
- Baseline BMI (< 24 kg/m^2 vs. ≥24 and < 28 kg/m^2 vs. ≥28 kg/m^2)

4.1.7. Analysis window

For the analysis of efficacy endpoints, the analysis window is defined as shown in Table 4 (based on study days). In addition:

1) If there is a protocol-scheduled visit meeting the criteria within the analysis window, this scheduled visit will be taken as the analysis visit;
2) If multiple assessment results (excluding the assessment of unscheduled visits) fall into the same analysis window, the assessment closest to the scheduled study day in the analysis window will be used for analysis (except baseline analysis; refer to the definition of baseline of each endpoint).
3) Based on 2), if there are still multiple assessment (one before and one after) that are equally close to the study date, the later assessment will be used for analysis. The division of analysis windows after baseline will not consider the assessment of unscheduled visits.
4) If a subject withdraws early from a Visit, the same method of calculating treatment duration will be used to determine the respective visit window for pooled Analysis.

D1 is defined as the date of first dose, and if the subject is randomized but not dosed, D1 is defined as the randomization date.

TABLE 4. ANALYSIS WINDOW SETTING

This document is proprietary to Jiangsu Hengrui Pharmaceuticals Co., Ltd. and any unauthorized use, disclosure or reproduction in any form is prohibited.
The contents of this document are confidential and proprietary to Jiangsu Hengrui Pharmaceuticals Co., Ltd. Unauthorized use, disclosure or reproduction is strictly prohibited.

Scheduled Visit	Analysis Window Label	Scheduled Study Day (day)	HbA1c, FPG, weight, waist circumference, systolic blood pressure, diastolic blood pressure Analysis Window (day)	Serum fasting serum insulin, fasting serum C-peptide, HOMA Analysis Window (day)	2-hour postprandial plasma glucose, 2-hour postprandial serum insulin, and 2-hour postprandial serum C-peptide Analysis Window (day)	TG、TC、LDL-C、HDL-C、Non-HDL-C Analysis Window (day)	UACR Analysis Window (day)
V4	Baseline	D1	<=1	<=1	<=1	<=1	<=1
V6	Week 4	D29	[2, 43]				[2, 43]
V7	Week 8	D57	[44, 71]			[2, 99]	[44,85]
V8	Week 12	D85	[72, 99]				
V9	Week 16	D113	[100, 127]	[2, 169]			[86, 148]
V10	Week 20	D141	[128, 162]			[100, 183]	
V11	Week 26	D183	[163, 204]				[149, 204]
V12	Week 32	D225	[205, 246]	[170, 295]	[2, 295]	[184, 295]	[205, 295]
V13	Week 38	D267	[247, 288]				
V14	Week 44	D309	[289,337]				
V15	Week 52	D364	[338, 394]	[296, 394]	[296, 394]	[296, 394]	[296, 394]

For the derived endpoints HOMA, the data of insulin and glucose within the same planned visit will be used for calculation, and the earliest test date of these indicators will be selected as the test date of HOMA. Then, the analysis window of HOMA will be determined based to this date. No analysis window will be set up for endpoints (including safety endpoints) other than efficacy, and analysis will be performed at each visit based on the actually recorded visit points. For endpoints such as HbA1c and plasma glucose that have both central and local laboratory data, only the central laboratory data will be used in efficacy analysis.

4.1.8. Analysis period

The division of the Run-in period and the core treatment period is based on the date of the first dose at V4 visit. The period before the V4 visit (excluding the day of the visit) is the run-inRun-in period, and the period after the V4 visit (including the day of the visit) is the treatment period. The division of the core treatment period and the extended treatment period is based on the last administration date before V12 (w32). The period before this date (including the same day) is the core treatment period, and the period after this date (excluding the same day) is the extended treatment period. For subjects who did not receive extended treatment, all data will be included in the core period analysis.

Confidential▲Long-term
This document is proprietary to Jiangsu Hengrui Pharmaceuticals Co., Ltd. and any unauthorized use, disclosure or reproduction in any form is prohibited.
The contents of this document are confidential and proprietary to Jiangsu Hengrui Pharmaceuticals Co., Ltd. Unauthorized use, disclosure or reproduction is strictly prohibited.

4.2. Characteristics of participants

The definition of "participant" in this section is the same as "subject". The analysis of participant description is based on ITT.

4.2.1. Participant distribution

Participants were summarized according to the following categories: number of screened subjects, number and reasons for screen failures, number of enrolled subjects, number and reasons for enrollment failures, number of randomized subjects, number of randomized but not dosed subjects, number of treated subjects, number of subjects who discontinued treatment prematurely, number of subjects who completed the study, and number of subjects who withdrew from the study prematurely. Categorized summaries will also be provided for premature treatment discontinuation and withdrawal from the study.

4.2.2. Protocol deviations (protocol deviations)

Prior to database lock, all data will be inspected for major protocol deviations. All potential major protocol deviations will be reviewed and assessed before by the investigator and sponsor . Based on the ITT, all major protocol deviations will be summarized and presented in listings.

4.2.3. Summary of analysis sets

Descriptive summaries of the number of subjects in each analysis set will be provided.

4.2.4. Demographics and baseline characteristics

Based on the ITT, descriptive summaries will be provided on the following demographic and baseline characteristics of participants. Demographic characteristics included age, sex, body weight and body mass index (BMI), and metformin Treatment policy prior to Screening. Baseline characteristics included duration of type 2 diabetes, HbA1c, HbA1c (≤8.5% vs. > 8.5%), fasting plasma glucose, fasting serum insulin, fasting serum C-peptide, homeostasis model assessment of β-cell function (HOMA-β) and insulin resistance (HOMA-IR), 2-hour postprandial plasma glucose, 2-hour postprandial serum insulin, 2-hour postprandial serum C-peptide, blood lipids (non-HDL-C, HDL-C, LDL-C, TC, TG), eGFR, UACR, systolic blood pressure, diastolic blood pressure, heart rate, and waist.

Calculation of age in demographics and baseline characteristics:

Age (years) = (date of Informed Consent Form signing – date of birth + 1) / 365.25, rounded down.

BMI calculation:

BMI (kg/m^2) = body weight (kg) / height $(m)^2$, rounded to 1 decimal.

This document is proprietary to Jiangsu Hengrui Pharmaceuticals Co., Ltd. and any unauthorized use, disclosure or reproduction in any form is prohibited.
The contents of this document are confidential and proprietary to Jiangsu Hengrui Pharmaceuticals Co., Ltd. Unauthorized use, disclosure or reproduction is strictly prohibited.

Calculation of T2DM disease duration:

Duration of type 2 diabetes (years) = (date of signing Informed Consent Form - date of type 2 diabetes diagnosis + 1) / 365.25, rounded to one decimal place.

If the diagnosis date of diabetes is missing, the following rules will be applied for imputation: if only the year of the diagnosis date is recorded, the missing month and day will be imputed as the first day (01 Jan.). If only the year and month are recorded, the missing day will be imputed as the 1st, and the disease duration will be calculated accordingly.

Calculation of HOMA and Non-HDL-C

HOMA-β=[20 × fasting serum Insulin (μU/mL)] / [fasting plasma Glucose (mmol/L) - 3.5]. If fasting plasma glucose ≤ 3.5 mmol/L, HOMA-β will not be calculated .

HOMA-IR= [fasting serum Insulin (μU/mL) × fasting plasma Glucose (mmol/L)] / 22.5

Non-HDL-C = TC（mmol/L） – HDL-C（mmol/L）.

4.2.5. Past medical history

Medical history was coded by system organ class (SOC) and preferred term (PT) using the Chinese version of Medical Dictionary for Regulatory Activities (MedDRA v28.0).

Based on the ITT, descriptive statistics by treatment group will be performed on the medical history (excluded type 2 diabetes) of the subjects .

Listings will be provided for all medical history.

4.2.6. Prior and concomitant medication

Prior and concomitant medications will be coded using the World Health Organization Drug Dictionary Anatomical Therapeutic Chemical (ATC) system.

Prior medication refers to drugs whose administration started before the first dose of the investigational Product; concomitant medications include medications that are administered after the first does of the study drug during the treatment period (but no later than the date of the last administration of the study drug), or that are administered prior to the first administration of the study drug during the treatment period and still ongoing.

Based on SS, past medications and concomitant medications were classified and summarized by ATC2 and preferred term, respectively. In the summary description, if multiple occurrences fall under the same ATC2 or preferred term, they are counted only once. Furthermore, when both start and end dates are completely missing, they are classified as both concomitant and prior medications; if the end date is missing and no continuous administration information is available, it is also considered concomitant medication.

This document is proprietary to Jiangsu Hengrui Pharmaceuticals Co., Ltd. and any unauthorized use, disclosure or reproduction in any form is prohibited.
The contents of this document are confidential and proprietary to Jiangsu Hengrui Pharmaceuticals Co., Ltd. Unauthorized use, disclosure or reproduction is strictly prohibited.

4.3. Analysis of efficacy (efficacy analyses)

All efficacy analyses will be based on ITT. Unless otherwise specified, stratification factors involved in efficacy analyses will be based on the randomization system.

4.3.1. Primary endpoints analysis

The definition of the primary estimand is provided in Section 2.3.1.

4.3.1.1. Primary analysis

Primary analysis of primary estimand:

A hypothetical strategy will be applied to handle both Intercurrent Event 1 and Intercurrent Event 2. Specific analysis steps are as follows:

1) Efficacy data on the change in HbA1c from baseline at Weeks 4, 8, 12, 16, 20, 26, and 32 will be obtained based on the analysis window defined in Section 4.1.7.
2) For subjects who experience intercurrent events 1 and 2, data on or after the date of the earliest Intercurrent Event (using the planned visit date for missing data dates) will be set as missing and handled with the MMRM model under the MAR assumption.
3) The change in HbA1c from baseline at 32 weeks post-treatment will be analyzed using a mixed-effects model for repeated measures (MMRM) based on the restricted estimation maximum likelihood (REML). The MMRM model included baseline HbA1c as a covariate, treatment group, visit, and the interaction between treatment and visit as fixed effects, with within-subject variability modeled using an unstructured (UN) or alternative compound symmetry (CS) covariance matrix. The least squares mean, standard error, 95% confidence interval (CI) of the change from baseline in HbA1c at Week 32 for each treatment group, as well as the least squares mean difference, 95% CI, standard error, and non-inferiority p-value between each HRS-7535 treatment group and the control group will be estimated and presented.

4.3.1.2. Sensitivity analysis

Sensitive analysis 1: for the primary estimand, based on ITT. All missing data were imputed using Multiple Imputation (MI). Analysis was performed using analysis of covariance (ANCOVA) model with baseline HbA1c value as covariate and treatment group as fixed effect.

Sensitive analysis 2: for the primary estimand, based on ITT. The tipping point analysis method was used to impute all missing data, and a series of penalty coefficients (i.e., adding/subtracting a delta value to the imputed data of the experimental group and control group respectively) were set for the experimental group and control group data to reduce the inter-group differences,

This document is proprietary to Jiangsu Hengrui Pharmaceuticals Co., Ltd. and any unauthorized use, disclosure or reproduction in any form is prohibited.
The contents of this document are confidential and proprietary to Jiangsu Hengrui Pharmaceuticals Co., Ltd. Unauthorized use, disclosure or reproduction is strictly prohibited.

with the objective of finding the penalty coefficient (delta value) that would result in inferiority differences between groups. Under each pair of penalty coefficients, the MAR-based MI method was used for multiple imputation, a delta value was added/subtracted to the imputed data, and each imputed dataset was analyzed using the same method as the primary analysis, MMRM. Using Rubin's rule[3], the analysis results of the imputed datasets were combined.

4.3.1.3. Supplementary analysis

The supplementary estimand definition is detailed in section 2.3.2. The superiority test for the primary endpoint was based on the analysis results of supplementary estimand 1.

Intercurrent events 1-2 of supplementary estimand 1 were both treatment policy strategies. For subjects with any intercurrent events, if there are still missing data after adopting the treatment policy strategy, they will be imputed using the RDO (retrieved Dropout) method. If the data does not support the RDO method, the R2B (return to baseline) method will be used for imputation. Other missing data are assumed to be missing at random. The analysis method uses MMRM. Specific analysis steps are as follows:

First, all data were imputed using monotonic imputation, specifically with the MCMC (markov Chain Monte Carlo) method and the IMPUTE=MONOTONE statement to impute the data as monotonic missing data, with the number of imputations m=30 and the seed set to 7535302.

- For missing data of non-intercurrent events, MI imputation was performed under the MAR mechanism. Intercurrent events 1-2 were imputed by RDO and then merged with the remaining data for MMRM analysis. The model used HbA1c baseline value as a covariate, with treatment group, Visit, and treatment-by-visit interaction as fixed effects. Within-subject variability was modeled using UN or alternative CS covariance matrix.
- RDO data refers to the occurrence of intercurrent events 1-2 and successful follow-up to the primary endpoint data. The RDO imputation method refers to the method of using the intercurrent events within the group and collecting the primary endpoint data to impute the missing primary endpoint data after the occurrence of intercurrent events. This method is applied separately for each group and each Intercurrent Event, as follows:

Determine whether the RDO data of the same group reaches 30% of the number of subjects with the Intercurrent Event. If it is reached and the model could be estimated, the RDO method is used for imputation. Specifically, the missing primary endpoint data MI is imputed using the data collected for the same Intercurrent Event within the group, with m=1 and seed set to

Confidential▲Long-term
This document is proprietary to Jiangsu Hengrui Pharmaceuticals Co., Ltd. and any unauthorized use, disclosure or reproduction in any form is prohibited.
The contents of this document are confidential and proprietary to Jiangsu Hengrui Pharmaceuticals Co., Ltd. Unauthorized use, disclosure or reproduction is strictly prohibited.

7535302. If not reached, the R2B method is used for imputation, with m=1 and seed set to 7535302.

Finally, the imputation results of the 30 datasets were combined using Rubin's rules[3]. The least squares mean, standard error, 95% CI of the change from baseline in HbA1c at Week 32 for each treatment group, as well as the least squares mean, 95% CI, standard error, and one-sided p-value for the superiority test of the difference between each HRS-7535 treatment group and the control group were estimated and presented.

Supplemental Estimand 2 adopted hypothetical strategies for intercurrent events 1-3. For subjects experiencing intercurrent events 1-3, data subsequent to the earliest occurrence date (including that day) were set as missing (the date of the missing data used the scheduled visit date). The processed dataset was analyzed using an MMRM model based on REML. The model included baseline HbA1c as a covariate, with treatment group, visit, and treatment-by-visit interaction as fixed effects, and within-subject variability was modeled using an UNor alternative CS covariance matrix. The least squares mean, standard error, 95% CI of the change from baseline in HbA1c at Week 32 for each treatment group, as well as the least squares mean, standard error, one-sided p-value, and 95% CI of the difference between each HRS-7535 treatment group and the control group were estimated and presented.

4.3.2. Analysis of key secondary endpoints

Based on ITT, the analysis of key secondary endpoints adopted two Frameworks: treatment regimen and efficacy estimand, with the analysis methods shown in Table 5.

TABLE 5. SUMMARY TABLE OF KEY SECONDARY ENDPOINTS ANALYSIS

Endpoint	Treatment policy Estimand	Efficacy Estimands
• Proportion of Subjects with HbA1c < 7.0% at Week 32 of Treatment<br>• Proportion of Subjects with HbA1c ≤6.5% at Week 32	The generalized estimating equation (GEE) was used to analyze the measurements from weeks 0 to 32. The model included treatment, visit, and the interaction between treatment and visit as fixed effects, baseline HbA1c as a covariate, and subject as a random effect, with the within-subject variation using an UN or alternative CS covariance matrix. The odds ratio between the two groups, along with its 95% CI and one-sided p-value, was output. After imputing missing HbA1c values according to the rules of supplementary estimand 1, the proportion of subjects with HbA1c < 7.0%/≤6.5% was determined.<br>Superiority was tested based on the p-value of this analysis.	The analysis model for the estimand was the same as that for the therapy, using GEE to analyze the measurements from weeks 0 to 32. Data after intercurrent events were set as missing, and all missing data were handled by the GEE model.

Confidential▲Long-term
This document is proprietary to Jiangsu Hengrui Pharmaceuticals Co., Ltd. and any unauthorized use, disclosure or reproduction in any form is prohibited.
The contents of this document are confidential and proprietary to Jiangsu Hengrui Pharmaceuticals Co., Ltd. Unauthorized use, disclosure or reproduction is strictly prohibited.

• Change from Baseline in FPG at Week 32<br>• percentage change in body weight relative to baseline at 32 weeks of treatment	The measurements from Week 0 to 32 were analyzed using the same analysis method and imputation rules as in supplementary objective 1, namely MMRM analysis with RDO imputation. The model included stratification factors (HbA1c at screening phase (≤8.5% vs > 8.5%)), treatment, visit, and treatment-by-visit interaction as fixed effects, with the corresponding baseline value of each endpoint as the covariate. The within-subject variation was modeled using an UN or alternative CS covariance matrix. The least square mean difference between the two groups was output, along with its 95% CI and one-sided p-value. Superiority was tested based on the p-value of this analysis.	The same MMRM model as the estimand of therapy was used for analysis.<br>Data after intercurrent events were set as missing, and all missing data were handled by the MMRM model.

4.3.3. Secondary endpoints analysis

Based on ITT the estimand framework adopted for each endpoint is described in Section 2.3.3, and the specific analysis methods are shown in Table 6.

TABLE 6. SUMMARY OF SECONDARY ENDPOINTS ANALYSIS

**Endpoint**	**Analytical Method**	**Notes**
At Week 52 of treatment:<br>• Change from Baseline in HbA1c<br>• Change from Baseline in FPG<br>• Percentage change in body weight<br>• Change in body weight relative to baseline	**The Treatment policy estimand framework for therapy:**<br>The measurements from Week 0 to 52 were analyzed, with the analysis method and missing data imputation rules consistent with those for supplementary estimand 1, i.e., MMRM analysis was used, and missing data were imputed with RDO.<br><br>**Efficacy estimand framework:**<br>The measurements from Week 0 to 52 were analyzed using the same method as estimand 2, i.e., MMRM analysis, with data after intercurrent events set as missing and all missing data handled by the model.	
Week 32 of Treatment<br>• Change in body weight relative to baseline<br>• Proportion of subjects achieving HbA1c < 5.7%<br>• Changes from baseline in fasting serum insulin, fasting serum C-peptide, HOMA-β and HOMA-IR<br>• Changes from baseline in 2-	**Efficacy estimand framework:**<br>• Binary variables: the measurements from Week 0 to 32 were analyzed, with the analysis method and imputation rules consistent with those for the binary variables in the key secondary endpoints, i.e., GEE analysis was used, data after intercurrent events were set as missing, and all	Fasting serum insulin, fasting serum C-peptide, 2-hour postprandial serum insulin, 2-hour postprandial serum C-peptide, HOMA β and HOMA-IR will be analyzed using the model at baseline and after log transformation of

This document is proprietary to Jiangsu Hengrui Pharmaceuticals Co., Ltd. and any unauthorized use, disclosure or reproduction in any form is prohibited.
The contents of this document are confidential and proprietary to Jiangsu Hengrui Pharmaceuticals Co., Ltd. Unauthorized use, disclosure or reproduction is strictly prohibited.

Endpoint	Analytical Method	Notes
hour postprandial plasma glucose, 2-hour postprandial serum insulin, and 2-hour postprandial serum C-peptide.	missing data were handled by the model.<br>• Continuous variables with multiple post-baseline measurements:<br>the measurements from Week 0 to Week 32 were analyzed, with the analysis method and imputation rules consistent with those for supplementary estimand 2, i.e., MMRM analysis was used, data after intercurrent events were set as missing, and all missing data were handled by the model.	dependent variables. The model uses log-transformed baseline values as covariates. The antilogarithm of the least squares means and 95% CI for each treatment group output by the model is taken to obtain the ratio of geometric means relative to baseline and its 95% CI for each treatment group.
Other Endpoints	Not based on the estimand framework, descriptive summaries will be performed.	

The 7-point SMBG profile was defined as the area under the curve of the 24-hour glucose profile, specifically calculated as the sum of seven trapezoidal areas divided by 24 hours, unit: mmol/L.

The area of a single trapezoid = sum of the blood glucose measurements at two consecutive points * time difference between scheduled measurements / 2.

Scheduled measurement times are at 7:00 (before breakfast), 9:00 (after breakfast), 12:00 (before lunch), 14:00 (after lunch), 18:00 (before dinner), 20:00 (after dinner), and 22:00 (before sleep). When calculating the last trapezoid area, the last point value is considered the same as the first point, with the next scheduled sampling time defined as the next day at 7:00.

For each visit, the 7-point SMBG measurements at any scheduled sampling time point could be used as the measurement value for that point. If multiple measurements are taken, the mean value of that point is calculated. If all observation point data are missing, the mean of the remaining non-missing data points is used for imputation. If ≥ 3 measurement values are missing at the scheduled measurement times for a visit, the 7-point SMBG data for that visit is considered missing.

The 7-point SMBG analysis does not divide analysis windows and directly uses the visit information recorded in EDC. The 7-point SMBG at each time point of every visit will be described by group and plotted Accordingly.

Patient-Reported Outcome endpoint DTSQs

Confidential▲Long-term
This document is proprietary to Jiangsu Hengrui Pharmaceuticals Co., Ltd. and any unauthorized use, disclosure or reproduction in any form is prohibited.
The contents of this document are confidential and proprietary to Jiangsu Hengrui Pharmaceuticals Co., Ltd. Unauthorized use, disclosure or reproduction is strictly prohibited.

Descriptive statistical analysis will be performed on patient-reported outcome Endpoints, and missing data will not be imputed.

The DTSQs consists of 8 items, including three dimensions: overall treatment satisfaction (Items 1 and 4-8, a total of 6 items measuring the patient's satisfaction with current treatment, convenience, flexibility, satisfaction with their own knowledge of Diabetes, willingness to recommend the current treatment to other patients, and satisfaction with continuing the current treatment), self-perceived hyperglycemia (Item 2), and self-perceived hypoglycemia (Item 3). Items 1 and 4-8 are scored from 0 (Very Dissatisfied) to 6 (Very Satisfied), with a total of 7 score Points. The sum of the scores of these 6 items (Minimum score 0, maximum score 36) comprehensively reflects the Patient's treatment Satisfaction. Items 2 and 3 are scored from 0 (Never) to 6 (Most of the Time), with a total of 7 score Points. Higher scores indicate that the patient perceives more time spent in hyperglycemia or hypoglycemia. Descriptive summaries will be performed for overall treatment satisfaction and scores of each question.

4.3.4. Exploratory endpoint analysis

Descriptive analysis of metformin concentration in plasma.

For the percentage change from baseline in systolic blood pressure and diastolic blood pressure at treatment Week 32/52, the same analysis method as supplementary estimand 2 will be used. The specific estimand framework is shown in Table 3 of chapter 2.3.3.

For the percentage changes from baseline in Triglycerides (TG), Total Cholesterol (TC), Low-Density Lipoprotein Cholesterol (LDL-C), High-Density Lipoprotein Cholesterol (HDL-C), and UACR at Weeks 32/52 of treatment, the same analysis method as in Supplemental Estimand 2 will be applied after log transformation. The estimand framework is shown in Table 3 of section 2.3.3. Specifically, the dependent variable in the MMRM model is the difference between the log-transformed post-baseline value and the log-transformed baseline value of the parameter, with the log-transformed baseline value as a covariate. Treatment group, visit, randomization stratification factors, and the interaction between treatment group and visit are fixed effects. The within-subject error will be calculated using an UN or alternatively a CS covariance matrix. The denominator degrees of freedom for the model will be estimated using the Kenward-Roger approximation. The model will provide the LSMean and 95% CI for the log-transformed changes from baseline at Weeks 32/52 and other visits for each treatment group. These could be back-transformed to obtain the percentage changes from baseline in

Confidential▲Long-term
This document is proprietary to Jiangsu Hengrui Pharmaceuticals Co., Ltd. and any unauthorized use, disclosure or reproduction in any form is prohibited.
The contents of this document are confidential and proprietary to Jiangsu Hengrui Pharmaceuticals Co., Ltd. Unauthorized use, disclosure or reproduction is strictly prohibited.

geometric means for each treatment group, calculated as: $(\exp(\text{LSMean}) - 1) * 100\%$, along with the corresponding 95% CI, calculated as: $(\exp(95\%\ \text{CILimits}) - 1) * 100\%$. Additionally, the model will provide the LSMean and 95% CI for the between-group differences in log-transformed changes from baseline between each investigational drug group and dapagliflozin group at each Visit. These could be back-transformed to obtain the percentage differences in changes from baseline in geometric means between each investigational drug group and the dapagliflozin group, along with the corresponding 95% CI . Missing data were handled based on the MMRM itself.

4.3.5. Subgroup analyses

Based on the ITT, the following subgroup analyses for the primary efficacy endpoint were performed, using the same framework and analytical methods as the primary estimand and supplementary estimand 1.

- Age (< 65 years vs. ≥65 Years);
- Sex (Male vs. Female);
- metformin prolonged-release tablet (≤1500 mg/day vs. > 1500 mg/day);
- Disease course of type 2 diabetes (≤5 years vs. > 5 years);
- Baseline HbA1c (≤8.5% vs. > 8.5%), stratification factor based on EDC records;
- Baseline BMI (< 24 kg/m^2 vs. ≥24 and < 28 kg/m^2 vs. ≥28 kg/m^2).

The analysis method was the same as the primary estimand and supplementary estimand 1, i.e., the MMRM model was used for analysis, and forest plots were drawn.

Based on the efficacy estimand framework (same as the supplementary estimand 2 setting), the analysis will be performed for the following subgroups regarding the key secondary efficacy endpoint of percentage change in body weight from baseline at Week 32 , with the analysis method being the same as that for this key secondary endpoint under the efficacy estimand.

- Sex (Male vs. Female);
- Baseline BMI (< 24 kg/m^2 vs. ≥24 and < 28 kg/m^2 vs. ≥28 kg/m^2);
- Age (≥65 years vs. < 65 years).

Based on the efficacy estimand framework (same as the supplementary estimand 2 setting), the percentage change from baseline in UACR at Week 52 will be analyzed for the following subgroups, with the analysis method being the same as that for this secondary endpoint under the efficacy estimand.

Confidential▲Long-term
This document is proprietary to Jiangsu Hengrui Pharmaceuticals Co., Ltd. and any unauthorized use, disclosure or reproduction in any form is prohibited.
The contents of this document are confidential and proprietary to Jiangsu Hengrui Pharmaceuticals Co., Ltd. Unauthorized use, disclosure or reproduction is strictly prohibited.

- Baseline UACR (≤30 mg/g vs. > 30 mg/g)

4.3.6. Other analyses

Descriptive statistical analyses will be provided for the occurrence of intercurrent events. The assessment window for 32-week/52-week intercurrent events is defined as the period from baseline to the 32-week/52-week HbA1c measurement date (or planned HbA1c measurement date if missing).

4.4. Safety analysis (safety analyses)

The safety analyses will be summarized by treatment group during the treatment period. The definition of treatment period is described in Section 4.1.8. All safety analyses will be based on the SS.

4.4.1. Adverse event (adverse events)

The analysis of AEs was based on the concept of treatment-emergent adverse events (TEAE): all AEs occurring on or after the first day of taking the investigational product were classified as TEAE.

AEs that occurred before the first dose of the investigational product were included in "screening and Run-in period". Such AEs were only listed, and only TEAEs were summarized and analyzed.

TEAEs will be summarized using descriptive statistics and listed by MedDRA System Organ Class (SOC) and Preferred Term (PT), by treatment group and total, and further listed by drug correlation and severity. The descriptive statistical summaries will include, but not be limited to, the following:

- Incidence of AEs, treatment-related AEs ("treatment-related" included "definitely related", "possibly related", "unable to determine" and those with missing relationship to the study drug; treatment-related AEs in the HRS-7535 group were collected from the EDC's relationship with HRS-7535/Placebo, and treatment-related AEs in the dapagliflozin group were collected from the EDC's relationship with dapagliflozin/Placebo), AEs leading to treatment interruption, AEs leading to dose reduced, AEs leading to premature discontinuation of treatment (for the HRS-7535 group, statistics were collected from actions taken on HRS-7535/Placebo, and for the dapagliflozin group, from actions taken on dapagliflozin/Placebo); SAEs, and AESIs (count of subjects: number of subjects who experienced at least one Adverse event).

This document is proprietary to Jiangsu Hengrui Pharmaceuticals Co., Ltd. and any unauthorized use, disclosure or reproduction in any form is prohibited.
The contents of this document are confidential and proprietary to Jiangsu Hengrui Pharmaceuticals Co., Ltd. Unauthorized use, disclosure or reproduction is strictly prohibited.

- Severity of AEs, treatment-related AEs, SAEs, and AESIs (for multiple occurrences of the same AE in the same subject, the most severe instance will be considered for analysis; for different AEs in the same subject, the most severe AE will be considered for analysis);
- AEs and treatment-related AEs with an incidence of ≥ 5%.

Descriptive summaries will be provided for gastrointestinal adverse reactions (including TEAEs by PTs: nausea, vomiting, diarrhoea, constipation, abdominal distension, and decreased appetite), along with summaries by PT and maximum severity, prevalence plots, incidence plots, and listings.

The cumulative incidence of gastrointestinal adverse reactions (including TEAEs by PTs such as nausea, vomiting, diarrhoea, decreased appetite, and abdominal distension) over time will be provided.

Summarize the number and percentage of subjects with hypoglycemia events, the number and percentage of subjects with severe hypoglycemia events, the number of hypoglycemia events, and the number of severe hypoglycemia events.

Listing of all hypoglycemia event results will be provided.

4.4.2. Laboratory test

Laboratory evaluations (including hematology, routine urine, and chemistry) will be analyzed by treatment group, and will include, but not be limited to, the following analyses:

Descriptive summaries will be provided by Item, visit (Distinguishing between V15 and early Withdrawal), and changes from Baseline;

- In the summary by visit using descriptive Statistics, the results of unscheduled visits after baseline were not included in the Analysis.
- For ALT, AST, calcitonin, lipase, amylase, and urine albumin/creatinine ratio (UACR), statistical analyses will be conducted using log transformed values. In descriptive summaries by visit, geometric means and geometric standard deviations will be presented for descriptive statistics.
- The baseline data will be based on the results from the central Laboratory.
- Individual values of eGFR are retained to two decimal , calculation formula

$$\text{eGFR} = a \times \left(\frac{\text{blood creatinine(mg/dL)}}{b}\right)^{c} \times 0.993^{\text{Age}};$$

Confidential▲Long-term
This document is proprietary to Jiangsu Hengrui Pharmaceuticals Co., Ltd. and any unauthorized use, disclosure or reproduction in any form is prohibited.
The contents of this document are confidential and proprietary to Jiangsu Hengrui Pharmaceuticals Co., Ltd. Unauthorized use, disclosure or reproduction is strictly prohibited.

Gender	a	b	c	
Female	144	0.7	-0.329 (creatinine ≤0.7 mg/dL)	-1.209 (serum creatinine > 0.7 mg/dL)
Male	141	0.9	-0.411 (creatinine ≤0.9 mg/dL)	-1.209 (serum creatinine > 0.9 mg/dL)

Blood creatinine: 1 mg/dL = 88.4 μmol/L

- The age used in the formula is the age corresponding to each laboratory inspection date, and the specific calculation rules are as follows:
  Age (years) = (laboratory test date – date of birth + 1) / 365.25, rounded down.

4.4.3. Vital signs (vital signs)

Vital signs data will be analyzed by treatment group and will include, but not be limited to, the following analyses:

- Descriptive statistical summaries will be provided for each visit point for vital signs data (including baseline) and changes in vital signs data from baseline at each post-baseline visit by treatment group;

The number and proportion of subjects with a maximum post-dose pulse > 100, > 110, > 120, and > 130 beats/min will be counted in each treatment group.

In the summary by visit using descriptive statistics, the results of unscheduled visits after baseline were not included in the analysis.

4.4.4. ECG (electrocardiogram)

Based on the Electrocardiogram analysis set, laboratory inspections (hematology, Routine Urine, Chemistry) will be analyzed by treatment group, and will include, but not be limited to, the following analyses:

- Descriptive statistical summaries will be provided for each visit point for ECG data (including baseline) and changes in ECG data from baseline at each post-baseline visit by treatment group;
- The number and proportion of subjects with maximum heart rate > 100, > 110, > 120, and > 130 beats/min after medication in each treatment group will be counted.
- A descriptive statistical summary of the electrocardiogram data (including baseline) of each visit point and the changes from baseline at each visit post-baseline

Confidential▲Long-term
This document is proprietary to Jiangsu Hengrui Pharmaceuticals Co., Ltd. and any unauthorized use, disclosure or reproduction in any form is prohibited.
The contents of this document are confidential and proprietary to Jiangsu Hengrui Pharmaceuticals Co., Ltd. Unauthorized use, disclosure or reproduction is strictly prohibited.

is performed by treatment group. In Addition, the worst clinically significant changes from baseline in ECG post-baseline were analyzed descriptively by treatment group, with the summary method for clinical significance being the same as that for laboratory test.

- The last electrocardiogram data will be used for descriptive summary. In the summary by visit using descriptive statistics, the results of unscheduled visits after baseline were not included in the analysis.

4.4.5. Special safety analysis

Descriptive summaries were provided for Grade 3 hypoglycemia, abnormal liver function, abnormal calcitonin, acute pancreatitis, and lactic acidosis.

4.4.6. Other safety analyses

Descriptive summary of screening and evaluation results for diabetic retinopathy, including the following contents:

- The number of subjects with diabetic retinopathy and diabetic macular oedema in the left and right eyes, as well as the corresponding phases, were summarized according to the planned visit points;
- The number of subjects with diabetic retinopathy in both eyes was summarized: upgrade, no change, downgrade, or undetermined from baseline. This analysis will include data of both scheduled visits and unscheduled visits. The judgment rules for grade changes are as follows:

1) Convert the diabetic retinopathy staging of the left eye and right eye at each visit into scores respectively (the conversion method is shown in Table 6);

2) The scores of the left eye and the right eye were summed to generate the total score for both eyes. If the grade of lesions in both the left and right eyes were undetermined or missing, the total score for both eyes was missing;

3) Select the highest total score of both eyes after baseline and compare it with the total score of both eyes at baseline. If the total score increases, the grade is upgraded; if the total score remains unchanged, the grade remains the same; if the total score decreases, the grade is downgraded. If the total score of both eyes is missing at baseline or any Visit thereafter, the grade change is unassessable.

This document is proprietary to Jiangsu Hengrui Pharmaceuticals Co., Ltd. and any unauthorized use, disclosure or reproduction in any form is prohibited.
The contents of this document are confidential and proprietary to Jiangsu Hengrui Pharmaceuticals Co., Ltd. Unauthorized use, disclosure or reproduction is strictly prohibited.

Table 5. Calculation Table of Single-Eye Diabetic Retinopathy Score

	no lesions	Grade I.	Grade II	Grade III	Grade I-III	Grade IV	Grade V	Grade VI	Grade IV-VI	Unassessable
Score	0	1	2	3	2	4	5	6	5	0

* If the phases of lesions in both the left and right eyes are undeterminable or missing, the total score for both eyes is missing.

List all screening and assessment results for diabetic retinopathy.

The physical examination data were listed.

A listing will be provided for serum pregnancy tests.

4.5. Drug exposure and compliance

Based on the SS, the total drug exposure time, actual total drug exposure, and compliance during the Run-in period and treatment period will be summarized by treatment group. For each dose group of HRS-7535, the administration of HRS-7535/placebo will be calculated, and for the dapagliflozin group, the administration of dapagliflozin/placebo will be calculated. The relevant definitions are as follows:

Total exposure time (days) defined as: date of the last dose - date of the first dose + 1.

Exposure (patient-year)= (total exposure time) / 365.25, rounded.

Total exposure does (mg) is defined as: the cumulative total dosage of the Investigational Product actually received by the subject during the Run-in period or the treatment period.

Planned dose (mg): cumulative total of planned dose per administration (as judged by the Investigator);

Actual dose (mg): the cumulative sum of each actual dose administered.

The date of the last dose in the double-blind treatment period was extracted from the EDC dosing page, taking the last dosing record. If all doses were missed during the dosing period of this record, the date of the last dose was the start date of this dosing -1; in other cases, the date of the last dose was the end date of this dosing.

Descriptive summary of the dosage achieved by each treatment group at each visit was performed, with rules as follows:

Table 6. Dose Judgment Criteria at Each Visit

Dose (mg)	Mean daily dosage (mg) per visit
0	[0,7.5)
15	[7.5,22.5)
30	[22.5,37.5)
45	[37.5,52.5)

This document is proprietary to Jiangsu Hengrui Pharmaceuticals Co., Ltd. and any unauthorized use, disclosure or reproduction in any form is prohibited.
The contents of this document are confidential and proprietary to Jiangsu Hengrui Pharmaceuticals Co., Ltd. Unauthorized use, disclosure or reproduction is strictly prohibited.

60	[52.5,75)
90	≥75

Descriptive summaries of compliance with all groups during the Run-in period and treatment period will be provided by treatment group. Subject compliance (%) = actual dose (tablet)/planned dose (tablet) × 100%.

Compliance will be further categorized into three levels: < 80%, 80-120%, and > 120% for descriptive summary.

Pharmacokinetic (Pk) Analyses

Based on the PKCS, descriptive statistics will be provided for the plasma concentrations of HRS-7535, metformin, and dapagliflozin, by dose group, planned sampling time point, and visit, including the number of subjects, number not less than the lower limit of quantification, mean, standard deviation, median, minimum, maximum, geometric mean, and coefficient of variation (based on geometric mean).

Based on the PKPS, descriptive statistics will be performed for the PK parameters (T_{max}, C_{max} and $AUC_{0-T)}$ of HRS-7535 and metformin in subjects under each HRS-7535 dosage.

All plasma concentration results for subjects will be listed.

PD Analysis:

Not applicable.

Immunogenicity (Ada) Analysis

Not applicable.

4.6. Other analyses

Not Applicable.

4.7. Interim analysis (interim analysis)

None.

5. Changes to protocol-planned analyses

PKPS and its descriptive statistics were Supplemented.

6. References

[1] Center for Drug Evaluation, National Medical Products Administration, Technical Guidelines for Clinical Development of Drugs for Type 2 Diabetes in Adults, 2023

This document is proprietary to Jiangsu Hengrui Pharmaceuticals Co., Ltd. and any unauthorized use, disclosure or reproduction in any form is prohibited.
The contents of this document are confidential and proprietary to Jiangsu Hengrui Pharmaceuticals Co., Ltd. Unauthorized use, disclosure or reproduction is strictly prohibited.

[2] Clinical Trial Date Missing Data Entry Guidelines Document No.: WI-CRD-04-003, Version No.: v1.0, Effective Date: 15 Jun., 2021

[3] Rubin, DB. Multiple Imputation for Nonresponse in Surveys. New York: John Wiley & Sons. 1987

7. Appendices

Example SAS Code for Key Statistical Analyses.

(1) Example code for the MMRM Model:

```
proc mixed data=dataset;
    class treat time subj;
    model y=base treat time treat*time/solution ddfm=kr;
    repeated time/ subject=subj type=un;
    lsmeans treat*time / diff cl;
run;
```

Includes variables used in the analysis, such as treatment group (treat), visit time points (time), and subjects (subj).

model statement:

(a) y is the response variable, i.e., the measured value (the percentage of HbA1c change from baseline in the primary efficacy analysis in this study). The model included base, treat, time, and their interaction treat*time; for indicators requiring logarithmic transformation, replace with log(y) and log(base);

(B) The solution option displays estimates of fixed effects;

(c) The ddfm option specifies the method for calculating denominator degrees of freedom. As the repeated statement specifies type=un, ddfm=kr is specified.

repeated statement:

(a) time is the repeated effect;

(B) The subject option specifies subjects (in this study) in the mixed model, here as subject=subj;

(c) The type option specifies the covariance structure, which in this MMRM analysis is Unstructured (type=un). If the model does not converge, CS is used.

lsmeans statement:

Confidential▲Long-term

This document is proprietary to Jiangsu Hengrui Pharmaceuticals Co., Ltd. and any unauthorized use, disclosure or reproduction in any form is prohibited.

The contents of this document are confidential and proprietary to Jiangsu Hengrui Pharmaceuticals Co., Ltd. Unauthorized use, disclosure or reproduction is strictly prohibited.

(a) lsmeans treat*time tests differences in Least Squares Means between treatment groups across different time points;

(B) The diff option displays differences in Least Squares Means;

(c) The cl option calculates confidence intervals for each Least Squares Mean, defaulting to 95% CI.

(2) Example code for the ANCOVA Model:

```
Proc mixed data=data;
Class trt strata;
Model y = base trt strata/solution;
Lsmeans trt/diff cl;
Run;
```

(3) Example code for multiple imputation based on MAR:

```
Proc mi data=data seed=7535302 nimpute=30 out=data_aftermar;
class trt;
fcs regpmm(aval4= base trt);
fcs regpmm(aval8= base aval4 trt );
fcs regpmm(aval12= base aval4 aval8 trt );
fcs regpmm(aval16= base aval4 aval8 aval12 trt);
var base aval4 aval8 aval12 aval16 trt;
run;
```

seed is the number of seeds used to ensure reproducibility of the imputation results, nimpute specifies the number of imputations, and minimum sets the minimum value after imputation to 0.

Specifies the categorical variables in the var statement.

fcs reg statement: variables like aval4 represent the visit variables converted from longitudinal data in ADaM format to cross-sectional format.

Includes all variables that are part of the imputation model.

(4) Example code for combining analysis results based on Rubin's Rule:

```
/*Output analysis results first*/
proc mianalyze data= CommonPdiffTests;
```


This document is proprietary to Jiangsu Hengrui Pharmaceuticals Co., Ltd. and any unauthorized use, disclosure or reproduction in any form is prohibited.

The contents of this document are confidential and proprietary to Jiangsu Hengrui Pharmaceuticals Co., Ltd. Unauthorized use, disclosure or reproduction is strictly prohibited.

by trt;

modeleffects RiskDifference;

stderr stdErr;run;

parms specifies the dataset containing the analysis results that need to be combined.

modeleffects statement: list the factors in the dataset that need to be analyzed.

Lists the standard errors for the factors being analyzed.

(5) Example code of GEE model:

```
 proc gee data=test;
class trt visit strata2;
model Responder = base trt visit strata2 trt*visit / dist=bin link=logit;
repeated subject= subjid / type=UN covb corrw;
lsmeans trt*visit/diff cl exp;
run;
```

When the data volume is small, it may not converge and have errors. The TYPE could be changed to CS, AR, etc.

Confidential▲Long-term

This document is proprietary to Jiangsu Hengrui Pharmaceuticals Co., Ltd. and any unauthorized use, disclosure or reproduction in any form is prohibited.

The contents of this document are confidential and proprietary to Jiangsu Hengrui Pharmaceuticals Co., Ltd. Unauthorized use, disclosure or reproduction is strictly prohibited.
