# Supplementary material for: Oral small-molecule GLP-1RA safiglipron versus dapagliflozin in type 2 diabetes: a randomized, double-blind, active-comparator-controlled phase 3 trial
Source: Nat Med. 2026 Oct 5;32(11):4340–9. doi: 10.1038/s41591-026-04713-y (PMC13768175; doi:10.1038/s41591-026-04713-y)
Supplement: Supplementary file 2 [file 41591_2026_4713_MOESM2_ESM.pdf]

nature portfolio

Corresponding author(s): Lixin Guo, Zi Ye

Last updated by author(s): Sep 10, 2026

Reporting Summary

Nature Portfolio wishes to improve the reproducibility of the work that we publish. This form provides structure for consistency and transparency in reporting. For further information on Nature Portfolio policies, see our Editorial Policies and the Editorial Policy Checklist.

Statistics

For all statistical analyses, confirm that the following items are present in the figure legend, table legend, main text, or Methods section.

n/a	Confirmed	
☐	☒	The exact sample size (*n*) for each experimental group/condition, given as a discrete number and unit of measurement
☐	☒	A statement on whether measurements were taken from distinct samples or whether the same sample was measured repeatedly
☐	☒	The statistical test(s) used AND whether they are one- or two-sided<br>*Only common tests should be described solely by name; describe more complex techniques in the Methods section.*
☐	☒	A description of all covariates tested
☐	☒	A description of any assumptions or corrections, such as tests of normality and adjustment for multiple comparisons
☐	☒	A full description of the statistical parameters including central tendency (e.g. means) or other basic estimates (e.g. regression coefficient) AND variation (e.g. standard deviation) or associated estimates of uncertainty (e.g. confidence intervals)
☐	☒	For null hypothesis testing, the test statistic (e.g. *F*, *t*, *r*) with confidence intervals, effect sizes, degrees of freedom and *P* value noted<br>*Give P values as exact values whenever suitable.*
☒	☐	For Bayesian analysis, information on the choice of priors and Markov chain Monte Carlo settings
☐	☒	For hierarchical and complex designs, identification of the appropriate level for tests and full reporting of outcomes
☐	☒	Estimates of effect sizes (e.g. Cohen's *d*, Pearson's *r*), indicating how they were calculated

Our web collection on statistics for biologists contains articles on many of the points above.

Software and code

Policy information about availability of computer code

Data collection	Data were collected using the HRTAU EDC (version 4.1.20).
Data analysis	Statistical analyses were performed using SAS (version 9.4).

For manuscripts utilizing custom algorithms or software that are central to the research but not yet described in published literature, software must be made available to editors and reviewers. We strongly encourage code deposition in a community repository (e.g. GitHub). See the Nature Portfolio guidelines for submitting code & software for further information.

Data

Policy information about availability of data

All manuscripts must include a data availability statement. This statement should provide the following information, where applicable:

- Accession codes, unique identifiers, or web links for publicly available datasets
- A description of any restrictions on data availability
- For clinical datasets or third party data, please ensure that the statement adheres to our policy

Qualified researchers may request access to anonymized individual participant data that underlie the results reported in this article. Data will be made available beginning 24 months after primary publication of the trial results. Access will be provided to researchers who provide a methodologically sound proposal for a specific purpose. Proposals should be directed to the corresponding authors: Lixin Guo (glx1218@163.com) or Zi Ye (zi.ye@hengrui.com). Requests will be evaluated by the corresponding authors and sponsor on the basis of scientific merit, methodological feasibility, consistency with participants' informed consent and

protection of participant confidentiality. Applicants will normally be informed of the decision within 8 weeks. Approved access will be subject to execution of a data-use agreement, and data will be shared through a secure data-transfer process. Source data are provided with this paper.

Research involving human participants, their data, or biological material

Policy information about studies with human participants or human data. See also policy information about sex, gender (identity/presentation), and sexual orientation and race, ethnicity and racism.

Reporting on sex and gender	Sex was recorded from the national identification document used for trial registration; gender was not assessed separately. Participants were enrolled regardless of sex. Overall, 521 (64.3%) participants were male and 289 (35.7%) were female. Sex was a prespecified subgroup variable for the primary endpoint, although the trial was not powered for sex-specific treatment-effect comparisons. Sex-disaggregated participant numbers are reported in Table 1.
Reporting on race, ethnicity, or other socially relevant groupings	The trial was conducted in 98 sites in China. The findings may have limited generalizability to other ethnic populations.
Population characteristics	The mean age was 52.2 years, 64.3% of the participants were male, and the mean body weight was 74.7 kg. The median duration of type 2 diabetes was 5.0 years, and the mean HbA1c level at baseline was 8.60%. The baseline demographic and clinical characteristics were generally balanced across the treatment groups.
Recruitment	Potential participants were recruited by investigators at 98 participating hospitals in China and underwent protocol-defined screening after providing written informed consent. Screening began on 27 September 2024, and randomization occurred from 21 October 2024 to 30 April 2025. Of 1,431 individuals screened, 1,079 entered the 3-week single-blind placebo run-in period and 810 were randomized. Due to voluntary, hospital-based recruitment, strict eligibility criteria, and a run-in period, the study cohort may not fully represent the broader clinical population. Enrollment only in China may limit generalizability to other populations. Randomization and double-blinding reduce the risk of bias in treatment comparisons within the enrolled population but do not remove these limitations on generalizability.
Ethics oversight	The protocol, amendments and informed consent materials were approved by the Ethics Committee of Beijing Hospital, the coordinating center, and by the institutional review board or independent ethics committee at each of the 98 participating hospitals listed in Supplementary Table 1. All participants personally provided written informed consent before undergoing any study-specific procedures. Participants received transportation reimbursement and compensation for blood collection, as specified in the ethics committee-approved informed consent materials.

Note that full information on the approval of the study protocol must also be provided in the manuscript.

Field-specific reporting

Please select the one below that is the best fit for your research. If you are not sure, read the appropriate sections before making your selection.

☒ Life sciences ☐ Behavioural & social sciences ☐ Ecological, evolutionary & environmental sciences

For a reference copy of the document with all sections, see nature.com/documents/nr-reporting-summary-flat.pdf

Life sciences study design

All studies must disclose on these points even when the disclosure is negative.

Sample size	The sample-size calculation was based on change from baseline in HbA1c at week 32. A total of 800 participants (200 per group) was planned for randomization. An intercurrent-event incidence of 15% was assumed in sample-size planning. With a common standard deviation of 1.1 percentage points, no true between-group difference and a non-inferiority margin of 0.4 percentage points, this provided approximately 87% overall power to establish non-inferiority for all three safiglipron doses versus dapagliflozin at a one-sided alpha of 0.025. The 0.4-percentage-point margin was prespecified in the protocol. A total of 810 participants were randomized.
Data exclusions	All 810 randomized participants were included in the intention-to-treat population and analyzed according to randomized treatment group. One participant randomized to safiglipron 60 mg did not receive study treatment and was excluded from the safety population according to the prespecified definition. No participants were otherwise excluded from the prespecified analysis populations. Under estimands using a hypothetical strategy, observations on or after qualifying intercurrent events were set to missing as prespecified; this constituted estimand-specific data handling rather than participant exclusion.
Replication	Experimental replication was not applicable to this randomized clinical trial. Reproducibility was supported by the prospectively registered protocol, the prespecified statistical analysis plan and standardized procedures implemented across 98 sites.
Randomization	Participants were randomly assigned (1:1:1:1) to safiglipron 30 mg, 60 mg or 90 mg, or dapagliflozin 10 mg, using a centralized interactive web-response system. The allocation schedule was generated using SAS and used stratified block randomization according to screening HbA1c (≤8.5% versus >8.5%) measured by the central laboratory.
Blinding	Participants, investigators, site staff, sponsor clinical researchers and clinical research associates were masked to treatment assignment; statistical analysts remained masked until database lock. A third-party laboratory team was unmasked as operationally required. Matching tablets supported the double-dummy design. Blinding was maintained throughout the 20-week extension, during which participants remained

in their originally assigned randomized treatment groups.

Reporting for specific materials, systems and methods

We require information from authors about some types of materials, experimental systems and methods used in many studies. Here, indicate whether each material, system or method listed is relevant to your study. If you are not sure if a list item applies to your research, read the appropriate section before selecting a response.

Materials & experimental systems

n/a	Involved in the study
☒	☐ Antibodies
☒	☐ Eukaryotic cell lines
☒	☐ Palaeontology and archaeology
☒	☐ Animals and other organisms
☐	☒ Clinical data
☒	☐ Dual use research of concern
☒	☐ Plants

Methods

n/a	Involved in the study
☒	☐ ChIP-seq
☒	☐ Flow cytometry
☒	☐ MRI-based neuroimaging

Clinical data

Policy information about clinical studies

All manuscripts should comply with the ICMJE guidelines for publication of clinical research and a completed CONSORT checklist must be included with all submissions.

Clinical trial registration	NCT06589765
Study protocol	The full trial protocol and statistical analysis plan are provided as Supplementary Files.
Data collection	Data were collected at 98 clinical sites in China. Screening and randomization took place between 27 September 2024 and 30 April 2025; the last participant completed trial follow-up on 21 May 2026. The trial included screening, a 3-week single-blind placebo run-in, 52 weeks of double-blind treatment and a 2-week safety follow-up. Scheduled study visits occurred at baseline and weeks 2, 4, 8, 12, 16, 20, 26, 32, 38, 44 and 52. Clinical measurements, blood and urine samples, adverse events, concomitant medications and treatment adherence were collected according to the protocol using HRTAU EDC (version 4.1.20).
Outcomes	The primary endpoint was change from baseline in HbA1c at week 32. Non-inferiority was evaluated under the confirmatory primary non-inferiority estimand using a hypothetical strategy for permanent treatment discontinuation and prolonged use of additional antihyperglycemic medication; the endpoint was analyzed using a mixed model for repeated measures. Prespecified superiority testing of the primary endpoint used the treatment-policy estimand within a fixed testing sequence. Prespecified key secondary endpoints at week 32 included HbA1c target achievement, fasting plasma glucose and percentage change in bodyweight and were analyzed under the efficacy estimand. Week-52 efficacy analyses were prespecified as supportive. Safety analyses through week 52 were descriptive and included adverse events, level 1–3 hypoglycemia, laboratory parameters, vital signs and electrocardiography.

Plants

Seed stocks	Not applicable. This study did not use any plant materials or seed stocks.
Novel plant genotypes	Not applicable. No novel plant genotypes (transgenic, gene-edited, mutagenized, or hybridized lines) were generated or used.
Authentication	Not applicable. Plant authentication procedures were not relevant to this study.
